# Advancing Global Health Education: Preparing Emergency Medicine Trainees for Low-Resource Settings Through Simulation-Based Training

**DOI:** 10.15766/mep_2374-8265.11582

**Published:** 2026-03-10

**Authors:** Julianne Jett, Halley J. Alberts, Heather A. Brown, Christopher Gainey, Joshua Skaggs

**Affiliations:** 1 Assistant Director of Simulation, Department of Emergency Medicine, Prisma Health Midlands; 2 Global Health Fellow, Department of Emergency Medicine, Prisma Health Midlands; 3 Director of Global Health, Department of Emergency Medicine, Prisma Health Midlands; 4 Director of Simulation, Department of Emergency Medicine, Prisma Health Midlands; 5 Assistant Professor, Department of Emergency Medicine, Prisma Health Midlands

**Keywords:** Simulation, Global Health, Emergency Medicine, Resource-Limited Setting

## Abstract

**Introduction:**

Global health experiences (GHEs) are increasingly popular among medical trainees. Predeparture training is crucial in preparing learners for the challenges of working in low- and middle-income countries (LMICs). However, few resources are tailored to training level or specialty. This simulation-based course was designed to enhance emergency medicine (EM) trainees’ preparedness for GHEs in LMICs.

**Methods:**

A 4-hour course consisting of 5 small-group simulations followed by a large-group summative lecture designed to reinforce case scenarios was presented to EM residents and medical students. Topics were chosen to represent common, high-acuity conditions in LMICs (e.g., multiple blunt-force trauma, postpartum hemorrhage, pericardial tamponade secondary to tuberculosis, cerebral malaria, intentional organophosphate poisoning). To mirror resource-constrained environments, learners managed each simulated case using limited equipment, diagnostic tools, and medications. A critical action checklist ensured that simulation educational objectives were achieved, and a postcourse survey assessed perceived relevance, realism, and impact on confidence.

**Results:**

Sixteen EM residents and 4 medical students participated. While most learners had previous experience managing postpartum hemorrhage and tension pneumothorax (each 75%), fewer had experience managing pulmonary tuberculosis (25%), malaria (25%), or organophosphate poisoning (15%). Following training, 80% supported inclusion in the residency curriculum annually. Most participants strongly agreed that the training increased confidence in practicing EM in LMICs. Confidence improved in handling all conditions, with the highest increases in confidently managing postpartum hemorrhage and organophosphate poisoning.

**Discussion:**

This EM-specific, simulation-based global health course was well-received and effectively enhanced participants’ confidence and preparedness for GHEs.

## Educational Objectives

By the end of this activity, learners will be able to:

1. Demonstrate knowledge of common etiologies of morbidity and mortality in low- and middle-income countries (LMICs) by verbalizing context-specific differential diagnoses.
2. Build confidence in the diagnosis and management of emergency medical conditions that are relevant in LMICs and resource-limited settings.
3. Demonstrate the ability to adapt and apply innovative management strategies for various pathologies in the context of resource limitations while maintaining an appropriate standard of care.
4. Apply key portions of the workup and treatment of the diseases discussed during simulation debriefing and summative lecture to future patients.

## Introduction

Interest in global health experiences (GHEs) among medical students and residents has significantly increased within the last 2 decades.^1^ This is particularly true among emergency medicine (EM) residents, as EM is an emerging specialty in many countries, and global EM has become its own specialty with 39 active fellowships.^2^ Within the global health context, EM is well suited for emphasis, as the 15 leading causes of death and disability can present as time-sensitive emergent conditions.^3^ Participation in GHEs during residency is correlated with accelerated milestone attainment in nearly every domain, with emphasis on medical knowledge, practice-based learning and improvement, and professionalism milestones.^4^ Additionally, EM residents who do not participate in GHEs still benefit from incorporating aspects of global health into the general EM curriculum.^5^

EM residents planning GHEs in countries where the specialty is underdeveloped may struggle due to resource limitations, cultural differences, and a lack of familiarity with tropical diseases. Despite an increasing focus on predeparture preparation for GHEs, many programs offering GHEs do not provide a formal predeparture training or have listed competency requirements.^6^ No standardized predeparture training for GHEs exists. Those available are often created for a broad audience and not specific to training level or medical specialty.^7^

Simulation has become an integral element in the training of physicians and other medical professionals. Simulation used specifically in predeparture training has been shown to improve trainee's skills, attitudes, and medical knowledge.^7^ Additionally, simulation is the preferred method of instruction for clinical skills training among both undergraduate and graduate health care students preparing for a GHE.^8,9^ A simulation's utility for predeparture training is best explained within the context of Kolb's experiential learning cycle. A simulation case provides a concrete experience for learners. Debriefing provokes reflective observation, followed by abstract conceptualization of the skills and concepts learned during the case. These concepts are then applied and refined in subsequent simulations.

Despite wide availability of published EM simulation scenarios, there remains a paucity of cases focused on global health topics. Of those published cases designed specifically for predeparture training, many focus on ethical dilemmas or managing emotional responses commonly experienced by learners during GHEs.^5,10,11^ While learners find these cases valuable, many want more time focused on hands-on clinical skills.^5^ Other cases present common presentations of chronic illness in residents of low- and middle-income countries (LMICs) that are rarely seen in high-income countries (HICs), such as malnutrition and neglected tropical diseases.^9,12^ While useful, these scenarios have limited applicability to the emergency department (ED) setting. Some published cases focus on disease processes commonly seen in LMICs, but they require high resource utilization that is unavailable in LMICs, limiting their utility for predeparture training.^13–15^ One predeparture simulation course increased participants’ confidence in managing patient conditions, but falls outside of the EM physicians’ scope of practice.^16^

Relevant simulation cases for learners planning a GHE based in the ED of an LMIC would combine high-acuity scenarios grounded in the contextual local burden of disease. Malaria and tuberculosis (TB) are both highly prevalent and among the top 10 causes of death in low-income countries (LICs) but are uncommonly seen in HICs.^17^ Cerebral malaria is most common in children and is a common ED presentation in malaria-endemic regions. Although both pulmonary and extrapulmonary TB comprise a vast number of emergency presentations, extrapulmonary TB is the most common cause of pericardial effusions in LMICs.^18^

Road injuries, maternal causes, and self-harm are among the top 25 causes of death in LICs.^17^ While all 3 of these conditions are common ED presentations globally, the presenting characteristics and management vary greatly depending on the resources available. We chose organophosphate poisoning for the self-harm case, as intentional exposure to pesticides is rarely seen in HICs, but is responsible for 1 in 7 deaths by suicide globally, with higher proportions of deaths found in LMICs.^19^ While road-traffic injuries are prevalent in HICs, 90% of all trauma-related injuries occur in LMICs and cause more deaths globally than HIV, TB, and malaria combined.^20^ EM residents in HICs are acquainted with caring for patients with blunt-force trauma, but doing so without the typical supplies and medications requires innovation. Postpartum hemorrhage is the leading cause of maternal deaths worldwide, but the majority occur in LMICs.^21^ Similar to the requirements for managing trauma, EM residents from HICs may not have the same medications, supplies, or support when managing postpartum hemorrhage in an LMIC.

Recognizing the need for additional formalized global EM education in residency and the simulation's unique ability to meet that need, we developed a course that combines simulation, small-group discussion, and a supplementary summary lecture to prepare trainees for common emergency presentations in LMICs with limited diagnostic and therapeutic options.

## Methods

We created a novel course in the form of 5 small-group simulation scenarios and a large-group summative didactic session. We designed the course to be integrated into our residency program's weekly conference for EM residents and EM-bound medical students. Course authors included 2 global EM fellowship-trained faculty and 2 EM residents with experience in global health. The course authors and a global health fellow facilitated the case debriefings.

### Development

We developed an initial list of case topics based on extensive cumulative experience working in EM settings in LMICs. We then analyzed each topic using the following criteria: disease processes that are prominent causes of mortality in LMICs; disease processes commonly seen in emergency or acute care settings in LMICs; disease processes not commonly seen in emergency settings in HICs; or disease processes commonly seen in emergency settings in HICs but for which diagnosis and management in LMICs varies substantially.

### Equipment/Environment

This training took place within 5 separate rooms in our institution's simulation center. Available patient information, equipment, and medications are detailed in the notes for each case and in Appendix A. Patients were represented by high-fidelity medical simulators with programmable vital signs and physical exam findings. Sophisticated simulators are not required for the successful implementation of these cases. However, if basic mannequins or actors with moulage are used, changes in vital signs and physical exam findings will need to be verbally reported to participants by the case facilitator. The described scenarios were set in hypothetical clinics or hospitals in LMICs with varying degrees of resource limitation.

Scenario 1 (Appendix B) involved management of a patient who experienced blunt-force trauma requiring recognition and evacuation of tension pneumothorax, which learners had to perform without the standard equipment that would typically be available in a high-resource setting. The simulated patient, presenting to a health care center in Nicaragua, was represented by an adult male simulator with an obvious injury to his lower extremity and abrasions over his chest wall. We used a prefabricated simulator leg for the wounded lower extremity, but a moulage kit or a sponge and red paint products could also be used to simulate wounds. A chest tube was not included in the available supplies for this case, requiring learners to construct a makeshift chest tube drainage system with a supplied extra endotracheal tube and empty bottles.^22^ Other possibilities for a makeshift tube could include a central venous catheter or a Foley catheter.^23,24^ A thoracostomy task trainer could be utilized for chest tube placement if available.

Scenario 2 (Appendix C) featured a patient with tuberculous pericarditis requiring pericardiocentesis after decompensating and displaying signs of cardiac tamponade. The scenario was placed at a tertiary care center in Tanzania. The simulated patient was represented by an adult female simulator on a stretcher with glycerin spray to represent diaphoresis. A task trainer and equipment for pericardiocentesis were present in the room but hidden from view.

In scenario 3 (Appendix D), participants diagnosed and managed a simulated case of cerebral malaria in a child presenting with fever, seizures, and coma at a tertiary care center in Malawi. The simulated patient was represented by a high-fidelity pediatric medical simulator with seizure capability. If the learners elected to perform a lumbar puncture, a task trainer was provided, but this could also be accomplished by having participants describe the steps of the procedure.

Scenario 4 (Appendix E) required participants to recognize and treat a case of self-harm in which the patient presented with organophosphate poisoning in a district hospital in Nepal. The simulated patient was represented by an adult male simulator with bradycardia and rales on lung auscultation, with simulated diaphoresis. Commercial products or a variety of homemade formulations can be used to simulate vomit on the patient's clothing. An additional actor was incorporated to prompt learners that the patient had been exposed to fertilizer.

For scenario 5 (Appendix F), participants had to use innovative methods to control a case of refractory postpartum hemorrhage in an unstable patient after typical interventions either failed or were unavailable. This scenario was placed in a district hospital in Uganda. The simulated patient was represented by a high-fidelity obstetrical simulator, and stage blood was used for vaginal bleeding. In our implementation, the facilitator voiced both the patient and the accompanying midwife. Learners were provided an assortment of medical and household supplies, which could be used to craft a provisional balloon tamponade device shown to be effective in resource-limited settings^20^ after other methods failed to control the hemorrhage. Various pharmacologic uterotonics were also provided.

### Personnel

Successful use of each of these simulation cases requires a facilitator who is knowledgeable about the subject, preferably an EM attending with global health experience. Technical support for each case was provided by a simulation technician. Facilitators led the debriefing discussion following each case, reviewing critical actions and key learning points. Most patients were portrayed by high-fidelity patient simulators, with only scenario 4 utilizing a live actor.

### Implementation

In consultation with a simulation fellowship-trained EM faculty member, the course was administered during the residency program's routine weekly didactics. This course was conducted in a hospital-based simulation center, with a 4-hour time allocation for the entire course. Cases took place in simulated exam rooms with varied available equipment and medications, as described in Appendix A, and the individual simulation case templates. Diagnostic imaging, if available to participants as dictated in the case notes, was displayed on a monitor within the exam room.

Participants were divided into 5 groups, with each group composed of members of different training levels. The groups rotated through the simulation cases, with 30 minutes allotted to each case, with a 1:1 simulation-to-debriefing ratio. Facilitators provided direction as indicated by the instructor notes in each case. Each simulation case concluded with a small-group debriefing discussion outlined in the instructor notes and led by the case facilitator. The simulation portion of the course was immediately followed by the didactic presentation described above and included in Appendix G.

### Debriefing

Debriefing discussions based on the PEARLS framework (Promoting Excellence and Reflective Learning in Simulation) were led by case facilitators and intended to summarize the case, provide additional education on the disease or problem presented, and reinforce key educational objectives.^25^ Debriefing outlines were included with the instructor notes for all 5 cases. These guides would be particularly useful for facilitators with limited exposure to the clinical topics addressed within the cases but could be modified at the facilitator's discretion. Additionally, the case facilitators performed a short debriefing following the simulations, which helped identify knowledge gaps needing emphasis in the didactic presentation. There was overlap between the teaching points given by the facilitators and the didactic presentation, which was intended to reinforce the desired teaching points, ensure participant recall of fact material, and focus on specific topics where remediation was required.

### Assessment

Each scenario had multiple critical actions that were required to be completed to pass the scenario. Facilitators administering the cases ensured that participants met all the critical actions by utilizing mechanisms such as adjusting the patient condition, enlisting embedded participants to supply additional information, or directly prompting trainees to meet those critical actions. Trainees had to utilize the available equipment in each scenario room to successfully treat the patient's emergent condition.

Immediately following completion of the course, participants were given voluntary anonymous postparticipation paper questionnaires (Appendix H). Participants indicated their training level, previous global health experience in medical school or residency, plans for future global health experience, and previous experience with the conditions presented in the course. Five-point Likert scales (1 = *Strongly disagree*, 5 = *Strongly agree*) were used to assess participants’ attitudes regarding the quality of the training and its effect on their confidence in diagnosing and managing the conditions presented. The perception of future ability to practice medicine in resource-limited settings and the relative importance of global health simulation cases was also queried. The study was reviewed by the Prisma Health Institutional Review Board and designated as exempt (Prisma Health No. 2023643-1, March 3, 2023).

## Results

### Participant Training Level and Experience

**Figure 1.**
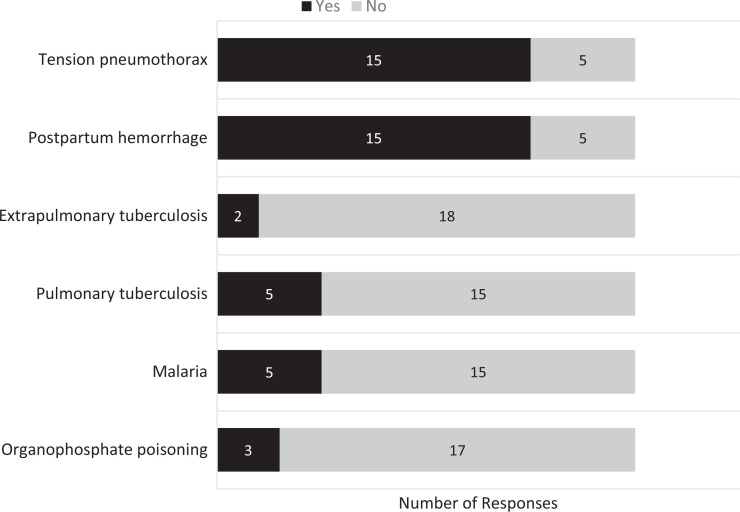
Participants’ responses (*N* = 20) on postparticipation surveys indicating if they had prior experience with the conditions presented in the course preparing emergency medicine trainees for low-resource settings through simulation-based training.

The 20 participants included 16 residents and 4 fourth-year medical students. All participants completed the postparticipation questionnaire, for a 100% response rate. Of the 16 resident participants, 6 were PGY 3 students, 4 were PGY 2, and 6 were PGY 1. Eight participants reported participation in GHEs during medical school. Only 2 had participated in a GHE during residency. Ten residents had not participated in GHEs during residency but had plans to do so. The number of participants with previous experience in caring for patients with the pathologies presented in each case is illustrated in Figure 1.

### Case Scenarios

The 5 groups that rotated through the scenarios performed all critical actions for the blunt-force trauma patient. While all groups recognized pericardial tamponade, this was not easily associated with the underlying context of being related to TB. Two of the 5 groups never considered malaria as a cause of their patient's seizure. The organophosphate overdose was the most commonly missed diagnosis of the simulation scenarios. Critical actions were met by all groups for the postpartum hemorrhage case, although equipment improvisation to achieve uterine tamponade required additional discussion by the facilitator. For multiple cases, trainees initially developed incorrect initial working diagnoses, including high-altitude sickness or sepsis for organophosphate poisoning, and pulmonary embolism or bacterial pneumonia for the TB case.

### Course Evaluation

**Figure 2.**
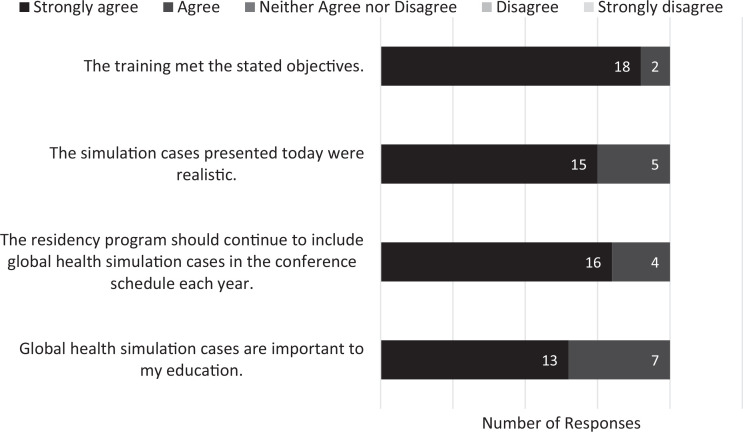
Participants’ responses (*N* = 20) on postparticipation surveys indicating their level of agreement with statements about the importance of global health simulation cases and the quality of the training.

Thirteen participants (65%) strongly agreed and 7 (35%) agreed that global health simulation cases are important to their education. Sixteen participants (80%) strongly agreed and 4 (20%) agreed that the cases should be included in the residency program's conference schedule annually. Fifteen participants (75%) strongly agreed and 5 (25%) agreed that the simulation cases were realistic (Figure 2).

**Figure 3.**
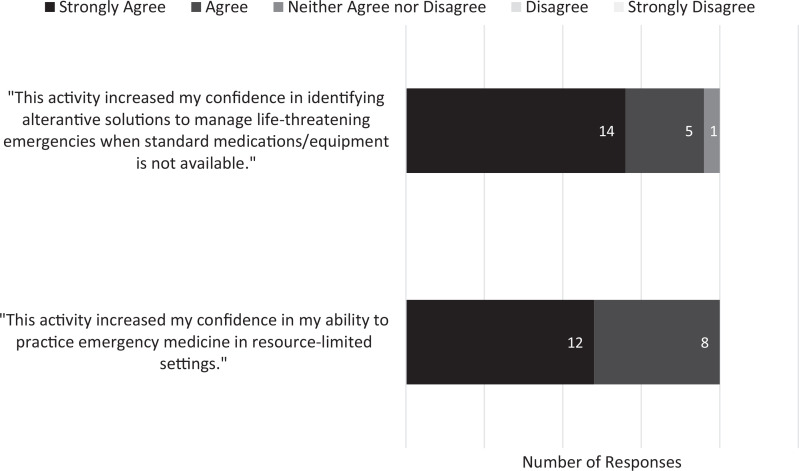
Participants’ responses (*N* = 20) on postparticipation surveys indicating their level of agreement with statements regarding self-confidence in their ability to work in resource-limited settings and their ability to identify alternative solutions to manage life-threatening emergencies when standard resources are not available.

Twelve participants (60%) strongly agreed and 8 (40%) agreed that the activity increased confidence in their ability to practice EM in resource-limited settings. Fourteen participants (70%) strongly agreed and 5 (25%) agreed that the activity increased their confidence in managing life-threatening emergencies without standard medications and equipment (Figure 3).

**Figure 4.**
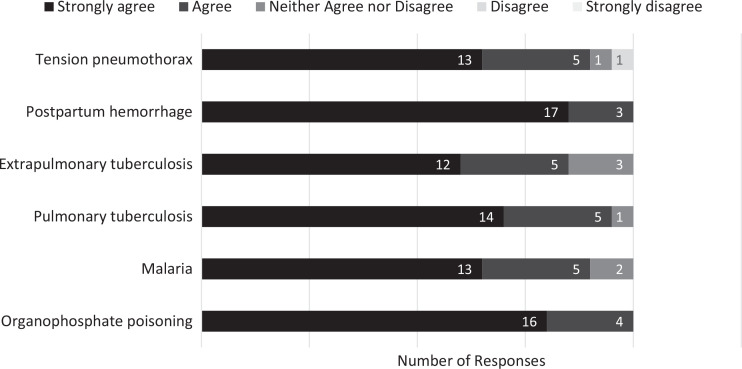
Participants’ responses (*N* = 20) on postparticipation surveys to the question, “This activity increased my confidence in diagnosing and managing patients with…”, indicating their level of agreement with statements regarding their self-confidence in diagnosing and managing patients with the conditions presented in the course.

Participants tended to strongly agree that the activity increased their confidence in diagnosing and managing patients with organophosphate poisoning, malaria, TB, postpartum hemorrhage, and tension pneumothorax, as illustrated in Figure 4.

## Discussion

This project exposed trainees to practicing EM in alternative environments with a goal of increasing their comfort and proficiency during clinical rotations abroad. The cases provided hands-on experience with potential challenges of working in LMICs, examining pathologies seen infrequently in HIC settings, and exploring alternative uses for supplies in resource-limited settings. The summative lecture expanded on clinical knowledge and allowed for large-group discussions on themes in mental health, women and infant care, and global discrepancies in health care access. The course was overall well received by participants, and the results of the postparticipation survey suggest that it was a relevant and beneficial addition to their education.

While many participants cited prior experience with tension pneumothorax and postpartum hemorrhage, the challenge in these cases was to identify alternative management strategies in the absence of standard equipment by adapting available resources. Conversely, few participants reported previous experience caring for patients with organophosphate poisoning, malaria, or TB, and knowledge gaps in these areas were exposed during the simulation portion of the course.

Despite malaria being a leading cause of global morbidity, only one-quarter of the trainees attending this course endorsed previous experience in evaluating or treating malaria. Inexperience with TB was also evident, with most groups considering other etiologies for dyspnea that are more frequently encountered in HIC EDs. Sepsis was a frequent working diagnosis in the pesticide toxicity case, with availability bias likely playing a role, as sepsis is more familiar to trainees.

This course's contribution lies in its focus on global EM, with the use of simulation to address constraints and conditions prevalent in LMICs. The feedback indicated a significant increase in participants’ confidence and preparedness for managing emergencies in low-resource settings. This innovation contributes to the existing literature by addressing the gap in practical training for global health emergencies and resource-constrained environments. Addressing these gaps helps prepare learners for future GHEs and clinical situations where standard resources may not be available. Feedback indicated that participants felt more prepared to manage emergencies in low-resource settings and recognized the importance of adaptive problem-solving. Participants seemed to particularly enjoy the innovative aspects of the cases that encouraged them to brainstorm with their team and think outside of the box.

This course was case-based and limited to 5 common presentations. As disease pathology and resource availability is dependent on the location, this does limit training applicability to the specific global health context. Although the scenarios are designed to be adaptable, the simulations rely on specific equipment and medical supplies that may not be universally available, potentially limiting their effectiveness in settings with different resource constraints. The effectiveness of the simulations and debriefing sessions is dependent on the facilitators’ expertise. In cases where facilitators lack in-depth knowledge or experience in global health or the specific conditions covered, the quality of instruction and feedback could be inconsistent. To address this, written guides were provided ahead of time to standardize the debriefing experience. Additionally, the summative lecture was intended to reinforce the scenario topics and give learners the opportunity to discuss their understanding of the content. The course evaluation was limited by an overall low number of participants and lack of direct measurement of knowledge gain. We included both medical students and all levels of residents in the course and evaluation; however, their results may not be directly comparable given their varying levels of competence. Additionally, the only course evaluation was performed directly following the course and therefore no direct evaluation of how the course impacts knowledge retention or behavior during participants’ GHEs was measured.

Measuring changes in participant behavior or knowledge retention following the training should be considered in future implementation. Future directions could include a wider variety of scenarios representing a broader spectrum of global health pathologies or emerging infections. Incorporating precourse materials on less familiar conditions, global disease burdens, and social determinants of health could better prepare participants before the simulations and should be considered.

This course addresses a gap in traditional EM training by providing focused simulation experiences that specifically prepare trainees for the unique challenges encountered in LMICs. It integrates practical problem-solving with resource constraints in the setting of global health–specific pathology, not commonly emphasized in HIC-based training programs.

## Appendices


Equipment for Implementation.docxTraumatic Hemopneumothorax Case.docxTuberculous Pericarditis Case.docxCerebral Malaria Case.docxOrganophosphate Poisoning Case.docxPostpartum Hemorrhage Case.docxLecture.pptxCourse Evaluation.docx
*All appendices are peer reviewed as integral parts of the Original Publication.*

## References

[1] Greyson SR, Richards AK, Coupet S, Desai MM, Padela AI. Global health experiences of U.S. physicians: a mixed methods survey of clinician-researchers and health policy leaders. Global Health. 2013;9:19. 10.1186/1744-8603-9-19 23663501 PMC3655883

[2] GEM Fellowships Current Programs. Global Emergency Medicine Fellowship Consortium. Accessed August 23, 2024. https://www.saem.org/GEMFC/gem-fellowships-current-programs

[3] Shanahan T, Risko N, Razzak J, Bhutta Z. Aligning emergency care with global health priorities. Int J Emerg Med. 2018;11(1):52. 10.1186/s12245-018-0213-8 31179932 PMC6326121

[4] Hayward AS, Lee SS, Douglass K, et al. The impact of global health experiences on the emergency medicine residency milestones. J Med Educ Curric Dev. 2022;9:23821205221083755. 10.1177/23821205221083755 35572845 PMC9102119

[5] Pirrocco F, Goodman I, Pitt MB. Leveraging peer teaching for global health preparation: Implementation of a resident-led global health simulation curriculum. Glob Pediatr Health. 2019;6:2333794X19851108. 10.1177/2333794X19851108 PMC6537236 31205986

[6] Tupesis JP, Babcock C, Char D, Alagappan K, Hexom B, Kapur GB. Optimizing global health experiences in emergency medicine residency programs: a consensus statement from the Council of Emergency Medicine Residency Directors 2011 Academic Assembly global health specialty track. Int J Emerg Med. 2012;5(1):43. 10.1186/1865-1380-5-43 23148459 PMC3518176

[7] Kalbarczyk A, Nagourney E, Martin NA, Chen V, Hansoti B. Are you ready? A systematic review of pre-departure resources for global health electives. BMC Med Educ. 2019;19(1):166. 10.1186/s12909-019-1586-y 31118015 PMC6532266

[8] Kironji AG, Cox JT, Edwardson J, et al. Pre-departure training for healthcare students going abroad: Impact of preparedness. Ann Glob Health. 2018;84(4):683–691. 10.9204/aogh.2378 30779518 PMC6748281

[9] Schwartz KR, Prentiss KA. Simulation in pre-departure training for residents planning clinical work in a low-income country. West J Emerg Med. 2015;16(7):1166–1172. 10.5811/westjem.2015.9.28164 26759672 PMC4703167

[10] Hau DK, Howell JD, Ayeni A, Alfonzo MJ, Ching K. Child death in a resource-limited setting: A simulation case for pediatric residents to prepare for global health electives. MedEdPORTAL. 2023;19:11341. 10.15766/mep_2374-8265.11341 37662497 PMC10471738

[11] Asao S, Lewis B, Harrison JD, et al. Ethics simulation in global health training (ESIGHT). MedEdPORTAL. 2017;13:10590. 10.15766/mep_2374-8265.10590 30800792 PMC6338194

[12] Mankbadi M, Goyack L, Thiel B, Weinstein D, Simms-Cendan J, Hernandez C. Dermatologic simulation of neglected tropical diseases for medical professionals. MedEdPORTAL. 2016;12:10525. 10.15766/mep_2374-8265.10525 30984867 PMC6440398

[13] Kestler A, Kestler M, Morchi R. Severe malaria simulation scenario. MedEdPORTAL. 2009;5:1740. 10.15766/mep_2374-8265.1740

[14] Brooks J, Zosel A, Anderson B. Organophosphate poisoning simulation case. MedEdPORTAL. 2010;6:7952. 10.15766/mep_2374-8265.7952

[15] Hart D, Gorlin J. Massive transfusion for postpartum hemorrhage. MedEdPORTAL. 2013;9:9651. 10.15766/mep_2374-8265.9651

[16] Kynes JM, Kauffman R, Walters CB, Sizemore C, Banerjee A. The preparing residents for international medical experiences (PRIME) simulation workshop: equipping surgery and anesthesia trainees for international rotations. MedEdPORTAL. 2021;17:11088. 10.15766/mep_2374-8265.11088 33598534 PMC7880254

[17] GBD 2021 Causes of Death Collaborators. Global burden of 288 causes of death and life expectancy decomposition in 204 countries and territories and 811 subnational locations, 1990–2021: a systematic analysis for the Global Burden of Disease Study 2021. Lancet. 2024;403(10440):2100–2132. 10.1016/S0140-6736(24)00367-2 38582094 PMC11126520

[18] Ntsekhe M. Pericardial disease in the developing world. Can J Cardiol. 2023;39(8):1059–1066. 10.1016/j.cjca.2023.05.005 37201721

[19] Mew EJ, Padmanathan P, Konradsen F, et al. The global burden of fatal self-poisoning with pesticides 2006-15: systematic review. J Affect Disord. 2017;219:93–104. 10.1016/j.jad.2017.05.002 28535450

[20] Whitaker J, O'Donohoe N, Denning M, et al. Assessing trauma care systems in low-income and middle-income countries: a systematic review and evidence synthesis mapping the Three Delays framework to injury health system assessments. BMJ Glob Health. 2021;6(5):e004324. 10.1136/bmjgh-2020-004324 PMC8118008 33975885

[21] World Health Organization. Trends in Maternal Mortality 2000 to 2020: Estimates by WHO, UNICEF, UNFPA, World Bank Group and UNDESA/Population Division. World Health Organization; 2023. Accessed February 7, 2026. https://iris.who.int/server/api/core/bitstreams/c3957b94-cdd5-47d7-85f8-6202be229f8e/content

[22] Beer RG, Grimmett WG, Fraser JF. Appraisal of the endotracheal tube as an alternative to the intercostal catheter. Emerg Med Australas. 2010;22(6):573–574. 10.1111/j.1742-6723.2010.01359.x 21143408

[23] Singh K, Loo S, Bellomo R. Pleural drainage using central venous catheters. Crit Care. 2003;7(6):R191–R194. 10.1186/cc2393 14624695 PMC374384

[24] Lai Y, Wang X, Zhou H, Kunzhou PL, Che G. Is it safe and practical to use a Foley catheter as a chest tube for lung cancer patients after lobectomy? A prospective cohort study with 441 cases. Int J Surg. 2018;56:215–220. 10.1016/j.ijsu.2018.06.028 29936194

[25] Bajaj K, Meguerdichian M, Thoma B, Huang S, Eppich W, Cheng A. The PEARLS healthcare debriefing tool. Acad Med. 2018;93(2):336. 10.1097/ACM.0000000000002035 29381495

