# Supplementary material for: Advancing Global Health Education: Preparing Emergency Medicine Trainees for Low-Resource Settings Through Simulation-Based Training
Source: MedEdPORTAL. 2026 Mar 10;22:11582. doi: 10.15766/mep_2374-8265.11582 (PMC12972016; doi:10.15766/mep_2374-8265.11582)
Supplement: Supplementary file 1 — Equipment for Implementation.docxTraumatic Hemopneumothorax Case.docxTuberculous Pericarditis Case.docxCerebral Malaria Case.docxOrganophosphate Poisoning Case.docxPostpartum Hemorrhage Case.docxLecture.pptxCourse Evaluation.docx [file mep_2374-8265.11582-s001.zip › A. Equipment for Implementation.docx]

Appendix A. Equipment for Implementation

Purpose: This appendix lists the equipment, supplies, medications, and diagnostic tools required to implement each simulation case.

When and How to Use: Facilitators should review this appendix during case preparation to assemble materials prior to the simulation session. Items marked with an asterisk (*) are available upon learner request during the scenario. When needed, materials may be substituted with locally available or low-fidelity alternatives.

| Case | Simulation Equipment | Available Medical Equipment^*^ | Available Medications^*^ | Available Diagnostic Tools^*^ |
| --- | --- | --- | --- | --- |
| 1 - Trauma | Adult simulation mannequin  Moulage leg with large wound  Stretcher  Cardiac monitor  Thoracostomy task trainer | Cardboard splint  C-collar  Direct laryngoscopy  2 Endotracheal tubes  3 empty water bottles  IV tubing  Suction tubing  Tape | 0.9% Normal Saline or Lactated Ringers  Ceftriaxone | Glucometer  Point-of-care ultrasound |
| 2 – TB Pericarditis | Adult simulation mannequin  Stretcher  Cardiac monitor  Glycerin spray for diaphoresis  Pericardiocentesis task trainer | Nasal cannula  Oxygen  Pericardiocentesis kit (or 18 gauge spinal needle, 3 way stopcock and large syringe) | Furosemide  Prednisolone  Rifampin  Isoniazid  Pyrazinamide  Ethambutol  Ceftriaxone  0.9% NS or LR  Standard ACLS medications | Glucometer  Point-of-care ultrasound  Chest x-ray  EKG  CBC  BMP  Troponin level |
| 3 – Cerebral Malaria | Child simulation mannequin  Stretcher  Cardiac monitor | Nasal cannula  Oxygen  Simple face mask  Suction | Acetaminophen  Rectal diazepam  D10W  3% hypertonic saline  Artesunate  Ceftriaxone  0.9% NS or LR | Glucometer  CBC  BMP  Magnesium level  Malaria RDT |
| 4 – Organophosphate Poisoning | Adult simulation mannequin  Stretcher  Cardiac Monitor  Glycerin spray for diaphoresis | Nasal cannula  Nonrebreather mask  Oxygen | Atropine  0.9% NS or LR  Ceftriaxone  Metronidazole  Naloxone | Glucometer  Chest x-ray  EKG  CBC  BMP  UA  Malaria RDT |
| 5 – Postpartum Hemorrhage | Pregnant OB Simulator (Elevate Healthcare)  Stretcher  Cardiac monitor | Nasal cannula  Nonrebreather  Oxygen  Gauze  Forceps  Foley catheter  Condom  Towels | Oxytocin  Misoprostol  Tranexamic acid  Ceftriaxone  Metronidazole  0.9% NS or LR  PRBCs | Glucometer  CBC  Type and screen  UA |

^*^Items listed are available during the case if requested by participants.
