# Supplementary material for: Advancing Global Health Education: Preparing Emergency Medicine Trainees for Low-Resource Settings Through Simulation-Based Training
Source: MedEdPORTAL. 2026 Mar 10;22:11582. doi: 10.15766/mep_2374-8265.11582 (PMC12972016; doi:10.15766/mep_2374-8265.11582)
Supplement: Supplementary file 1 — Equipment for Implementation.docxTraumatic Hemopneumothorax Case.docxTuberculous Pericarditis Case.docxCerebral Malaria Case.docxOrganophosphate Poisoning Case.docxPostpartum Hemorrhage Case.docxLecture.pptxCourse Evaluation.docx [file mep_2374-8265.11582-s001.zip › B. Traumatic Hemopneumothorax Case.docx]

Appendix B. Traumatic Hemopneumothorax Case

Purpose: This appendix contains the complete facilitator-facing simulation case for a trauma scenario focused on recognition and management of hemopneumothorax in a limited-resource setting.

When and How to Use: Facilitators should review this appendix prior to the session and use it as a step-by-step guide during simulation delivery and debriefing. Instructor notes outline expected learner actions, case progression, and prompts. Diagnostic studies, labs, and imaging should be released only when indicated. Debriefing materials support a structured post-simulation discussion.

| **SIMULATION CASE TITLE:**  **Traumatic Hemopneumothorax Management in a Limited Resource Setting**  **AUTHORS: Julianne Jett, MD, Halley J. Alberts, MD, Heather A. Brown, MD, Christopher Gainey, MD, Joshua Skaggs, MD.**  **LEARNER AUDIENCE: Emergency Medicine Residents and Medical Students** | |
| --- | --- |
| **PATIENT NAME: Unknown**  **PATIENT AGE: 34 years**  **CHIEF COMPLAINT: Leg Pain**  **PHYSICAL SETTING: Tertiary Care Center in Nicaragua with access to ultrasound, portable x-ray machine, laboratory services, EKG machines and surgical specialties** | |
|  | |
| **Brief Narrative Description of Case** | A 34-year-old man is brought to the emergency department by coworkers after a construction vehicle accidentally backed into the patient, knocking him to the ground and running over him. The patient is awake and alert on arrival, frantically complaining of leg pain. He has an obvious open fracture of the lower extremity. After initial stabilization, the patient suffers from acute decompensation and is ultimately found to have an expanding hemopneumothorax ultimately requiring tube thoracostomy. |
| **Primary Learning Objectives** | By the end of this activity, residents will be able to:   - Perform primary and secondary surveys to a trauma patient - Use ultrasound in the trauma setting - Consider splinting techniques for an open fracture - Choose appropriate antibiotics and tetanus prophylaxis for an open fracture - Identify and manage life-threatening pneumothorax |
| **Critical Actions** | - Perform primary and secondary survey according to ATLS protocol - Splint patient and administer antibiotics - Address patient’s pain - Repeat primary survey when patient decompensates - Appropriately manage tension pneumothorax |
| **Learner Preparation or Prework** | Active participation in continued medical knowledge and skills via status as Medical Student Year 3 or 4 or Emergency Medicine Resident Year 1-4 |

| **Initial Presentation** | | | |
| --- | --- | --- | --- |
| **Initial Vital Signs** | BP 132/79, HR 112, RR 26, SpO2 97%, T 37.0 | | |
| **Overall Setting, equipment required and Appearance** | Emergency department room with cardiac monitor and ultrasound machine  Mannequin: Adult male, obvious deformity to lower extremity, road rash over left side of chest wall.  Equipment: bottles and tubing for DIY pleur-evac, ultrasound probe, cardboard splint for lower extremity, ceftriaxone, ETT x2, intubation equipment, suction tubing, c-collar | | |
| **Standardized Participants (and Their Roles in the Room at Case Start)** | **Patient** (simulator mannequin voiced by instructor, provides history)  **Nurse** (voiced by instructor, gives prompts when needed as specified below in instructor’s notes) | | |
| **HPI** | **Volunteered by patient:**  “I was stooped over at a construction site when my co-workers accidentally backed over me. My leg is killing me! Can’t I get anything for my leg?”  If asked how he landed, the patient can volunteer that he landed on his back and then the tire ran over his leg. If asked about the bruising on his chest, he can report that some cinder blocks fell off the truck when the driver hit the brakes and landed on the left side of his chest. On review of systems, if asked, he can note some mild chest and back pain but no shortness of breath. He did not hit head or lose consciousness. There is no pain to arms, neck, no headache, no vision changes. He should interrupt questioning to complain about his leg. | | |
| **Past Medical/Surgical History/Social History** | **Medications** | **Allergies** | **Family History** |
| GERD  Smokes ½ ppd, if asked | Over the counter antacid | None | Diabetes |
| **Physical Examination** | | | |
| **General** | Well-developed, well-nourished man in his 30s who is awake, alert, in moderate distress secondary to his leg pain, requesting pain medication. | | |
| **HEENT** | PERRL. No scleral icterus. Oropharynx clear. Scalp is atraumatic. No hemotympanum or evidence of basilar skull fracture. | | |
| **Neck** | Trachea is midline. No cervical spine tenderness on log roll. No bruit or swelling. | | |
| **Lungs** | Mildly tachypneic but clear bilaterally with the exception of mild crackles in the left lung base. | | |
| **Cardiovascular** | Tachycardic. Regular rhythm. Heart sounds distant. No JVD. No peripheral edema. | | |
| **Abdomen** | No tenderness to palpation. Non distended. | | |
| **Neurological** | Oriented to person, place and time. Normal strength and sensation of bilateral upper and lower extremities with the exception of left lower extremity secondary to pain. The patient is able to wiggle toes on that side however. Sensation is intact in the left foot. Gluteal squeeze intact on log roll. | | |
| **MSK** | No bony midline tenderness or step offs in thoracic or lumbar spine. Obvious deformity to lower left lower extremity with concerns for open fracture. Neurovascularly intact distally. | | |
| **Skin** | Road rash over the left lateral chest wall. No other overlying skin changes, lacerations or bruising. | | |
| **GU** | No blood at the urethral meatus. | | |
| **Psychiatric** | Behaving appropriately. | | |

Instructor’s Notes:

| **Time Point/Intervention** | **Potential Actions/Change in Case** | **Additional Information/Prompting** |
| --- | --- | --- |
| T0-T1 | Participants are busy working in the emergency department when a nurse helps carry in a man from the back of a pick-up truck and lays the patient on the exam table. The man is shouting and appears to be in pain.  Participants should begin by asking for a brief history. Bystanders provide and then vacate the room.  Patient complaining of pain in his left leg. | If learners fail to request vital signs the nurse should say, “I will go look for some leads for the monitor.”  If they do not request a basic history the bystanders will ask, “Can we leave?” |
| T1-T2 | Participants should quickly assign roles for management and begin the primary survey.  The nurse places the patient on the monitor and obtains a set of vital signs. Temperature is not provided unless asked.  When asked ROS and history questions, the patient will answer but mostly complain about his leg. | Participants may request a c-collar to which the patient starts shouting, “It isn’t my neck that hurts, it’s my leg!”  If GCS is not assessed, the nurse should ask, “What’s his GCS?” |
| T2-T4 | Participants should begin FAST exam and secondary survey, including ‘AMPLE’ history.  Participants can request labs (CBC, BMP) and chest x-ray, but these are not yet available. | If ultrasound is not requested, the nurse should ask if they want to use it.  The instructor may show participants the negative RUQ image (provided below) and verbalize that the other views are also negative. |
| T4-T6 | Once deemed stable, learners should address the patient’s open fracture. | If given fluids and pain medications,vitals are improved:  HR 93, BP 109/71, 98% on RA, RR 16  Nurse: “Do you think we should rinse that off?”  “Can’t we give him something for pain?”  The nurse can prompt participants to splint with, “Here’s some cardboard.”  If IV access is not already requested, the nurse can say “IM antibiotics, right?” |
| T6-T8 | Patient should stop responding to questions  New vital signs should appear on the screen  HR 129, BP 87/64, 84% on RA, RR 18  Primary survey should be repeated.  When speaking to the patient, he mumbles but his airway is intact. This time, the patient will have diminished breath sounds on the left. Participants should repeat E-FAST. | If participants do not notice a change in patient status, the nurse should prompt.  If learners request chest XR or do not repeat the e-FAST, XR image may be given but the patient should continue to decompensate.  Labs requested earlier in case may be given to participants.  Repeat E-FAST will show lack of lung sliding on the left, no intraperitoneal fluid but large left-sided pleural effusion (hemopneumothorax). This can be verbalized, or the instructor can provide own images. |
| Thoracostomy/End of Case | The patient’s O2 saturation continues to drop, and he stops speaking.  Intubation and/or needle decompression may be performed, but the patient’s vital signs will not improve unless tube thoracostomy is performed.  If thoracostomy is successfully performed, vital signs improve.  Patient decompensates and codes if no recognition of tension hemopneumothorax | Reveal chest tube task trainer when participants verbalize plan for thoracostomy  If learners do not connect the end of the tubing to something, the nurse can say, “You’re making a mess,” or “How are you going to seal that?” to prompt further innovation attempts. |

**Ideal Scenario Flow**

*A 34-year-old male is carried into a busy emergency department by co-workers after being struck by a truck, which also ran over his leg. If asked, participants will learn that a cinder block also fell on his chest during the impact. Initially, the patient’s primary and secondary survey will only reveal an open fracture of the left lower extremity, which learners should address with splinting, antibiotics, and pain management. The patient will then decompensate due to a developing tension hemopneumothorax which participants should promptly recognize and manage with tube thoracostomy.*

**Debriefing Materials**

Reaction: “How do you think that went?”

Description: Have someone summarize the case.

Analysis: Discussion of key learning points.

**Basic Trauma epidemiology** ^1^

- Trauma causes more deaths globally than HIV, tuberculosis and malaria combined
- 90% of all trauma-related injuries worldwide occur in low- and middle-income countries
- Non-fatal injuries also represent an enormous burden with 1 billion people sustaining an injury requiring care annually
- Thoracic trauma causes 25% of trauma-related deaths (lead by abdominal and head trauma)

**General management of trauma patient ^2^**

- Approach each patient initially with the primary survey
  - Airway
    - Assess for patency; clear obstructions
    - Stabilize cervical spine if trauma is suspected
  - Breathing
    - Assess ventilation and oxygenation (look, listen, feel)
    - Treat tension pneumothorax, open chest wounds, or flail chest
  - Circulation
    - Assess pulses (radial, femoral, dorsalis pedis), capillary refill, and blood pressure
    - Control hemorrhage with direct pressure, tourniquet, or packing
    - Initiate IV access and fluid resuscitation (if needed)
  - Disability (neurological status)
    - Assess level of consciousness, measure GCS
    - Evaluate pupils (size, reactivity)
    - Look for signs of neurological injury or hypoglycemia
  - Exposure and Environment
    - Fully expose the patient to inspect for injuries
    - Prevent hypothermia (warm blankets, warm fluids)
- Perform the secondary survey and E-FAST
  - Head-to-toe exam looking for signs of injury
  - Perform an E-FAST exam
- If a patient deteriorates during exam, restart the primary survey beginning with airway
- Obtain AMPLE history
  - A – Allergies: Any known drug, food, or environmental allergies, especially to medications that may be administered during treatment (e.g., antibiotics, contrast agents)
  - M – Medications: Current medications, including prescribed, over-the-counter drugs, herbal supplements, and any anticoagulants
  - P – Past Medical History: Pre-existing medical conditions (e.g., diabetes, hypertension, cardiac disease), surgical history, prior hospitalizations, or significant injuries
  - L – Last Meal: Time of the patient’s last oral intake, important for planning anesthesia and surgical interventions
  - E – Events/Environment Related to Injury: Circumstances and mechanism of the injury (e.g., blunt, penetrating, fall, explosion), as well as environmental factors like temperature exposure, entrapment, or inhalation injury

**Differential for shortness of breath in setting of trauma**

- Pulmonary contusion
- Rib fracture/splinting from pain
- Pneumothorax
- Hemothorax
- Pericardial effusion/tamponade
- Diaphragm rupture
- Don’t forget about exacerbation of chronic conditions such as asthma playing a potential role

**Hemopneumothorax Diagnosis ^3^**

- Clinical Signs
  - Decreased or absent breath sounds on the affected side
  - Dullness to percussion over the chest wall if hemothorax, hyperresonance in pneumothorax
  - Hypotension or signs of shock (due to blood loss or tension effect)
  - Respiratory distress or tachypnea
- Bedside Diagnostics:
- Chest X-ray (CXR)
  - For hemothorax
    - Blunting of the costophrenic angle
    - Opaque appearance of the hemithorax if large
  - For pneumothorax
    - Visible pleural line with absent lung markings beyond it
- (E-FAST)
  - For hemothorax
    - Echogenic fluid in the pleural space
  - For pneumothorax
    - Absence of lung sliding or "barcode sign" (M-mode)

**Hemopneumothorax Management** ^4^

- Needle decompression
  - 12- to 16-gauge angiocatheter, 3 to 4.5 inches in length
  - 5 or 10mL syringe “Why is the syringe helpful?” (Air aspiration)
  - 2nd intercostal space in the mid clavicular line or 5th intercostal space of the anterior axillary line
  - Always place the needle or tube above the rib to avoid injuring the intercostal neurovascular bundle
- Tube thoracostomy
  - Tube sizing - A lot of debate on this
    - ATLS recommends 34-40 F, some studies showing no difference between tubes 28-32F and 36-40F
  - Same location as needle decompression
  - Review steps on mannequin
  - Options if you don’t have a chest tube in a resource limited setting include ETT, central line catheters, foley catheters
  - Drainage systems in resource limited settings have consisted of hard-sided IV fluid bottles, water bottles or jugs, plastic bags with water-seal, IVF bags
    - Can set up one, two, or three ‘bottle’ systems to control suction ^5^

**Open fracture management ^6^**

- Control hemorrhage if present, assess neurovascular function of limb
- Tetanus Prophylaxis
  - Administer based on vaccination history:
    - <3 doses or unknown: Tetanus toxoid + tetanus immunoglobulin
    - ≥3 doses: Only tetanus toxoid if >5 years since last dose
- Antibiotic Therapy
  - Start broad-spectrum antibiotics as soon as possible
    - Grade I (clean, <1 cm): 1st-generation cephalosporin
    - Grade II/III (contaminated, >1 cm): Add aminoglycoside or piperacillin / tazobactam
    - Farm injuries or soil contamination: Add penicillin for Clostridium coverage
- Wound Care
  - Remove gross contamination (dirt, debris) gently with irrigation
  - Perform copious irrigation using sterile saline or water
  - Avoid aggressive debridement until in the operating room
- Imaging
  - Obtain X-rays of the affected area (AP and lateral views)
  - Look for foreign bodies and fracture patterns
- Immobilization
  - Splint the fracture in the position of function
  - Avoid compromising circulation or soft tissue

References

​1. Whitaker J, O'Donohoe N, Denning M, et al. Assessing trauma care systems in low-income and middle-income countries: A systematic review and evidence synthesis mapping the three delays framework to injury health system assessments. *BMJ Glob Health*. 2021;6(5):e004324. [https://pubmed.ncbi.nlm.nih.gov/33975885/. Accessed Jan 25, 2025. doi: 10.1136/bmjgh-2020-004324.](about:blank)

​2. Kool DR, Blickman JG. Advanced trauma life support®. ABCDE from a radiological point of view. *Emerg Radiol*. 2007;14(3):135–141. [https://www.ncbi.nlm.nih.gov/pmc/articles/PMC1914302/. Accessed Jan 24, 2025. doi: 10.1007/s10140-007-0633-x.](about:blank)

​3. Sharma A, Jindal P. Principles of diagnosis and management of traumatic pneumothorax. *J Emerg Trauma Shock*. 2008;1(1):34–41. [https://www.ncbi.nlm.nih.gov/pmc/articles/PMC2700561/. Accessed Jan 24, 2025. doi: 10.4103/0974-2700.41789.](about:blank)

​4. Liman ST, Kuzucu A, Tastepe AI, Ulasan GN, Topcu S. Chest injury due to blunt trauma. *Eur J Cardiothorac Surg*. 2003;23(3):374–378. [https://pubmed.ncbi.nlm.nih.gov/12614809/. Accessed Jan 24, 2025. doi: 10.1016/s1010-7940(02)00813-8.](about:blank)

​5. Zisis C, Tsirgogianni K, Lazaridis G, et al. Chest drainage systems in use. *Ann Transl Med*. 2015;3(3):43. [https://www.ncbi.nlm.nih.gov/pmc/articles/PMC4356865/. Accessed Jan 24, 2025. doi: 10.3978/j.issn.2305-5839.2015.02.09.](about:blank)

​6. Sop JL, Sop A. Open fracture management. In: *StatPearls.* Treasure Island (FL): StatPearls Publishing; 2025. [http://www.ncbi.nlm.nih.gov/books/NBK448083/. Accessed Jan 24, 2025.](about:blank)

**Labs**

This should be given to the participants when indicated in the instructor notes.

BMP:

Na 139 mEq/L

K 4.1 mEq/L

Cl 104 mEq/L

CO2 22 mEq/L

Glucose 102 mg/dL

BUN 12 mg/dL

Cr 0.7 mg/dL

Ca 8.9 mg/dL

CBC:

WBC 6.3 x 10^9^/L

Hgb 13.5 g/dL

Plt 224 K/mm^3^

E-FAST RUQ View

This should be given to the participants when indicated in the instructor notes.


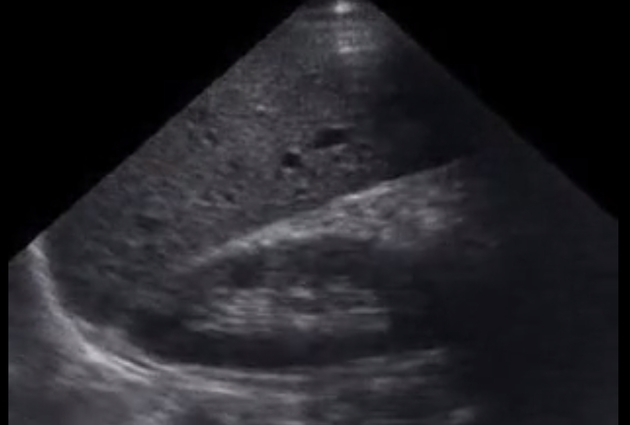


Image by David Carroll, retrieved from: https://radiopaedia.org/cases/64279 on February 1, 2023. Creative Commons License associated: https://creativecommons.org/licenses/by-nc-sa/3.0/legalcode.

Chest X-ray

This should be given to the participants when indicated in the instructor notes.


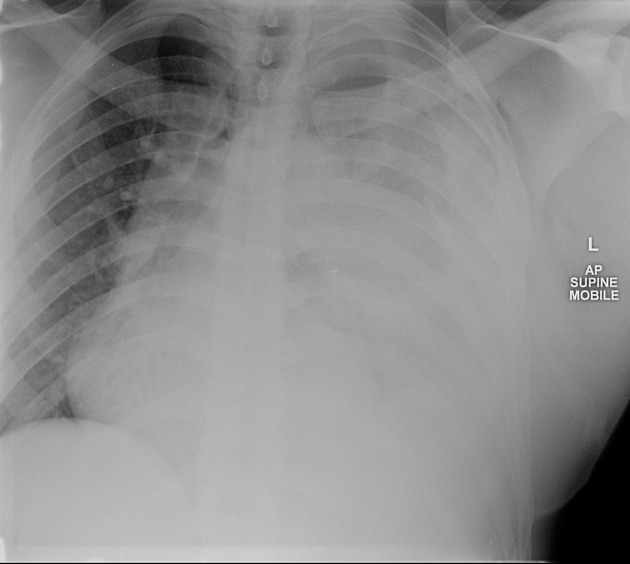


Image by Henry Knipe, retrieved from: https://radiopaedia.org/cases/47300 on February 1, 2023. Creative Commons License associated: https://creativecommons.org/licenses/by-nc-sa/3.0/legalcode.


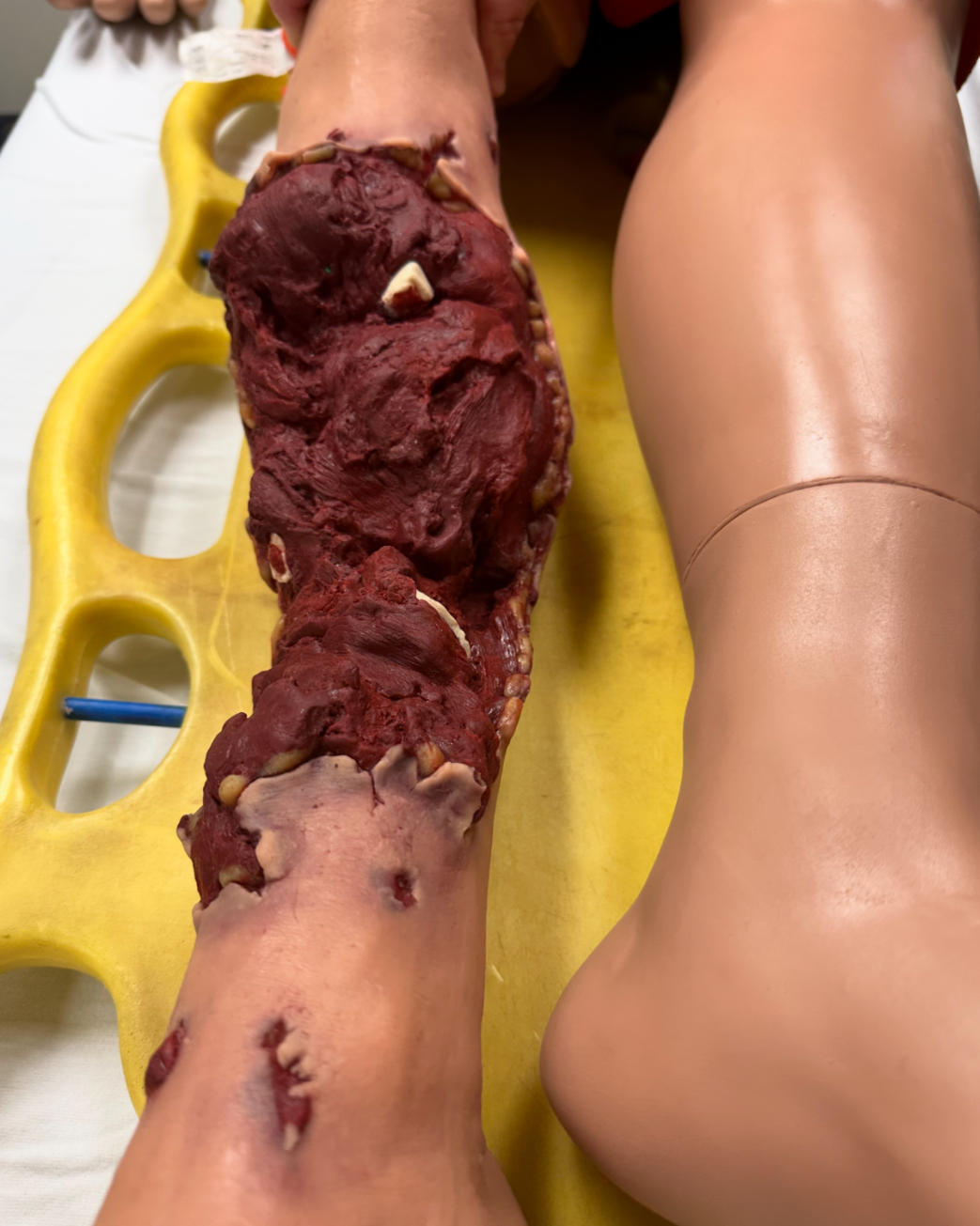


Author owned. Moulage used to simulate lower extremity trauma in the simulation lab.


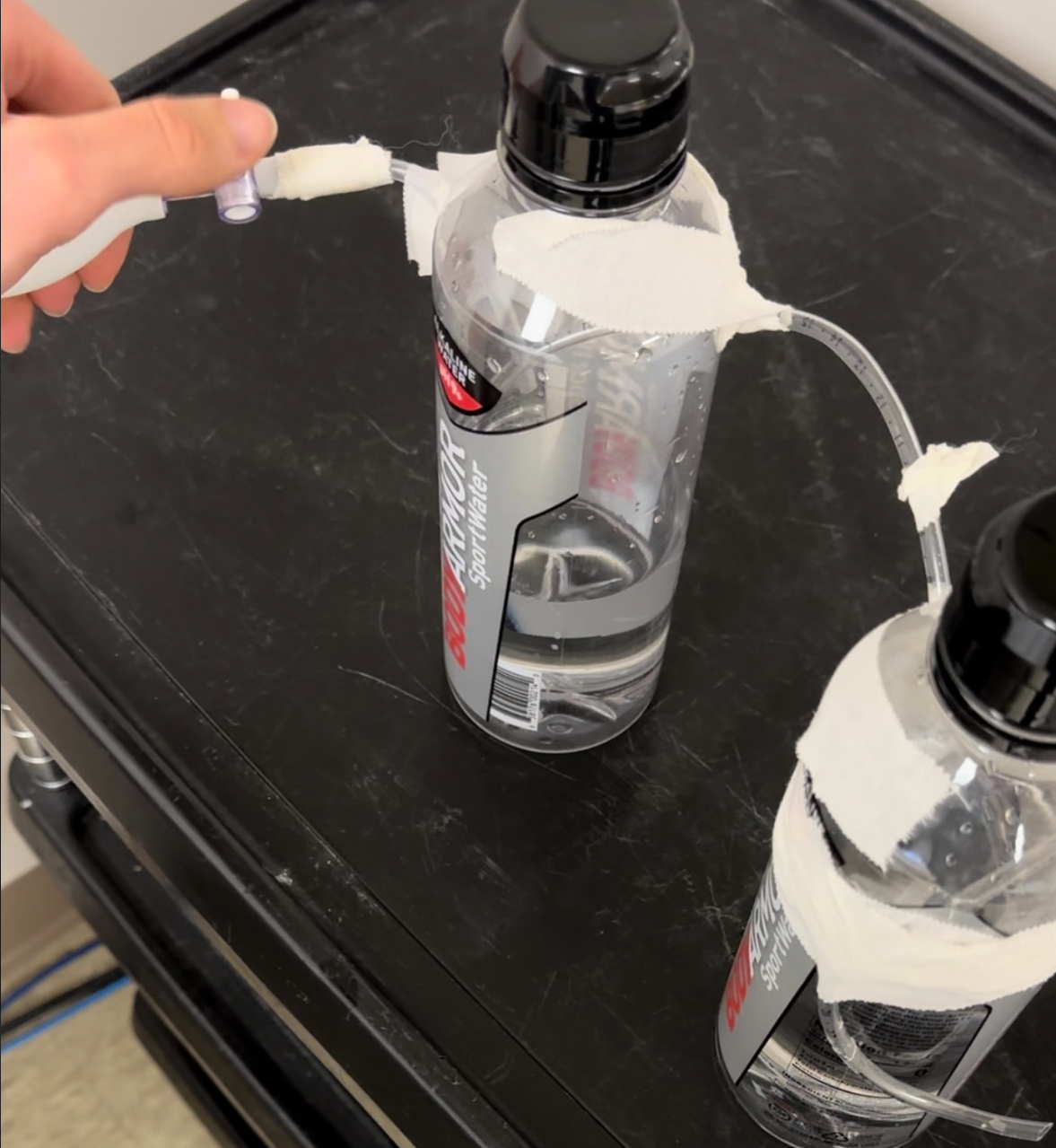


Author owned. Example of a two-bottle lung evacuation system that was made during the simulation day. Here, the inflatable bulb from a blood pressure cuff acts as a lung, with respirations achieved by manually squeezing the bulb.
