# Supplementary material for: Advancing Global Health Education: Preparing Emergency Medicine Trainees for Low-Resource Settings Through Simulation-Based Training
Source: MedEdPORTAL. 2026 Mar 10;22:11582. doi: 10.15766/mep_2374-8265.11582 (PMC12972016; doi:10.15766/mep_2374-8265.11582)
Supplement: Supplementary file 1 — Equipment for Implementation.docxTraumatic Hemopneumothorax Case.docxTuberculous Pericarditis Case.docxCerebral Malaria Case.docxOrganophosphate Poisoning Case.docxPostpartum Hemorrhage Case.docxLecture.pptxCourse Evaluation.docx [file mep_2374-8265.11582-s001.zip › C. Tuberculous Pericarditis Case.docx]

Appendix C. Tuberculosis Pericarditis

Purpose: This appendix contains the complete facilitator-facing simulation case focused on the recognition and management of tuberculous pericarditis with cardiac tamponade.

When and How to Use: Facilitators should review this appendix prior to the session and use it as a step-by-step guide during simulation delivery and debriefing. Instructor notes outline expected learner actions, case progression, and prompts. Diagnostic studies, labs, and imaging should be released only when indicated. Debriefing materials support a structured post-simulation discussion focused on tuberculosis-related cardiac pathology and management considerations in TB-endemic settings.

| **SIMULATION CASE TITLE: Tuberculous Pericarditis in a Patient with HIV**  **AUTHORS: Julianne Jett, MD, Halley J. Alberts, MD, Heather A. Brown, MD, Christopher Gainey, MD, Joshua Skaggs, MD**  **LEARNER AUDIENCE: Emergency Medicine Residents and Medical Students** | |
| --- | --- |
| **PATIENT NAME: Cynthia**  **PATIENT AGE: 30 years**  **CHIEF COMPLAINT: Shortness of breath and chest pain**  **PHYSICAL SETTING: Tertiary Care Center in Tanzania with access to ultrasound, portable x-ray machine, laboratory services, EKG machines.** | |
|  | |
| **Brief Narrative Description of Case** | A 30-year-old HIV+ female in Tanzania presents to the emergency department with shortness of breath and chest pain. Initial vital signs and exam are concerning for cardiac tamponade, and this is confirmed with bedside echocardiogram. Pericardiocentesis results in significant hemodynamic improvement, and further workup reveals evidence of pulmonary TB on chest x-ray. |
| **Primary Learning Objectives** | By the end of this activity, residents will be able to:   - Recognize cardiac tamponade with point-of-care ultrasound - Determine when pericardiocentesis vs. conservative management is indicated for pericardial effusion - Detect findings of pulmonary tuberculosis on chest radiograph - Recognize common manifestations of extrapulmonary tuberculosis - Utilize FASH exam to expedite TB treatment - Appreciate the high prevalence of HIV and TB coinfection |
| **Critical Actions** | - Identify pericardial effusion and tamponade with bedside echocardiogram - Perform pericardiocentesis - Obtain chest x-ray and recognize pulmonary tuberculosis - Initiate RIPE therapy despite not having pericardial fluid results |
| **Learner Preparation or Prework** | Active participation in continued medical knowledge and skills via status as Medical Student Year 3 or 4 or Emergency Medicine Resident Year 1-4 |

| **Initial Presentation** | | | |
| --- | --- | --- | --- |
| **Initial Vital Signs** | BP 85/70, HR 131, RR 28, SpO2 92%, T 38.2 | | |
| **Overall Setting and Appearance** | **Setting:** Emergency department room with cardiac monitor, oxygen, code cart and ultrasound machine.  **Mannequin:** Adult sim mannequin with respiratory rate 28, eyes open, and instructor playing the voice of the patient. Glycerin spray for diaphoresis. A task trainer can be brought into the room when participants express the need for pericardiocentesis, or present in the room from the beginning of the case.  **Additional equipment:** Nasal cannula, ultrasound machine, glucometer, spinal needle with stopcock and syringe or pericardiocentesis set, ceftriaxone, antimycobacterial agents, prednisolone, furosemide, code cart with standard ACLS medications. No intubation equipment. | | |
| **Standardized Participants (and Their Roles in the Room at Case Start)** | **Patient:** Simulator mannequin voiced by instructor, provides history  **Nurse**: Voiced by instructor, gives prompts when needed as specified below in instructor’s notes | | |
| **HPI** | **Volunteered by patient:**  Progressive chest pain and shortness of breath for the past 2 weeks. The pain is midsternal and is worse with deep breathing and lying down.  **Must be asked:**  ROS positive for non-productive cough, generalized abdominal pain, intermittent fever (usually in evening), night sweats, generalized myalgias, weight loss (a few kilograms).  Denies unilateral leg pain or swelling. | | |
| **Past Medical/Surgical History**  HIV (participants must ask)  The patient is unsure if she has ever been treated for TB. | **Medications**  None  If asked, denies currently taking antiretroviral therapy. | **Allergies**  None | **Family History**  Hypertension |
| **Physical Examination** | | | |
| **General** | Cachectic, ill-appearing, diaphoretic. | | |
| **HEENT** | PERRLA. No scleral icterus. Oropharynx clear. | | |
| **Neck** | No lymphadenopathy. | | |
| **Lungs** | Tachypneic. Crackles auscultated at the bilateral lung bases. | | |
| **Cardiovascular** | Tachycardic. Regular rhythm. Heart sounds distant. JVD present. Bilateral lower extremity edema. | | |
| **Abdomen** | RUQ abdominal tenderness to palpation. Hepatomegaly present. | | |
| **Neurological** | Oriented to person, place and time. Full strength and sensation of bilateral upper and lower extremities. | | |
| **Skin** | No rash. No bruises. | | |
| **GU** | NA | | |
| **Psychiatric** | NA | | |

**Instructor Notes**

| **Time Point/Intervention** | **Potential Actions/Change in Case** | **Additional Information/Prompting** |
| --- | --- | --- |
| T0 | Appearance from doorway: Cachecticfemale patient struggling to answer questions due to dyspnea.  Vitals: BP 85/70, HR 131, RR 28, SpO2 92%, T 38.2  Physical Exam: ll-appearing, diaphoretic. Tachypneic, crackles at the lung bases. Tachycardic, regular rhythm, heart sounds distant. JVD. RUQ abdominal tenderness, hepatomegaly. Bilateral lower extremity edema. |  |
| T 2-3 min | The patient should be placed on supplemental O2—only requires nasal cannula, SpO2 improves to 95%.  EKG (if requested) shows electrical alternans.  Bedside echocardiogram shows large pericardial effusion with impaired RV filling and pericardial stranding (if they request other views: lungs show B lines, no pleural effusion, LUQ/RUQ/pelvis show trace ascites, normal liver and spleen, no significantly enlarged abdominal lymph nodes) | If participants fail to perform POCUS, RN comments, “Should we use our new ultrasound machine?”  If participants administer fluids, the patient’s blood pressure does not improve.  If participants fail to recognize cardiac tamponade and need for pericardiocentesis, the patient’s blood pressure drops to 75/60. If participants fail to perform pericardiocentesis after hypotension worsens, the patient suffers a PEA arrest. |
| Pericardiocentesis | Repeat vitals after pericardiocentesis: BP 110/65, HR 105  If bedside echo is repeated, it shows significantly smaller effusion, no RV diastolic collapse, and grossly reduced EF (this can be verbalized) | If needed, RN can prompt participants to order additional labs or imaging after stabilization. |
| **Further workup after stabilization:** Participants should ask for CBC, BMP, BNP, troponin, CXR, pericardial fluid tests (cell count, protein, gram stain and culture acid fast smear and cultures). | Labs given to participants.  CXR shows mild edema and RUL infiltrate with cavitation and hilar lymphadenopathy.  Pericardial fluid results not yet resulted. |  |
| **End of case:** Participants start empiric anti-tuberculous therapy prior to fluid results, steroids for tuberculous pericarditis, and furosemide for pulmonary edema if not given previously. The patient is admitted. |  |  |

**Ideal Scenario Flow**

*A 30 year old Tanzanian female patient presents to the emergency department for chest pain and shortness of breath. She endorses a history of HIV if asked. She is ill-appearing, hypotensive, tachycardic, and hypoxic. She has distant heart sounds and JVD is present on exam. Bedside echocardiogram will reveal a large pericardial effusion causing RV diastolic collapse. Participants should perform pericardiocentesis, which results in improved blood pressure. Once the patient has been stabilized, the participants should investigate further with labs and imaging. Chest x-ray will show evidence of pulmonary tuberculosis. Pericardial fluid studies will not result during the case, but learners should request cell count, protein level, Gram stain and culture, and acid-fast smear. Participants should start treatment for tuberculosis prior to these results based on the chest x-ray findings, the patient’s history of HIV, and the location in a TB-endemic region.*

**Debriefing Materials**

Reaction: “How do you think that went?”

Description: Have someone summarize the case.

Analysis: Discussion of key learning points.

**Causes of pericarditis/pericardial effusion**

- Autoimmune (SLE, RA, scleroderma)
- Malignancy
- Hypothyroid
- Trauma with hemopericardium
- Bacterial infection (*Staphylococcus, Streptococcus pneumoniae*, beta-hemolytic strep, TB)
- Fungal infection (*Histoplasma*)
- Uremia
- Postmyocardial infarction (Dressler’s syndrome)

**Tuberculous Pericarditis**

- Most common cause of pericardial effusion in southern Africa^1^
- Higher incidence with HIV coinfection^2^
- Symptoms overlap with PTB
- Cardiac tamponade in 10% of cases with tuberculous pericardial effusion^1^
- Reabsorption may occur in approximately half the cases^1^ (Be cautious about pericardiocentesis in hemodynamically stable patient in this setting)
- Treatment: same antituberculous therapy as PTB, plus steroids

**Tuberculosis**

- Caused by acid-fast bacillus *Mycobacterium tuberculosis*
- Transmitted by inhalation of aerosol droplets
- TB infection (latent, asymptomatic) vs. TB disease (symptomatic and contagious)
- **Pulmonary TB**
  - Fever, cough, malaise, night sweats, weight loss, chest pain, dyspnea
  - Primary disease CXR: often normal, can also see hilar lymphadenopathy, parenchymal infiltrates
  - Latent infection CXR: upper lobe or hilar nodules, fibrosis, pleural scarring, Ghon foci
  - Reactivation disease CXR: upper lobe consolidation with cavitation
- **Extrapulmonary TB**
  - 20% of all TB cases in Africa^2^.
  - Can affect cardiac, nervous, renal, musculoskeletal, GI, ophthalmologic, dermatologic, and endocrine systems

**FASH Exam: Focused Assessment with Sonography for HIV-associated Tuberculosis^2^**

- Way to evaluate for common manifestations of EPTB
- Use when there is high suspicion for extrapulmonary TB in patients with known or suspected HIV in settings with high HIV prevalence (>5%). If positive, start TB treatment.

6 Questions:

1. Is there a pericardial effusion?
2. Is there a pleural effusion?
3. Is there free fluid in the abdomen or pelvis?
4. Are there focal liver lesions?
5. Are there focal splenic lesions?
6. Are there periportal/para-aortic lymph nodes?

**HIV/TB coinfection**

- If considering a TB diagnosis, also test for HIV.
- Individuals with HIV are 20-30 times more likely to develop tuberculosis.^3^
- Individuals with new TB are 20 times more likely to be HIV positive.^3^

**References**

1. Stout J. Tuberculous pericarditis. In: Post TW, UpToDate. Waltham, MA: UpToDate; 2023.
2. Heller T, Wallrauch C, Goblirsch S, Brunetti E. Focused assessment with sonography for HIV-associated tuberculosis (FASH): a short protocol and a pictorial review. Crit Ultrasound J. 2012;4(1):21. doi:10.1186/2036-7902-4-21.
3. Sonnenberg P, Glynn JR, Fielding K, Murray J, Godfrey-Faussett P, Shearer S. How soon after infection with HIV does the risk of tuberculosis start to increase? A retrospective cohort study in South African gold miners. J Infect Dis. 2005;191:150. doi:15609223.

**Labs**

These should be given to the participants when indicated in the instructor notes.

BMP:

Na 138 mEq/L

K 4.0 mEq/L

Cl 104 mEq/L

CO2 19 mEq/L

Glucose 102 mg/dL

BUN 12 mg/dL

Cr 1.2 mg/dL

Ca 8.9 mg/dL

CBC:

WBC 6.3 x 10^9^/L

Hgb 10.3 g/dL

Plt 175 K/mm^3^

Troponin level 0.23 ng/mL (normal <0.03 ng/mL)

EKG

This should be given to the participants when indicated in the instructor notes.

**
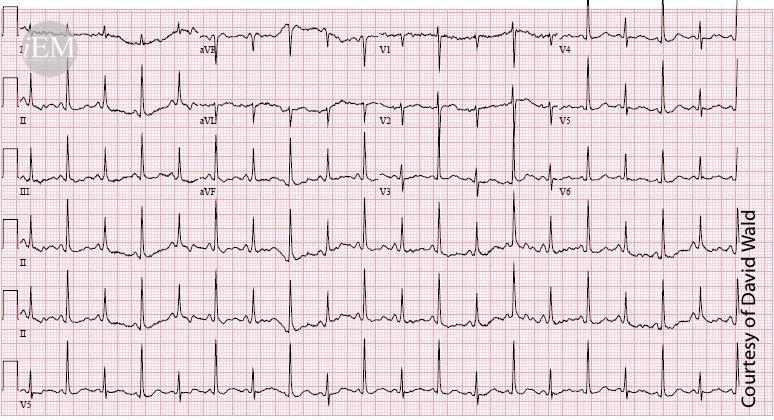
**

Image by David Wald, retrieved from:https://iem-student.org/pericardiocentesis/ on February 2, 2023. Creative Commons License associated: https://creativecommons.org/licenses/by-nc-sa/4.0/

Bedside Echo

This should be given to the participants when indicated in the instructor notes.


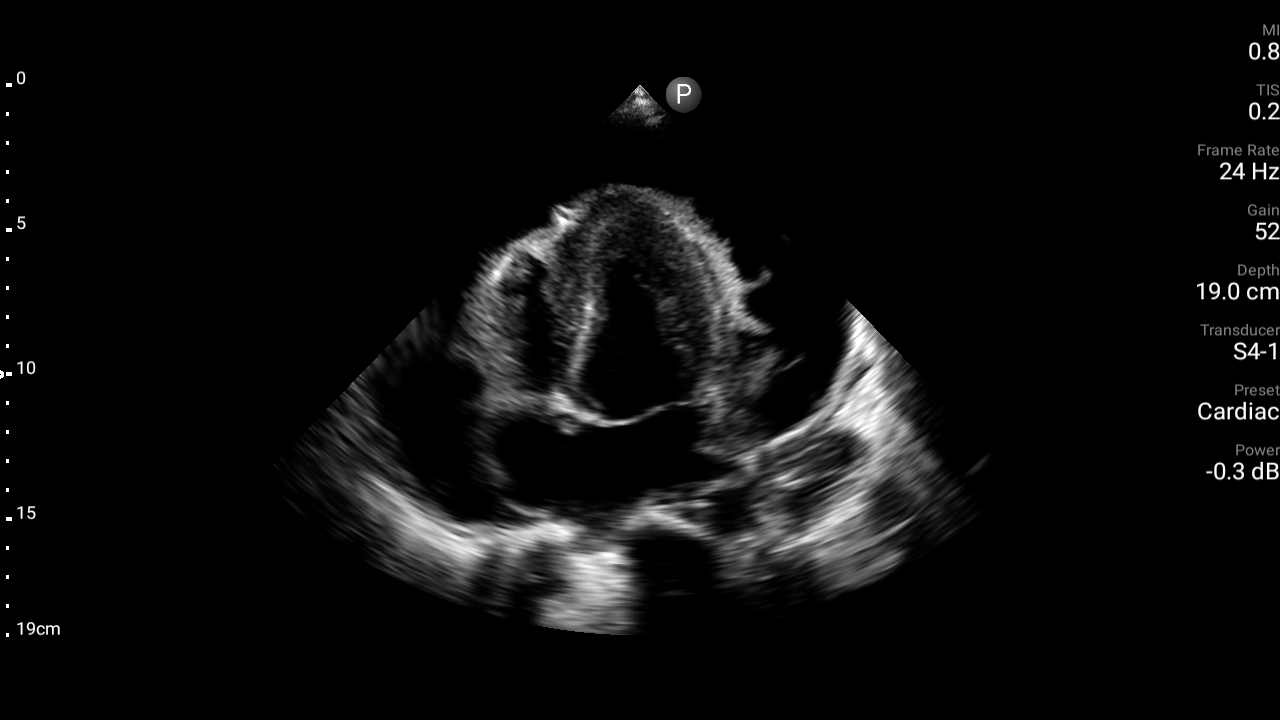


Author owned.

Chest X-ray

This should be given to the participants when indicated in the instructor notes.


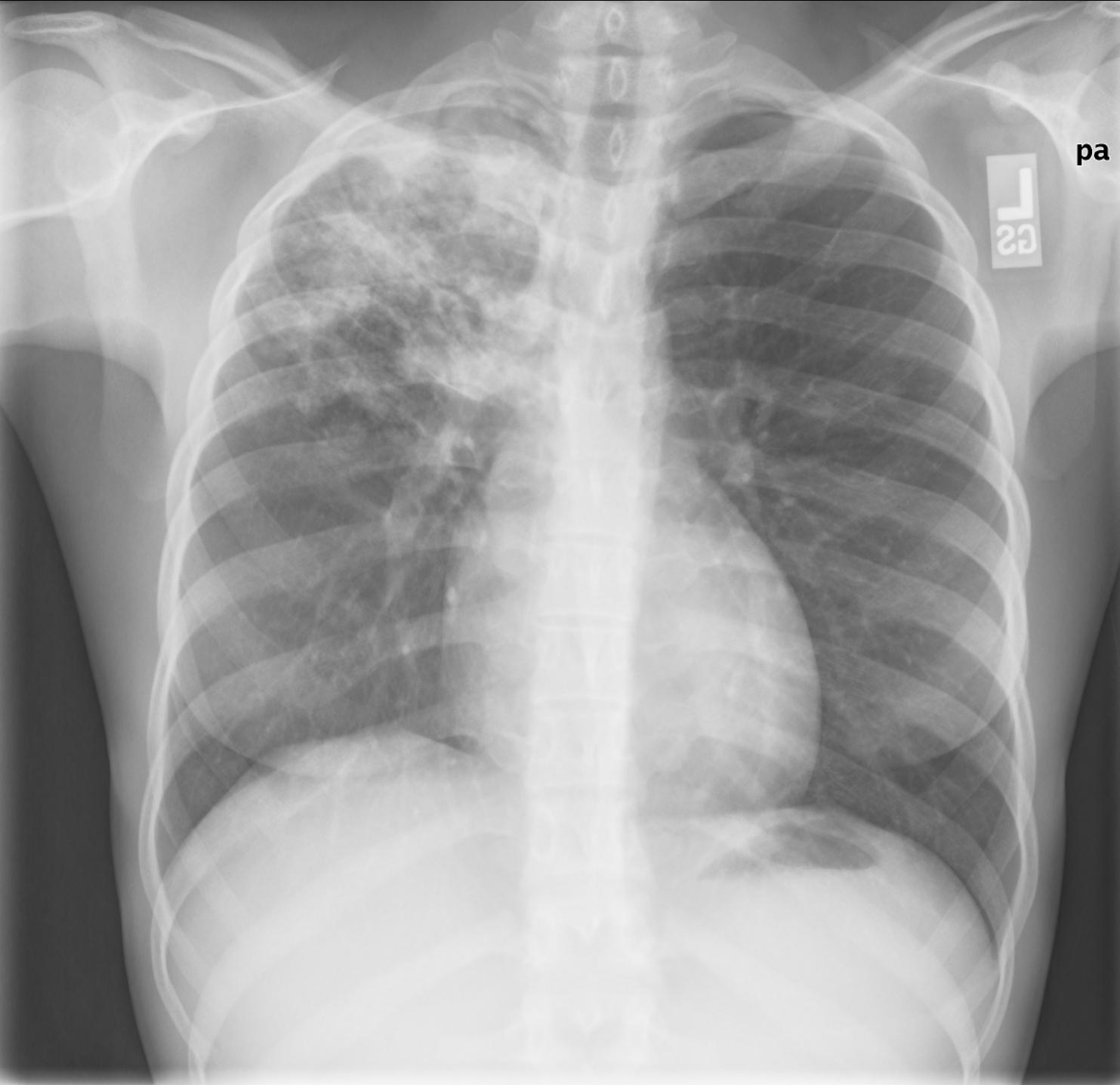


Image by Frank Gaillard, retrieved from: https://radiopaedia.org/cases/8632 on February 3, 2023. Creative Commons License associated:https://creativecommons.org/licenses/by-nc-sa/3.0/legalcode.


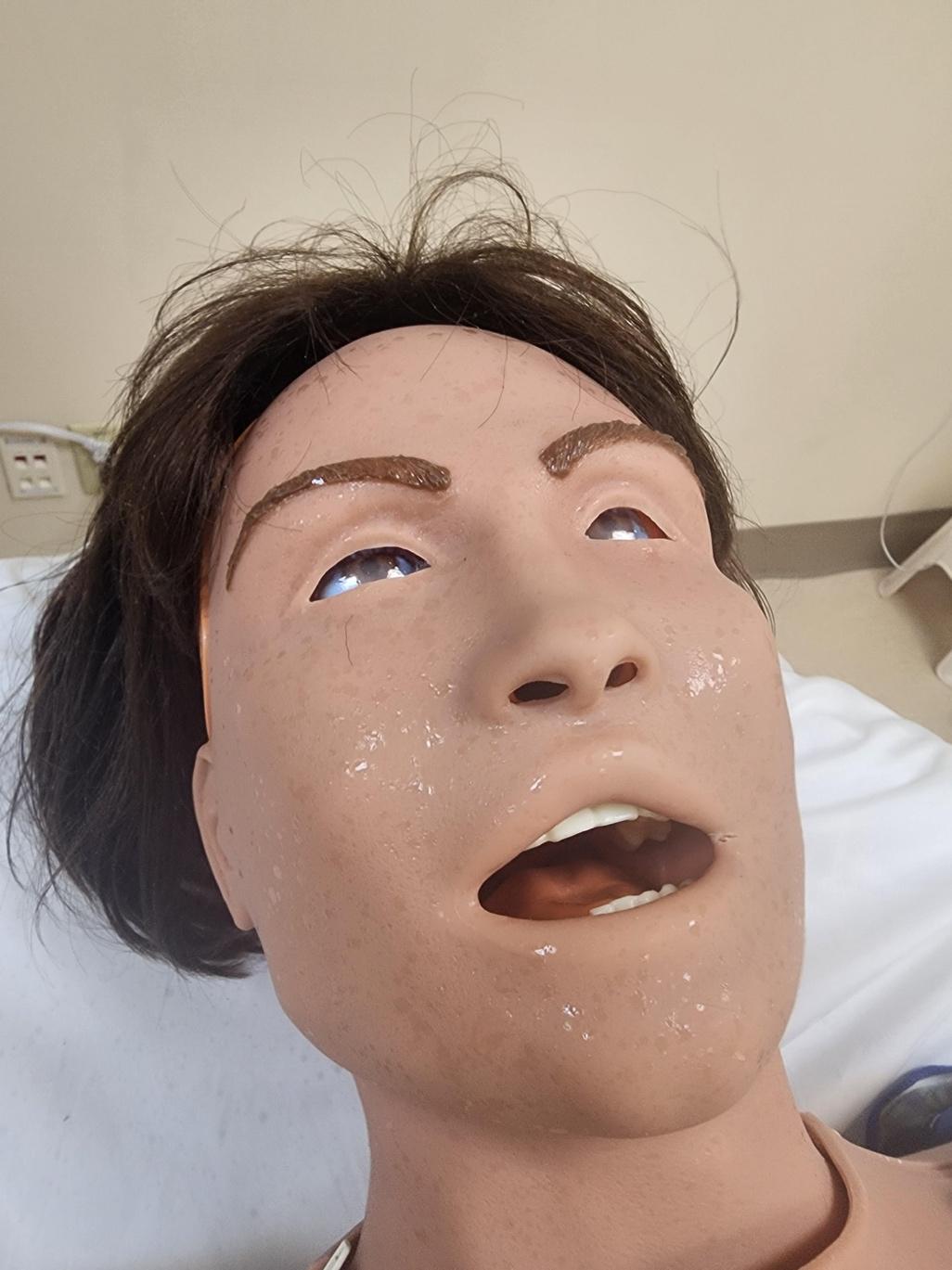


Author owned. Diaphoresis was simulated using glycerin spray.
