# Supplementary material for: Advancing Global Health Education: Preparing Emergency Medicine Trainees for Low-Resource Settings Through Simulation-Based Training
Source: MedEdPORTAL. 2026 Mar 10;22:11582. doi: 10.15766/mep_2374-8265.11582 (PMC12972016; doi:10.15766/mep_2374-8265.11582)
Supplement: Supplementary file 1 — Equipment for Implementation.docxTraumatic Hemopneumothorax Case.docxTuberculous Pericarditis Case.docxCerebral Malaria Case.docxOrganophosphate Poisoning Case.docxPostpartum Hemorrhage Case.docxLecture.pptxCourse Evaluation.docx [file mep_2374-8265.11582-s001.zip › D. Cerebral Malaria Case.docx]

Appendix D. Cerebral Malaria.

Purpose: This appendix contains the complete facilitator-facing simulation case focused on the recognition and management of cerebral malaria in a pediatric patient in a malaria-endemic, resource-limited setting.

When and How to Use: Facilitators should review this appendix prior to the session and use it as a step-by-step guide during simulation delivery and debriefing. Instructor notes outline expected learner actions, case progression, and prompts. Diagnostic studies and laboratory results should be released only when indicated. Debriefing materials support discussion of severe malaria, seizure management, and contextual considerations in endemic settings.

| **SIMULATION CASE TITLE: Cerebral Malaria: A Simulation Case for Emergency Medicine Residents**  **AUTHORS: Julianne Jett, MD, Halley J. Alberts, MD, Heather A. Brown, MD, Christopher Gainey, MD, Joshua Skaggs, MD.**  **LEARNER AUDIENCE: Emergency Medicine Residents and Medical Students** | |
| --- | --- |
| **PATIENT NAME: Kwende**  **PATIENT AGE: 5 years**  **CHIEF COMPLAINT: Lethargy**  **PHYSICAL SETTING: Tertiary care center in Malawi. A laboratory with specialized testing equipment, a pharmacy, oxygen, and basic airway equipment is available.** | |
|  | |
| **Brief Narrative Description of Case** | A 5-year-old male is brought in by his mother for lethargy. On obtaining initial vital signs, he is found to be febrile. The patient develops seizures shortly after arrival and has a prolonged coma despite successful treatment of seizure, fever, and hypoglycemia. Ultimately the patient requires airway management and treatment for malaria. |
| **Primary Learning Objectives** | By the end of this activity, residents will be able to:   - Understand the similarities and differences between workup of seizure with fever in a high-income country that is not malaria endemic vs. in malaria-endemic areas. - Develop a differential diagnosis for likely causes of seizure based on this understanding. - Calculate and apply Glasgow and/or Blantyre Coma Scales to assess the level of consciousness in pediatric patients. - Understand the clinical manifestations, diagnosis, complications, and treatment of malaria. - Recognize the need for airway intervention and employ basic airway management techniques when intubation is not feasible. |
| **Critical Actions** | - Recognize and treat seizures. - Recognize and treat hypoglycemia. - Recognize the inability to protect the airway and apply basic airway management techniques. - Test for malaria and start antimalarial treatment. - Treat with empiric antibiotics for possible concomitant bacterial infection. |
| **Learner Preparation or Prework** | Active participation in continued medical knowledge and skills via status as Medical Student Year 3 or 4 or Emergency Medicine Resident Year 1-4 |

| **Initial Presentation** | | | |
| --- | --- | --- | --- |
| **Initial Vital Signs** | BP 92/50, HR 142, T 40C, SpO2 98%, RR 30 | | |
| **Overall Setting and Appearance** | **Setting:** Emergency department room with stretcher, cardiac monitor, and oxygen concentrator.  **Mannequin:** Child mannequin on stretcher, with closed eyes that open briefly to voice, normal respiratory rate, clear bilateral breath sounds, and palpable pulses.  **Additional equipment:** Glucometer, nasal cannula, simple mask, and medications including oral and rectal acetaminophen, rectal diazepam, D10, NS, 3% saline, artesunate, ceftriaxone. | | |
| **Standardized Participants (and Their Roles in the Room at Case Start)** | **Mother:** voiced by instructor, not played by an actor in the room. Answers history questions.  **Nurse:** voiced by instructor, gives prompts when needed as specified below in instructor’s notes. | | |
| **HPI** | **Volunteered by mother:**  The patient has been less active the past few days and had poor oral intake. He has had progressive lethargy. He felt warm at home but the family does not own a thermometer. He vomited once earlier today.  **Must be asked:**  Lives with mother, grandmother, aunt, siblings, cousins.  Unvaccinated.  No known sick contacts.  ROS negative for cough, congestion, sore throat, rash, diarrhea, abdominal pain. | | |
| **Past Medical/Surgical History** | **Medications** | **Allergies** | **Family History** |
| None. | None. | None. | No pertinent family history. |
| **Physical Examination** | | | |
| **General** | Ill-appearing, obtunded. | | |
| **HEENT** | PERRLA. | | |
| **Neck** | No lymphadenopathy. No nuchal rigidity. | | |
| **Lungs** | Clear to auscultation bilaterally. Normal respiratory rate. | | |
| **Cardiovascular** | Slightly tachycardic. Regular rhythm. No murmur. | | |
| **Abdomen** | Nontender to palpation. Nondistended. | | |
| **Neurological** | Withdraws to pain, briefly opens eyes to pain, moans, otherwise unresponsive. | | |
| **Skin** | No bruises, lacerations, or other external evidence of trauma. | | |
| **GU** | NA | | |
| **Psychiatric** | NA | | |

Instructor Notes – Changes and Case Branch Points

| **Time Point/Intervention** | **Potential Actions/Change in Case** | **Additional Information/Prompting** |
| --- | --- | --- |
| T0 | Appearance from doorway: Ill-appearing, lethargic child.  Vitals: BP 92/50, HR 142, T 40C, SpO2 98%, RR 30  Weight: Motherdoesn’t know, use Broselow tape (20kg)  Physical Exam: Lying supine, eyes closed, will open to pain. Neck is supple, Heart is tachycardic, Lungs are clear, Abdomen is nontender and nondistended. The child will withdraw from pain and occasionally moan. | If participants request IV and/or labs, the nurse tells them they are working on getting an IV. |
| T 2 min: Patient has generalized tonic clonic seizure. Still no IV yet. | Correct Action: give rectal diazepam (0.2mg/kg = 4mg) and rectal acetaminophen (15mg/kg = 300mg).  If rectal diazepam is given, the patient stops seizing. If rectal acetaminophen is given, the fever resolves.  If rectal diazepam is not given, the patient continues to seize. If rectal acetaminophen is not given, the temperature does not improve. | If participants fail to treat seizure, the nurse asks, “What should we do about his seizure?”  If participants fail to treat fever, the nurse asks, “What should we do about his fever?”  If participants ask what medications for seizure are available, the nurse can tell them rectal diazepam is available. |
| Participants give rectal diazepam and acetaminophen. | Patient stops seizing.  Repeat temperature 37.2C. |  |
| Seizure resolves after rectal diazepam, but the patient remains unarousable. IV and labs are still not available. | Correct Action: check fingerstick blood glucose level.  If POC blood glucose is checked, it is 45 mg/dL.  If blood glucose is not checked and repleted, the patient seizes again. | If participants fail to check glucose, the nurse asks, “Do you want me to check his glucose level?” |
| Participants request glucose level. | Glucose level is 45 mg/dL |  |
| Glucose level is 45 mg/dL | Correct Action: treat hypoglycemia.  If discussing PO vs. IV, the patient’s nurse tells participants she has obtained IV access.  If treated with IV D10 (5 ml/kg or 100ml), glucose improves to 115 mg/dL but mentation remains poor (GCS 8 or BCS 2).  If not treated with IV D10, the patient seizes again. | If participants fail to treat hypoglycemia, the nurse asks, “What should we do about his glucose level?” |
| Participants give IV D10 | Repeat glucose level 115 mg/dL |  |
| Repeat glucose is 115 mg/dL, but the patient’s mental status remains poor (GCS 8 or BCS 2). The patient has increased snoring respirations. | Correct Action: recognize the need for airway intervention.  If intubation is requested, the patient's nurse tells participants no ventilator is available and they will need to manage his airway without intubation (raise head of bed, chin tilt, suction available).  If participants request a head CT, it is not available.  If participants request LP, CSF results are not immediately available. | If mental status/intubation is not discussed, the patient’s nurse asks participants, “What do you think his GCS is?” |
| Participants ask for labs (CBC, BMP, thick and thin smears, blood cultures, Malaria RDT) and are given results of the available labs. | Correct actions:  Hgb 7.7g/dL: Consider blood transfusion of 10 ml/kg PRBCs for a total of 200ml (not required to move forward in case).  Plt 89,000 /µL: No intervention for this.  Na 124 mEq/L: Can consider 3% saline of 2 ml/kg up to max of 100ml over 10 minutes for a total of 40ml (not required to move forward in case).  Malaria RDT positive: Treat presumed cerebral malaria with artesunate (2.4 mg/kg or 48mg) .  Treat with ceftriaxone (100 mg/kg or 2g) for possible concomitant bacterial infection to include possible meningitis and sepsis. | If labs are not ordered yet, the nurse asks participants if they want labs. |
| **End of case:** Participants recognize and start treatment for cerebral malaria and admit the patient. | Call the appropriate consultant and admit the patient. |  |

**Ideal Scenario Flow**

*A 5-year-old Malawian male patient is brought in by his mother for lethargy and fever. He begins seizing while participants are taking a history and performing an exam. Learners treat the seizure with rectal diazepam and treat his fever. He has a prolonged comatose state after the seizure resolves. Participants request a point-of-care glucose level which is low. Participants treat the hypoglycemia with IV dextrose, which normalizes the glucose level but does not lead to any improvement in mental status. His airway is managed conservatively. Labs are remarkable for anemia, thrombocytopenia, hyponatremia, acidosis, and positive malaria RDT. Participants consider giving hypertonic saline and blood products but this is not required. The participants do begin treatment with artesunate for cerebral malaria as well as empiric antibiotics for possible concomitant bacterial meningitis.*

**Debriefing materials**

Reaction: “How do you think that went?”

Description: Have someone summarize the case.

Analysis: Discussion of key learning points.

**Causes of Seizures in the Global Health Setting**

- Intracranial Trauma
- Mass
- Vascular lesions
- Electrolyte disturbances (hypoglycemia, hyponatremia, hypocalcemia)
- Toxic ingestion (medications, organophosphates, carbon monoxide, drugs of abuse, heavy metals)
- Fever
- Meningitis / Encephalitis
- Neurocysticercosis (most common cause of acquired epilepsy worldwide)^1^
- Tuberculosis (increased risk for meningitis and for intracranial lesions)
- HIV (increased risk for meningitis and for intracranial lesions)
- Malaria

**Approach to Pediatric Seizures with Fever based on Geography**

High-Income Countries: If a patient meets criteria for a simple febrile seizure and returns to neurologic baseline, while a source for the fever is explored, frequently the patient is discharged to follow up with their pediatrician.

Low and Middle Income Countries: Seizures with fever in tropical regions and low resource settings are more concerning for the infectious causes listed as causes of seizures and may require additional workup.

**Malaria**

- Parasitic disease belonging to the genus Plasmodium and transmitted by the bite of the Anopheles mosquito.
- *Plasmodium falciparum* is the most common species and most likely to cause severe disease.
- Globally in 2022 there were 249 million cases of malaria, with 608,000 deaths occurring as a result.^2^ The greatest burden of malaria is in Africa, with 94% of cases being found in this region.^2^
- Malaria is diagnosed by visualization of parasites in blood smears under light microscopy, or by rapid diagnostic tests which detect specific malaria parasite antigens.

**High Risk Populations**

- Children 1 - 5 years old (lack of acquired functional immunity)
  - Children under 5 account for 76% of annual malaria deaths.^2^
- Pregnant Women (reduced immunity and placental complications)
- Travelers without previous exposure (no natural immunity)

**Uncomplicated Malaria**

- Febrile illness with fatigue, headache, anorexia, nausea/vomiting, diarrhea, myalgias and arthralgias.
- May have splenomegaly on exam, particularly in chronic cases.
- In addition to parasitemia, lab findings may include anemia, thrombocytopenia, elevated aminotransferases, elevated BUN and creatinine.

**Severe Malaria** - Includes one of the following features:

- Altered mental status (GCS < 11 or BCS < 3 in children)
- Metabolic Acidosis (CO2 < 15 mEq/L)
- Hypoglycemia (BGL < 40 mg/dL)
- Severe Anemia (Hgb < 5g/dL in children or < 7g/dL in adults)
- Renal Failure
- Pulmonary Edema
- Significant Bleeding
- Shock and Lactic Acidosis
- Hyperparasitemia ( > 5% in low or > 10% in high transmission areas)

**Cerebral Malaria**

- Mortality is high, with a case fatality rate of 10-20% even with timely treatment.^3^
- WHO Criteria include^3^:
  - Coma at least 30 minutes after termination of seizure or correction of hypoglycemia.
    - Blantyre Coma Score less than or equal to 2.
  - P. falciparum parasitemia (positive blood smears or rapid diagnostic test).
  - No alternative cause of coma present.

**Treatment for Severe Malaria (including Cerebral Malaria):**

- Start with IV therapy. Parenteral artesunate is preferred for all patients with severe malaria.^3^
- If the patient can tolerate oral medication after 24 hours of parenteral treatment, then transition to an oral regimen is acceptable.^3^
- A significant percentage of children with cerebral malaria may have a concomitant invasive bacterial infection, and parenteral antibiotics are typically indicated until meningitis or sepsis can be ruled out.^4^

**Blantyre Coma Scale**

|  | **Score** |
| --- | --- |
| **Eye Movement** |  |
| Watches or follows | 1 |
| Fails to watch or follow | 0 |
| **Best motor response** |  |
| Localizes painful stimulus | 2 |
| Withdraws limb from painful stimulus | 1 |
| No response or inappropriate response | 0 |
| **Best verbal response** |  |
| Cries appropriately with pain or, if verbal, speaks | 2 |
| Moan or abnormal cry with pain | 1 |
| No vocal response to pain | 0 |
| **Total Score** |  |

**References**

1. Reddy, D.S. and Volkmer, R. (2017) ‘Neurocysticercosis as an infectious acquired Epilepsy Worldwide’, *Seizure*, 52, pp. 176–181. doi:10.1016/j.seizure.2017.10.004.
2. World Health Organization. *World Malaria Report 2023*. World Health Organization; 2023. <https://www.who.int/publications/i/item/9789240086173>
3. Guidelines for the treatment of malaria. Third edition. WHO | Regional Office for Africa. Published 2017. <https://www.afro.who.int/publications/guidelines-treatment-malaria-third-edition>
4. Berkley JA. Cerebral malaria versus bacterial meningitis in children with impaired consciousness. *QJM*. 1999;92(3):151-157. doi:https://doi.org/10.1093/qjmed/92.3.151

**Labs**

These should be given to the participants when indicated in the instructor notes.

CBC

WBC 5.3 x10^9^/L

Hgb 7.7 g/dL

Plt 89 K/mm^3^

All other CBC values normal

BMP

Na 124 mEq/L

K 4.1 mEq/L

Cl 106 mEq/L

CO2 16 mEq/L

Glucose 110 mg/dL

BUN 12 mg/dL

Cr 1.0 mg/dL

Ca 9.0 mg/dL

Magnesium 2.0 mg/dL

Malaria RDT:

**
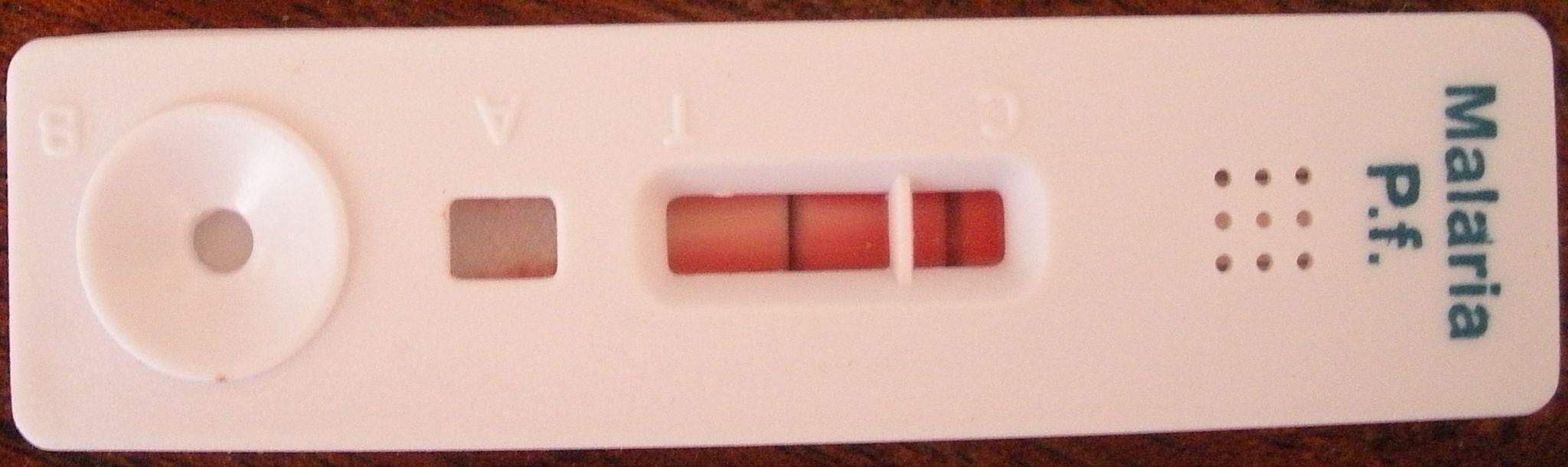
**

Author’s own image
