# Supplementary material for: Advancing Global Health Education: Preparing Emergency Medicine Trainees for Low-Resource Settings Through Simulation-Based Training
Source: MedEdPORTAL. 2026 Mar 10;22:11582. doi: 10.15766/mep_2374-8265.11582 (PMC12972016; doi:10.15766/mep_2374-8265.11582)
Supplement: Supplementary file 1 — Equipment for Implementation.docxTraumatic Hemopneumothorax Case.docxTuberculous Pericarditis Case.docxCerebral Malaria Case.docxOrganophosphate Poisoning Case.docxPostpartum Hemorrhage Case.docxLecture.pptxCourse Evaluation.docx [file mep_2374-8265.11582-s001.zip › F. Postpartum Hemorrhage Case.docx]

Appendix F. Postpartum Hemorrhage

Purpose: This appendix contains the complete facilitator-facing simulation case focused on recognition and management of postpartum hemorrhage with hemorrhagic shock in a resource-limited setting.

When and How to Use: Facilitators should review this appendix prior to the session and use it as a step-by-step guide during simulation delivery and debriefing. Instructor notes outline expected learner actions, case progression, and prompts. Labs and diagnostics should be released only when indicated. Debriefing materials support discussion of postpartum hemorrhage etiologies, initial resuscitation, uterotonics and TXA use, and low-resource hemorrhage control options (e.g., packing or improvised balloon tamponade) while awaiting definitive care.

| **SIMULATION CASE TITLE: Management of Shock Secondary to Postpartum Hemorrhage (PPH)**  **AUTHORS: Julianne Jett, MD, Halley J. Alberts, MD, Heather A. Brown, MD, Christopher Gainey, MD, Joshua Skaggs, MD**  **LEARNER AUDIENCE: Emergency Medicine Residents and Medical Students** | |
| --- | --- |
| **PATIENT NAME: Gloria**  **PATIENT AGE: 25 year old female**  **CHIEF COMPLAINT: Vaginal Bleeding**  **PHYSICAL SETTING: District hospital in Uganda with limited laboratory capabilities, no advanced imaging and minimal specialist coverage (OBGYN, general surgeon only)** | |
|  | |
| **Brief Narrative Description of Case** | *25-year-old G3P3 female brought into a district hospital by her family members accompanied by a village midwife. Family reports that she had just given birth at home about two hours prior and has continued vaginal bleeding despite uterine massage at home. Pregnancy was assumedly uncomplicated with scheduled midwife visits. Delivery was without event and the baby appeared appropriate and crying at birth.* |
| **Primary Learning Objectives** | By the end of this activity, learners will be able to:   - Recognize and manage hemorrhagic shock - Discuss causes of postpartum hemorrhage - Source control in postpartum hemorrhage - Use appropriate medications for postpartum hemorrhage |
| **Critical Actions** | - Establish large bore IV access - Begin fluid resuscitation promptly - Recognize the need for blood transfusion early - Consider all causes of postpartum hemorrhage - Explore hemostatic options |
| **Learner Preparation or Prework** | Active participation in continued medical knowledge and skills via status as Medical Student Year 3 or 4 or Emergency Medicine Resident Year 1-4 |

| **Initial Presentation** | | | |
| --- | --- | --- | --- |
| **Initial Vital Signs** | BP 85/54, HR 132, O2 94%, 37.6C (if requested), RR 19 | | |
| **Overall Setting and Appearance** | **Setting:** Emergency department room with stretcher and cardiac monitor, O2 available  **Mannequin:** Obstetric mannequin lying on stretcher  **Equipment**: packing agents such as 4x4 gauze, forceps, oxytocin, misoprostol, TXA, medication tubes to represent topical hemostatic agents if requested, equipment for makeshift balloon tamponade device | | |
| **Standardized Participants (and Their Roles in the Room at Case Start)** | **Midwife:** voiced by instructor, not played by an actor in the room. Provides OB and birth history  **The patient:** Voiced by instructor. Mostly just moans but can answer yes and no questions.  **Nurse:** Voiced by instructor, gives prompts when needed as specified below in instructor’s notes. | | |
| **HPI** | The patient is brought in by her family and midwife. She just delivered her third child about two hours prior to presentation. She continued to have vaginal bleeding despite uterine massage attempted by the midwife. She began to get lightheaded and confused so the family brought her to the hospital.  If asked on ROS: Patient feels lightheaded and short of breath. She reports lower abdominal and back pain. No recent illnesses. No HA, vision changes/neuro symptoms. The baby is doing well. | | |
| **Past Medical/Surgical History** | **Medications** | **Allergies** | **Family History** |
| G3P3 with good prenatal care from midwife  No tobacco, alcohol, or drug use | Vitamin at times | None | No known history of bleeding disorders or cancers, if requested |
| **Physical Examination** | | | |
| **General** | Patient is well developed, well nourished, lying on stretcher groaning, appears pale and uncomfortable | | |
| **HEENT** | Pupils 3 mm, equal, round and reactive. | | |
| **Neck** | Midline trachea, no JVD | | |
| **Lungs** | Mildly tachypneic, clear lung fields | | |
| **Cardiovascular** | Tachycardic, no murmurs, thready peripheral pulses | | |
| **Abdomen** | Lower abdominal tenderness to palpation, bowel sounds intact, no peritoneal signs | | |
| **Neurological** | She is awake with decreased alertness, but able to answer questions, moving all extremities, oriented | | |
| **Skin** | Clammy extremities | | |
| **GU** | Brisk bright red vaginal bleeding with passage of 1 cm clots, postpartum cervix dilated to 7 cm, boggy uterus, no evidence of retained products | | |
| **Psychiatric** | Appropriate | | |

| **Instructor Notes - Changes and CASE Branch Points** | | |
| --- | --- | --- |
| **Intervention / Time Point** | **Potential Actions/Change in Case** | **Additional Information** |
| *T0-T1* | Participants obtain history | If IV access/monitor is requested, the nurse should say, “I am working on it.” |
| *T1-T2* | Participants can request vital signs. Nurse provides above vitals and connects patient to the monitor  IV access obtained |  |
| *T2-T4* | Physical exam performed | If no fluids administered in the previous step, BP should drop to 79/50.  The nurse can ask, “Do you want her to get any fluids?”  If temperature is not requested, the nurse can say, “She feels warm.” |
| *T4-T6* | If fluids were started, patient’s BP improves slightly (82/54), HR 122  Learners can request basic labs:   - CBC - not immediately available - POC glucose - 93mg/dL - UA - not immediately available, but appears bloody - Type and screen can be sent   If US requested, bedside ultrasound shows a normal postpartum uterus  If EKG requested, shows sinus tachycardia  If OBGYN is consulted, they are en route but 30 minutes out. | If learners have not requested an US, the nurse can say, “Do you want the ultrasound?” |
| *T6-T7* | Participants may begin to request interventions:   - Additional fluids - TXA - Oxytocin - Misoprostol - Blood should be requested but is not immediately available - Antibiotics (ceftriaxone and metronidazole are available) - Can place on oxygen | If fluids are not administered, the nurse can say, “Her repeat blood pressure is 81/64.”  TXA takes 10 minutes for the courier to get  If blood is requested, the nurse says, “I will send a courier to get blood.”  If more than two IV interventions are requested, the nurse should say “I’m going to need help with IV access here.” |
| *T7-T8* | Lab results are given to participants | The nurse says, “Courrier is coming with blood.” (30 minutes) |
| T8-15 | - Learners should try to control the bleeding - Should attempt uterine massage - TXA, oxytocin, misoprostol are available - Improvised uterine balloon tamponade with miscellaneous items provided - packing, Blakemore, condom, foley etc. |  |
|  | Patient’s BP stabilizes after several attempts, vaginal bleeding stops or OBGYN arrives |  |

**Ideal Scenario Flow**

*A 25-year-old Ugandan G3P3 presents to a district hospital with limited capabilities two hours after giving birth at home attended by a midwife. The patient continues to have significant vaginal bleeding and appears pale and uncomfortable with clammy extremities and a thready pulse. She is found to be tachycardic and hypotensive with brisk bleeding from the cervix. IV is established and the patient is resuscitated with IVF while blood transfusion is being arranged. OBGYN is consulted early but not readily available so the patient is given uterine massage, TXA, oxytocin, misoprostol and antibiotics. The patient continues to bleed so source control is attempted with uterine packing or improvised uterine balloon tamponade. OBGYN arrives and takes the patient to the operating room for definitive care.*

**Debriefing:**

- “How did that go?”
- Summary of the case - diagnosis, interventions, management
- Brief epidemiology
  - Nearly 800 women die every day from preventable causes related to pregnancy and childbirth. 95% of those occurred in LMICs^1^.
  - PPH is the leading cause of maternal deaths worldwide, affecting about 14 million women each year and killing 70,000^2^.
  - From 2003-2009, 27.1% of maternal deaths worldwide were due to hemorrhage^3^.
  - Other causes: HIV, birth obstruction, sepsis/infections.
- What is PPH?
  - ≥ 1,000 mL of blood loss accompanied by signs or symptoms of hypovolemia within 24 hours after the birth process^4^.
- Causes of PPH: Consider the 4 Ts
  - Tone - LEADING cause of PPH. Uterus will feel “boggy” on bimanual exam. Uterine massage was unsuccessful in this case, but should always be attempted. Having the infant breastfeed can stimulate oxytocin release and may help improve tone.
  - Trauma - Check for signs of trauma on exam. Ensure uterus is intact with bedside ultrasound.
  - Tissue - Check for retained products on exam and with bedside US.
  - Thrombin - Bleeding diathesis.
    - “Can you think of any pregnancy-related processes that increase risk of bleeding?”
    - Especially consider thrombocytopenia 2/2 HELLP or preeclampsia with severe features
- Management of PPH
  - Medications
    - Oxytocin
      - Uterotonic
      - IM injection (10 units) or IV infusion (10-40 U in 500-1000mL of normal saline, rate titrated based on uterine tone, with max rate of 500mL/hr). IV bolus dose has questionable safety^5^.
      - Side effects: Hypotension and tachycardia with high doses especially IV push, hyponatremia with prolonged infusion
        - Oxytocin (10 IU intravenously/intramuscularly [IV/IM]) is recommended for the **prevention** of PPH for vaginal delivery and cesarean section
    - Misoprostol
      - Uterotonic, prostaglandin E1 analog
      - Oxytocin is more effective, but misoprostol has multiple advantages for low resource settings: heat stable, lower cost, and can be administered sublingually, orally, rectally, or vaginally.
      - Vaginal administration is not recommended for postpartum hemorrhage because heavy bleeding may impair absorption.
      - Added to WHO Model List of Essential Medicines (EML) in April 2015^6^
      - WHO recommends 800 mcg sublingually^7^
      - Side effects: Nausea, vomiting, diarrhea, fever, headache
    - Tranexamic acid (TXA)
      - Antifibrinolytic
      - 1g IV pushed over 10 minutes (100mg/mL, 1mL per minute)
      - Can give a second dose of 1g IV if bleeding continues after 30 minutes, or if bleeding restarts within 24 hours of completing the first dose
      - World Maternal Antifibrinolytic Trial found that TXA reduced death from PPH by 20-30%^8^.
  - Tamponade techniques
    - Packing
    - Uterine balloon tamponade device (Bakri): 500 +/- 200 mL sterile saline or sterile water
    - Get creative: Improvised uterine balloon tamponade with condom and Foley catheter^9,10^
      - Small studies with improvised uterine balloon tamponade suggest efficacy in bleeding control and preventing hysterectomy^9,10^

References

1. Maternal Mortality. World Health Organization. Accessed March 1, 2023. https://www.who.int/news-room/fact-sheets/detail/maternal-mortality#:~:text=Key%20facts,dropped%20by%20about%2034%25%20worldwide
2. WHO Postpartum Haemorrhage (PPH) Summit: Current Project Brief. World Health Organization. Accessed March 1, 2023. https://cdn.who.int/media/docs/default-source/hrp/projects/mph/project-brief-pph-summit.pdf?sfvrsn=3b0e505a_6&download=true
3. Say L, Chou D, Gemmill A, et al. Global causes of maternal death: A who systematic analysis. *The Lancet Global Health*. 2014;2(6):323-333. doi:10.1016/s2214-109x(14)70227-x
4. American postpartum hemorrhage. American College of Obstetricians and Gynecologists. Accessed March 1, 2023. https://www.acog.org/clinical/clinical-guidance/practice-bulletin/articles/2017/10/postpartum-hemorrhage.
5. Berghella, V. Management of the third stage of labor: prophylactic pharmacotherapy to minimize hemorrhage. In: UpToDate, Post, TW (Ed), UpToDate, Waltham, MA, 2023.
6. WHO model list of essential medicines - 22nd list, 2021. World Health Organization. September 30, 2021. Accessed March 1, 2023. https://www.who.int/publications/i/item/WHO-MHP-HPS-EML-2021.02.
7. WHO recommendations for the prevention and treatment of postpartum haemorrhage. World Health Organization. 2012. Accessed March 1, 2023. https://iris.who.int/bitstream/handle/10665/75411/9789241548502_eng.pdf
8. WOMAN Trial Collaborators. Effect of early tranexamic acid administration on mortality, hysterectomy, and other morbidities in women with post-partum haemorrhage (WOMAN): an international, randomised, double-blind, placebo-controlled trial. Lancet 2017; 389:2105
9. Mishra N, Gulabani K, Agrawal S, Shrivastava C. Efficacy and Feasibility of Chhattisgarh Balloon and Conventional Condom Balloon Tamponade: A 2-Year Prospective Study. J Obstet Gynaecol India. 2019;69(2):133-141. doi: 10.1007/s13224-018-1185-6.
10. Lothe SM, Bhalerao A*.* Condom-based Uterine Balloon Tamponade: An Innovation in the Management of Postpartum Hemorrhage. J South Asian Feder Obst Gynae 2022;14(5):557–562.

**Labs**

These should be given to the participants when indicated in the instructor notes.

- CBC
  - WBC 8.7 x10^9^/L
  - Hgb 7.2 g/dL
  - Plt 198 K/mm^3^
- POC glucose 93 mg/dL
- UA
  - Amber
  - Cloudy
  - Leukocyte esterase: trace
  - Nitrite: negative
  - WBC: 5 per high-power field (hpf)
  - RBC: >183 per hpf
  - Glucose negative
  - Ketones negative
  - Bacteria rare
- Blood type: O+


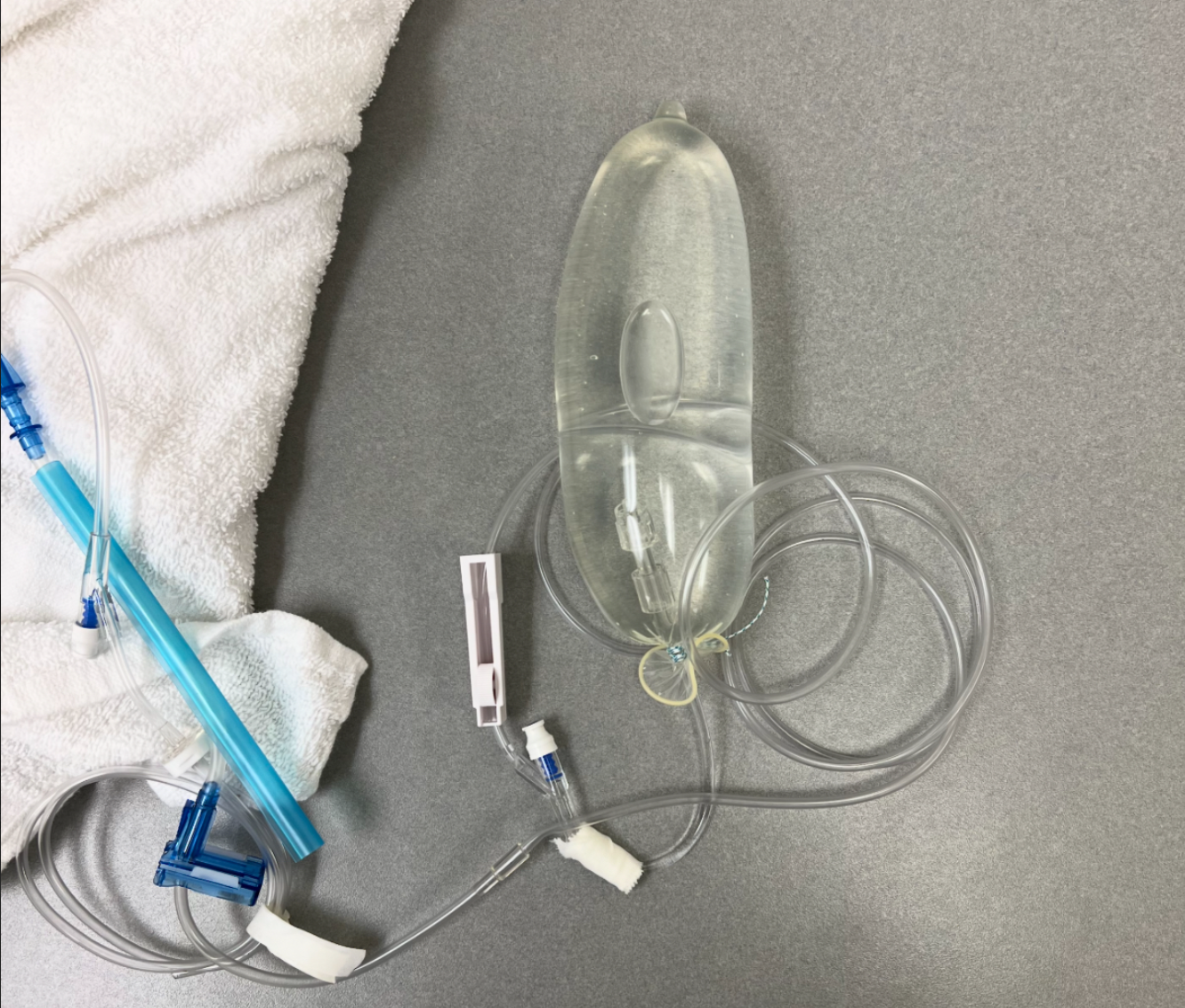


Author owned. Bakri balloon created using a condom, IV tubing, string, and water during simulation day.


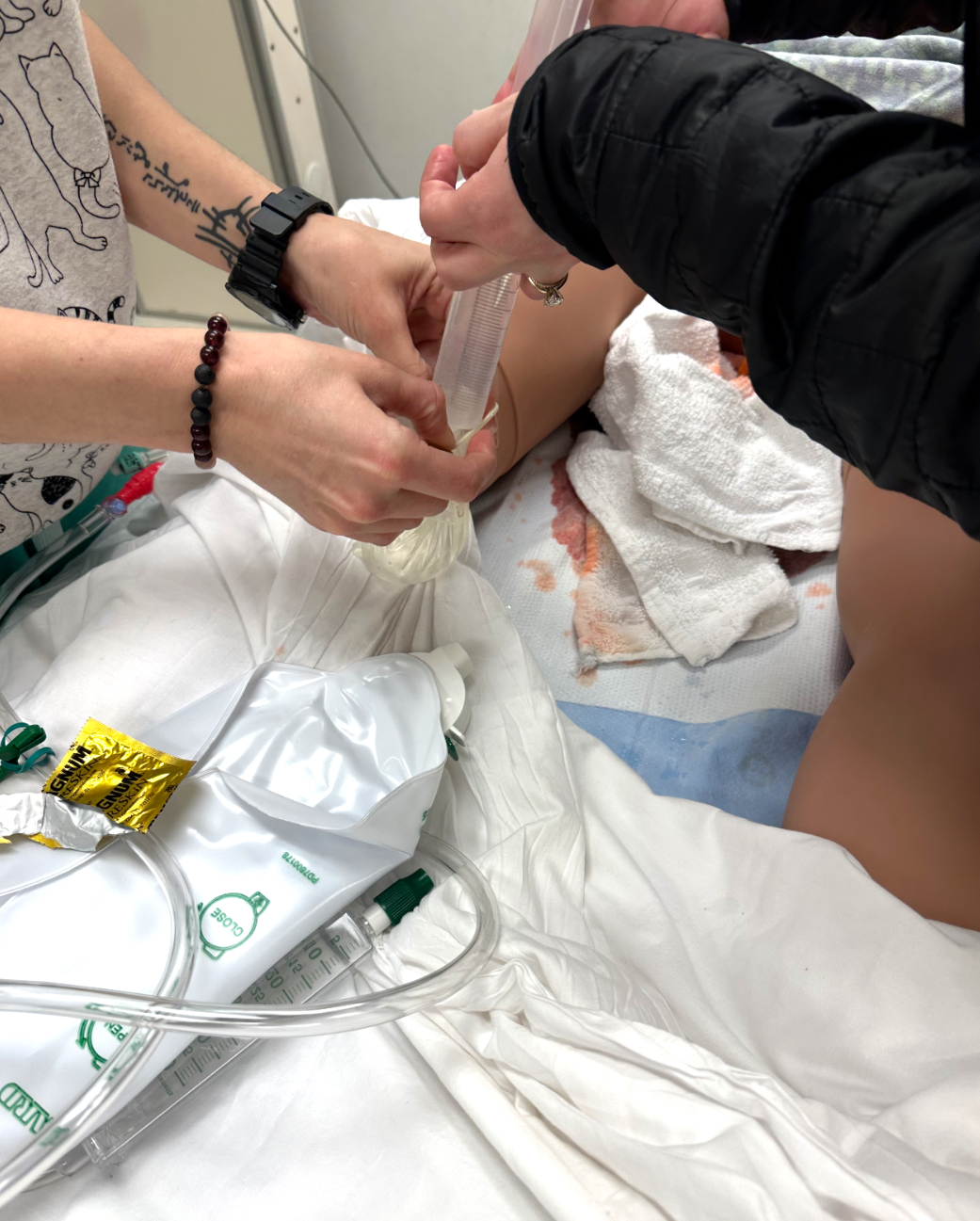


Author owned. Learners create a tamponade device with a foley bag and condom to control postpartum hemorrhage.
