# Supplementary material for: Advancing Global Health Education: Preparing Emergency Medicine Trainees for Low-Resource Settings Through Simulation-Based Training
Source: MedEdPORTAL. 2026 Mar 10;22:11582. doi: 10.15766/mep_2374-8265.11582 (PMC12972016; doi:10.15766/mep_2374-8265.11582)
Supplement: Supplementary file 1 — Equipment for Implementation.docxTraumatic Hemopneumothorax Case.docxTuberculous Pericarditis Case.docxCerebral Malaria Case.docxOrganophosphate Poisoning Case.docxPostpartum Hemorrhage Case.docxLecture.pptxCourse Evaluation.docx [file mep_2374-8265.11582-s001.zip › G. Lecture.pptx]

## Slide 1
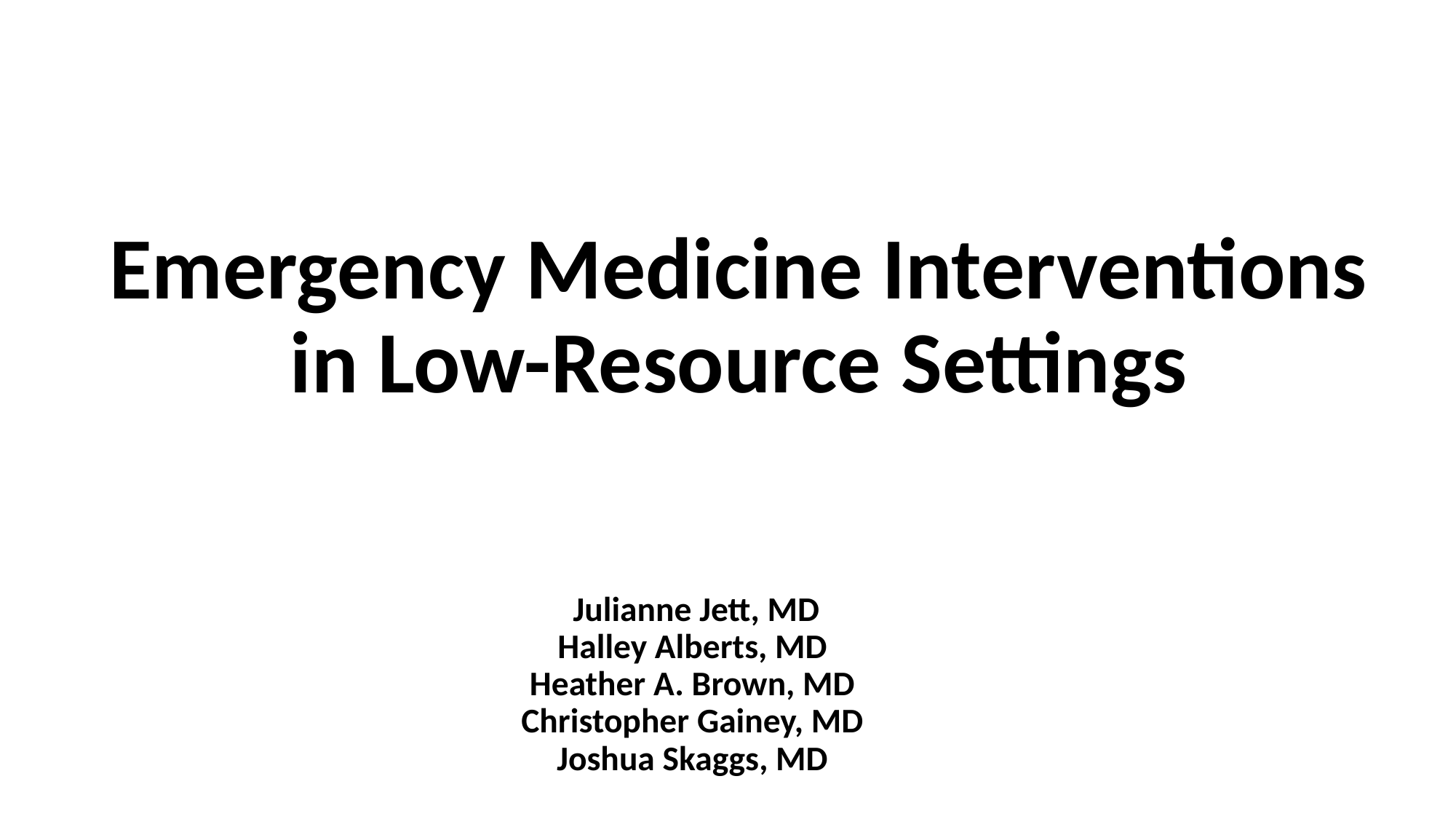

# Emergency Medicine Interventions in Low-Resource Settings
 Julianne Jett, MD
Halley Alberts, MD
Heather A. Brown, MD
Christopher Gainey, MD
Joshua Skaggs, MD

## Slide 2
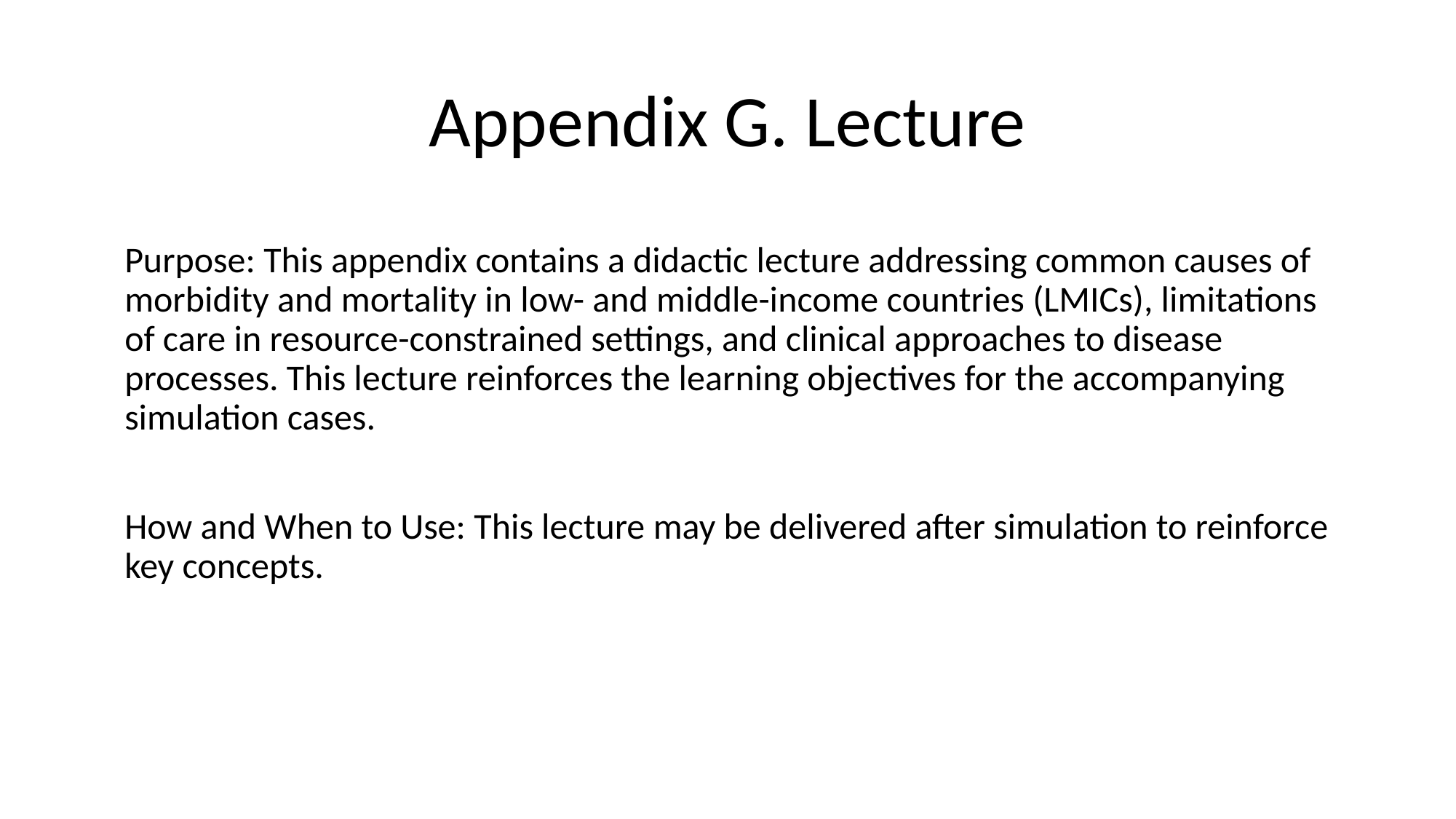

# Appendix G. Lecture
Purpose: This appendix contains a didactic lecture addressing common causes of morbidity and mortality in low- and middle-income countries (LMICs), limitations of care in resource-constrained settings, and clinical approaches to disease processes. This lecture reinforces the learning objectives for the accompanying simulation cases.
How and When to Use: This lecture may be delivered after simulation to reinforce key concepts.

## Slide 3
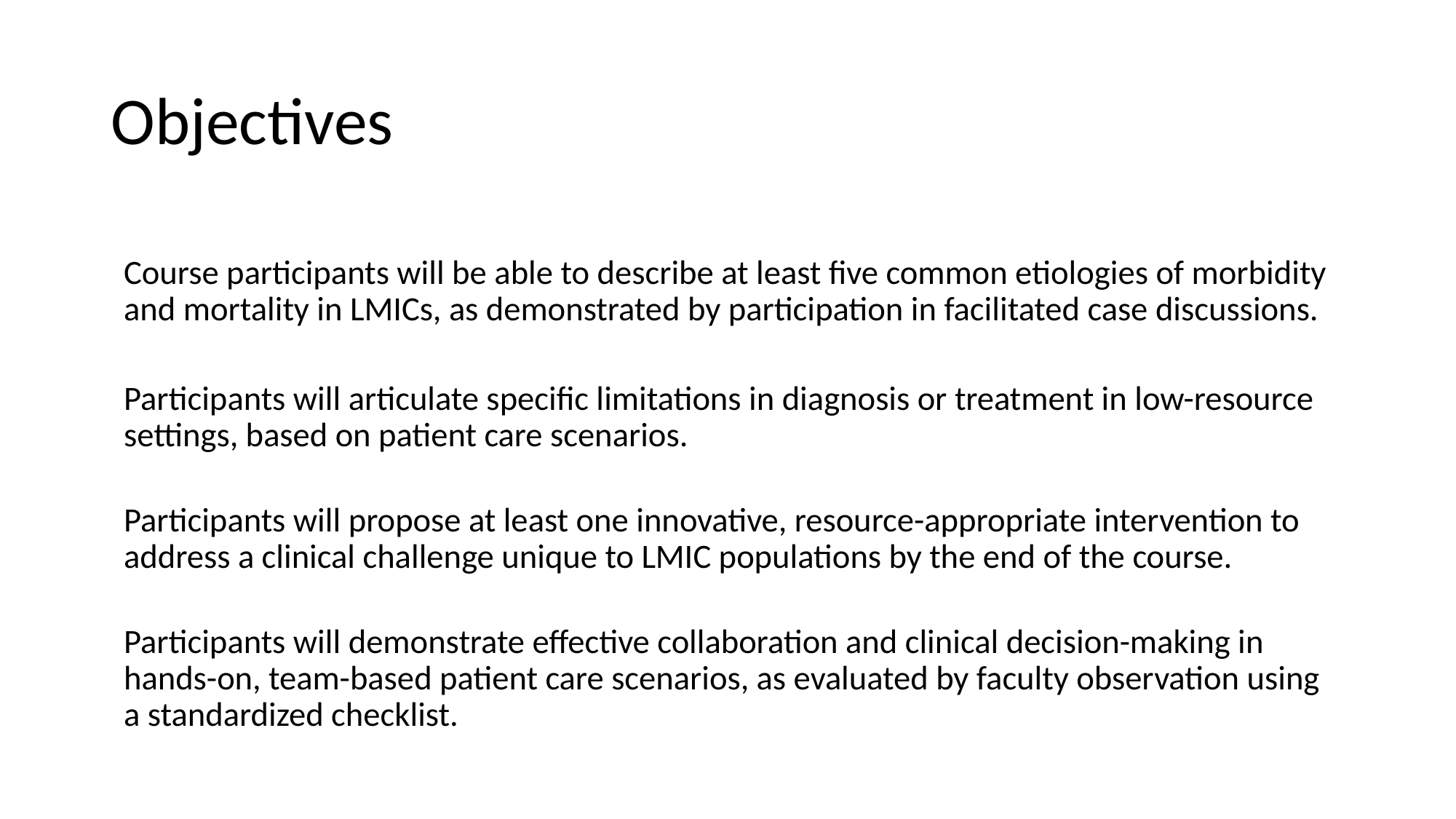

# Objectives
Course participants will be able to describe at least five common etiologies of morbidity and mortality in LMICs, as demonstrated by participation in facilitated case discussions.
Participants will articulate specific limitations in diagnosis or treatment in low-resource settings, based on patient care scenarios.
Participants will propose at least one innovative, resource-appropriate intervention to address a clinical challenge unique to LMIC populations by the end of the course.
Participants will demonstrate effective collaboration and clinical decision-making in hands-on, team-based patient care scenarios, as evaluated by faculty observation using a standardized checklist.

## Slide 4
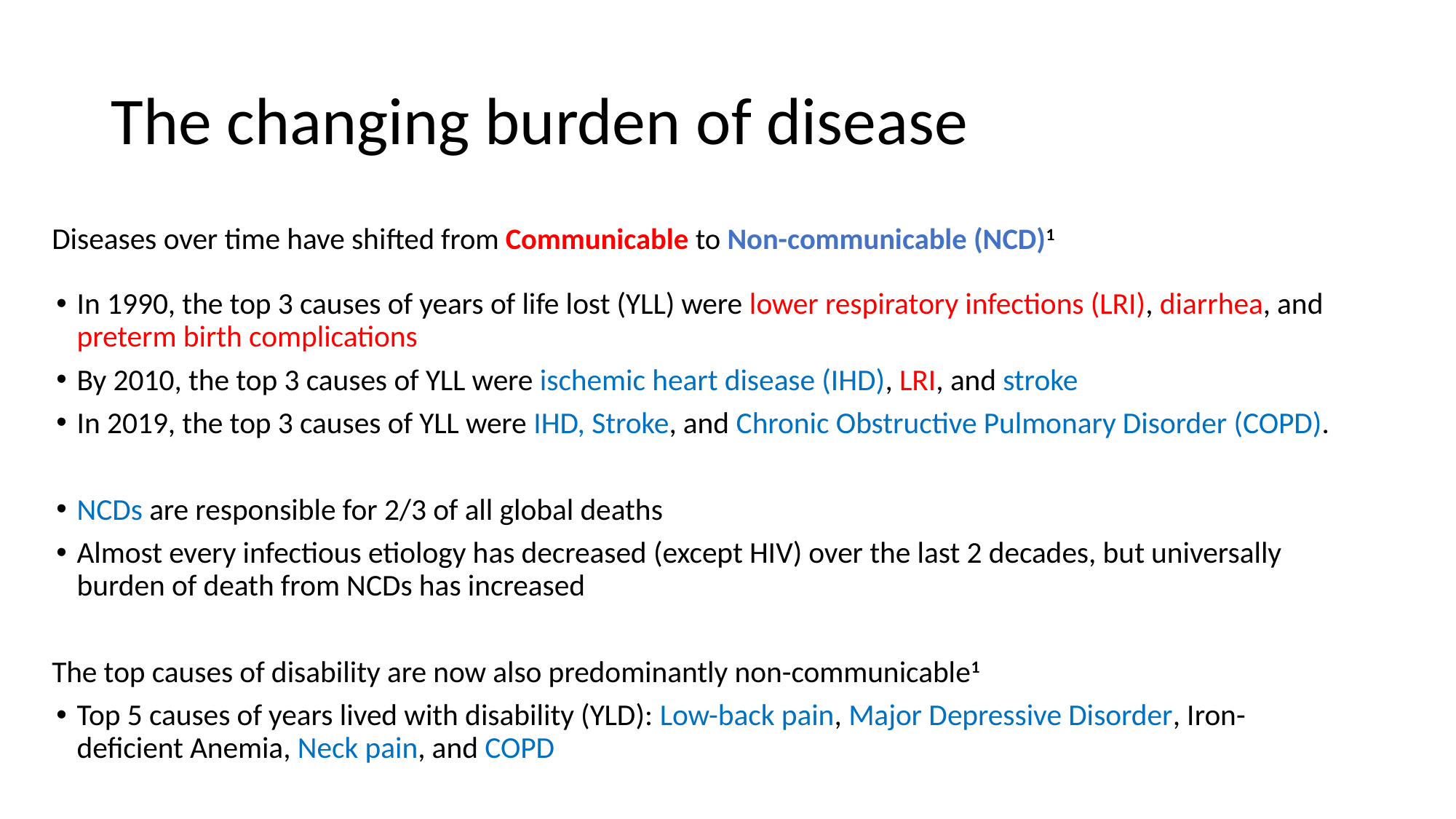

# The changing burden of disease
Diseases over time have shifted from Communicable to Non-communicable (NCD)1
In 1990, the top 3 causes of years of life lost (YLL) were lower respiratory infections (LRI), diarrhea, and preterm birth complications
By 2010, the top 3 causes of YLL were ischemic heart disease (IHD), LRI, and stroke
In 2019, the top 3 causes of YLL were IHD, Stroke, and Chronic Obstructive Pulmonary Disorder (COPD).
NCDs are responsible for 2/3 of all global deaths
Almost every infectious etiology has decreased (except HIV) over the last 2 decades, but universally burden of death from NCDs has increased
The top causes of disability are now also predominantly non-communicable1
Top 5 causes of years lived with disability (YLD): Low-back pain, Major Depressive Disorder, Iron-deficient Anemia, Neck pain, and COPD

## Slide 5
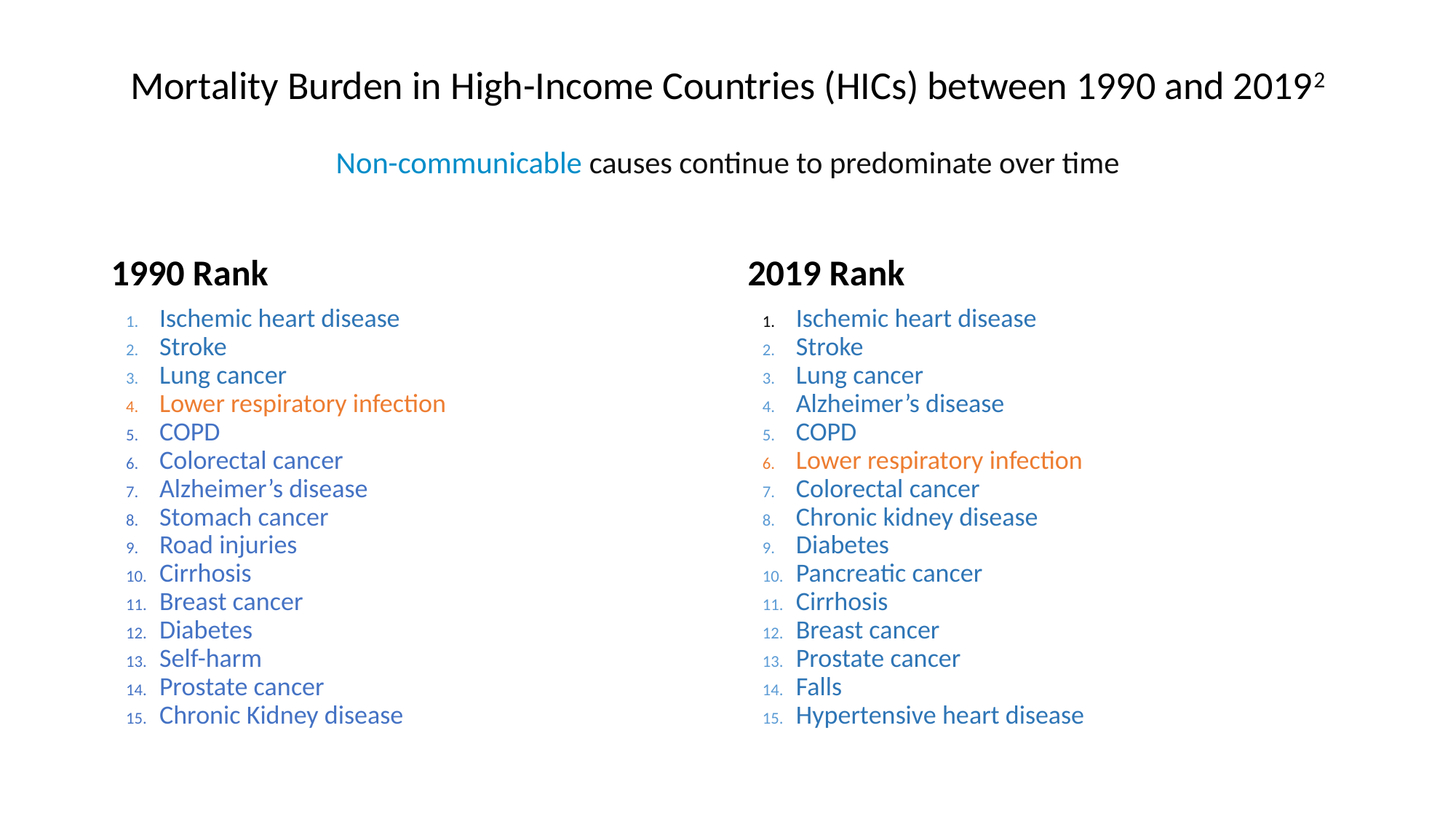

# Mortality Burden in High-Income Countries (HICs) between 1990 and 20192
Non-communicable causes continue to predominate over time
1990 Rank
2019 Rank
Ischemic heart disease
Stroke
Lung cancer
Lower respiratory infection
COPD
Colorectal cancer
Alzheimer’s disease
Stomach cancer
Road injuries
Cirrhosis
Breast cancer
Diabetes
Self-harm
Prostate cancer
Chronic Kidney disease
Ischemic heart disease
Stroke
Lung cancer
Alzheimer’s disease
COPD
Lower respiratory infection
Colorectal cancer
Chronic kidney disease
Diabetes
Pancreatic cancer
Cirrhosis
Breast cancer
Prostate cancer
Falls
Hypertensive heart disease

## Slide 6
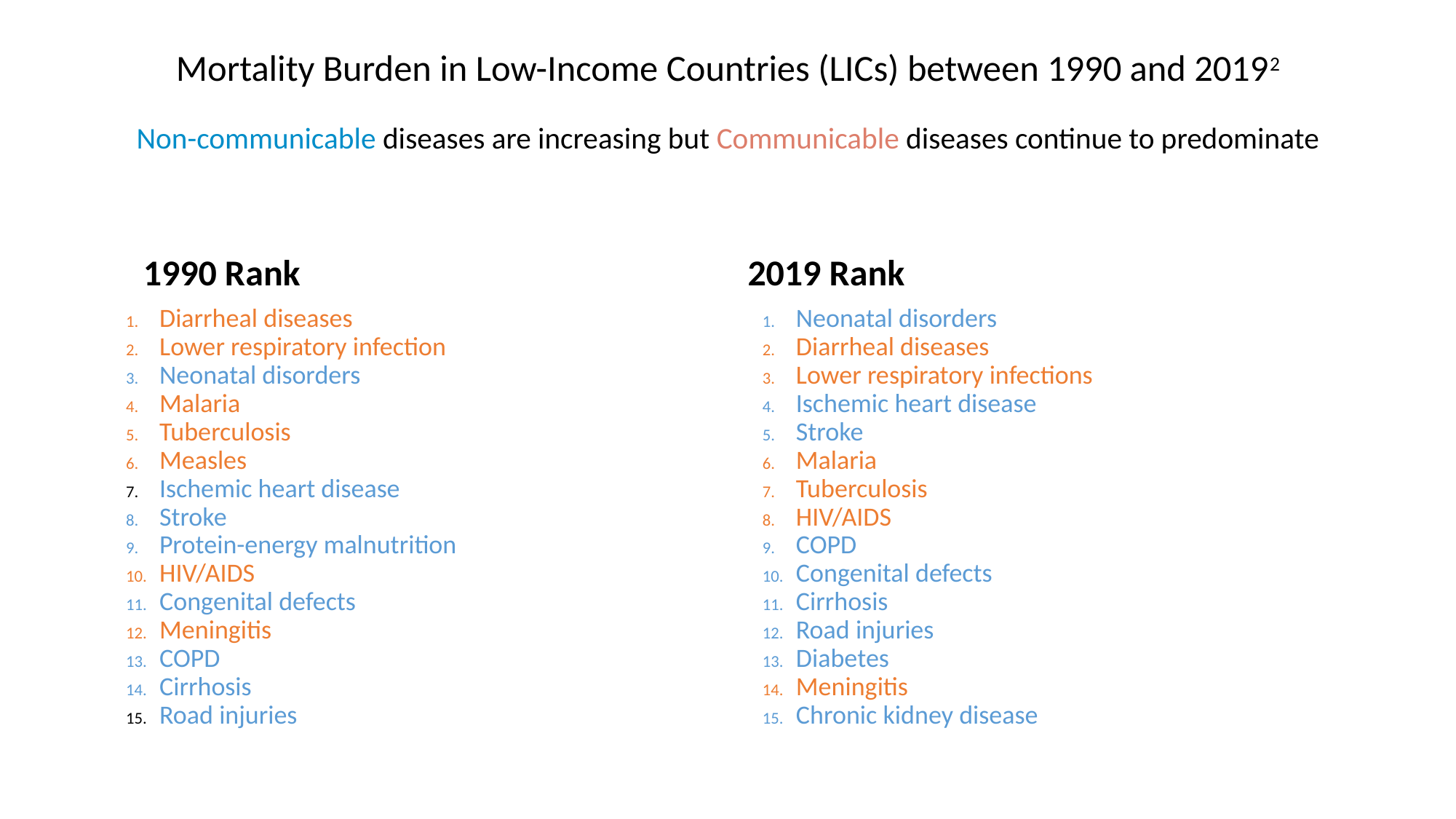

# Mortality Burden in Low-Income Countries (LICs) between 1990 and 20192
Non-communicable diseases are increasing but Communicable diseases continue to predominate
1990 Rank
2019 Rank
Diarrheal diseases
Lower respiratory infection
Neonatal disorders
Malaria
Tuberculosis
Measles
Ischemic heart disease
Stroke
Protein-energy malnutrition
HIV/AIDS
Congenital defects
Meningitis
COPD
Cirrhosis
Road injuries
Neonatal disorders
Diarrheal diseases
Lower respiratory infections
Ischemic heart disease
Stroke
Malaria
Tuberculosis
HIV/AIDS
COPD
Congenital defects
Cirrhosis
Road injuries
Diabetes
Meningitis
Chronic kidney disease

## Slide 7
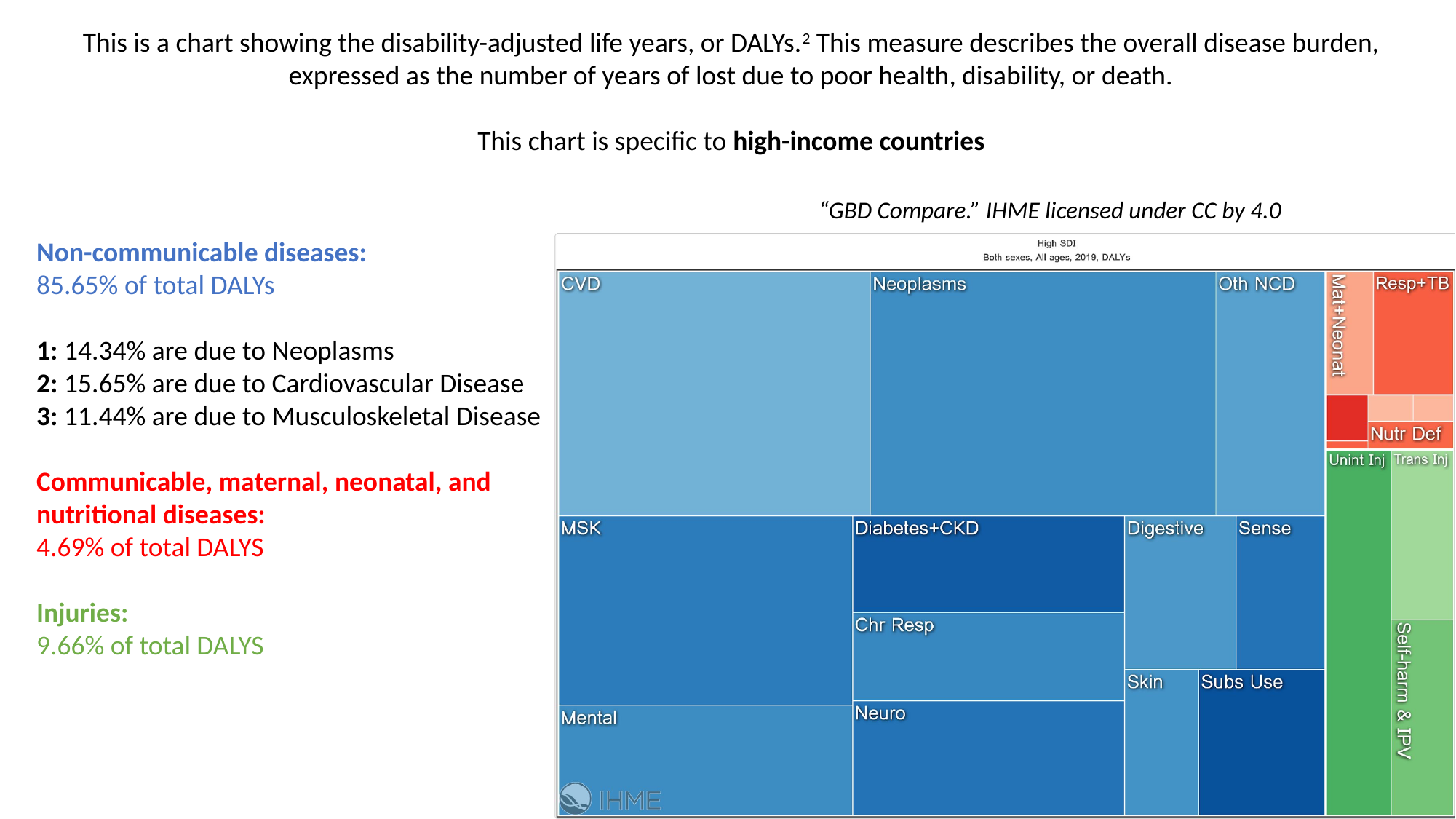

This is a chart showing the disability-adjusted life years, or DALYs.2 This measure describes the overall disease burden, expressed as the number of years of lost due to poor health, disability, or death.
This chart is specific to high-income countries
“GBD Compare.” IHME licensed under CC by 4.0
Non-communicable diseases:
85.65% of total DALYs
1: 14.34% are due to Neoplasms
2: 15.65% are due to Cardiovascular Disease
3: 11.44% are due to Musculoskeletal Disease
Communicable, maternal, neonatal, and nutritional diseases:
4.69% of total DALYS
Injuries:
9.66% of total DALYS

## Slide 8
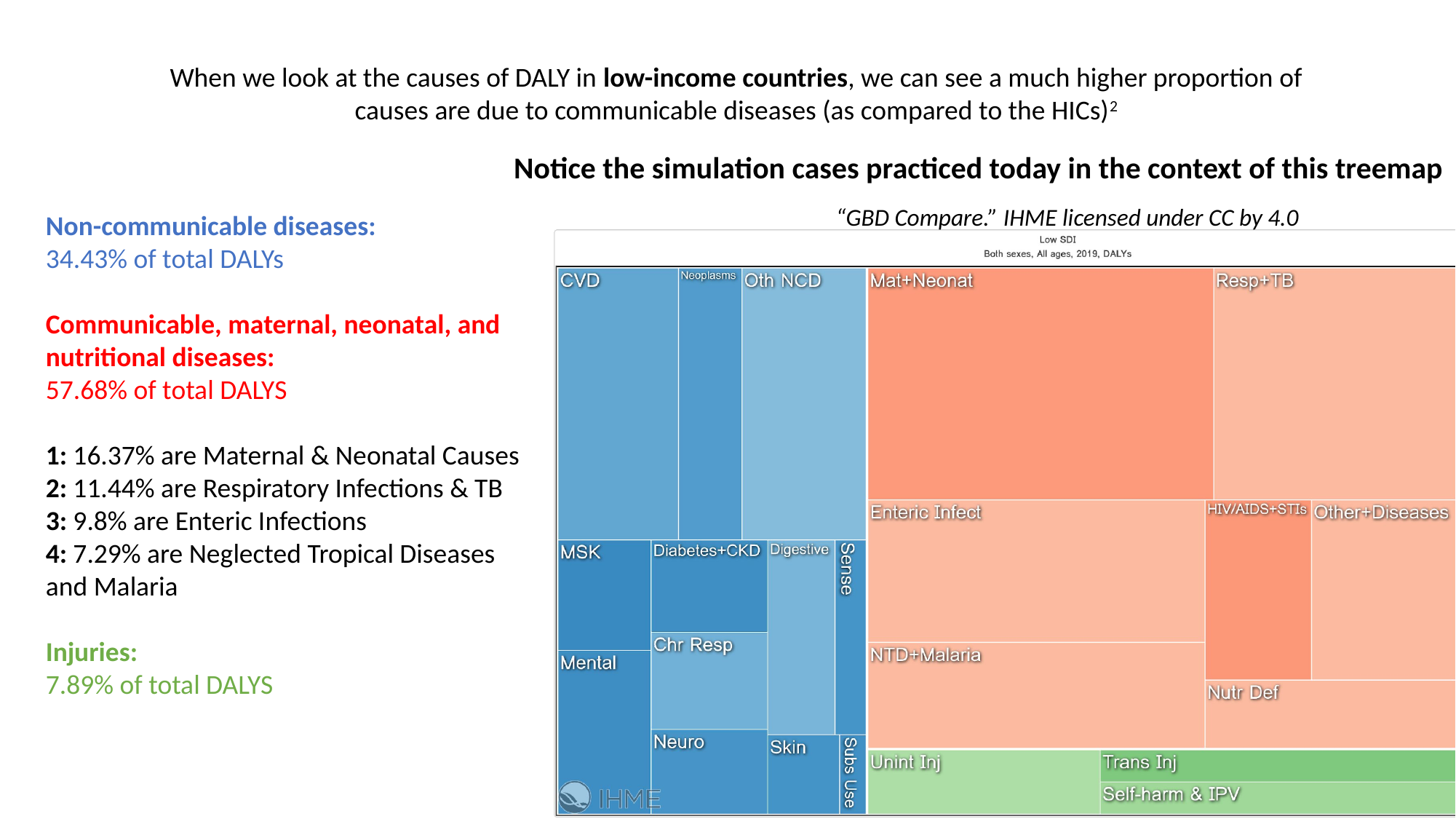

When we look at the causes of DALY in low-income countries, we can see a much higher proportion of causes are due to communicable diseases (as compared to the HICs)2
Notice the simulation cases practiced today in the context of this treemap
“GBD Compare.” IHME licensed under CC by 4.0
Non-communicable diseases:
34.43% of total DALYs
Communicable, maternal, neonatal, and nutritional diseases:
57.68% of total DALYS
1: 16.37% are Maternal & Neonatal Causes
2: 11.44% are Respiratory Infections & TB
3: 9.8% are Enteric Infections
4: 7.29% are Neglected Tropical Diseases and Malaria
Injuries:
7.89% of total DALYS

## Slide 9
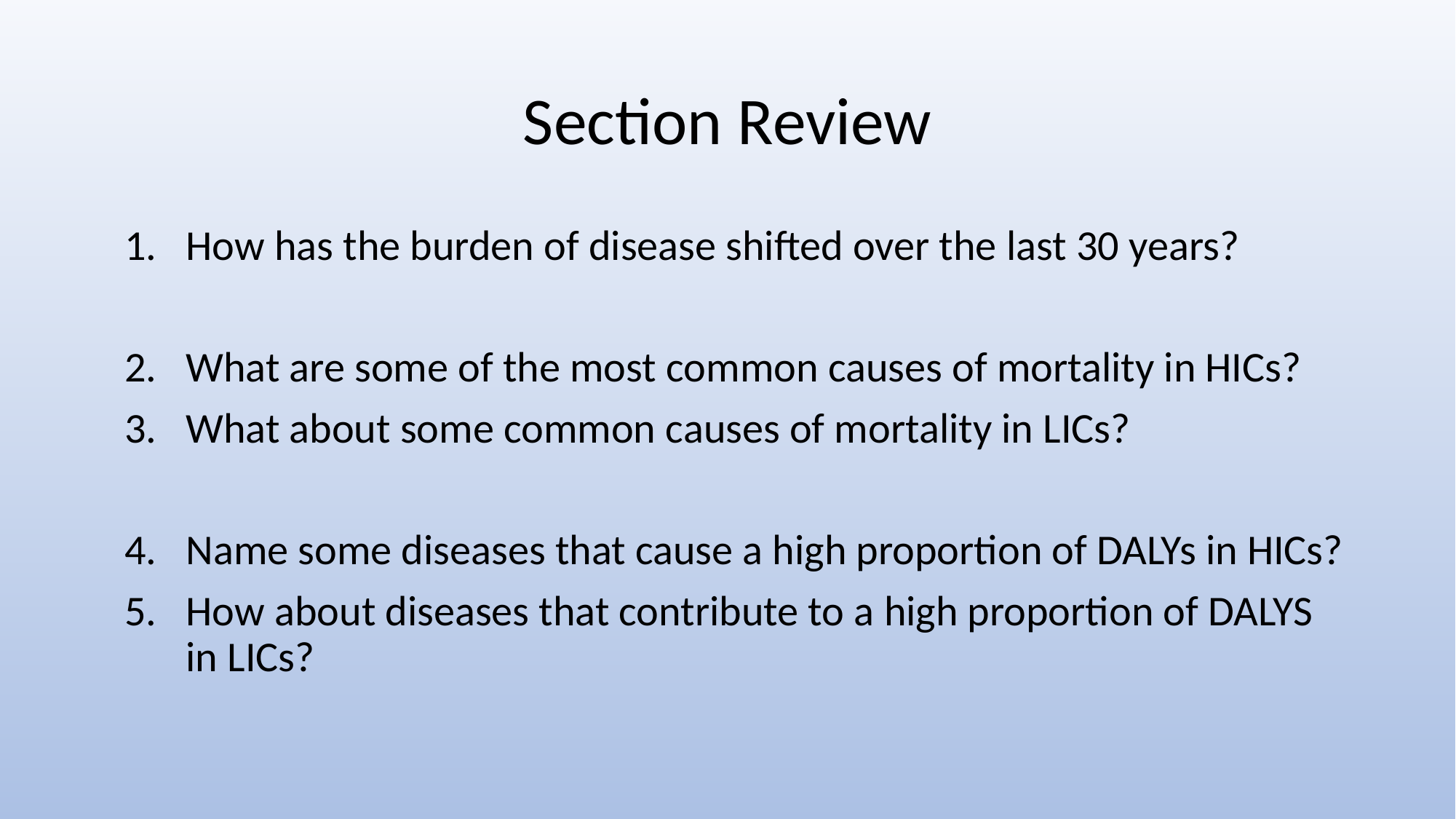

# Section Review
How has the burden of disease shifted over the last 30 years?
What are some of the most common causes of mortality in HICs?
What about some common causes of mortality in LICs?
Name some diseases that cause a high proportion of DALYs in HICs?
How about diseases that contribute to a high proportion of DALYS in LICs?

## Slide 10
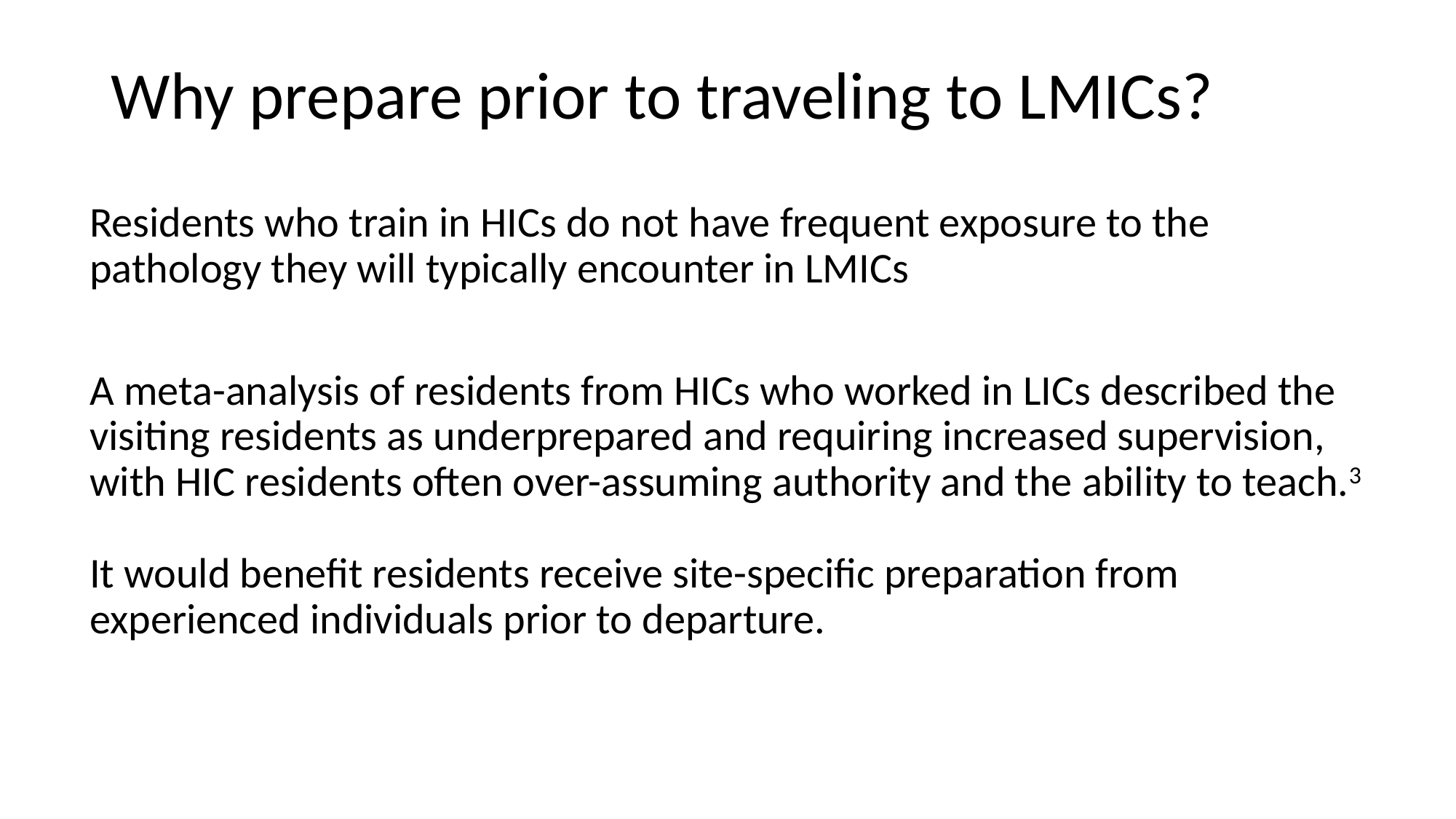

# Why prepare prior to traveling to LMICs?
Residents who train in HICs do not have frequent exposure to the pathology they will typically encounter in LMICs
A meta-analysis of residents from HICs who worked in LICs described the visiting residents as underprepared and requiring increased supervision, with HIC residents often over-assuming authority and the ability to teach.3
It would benefit residents receive site-specific preparation from experienced individuals prior to departure.

## Slide 11
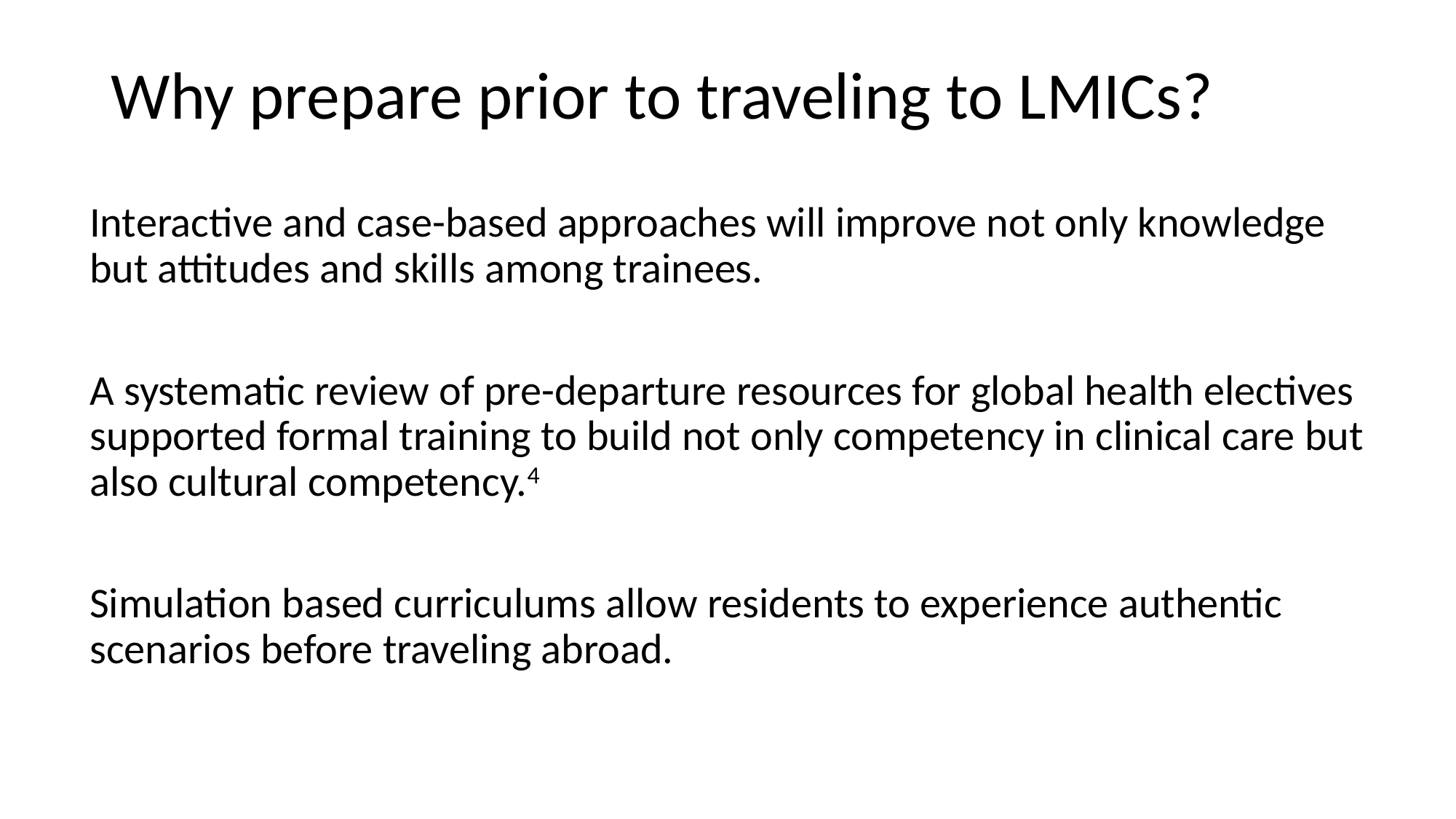

# Why prepare prior to traveling to LMICs?
Interactive and case-based approaches will improve not only knowledge but attitudes and skills among trainees.
A systematic review of pre-departure resources for global health electives supported formal training to build not only competency in clinical care but also cultural competency.4
Simulation based curriculums allow residents to experience authentic scenarios before traveling abroad.

## Slide 12
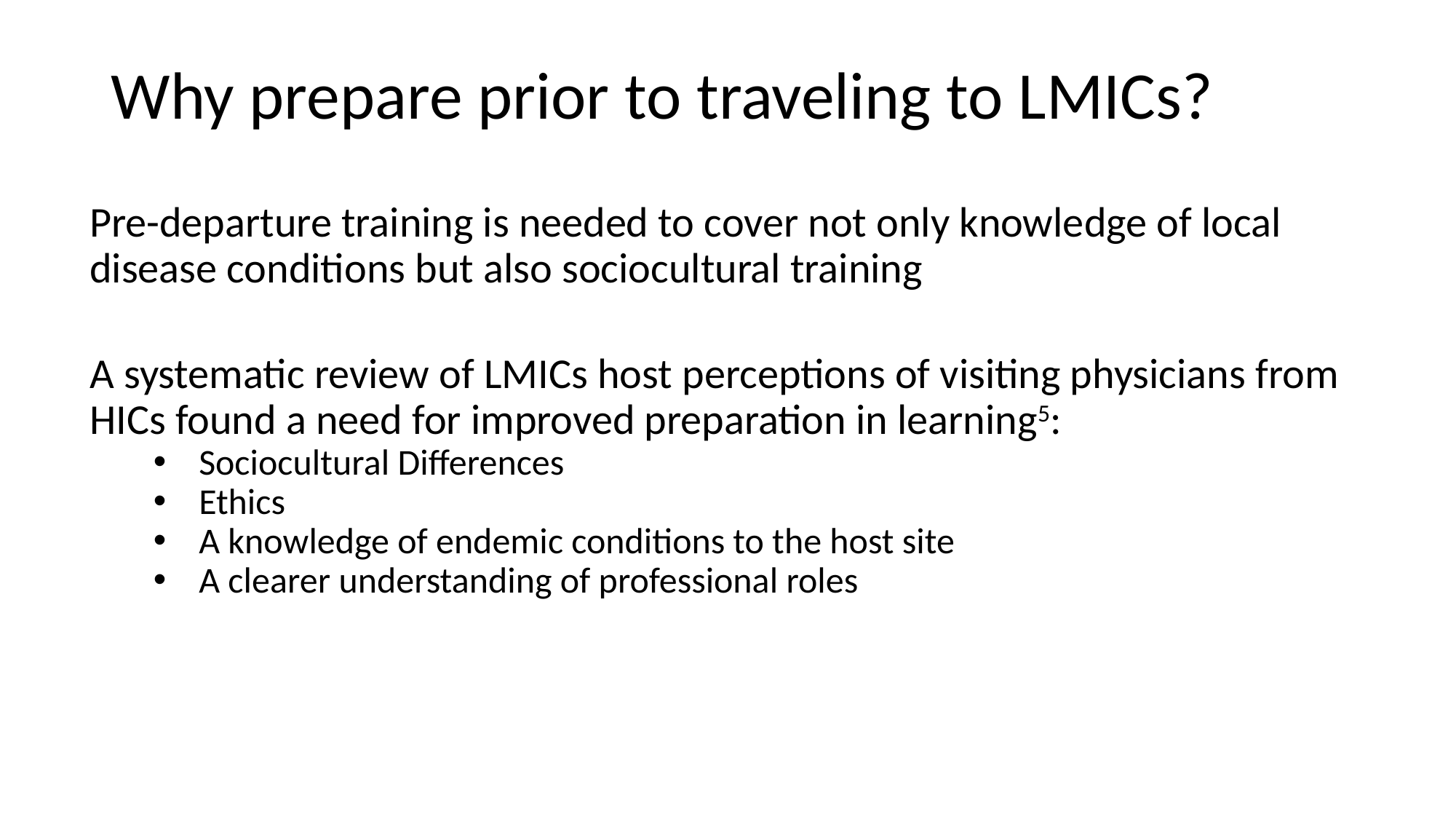

# Why prepare prior to traveling to LMICs?
Pre-departure training is needed to cover not only knowledge of local disease conditions but also sociocultural training
A systematic review of LMICs host perceptions of visiting physicians from HICs found a need for improved preparation in learning5:
Sociocultural Differences
Ethics
A knowledge of endemic conditions to the host site
A clearer understanding of professional roles

## Slide 13
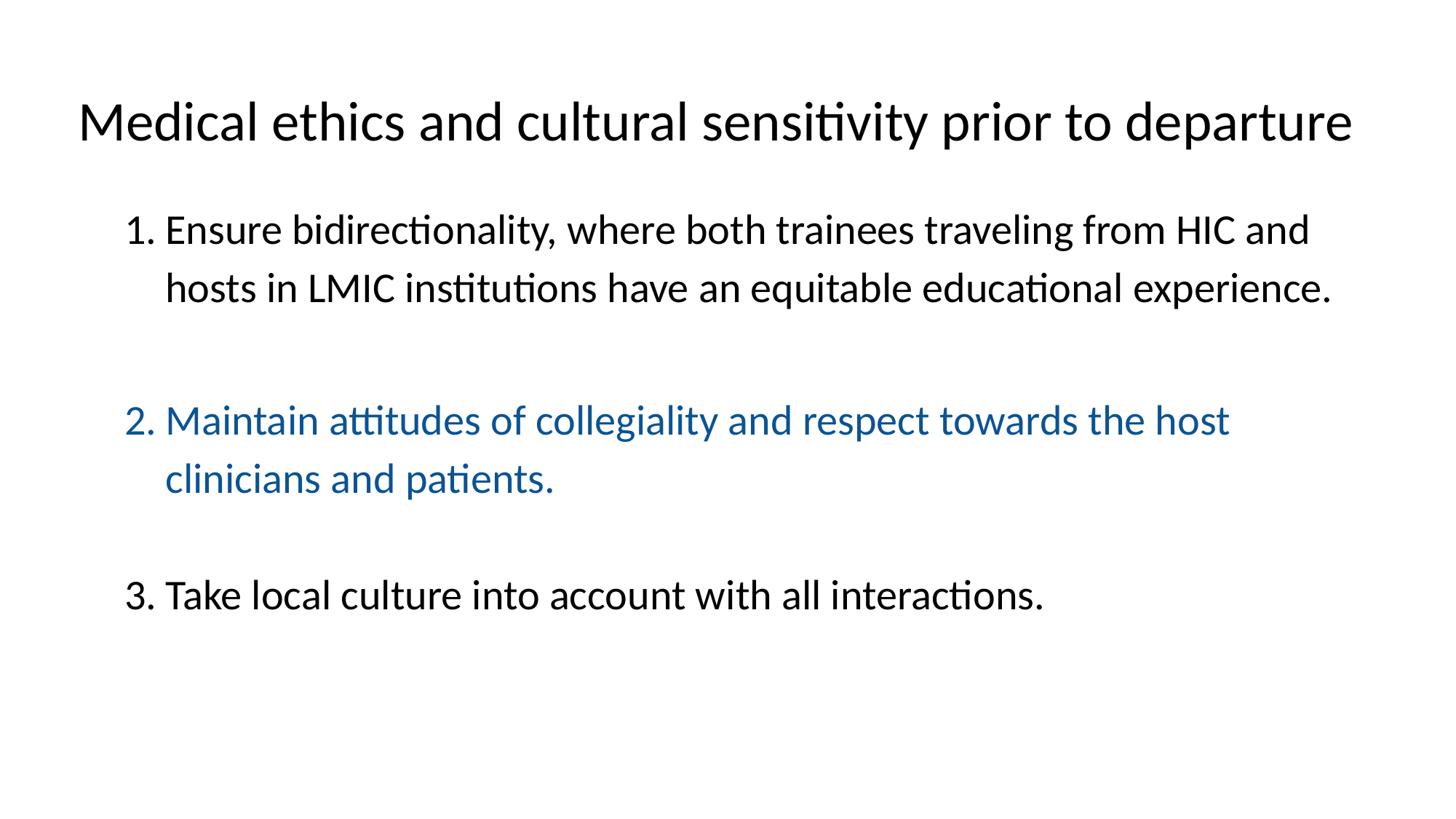

# Medical ethics and cultural sensitivity prior to departure
Ensure bidirectionality, where both trainees traveling from HIC and hosts in LMIC institutions have an equitable educational experience.
Maintain attitudes of collegiality and respect towards the host clinicians and patients.
Take local culture into account with all interactions.

## Slide 14
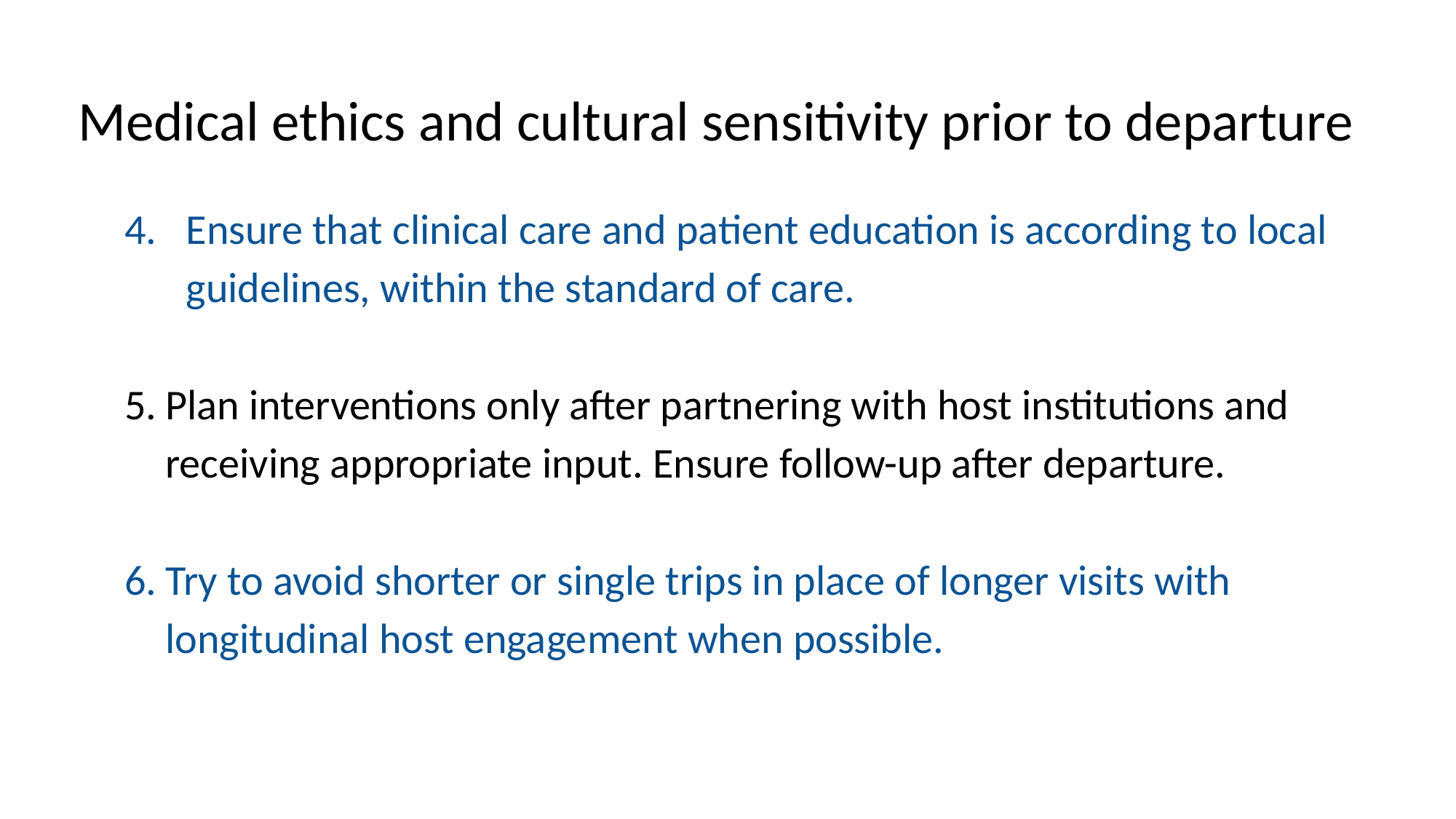

# Medical ethics and cultural sensitivity prior to departure
Ensure that clinical care and patient education is according to local guidelines, within the standard of care.
Plan interventions only after partnering with host institutions and receiving appropriate input. Ensure follow-up after departure.
Try to avoid shorter or single trips in place of longer visits with longitudinal host engagement when possible.

## Slide 15
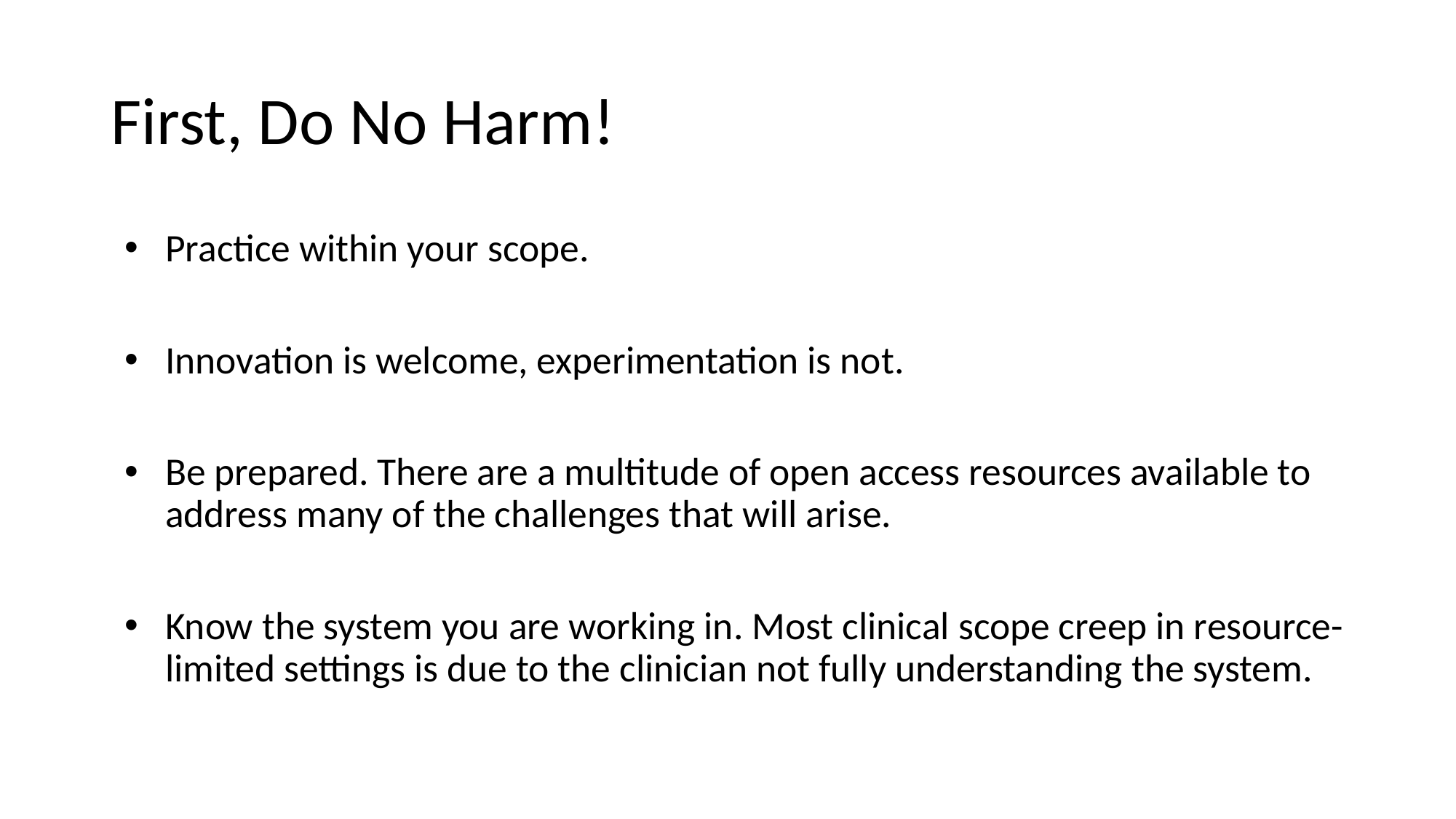

# First, Do No Harm!
Practice within your scope.
Innovation is welcome, experimentation is not.
Be prepared. There are a multitude of open access resources available to address many of the challenges that will arise.
Know the system you are working in. Most clinical scope creep in resource-limited settings is due to the clinician not fully understanding the system.

## Slide 16
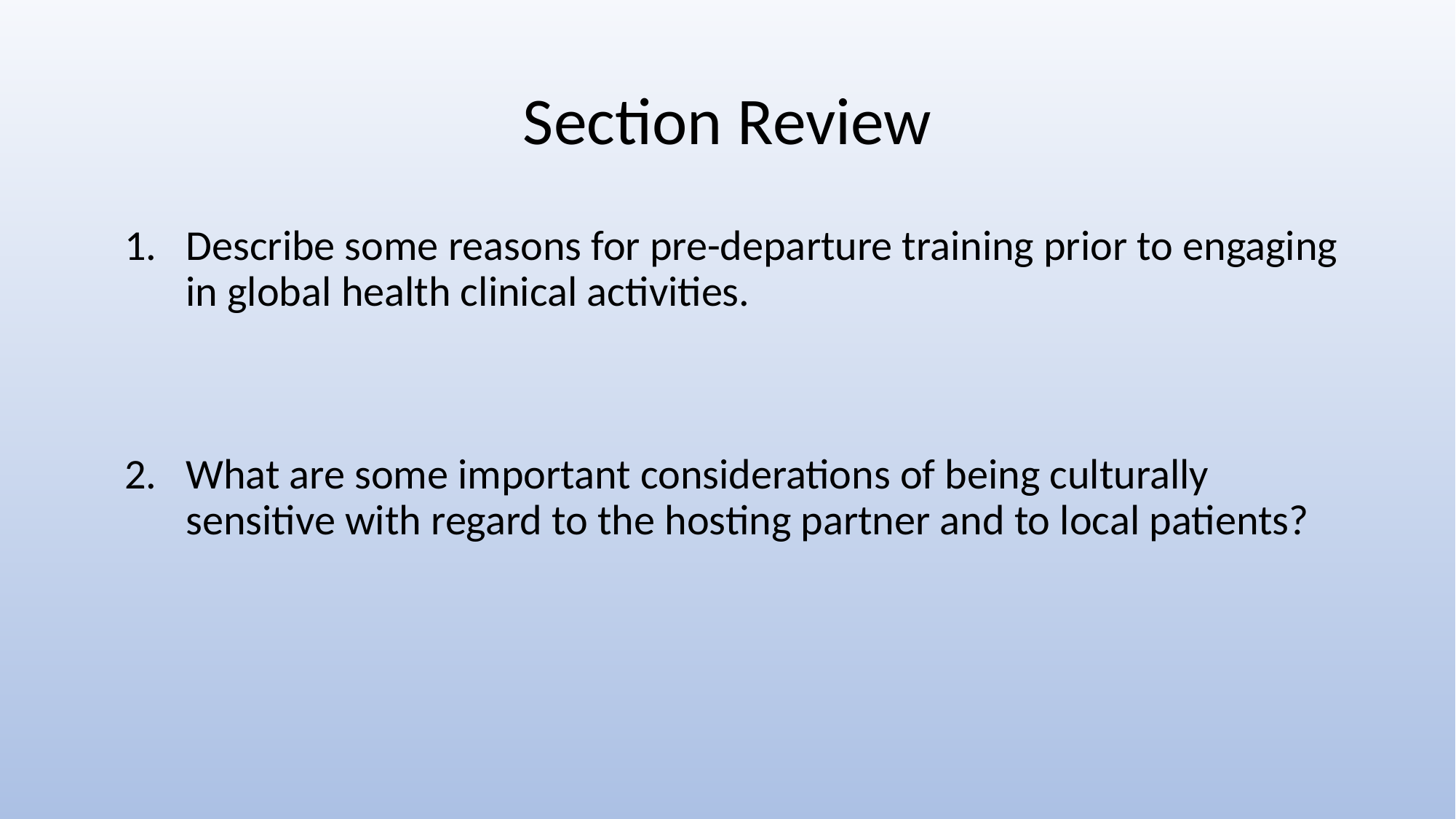

# Section Review
Describe some reasons for pre-departure training prior to engaging in global health clinical activities.
What are some important considerations of being culturally sensitive with regard to the hosting partner and to local patients?

## Slide 17
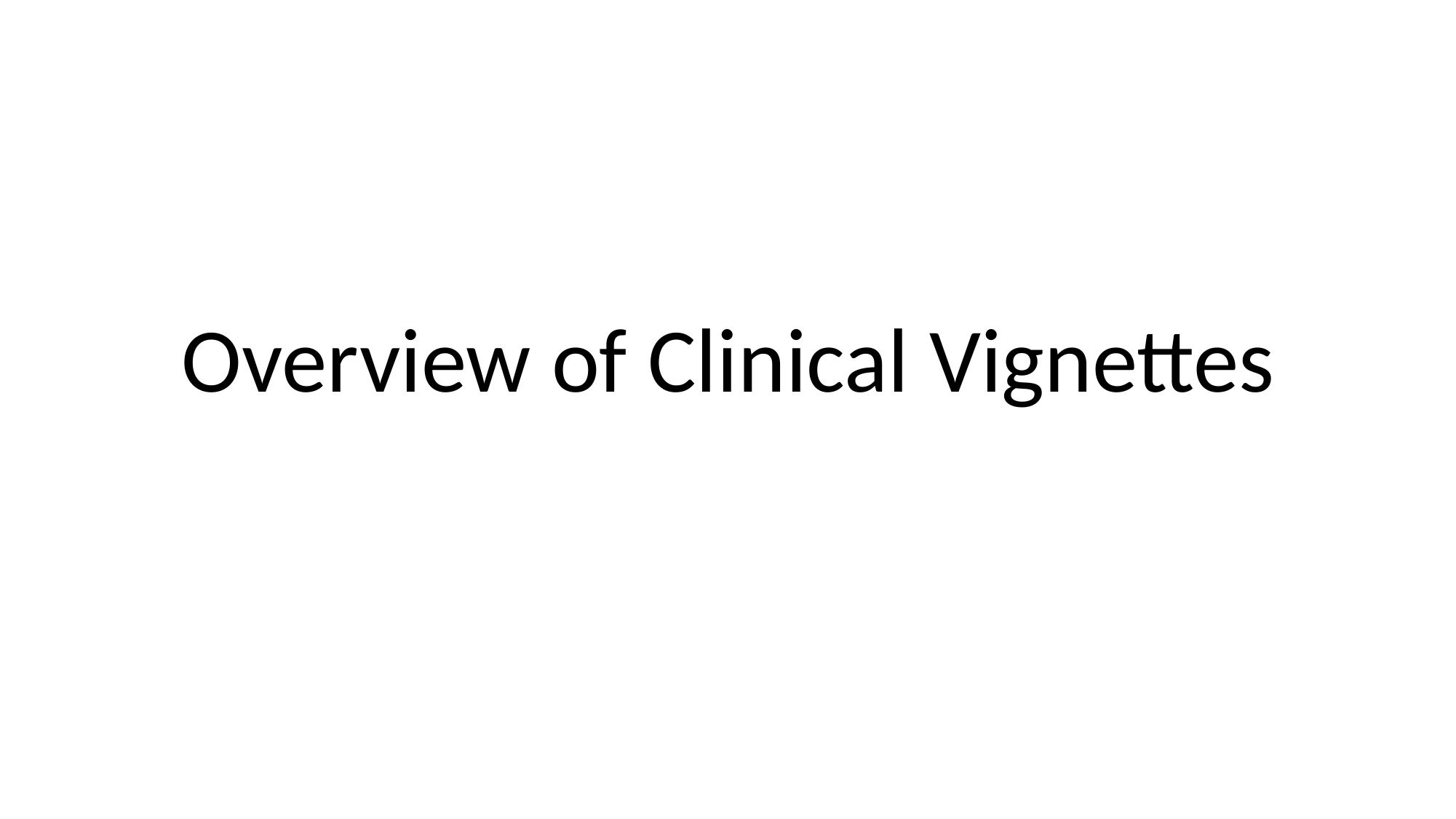

# Overview of Clinical Vignettes

## Slide 18
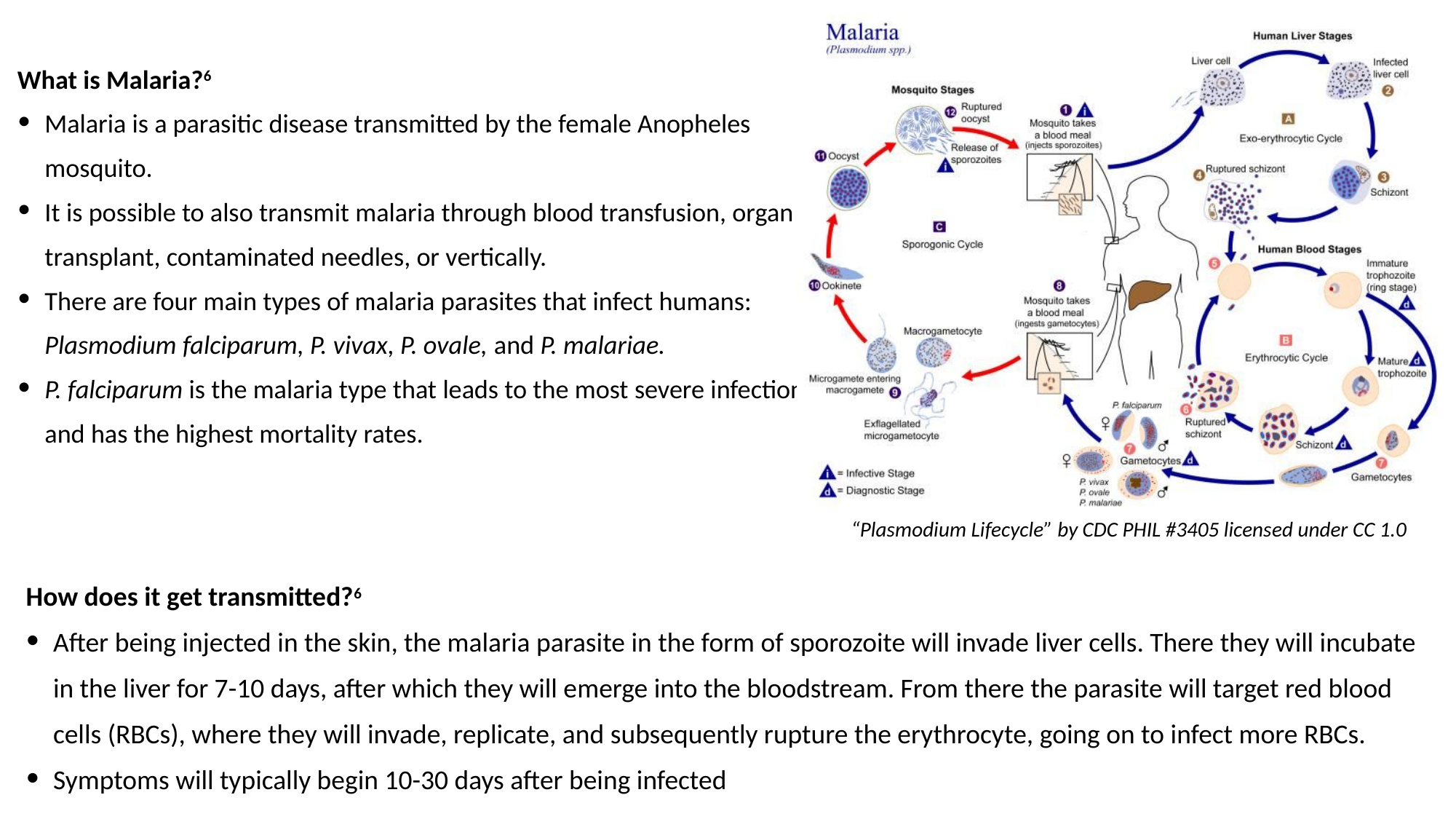

What is Malaria?6
Malaria is a parasitic disease transmitted by the female Anopheles mosquito.
It is possible to also transmit malaria through blood transfusion, organ transplant, contaminated needles, or vertically.
There are four main types of malaria parasites that infect humans:
Plasmodium falciparum, P. vivax, P. ovale, and P. malariae.
P. falciparum is the malaria type that leads to the most severe infections and has the highest mortality rates.
  “Plasmodium Lifecycle” by CDC PHIL #3405 licensed under CC 1.0
How does it get transmitted?6
After being injected in the skin, the malaria parasite in the form of sporozoite will invade liver cells. There they will incubate in the liver for 7-10 days, after which they will emerge into the bloodstream. From there the parasite will target red blood cells (RBCs), where they will invade, replicate, and subsequently rupture the erythrocyte, going on to infect more RBCs.
Symptoms will typically begin 10-30 days after being infected

## Slide 19
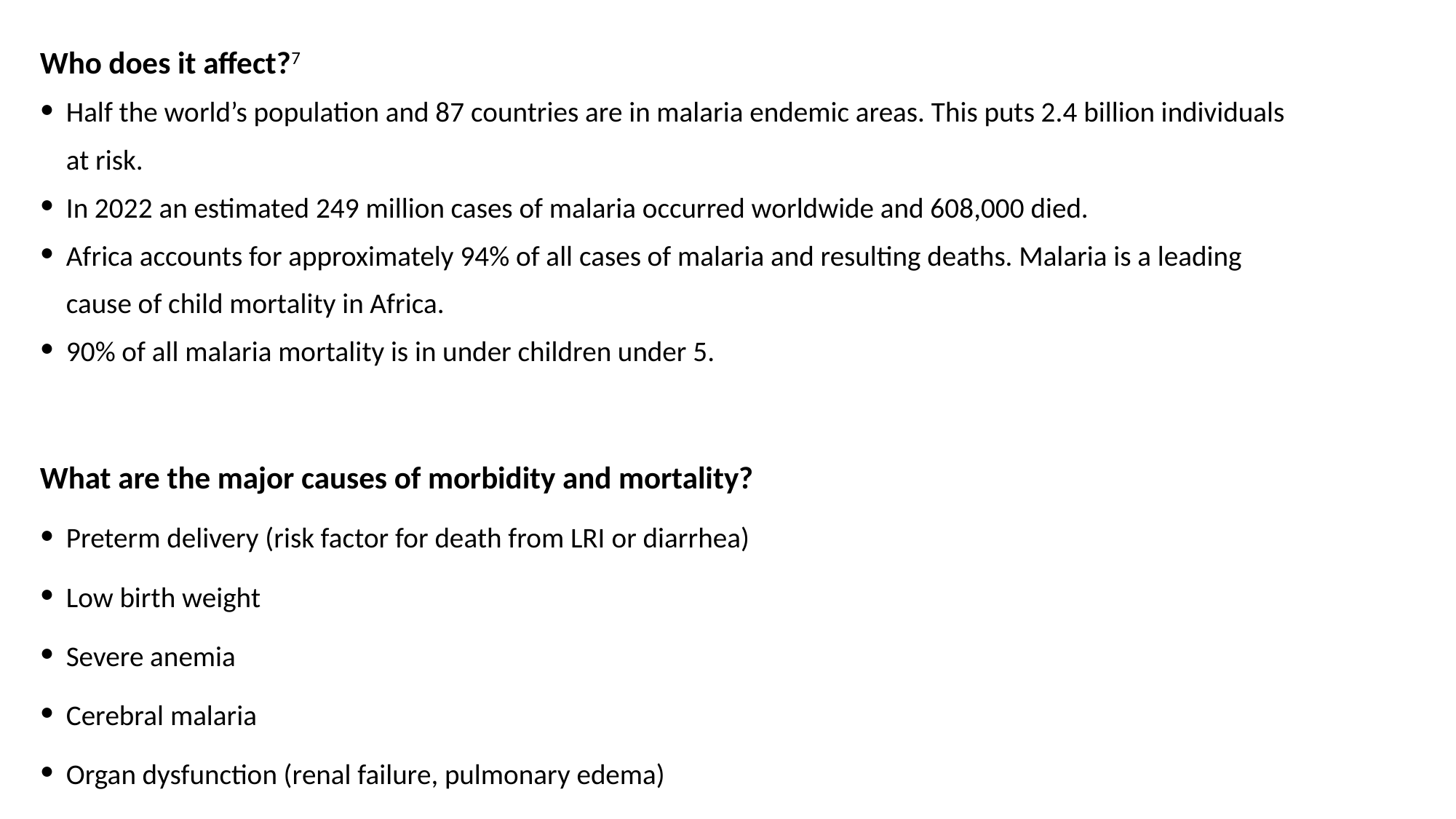

Who does it affect?7
Half the world’s population and 87 countries are in malaria endemic areas. This puts 2.4 billion individuals at risk.
In 2022 an estimated 249 million cases of malaria occurred worldwide and 608,000 died.
Africa accounts for approximately 94% of all cases of malaria and resulting deaths. Malaria is a leading cause of child mortality in Africa.
90% of all malaria mortality is in under children under 5.
What are the major causes of morbidity and mortality?
Preterm delivery (risk factor for death from LRI or diarrhea)
Low birth weight
Severe anemia
Cerebral malaria
Organ dysfunction (renal failure, pulmonary edema)

## Slide 20
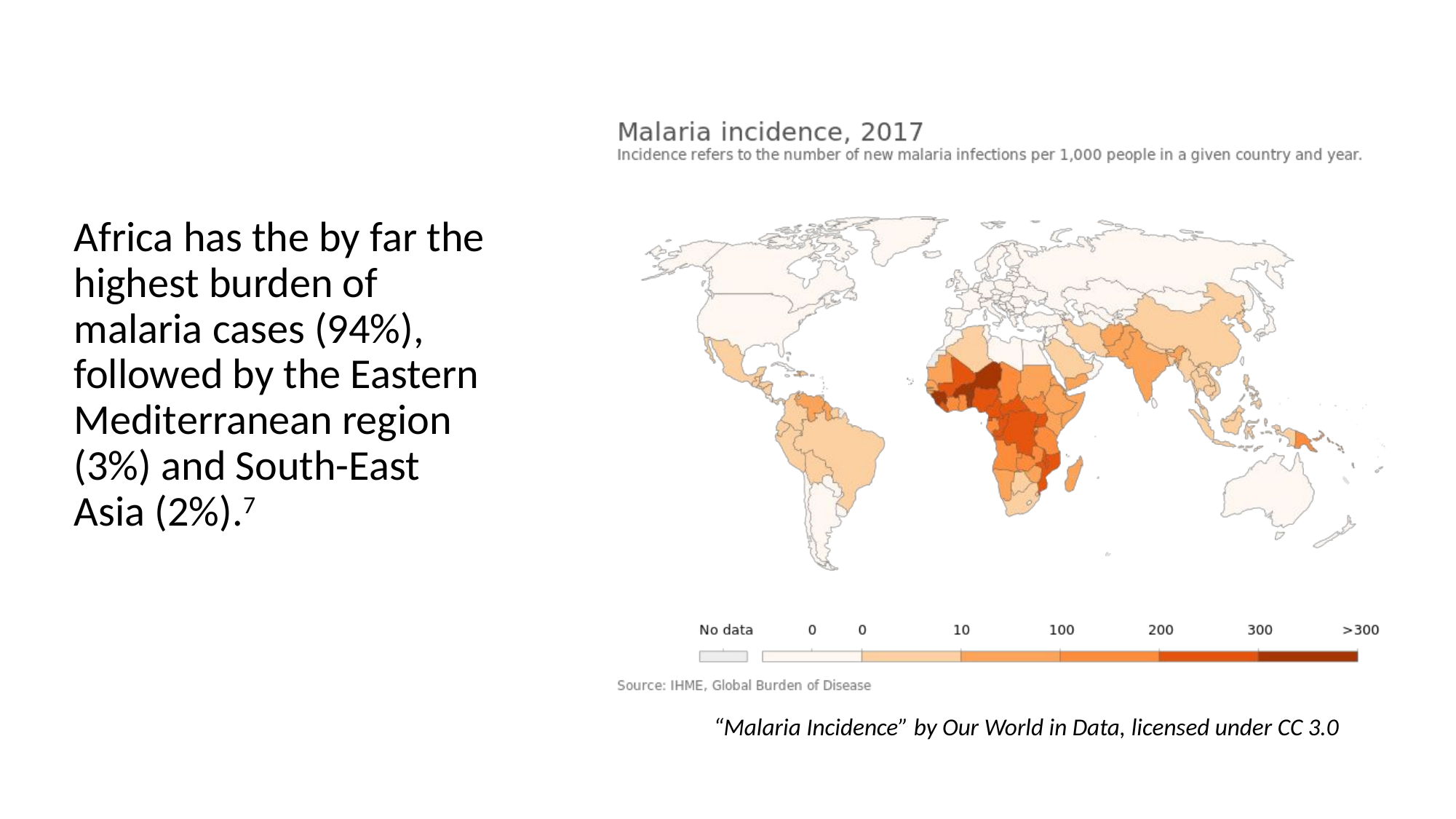

Africa has the by far the highest burden of malaria cases (94%), followed by the Eastern Mediterranean region (3%) and South-East Asia (2%).7
“Malaria Incidence” by Our World in Data, licensed under CC 3.0

## Slide 21
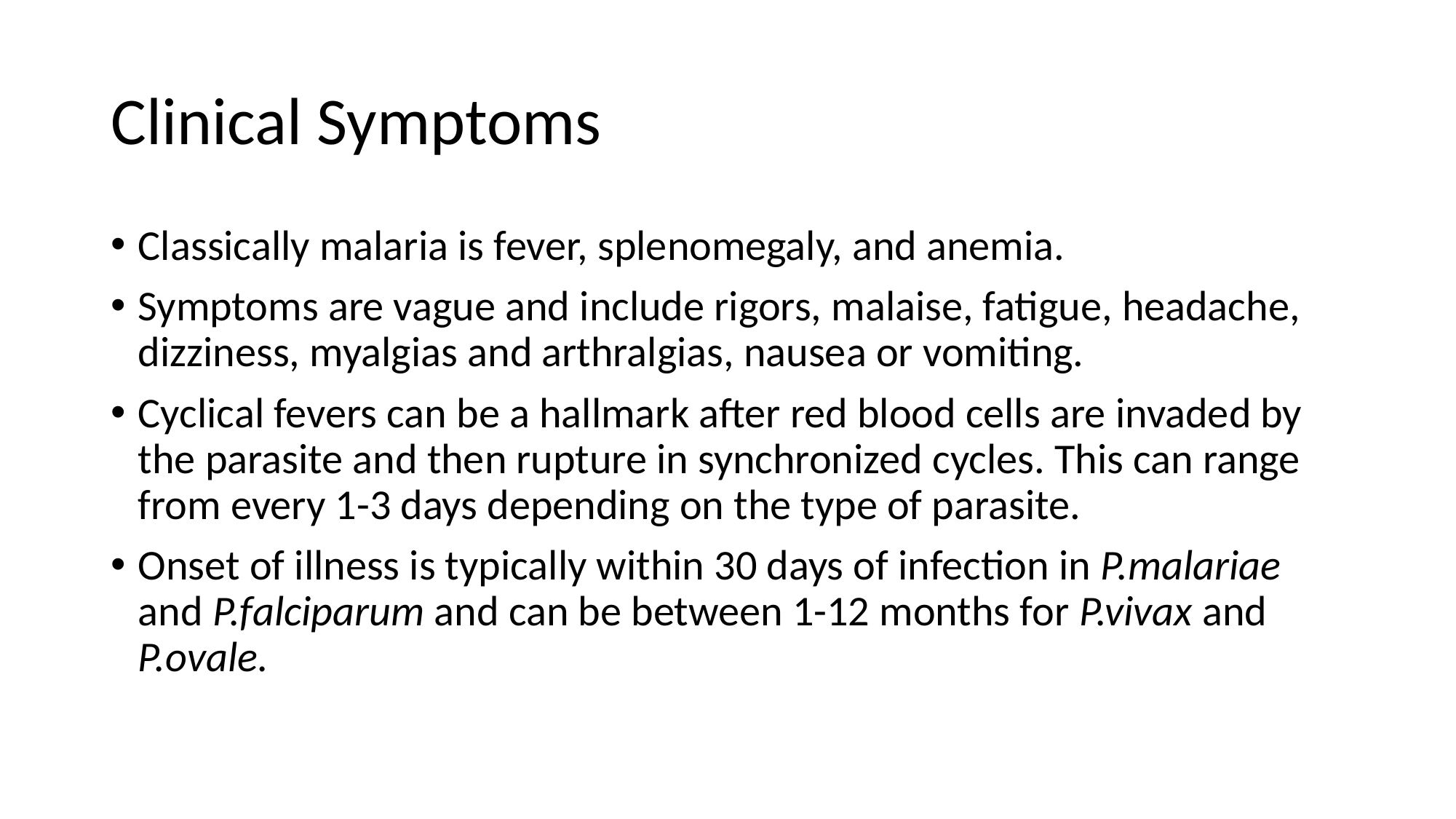

# Clinical Symptoms
Classically malaria is fever, splenomegaly, and anemia.
Symptoms are vague and include rigors, malaise, fatigue, headache, dizziness, myalgias and arthralgias, nausea or vomiting.
Cyclical fevers can be a hallmark after red blood cells are invaded by the parasite and then rupture in synchronized cycles. This can range from every 1-3 days depending on the type of parasite.
Onset of illness is typically within 30 days of infection in P.malariae and P.falciparum and can be between 1-12 months for P.vivax and P.ovale.

## Slide 22
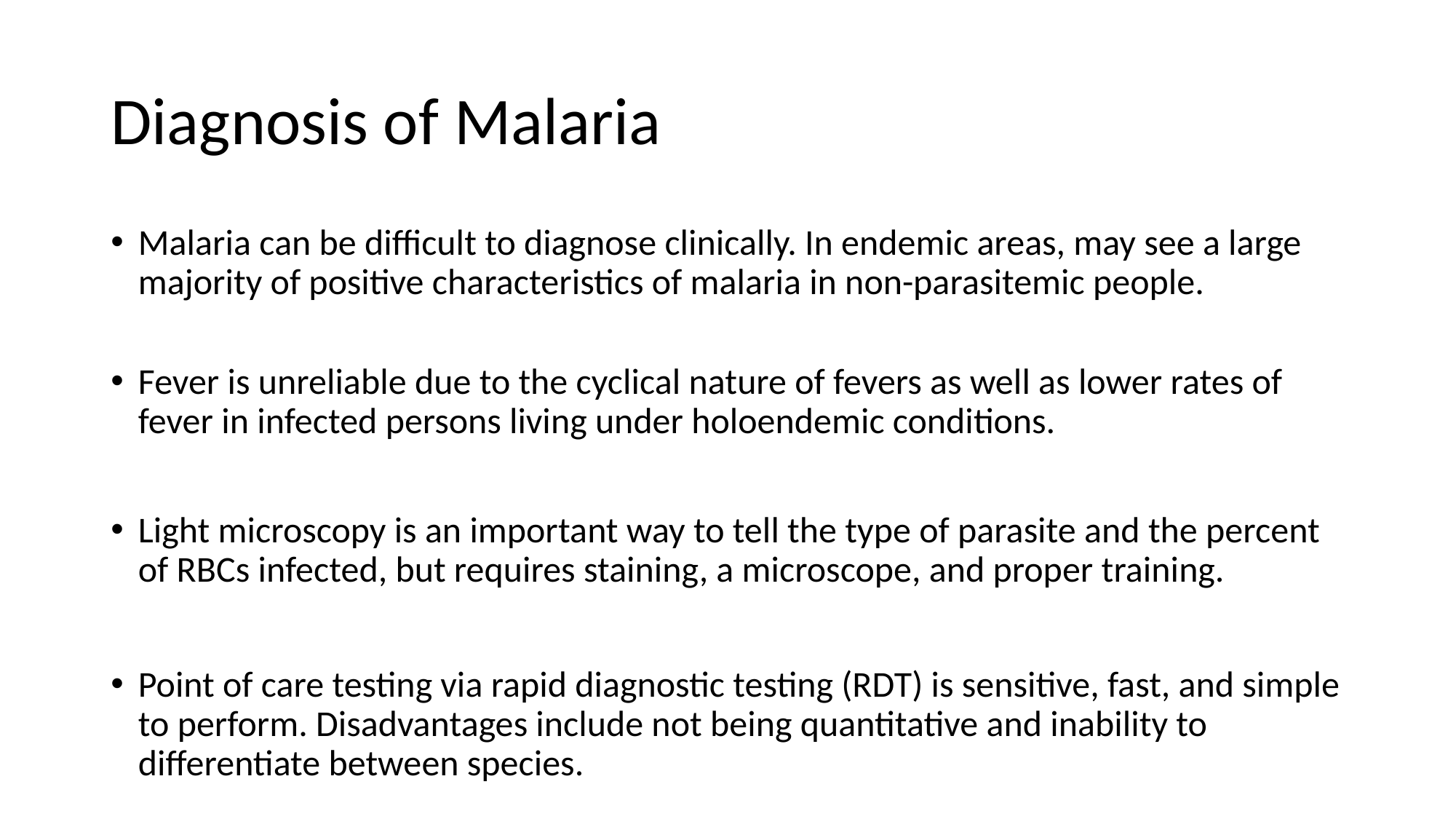

# Diagnosis of Malaria
Malaria can be difficult to diagnose clinically. In endemic areas, may see a large majority of positive characteristics of malaria in non-parasitemic people.
Fever is unreliable due to the cyclical nature of fevers as well as lower rates of fever in infected persons living under holoendemic conditions.
Light microscopy is an important way to tell the type of parasite and the percent of RBCs infected, but requires staining, a microscope, and proper training.
Point of care testing via rapid diagnostic testing (RDT) is sensitive, fast, and simple to perform. Disadvantages include not being quantitative and inability to differentiate between species.

## Slide 23
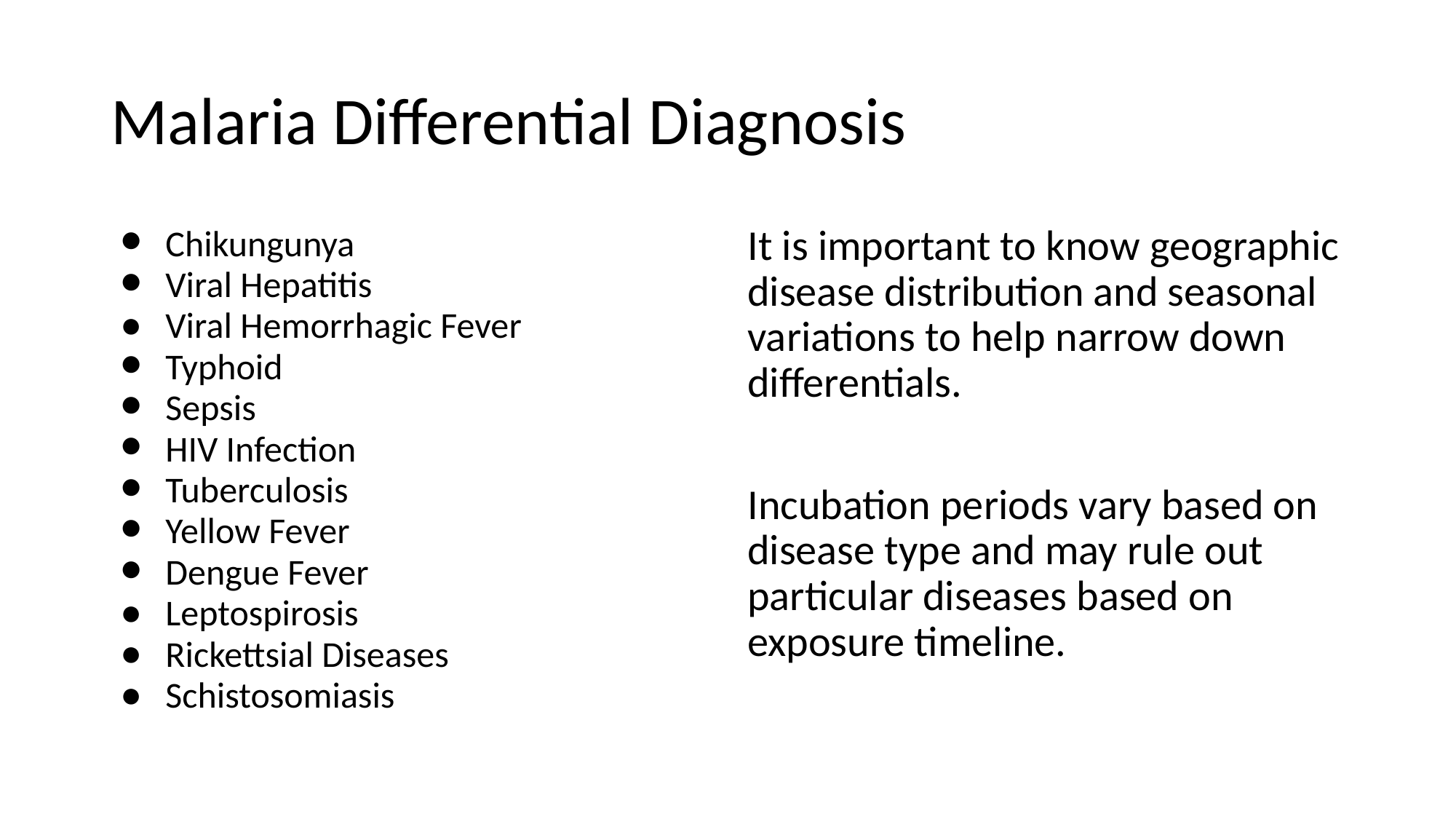

# Malaria Differential Diagnosis
Chikungunya
Viral Hepatitis
Viral Hemorrhagic Fever
Typhoid
Sepsis
HIV Infection
Tuberculosis
Yellow Fever
Dengue Fever
Leptospirosis
Rickettsial Diseases
Schistosomiasis
It is important to know geographic disease distribution and seasonal variations to help narrow down differentials.
Incubation periods vary based on disease type and may rule out particular diseases based on exposure timeline.

## Slide 24
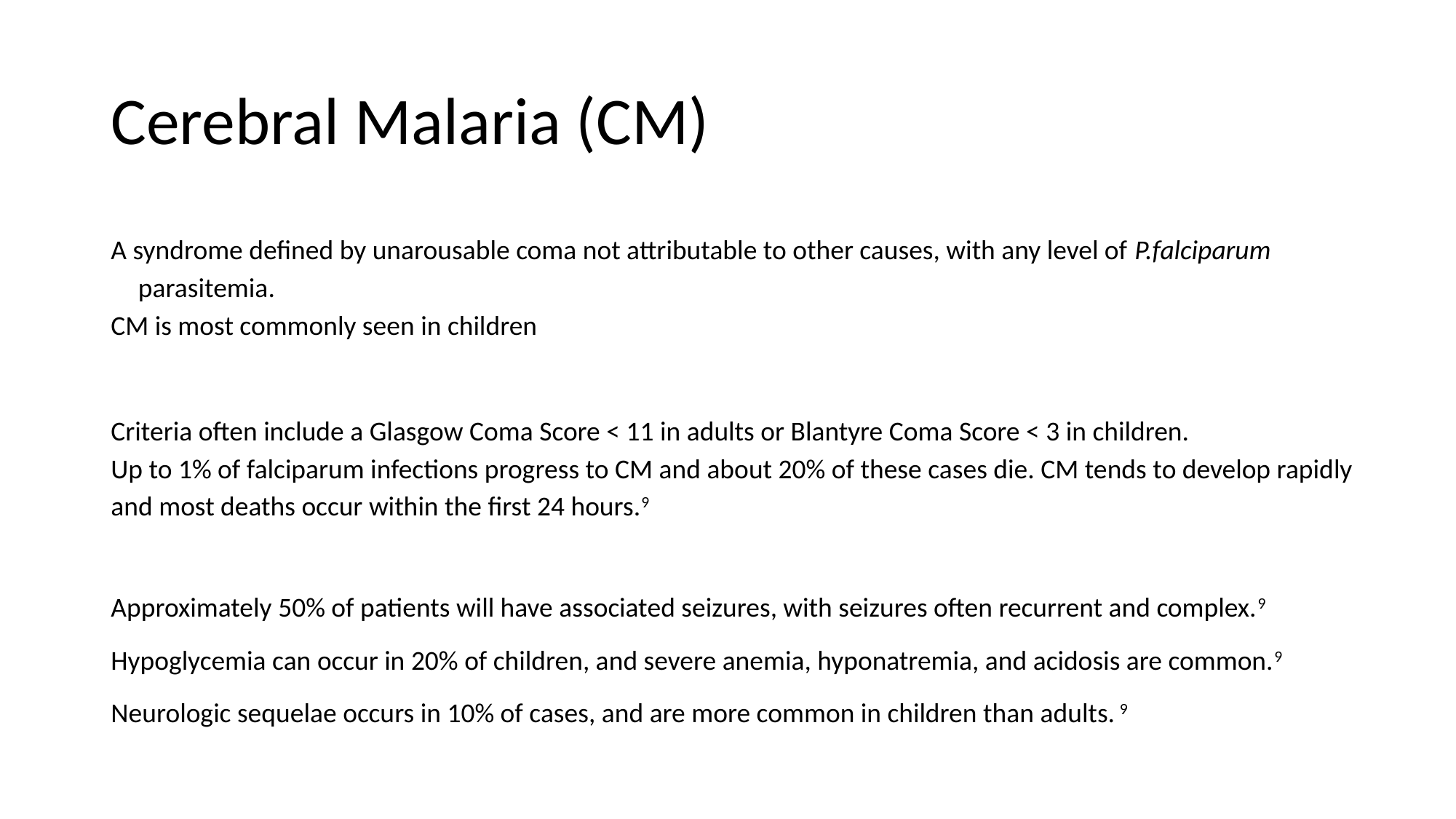

# Cerebral Malaria (CM)
A syndrome defined by unarousable coma not attributable to other causes, with any level of P.falciparum parasitemia.
CM is most commonly seen in children
Criteria often include a Glasgow Coma Score < 11 in adults or Blantyre Coma Score < 3 in children.
Up to 1% of falciparum infections progress to CM and about 20% of these cases die. CM tends to develop rapidly and most deaths occur within the first 24 hours.9
Approximately 50% of patients will have associated seizures, with seizures often recurrent and complex.9
Hypoglycemia can occur in 20% of children, and severe anemia, hyponatremia, and acidosis are common.9
Neurologic sequelae occurs in 10% of cases, and are more common in children than adults. 9

## Slide 25
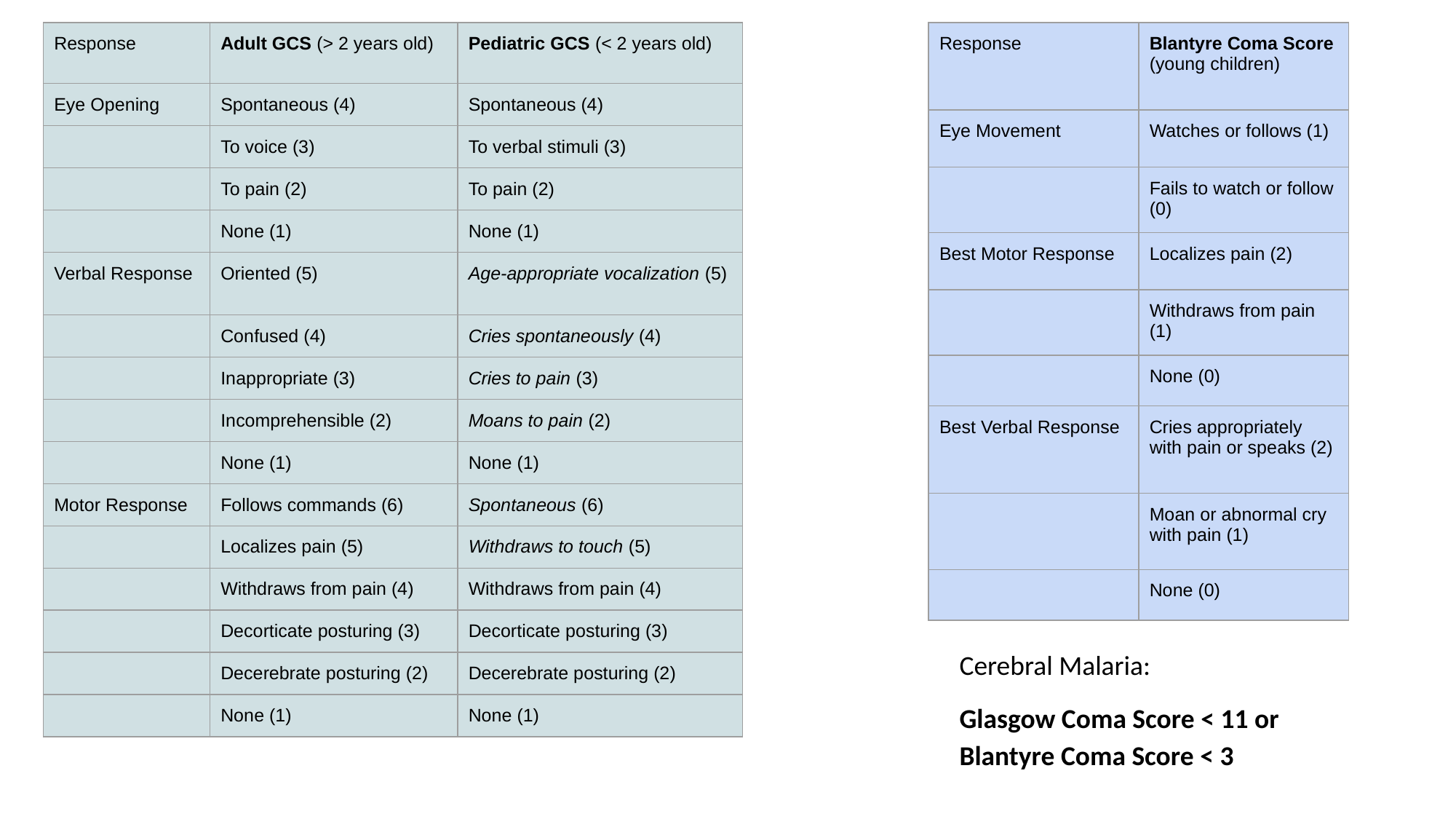

| Response | Blantyre Coma Score (young children) |
| --- | --- |
| Eye Movement | Watches or follows (1) |
| | Fails to watch or follow (0) |
| Best Motor Response | Localizes pain (2) |
| | Withdraws from pain (1) |
| | None (0) |
| Best Verbal Response | Cries appropriately with pain or speaks (2) |
| | Moan or abnormal cry with pain (1) |
| | None (0) |
| Response | Adult GCS (> 2 years old) | Pediatric GCS (< 2 years old) |
| --- | --- | --- |
| Eye Opening | Spontaneous (4) | Spontaneous (4) |
| | To voice (3) | To verbal stimuli (3) |
| | To pain (2) | To pain (2) |
| | None (1) | None (1) |
| Verbal Response | Oriented (5) | Age-appropriate vocalization (5) |
| | Confused (4) | Cries spontaneously (4) |
| | Inappropriate (3) | Cries to pain (3) |
| | Incomprehensible (2) | Moans to pain (2) |
| | None (1) | None (1) |
| Motor Response | Follows commands (6) | Spontaneous (6) |
| | Localizes pain (5) | Withdraws to touch (5) |
| | Withdraws from pain (4) | Withdraws from pain (4) |
| | Decorticate posturing (3) | Decorticate posturing (3) |
| | Decerebrate posturing (2) | Decerebrate posturing (2) |
| | None (1) | None (1) |
Cerebral Malaria:
Glasgow Coma Score < 11 or Blantyre Coma Score < 3

## Slide 26
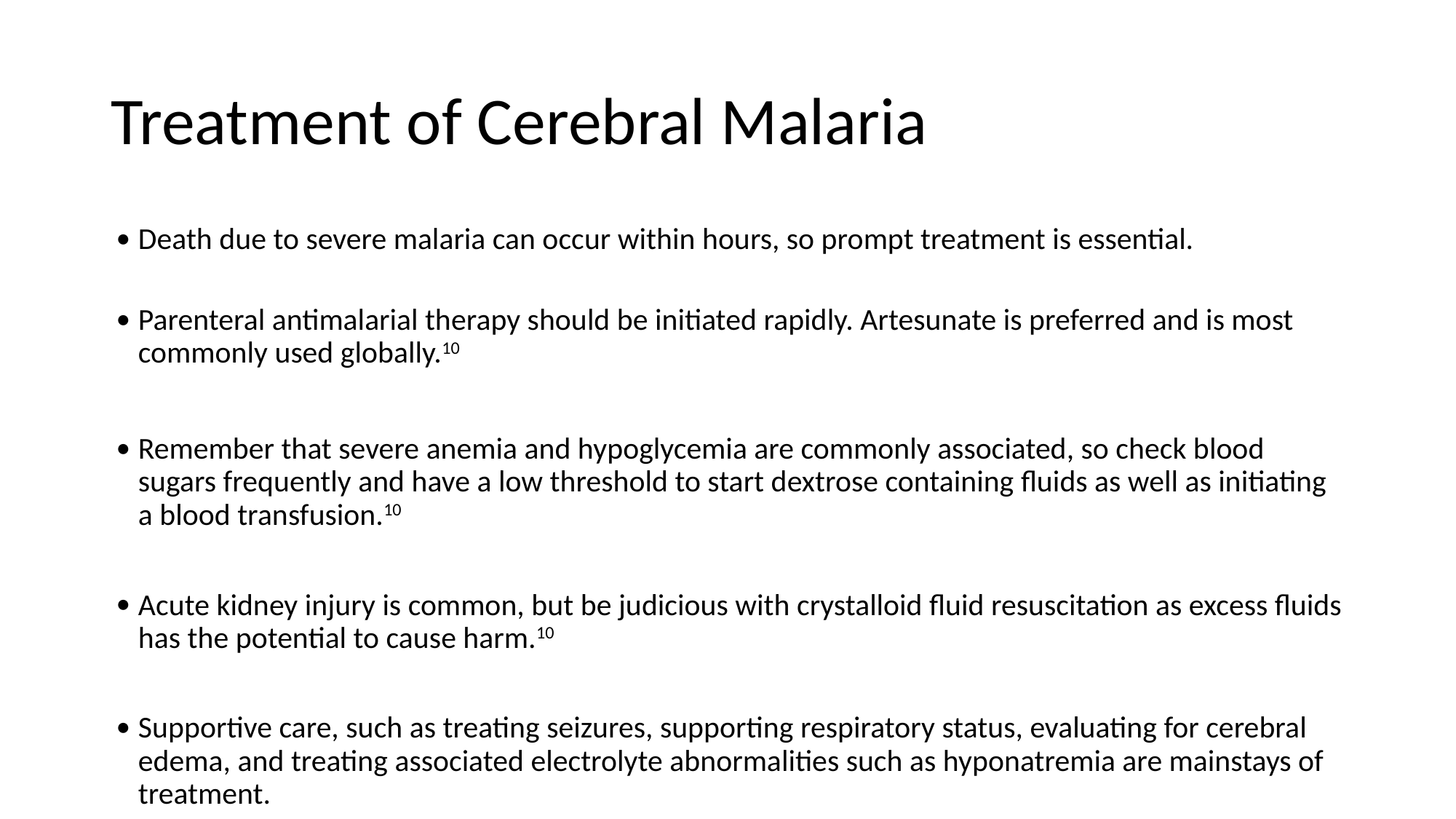

# Treatment of Cerebral Malaria
Death due to severe malaria can occur within hours, so prompt treatment is essential.
Parenteral antimalarial therapy should be initiated rapidly. Artesunate is preferred and is most commonly used globally.10
Remember that severe anemia and hypoglycemia are commonly associated, so check blood sugars frequently and have a low threshold to start dextrose containing fluids as well as initiating a blood transfusion.10
Acute kidney injury is common, but be judicious with crystalloid fluid resuscitation as excess fluids has the potential to cause harm.10
Supportive care, such as treating seizures, supporting respiratory status, evaluating for cerebral edema, and treating associated electrolyte abnormalities such as hyponatremia are mainstays of treatment.

## Slide 27
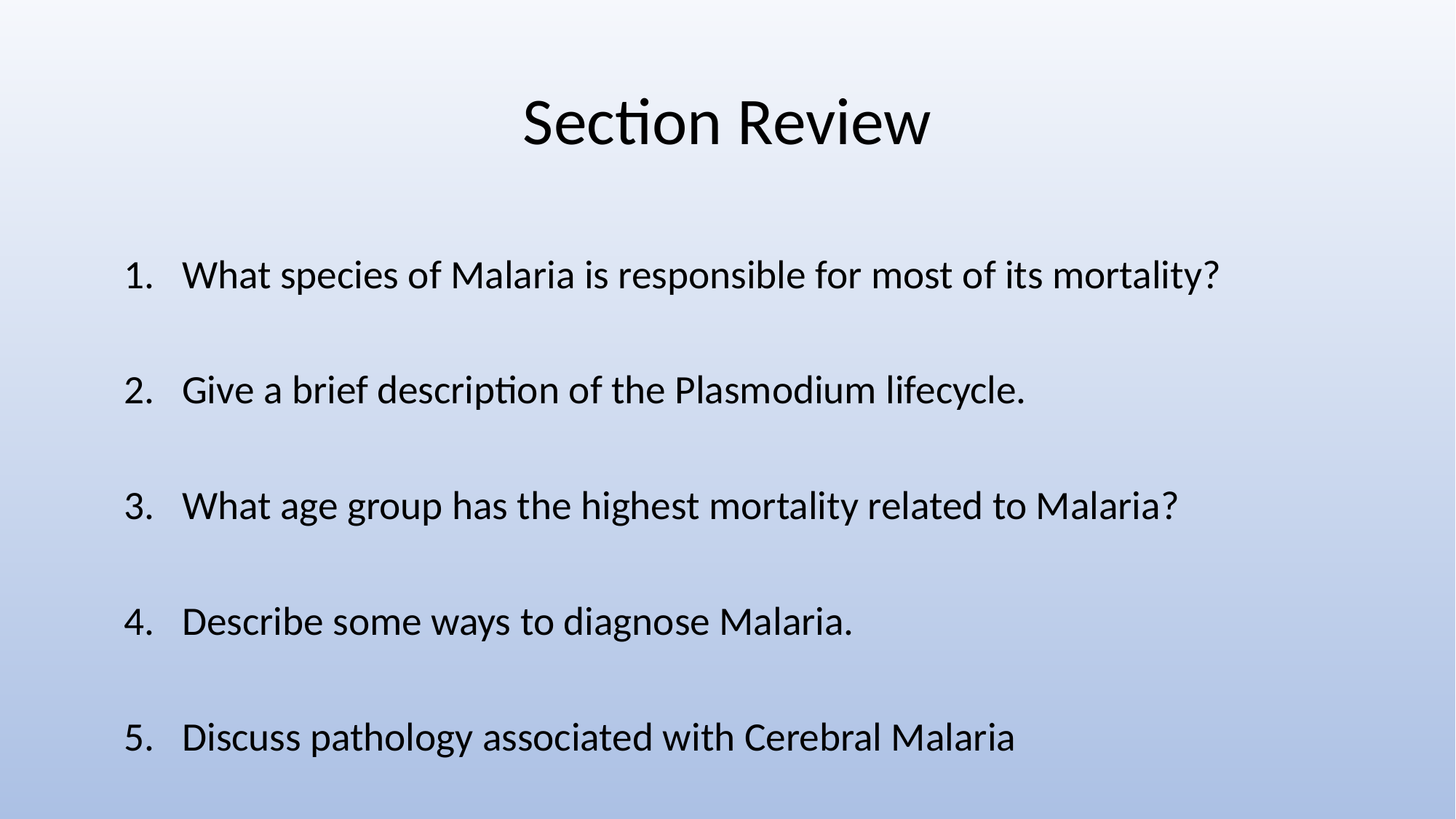

# Section Review
What species of Malaria is responsible for most of its mortality?
Give a brief description of the Plasmodium lifecycle.
What age group has the highest mortality related to Malaria?
Describe some ways to diagnose Malaria.
Discuss pathology associated with Cerebral Malaria

## Slide 28
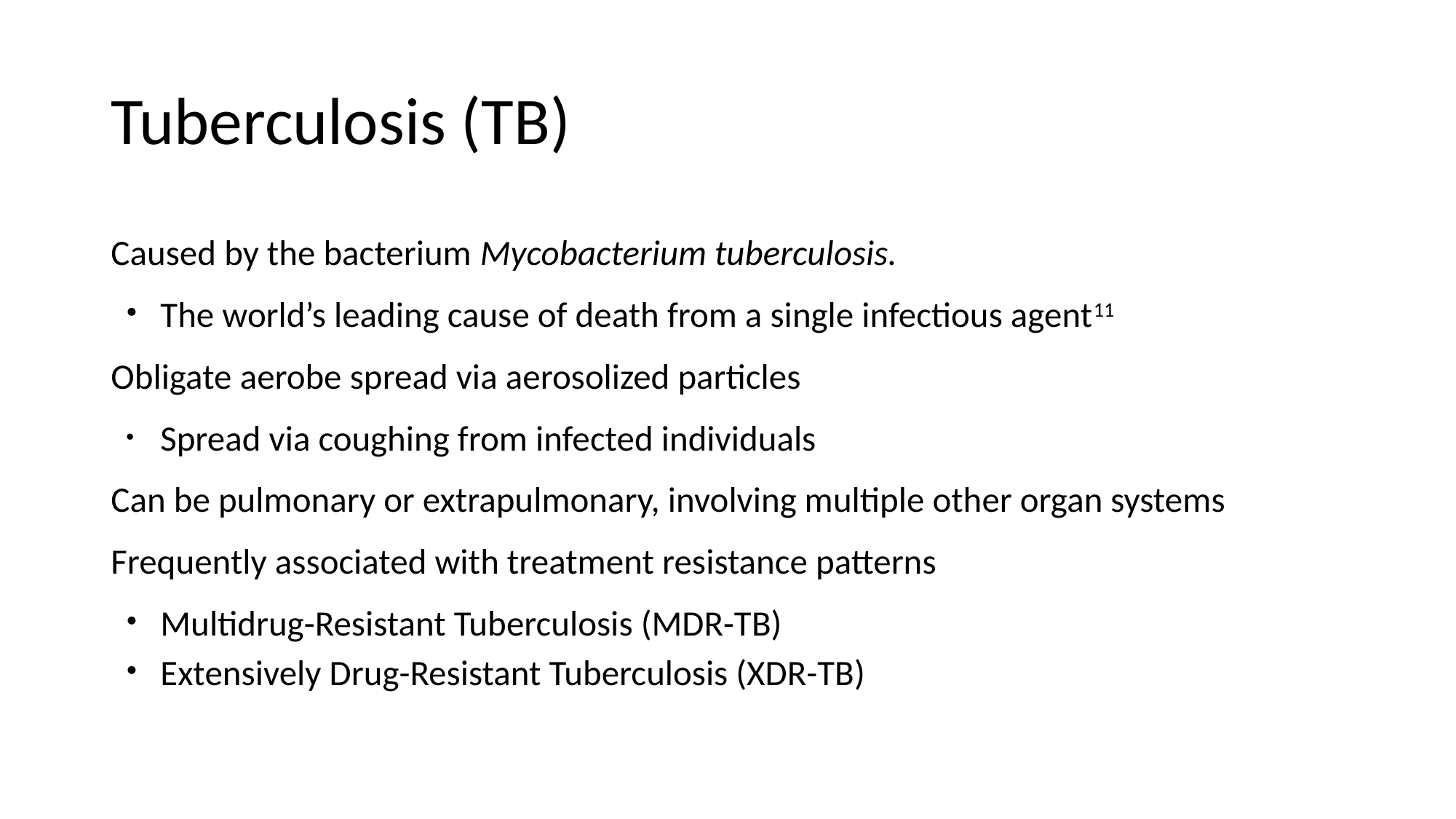

# Tuberculosis (TB)
Caused by the bacterium Mycobacterium tuberculosis.
The world’s leading cause of death from a single infectious agent11
Obligate aerobe spread via aerosolized particles
Spread via coughing from infected individuals
Can be pulmonary or extrapulmonary, involving multiple other organ systems
Frequently associated with treatment resistance patterns
Multidrug-Resistant Tuberculosis (MDR-TB)
Extensively Drug-Resistant Tuberculosis (XDR-TB)

## Slide 29
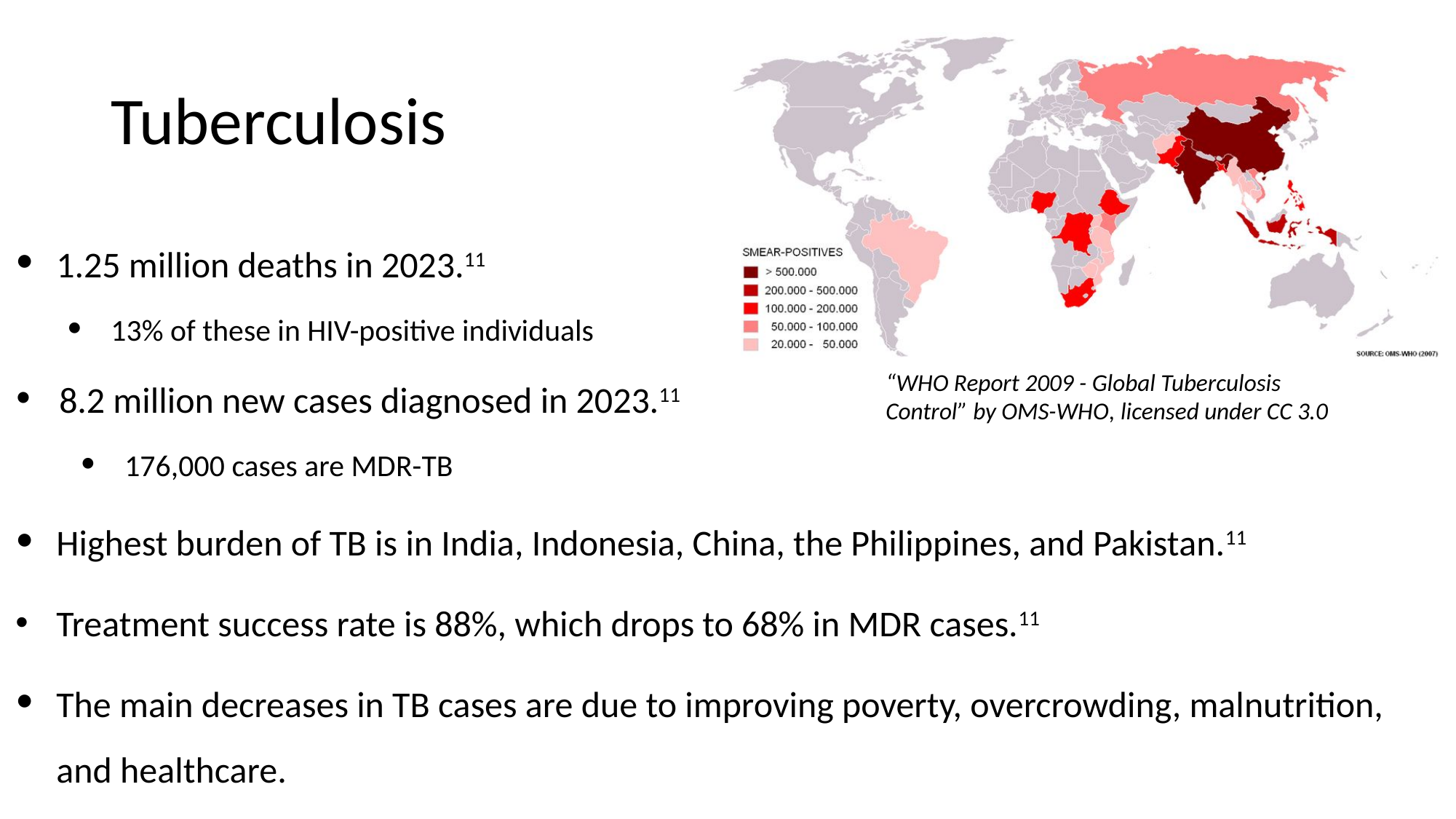

# Tuberculosis
1.25 million deaths in 2023.11
13% of these in HIV-positive individuals
 8.2 million new cases diagnosed in 2023.11
176,000 cases are MDR-TB
Highest burden of TB is in India, Indonesia, China, the Philippines, and Pakistan.11
Treatment success rate is 88%, which drops to 68% in MDR cases.11
The main decreases in TB cases are due to improving poverty, overcrowding, malnutrition, and healthcare.
“WHO Report 2009 - Global Tuberculosis Control” by OMS-WHO, licensed under CC 3.0

## Slide 30
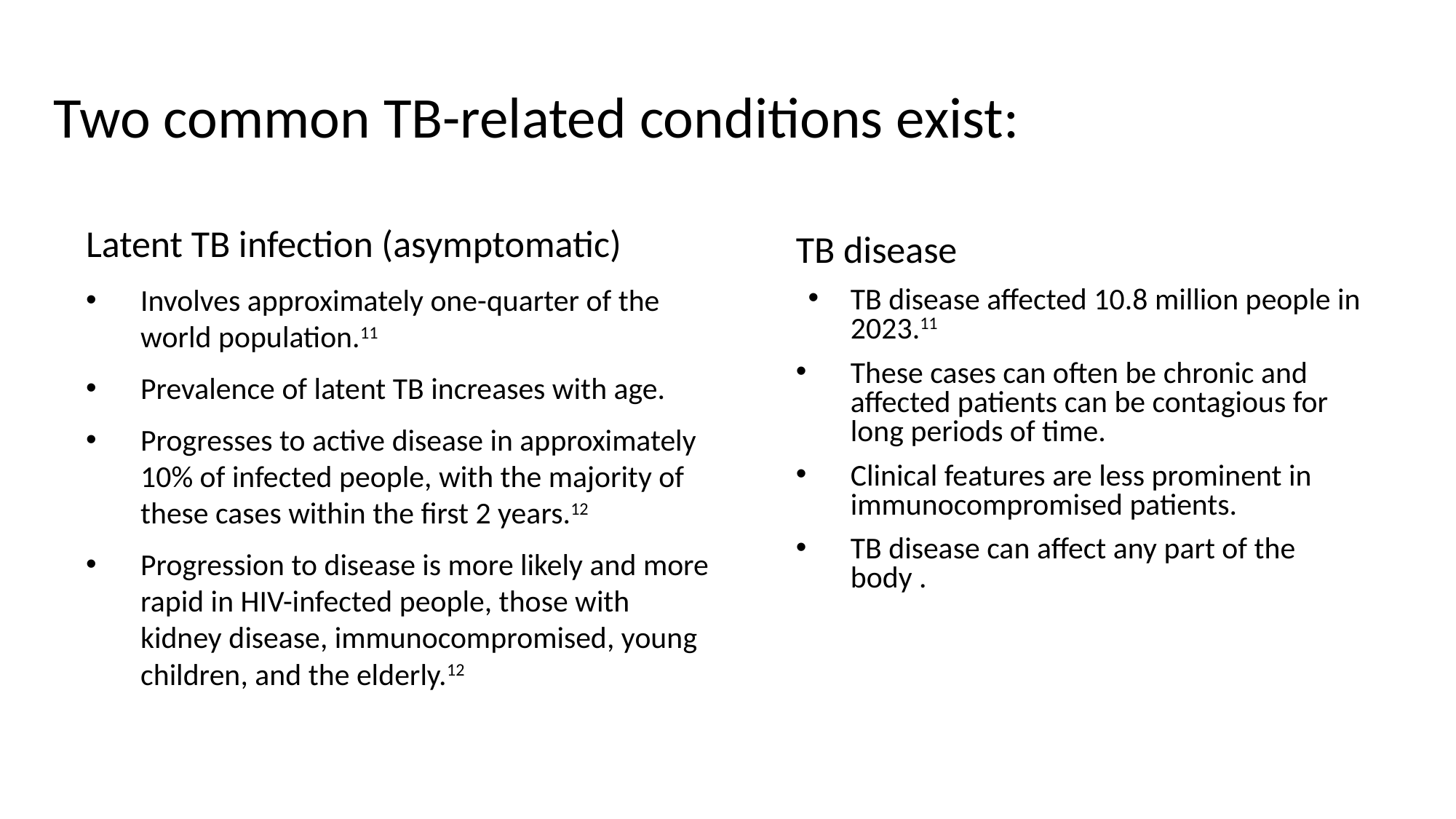

# Two common TB-related conditions exist:
Latent TB infection (asymptomatic)
Involves approximately one-quarter of the world population.11
Prevalence of latent TB increases with age.
Progresses to active disease in approximately 10% of infected people, with the majority of these cases within the first 2 years.12
Progression to disease is more likely and more rapid in HIV-infected people, those with kidney disease, immunocompromised, young children, and the elderly.12
TB disease
TB disease affected 10.8 million people in 2023.11
These cases can often be chronic and affected patients can be contagious for long periods of time.
Clinical features are less prominent in immunocompromised patients.
TB disease can affect any part of the body .

## Slide 31
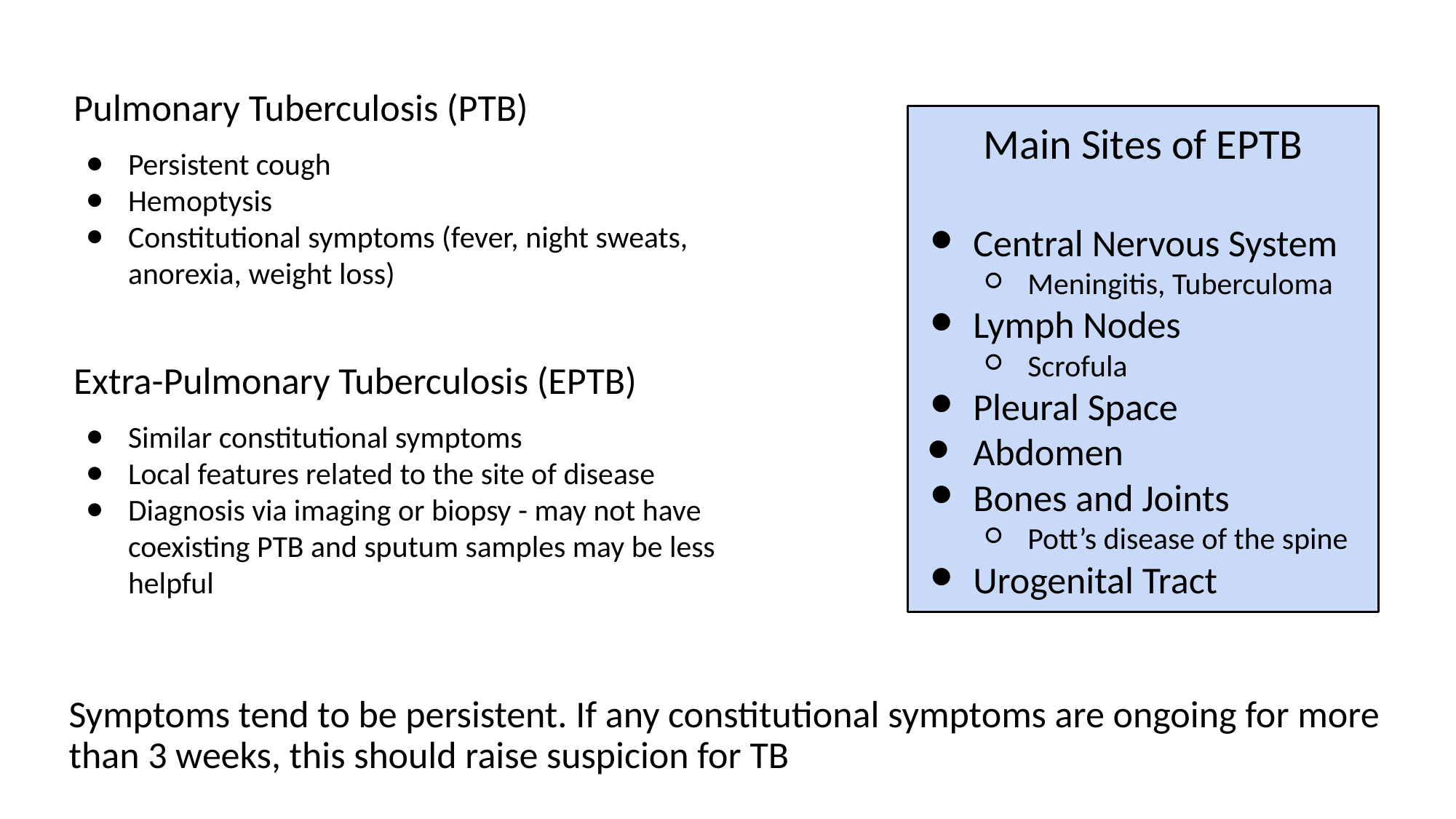

Pulmonary Tuberculosis (PTB)
Persistent cough
Hemoptysis
Constitutional symptoms (fever, night sweats, anorexia, weight loss)
Extra-Pulmonary Tuberculosis (EPTB)
Similar constitutional symptoms
Local features related to the site of disease
Diagnosis via imaging or biopsy - may not have coexisting PTB and sputum samples may be less helpful
Main Sites of EPTB
Central Nervous System
Meningitis, Tuberculoma
Lymph Nodes
Scrofula
Pleural Space
Abdomen
Bones and Joints
Pott’s disease of the spine
Urogenital Tract
Symptoms tend to be persistent. If any constitutional symptoms are ongoing for more than 3 weeks, this should raise suspicion for TB

## Slide 32
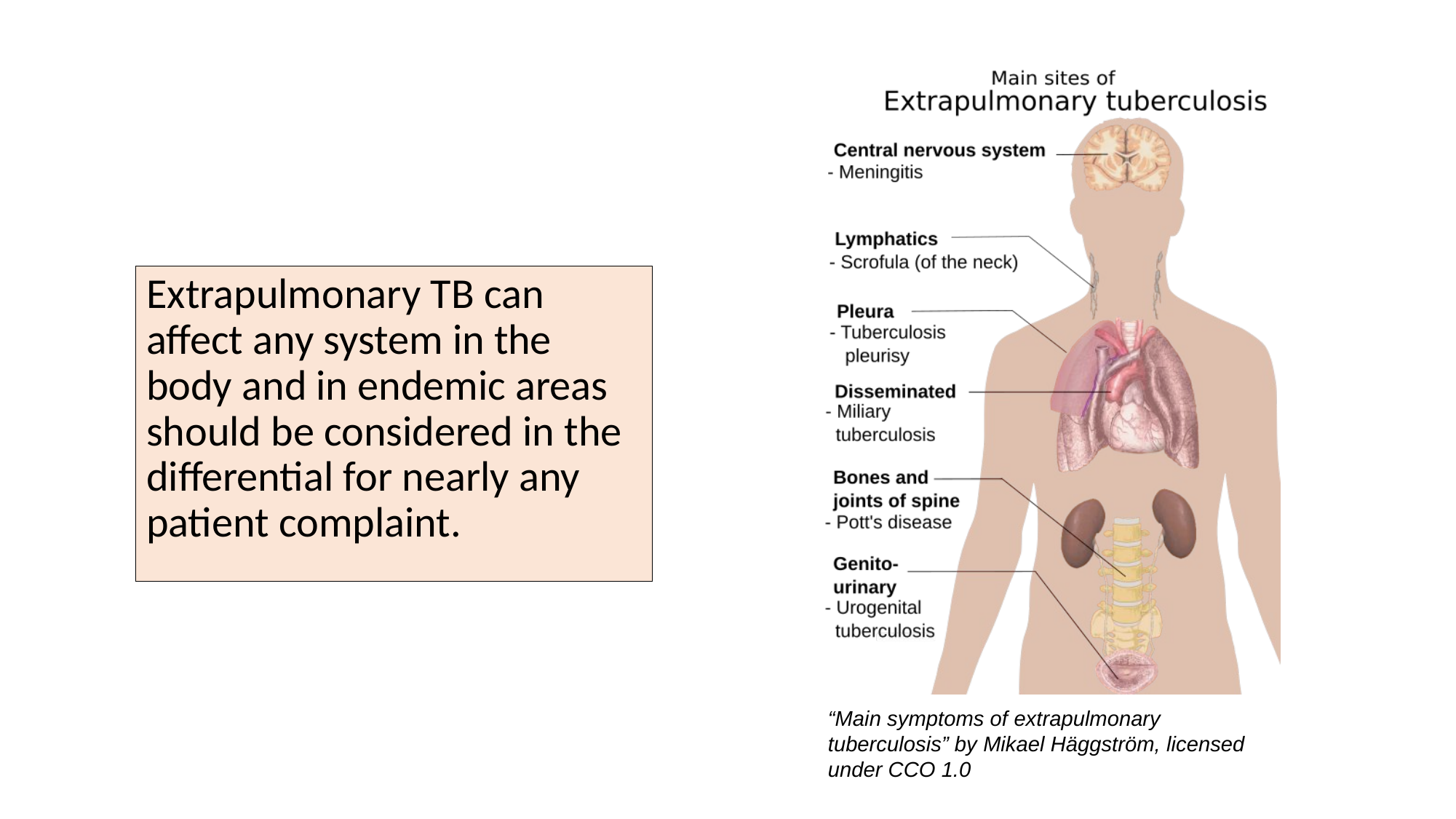

Extrapulmonary TB can affect any system in the body and in endemic areas should be considered in the differential for nearly any patient complaint.
“Main symptoms of extrapulmonary tuberculosis” by Mikael Häggström, licensed under CCO 1.0

## Slide 33
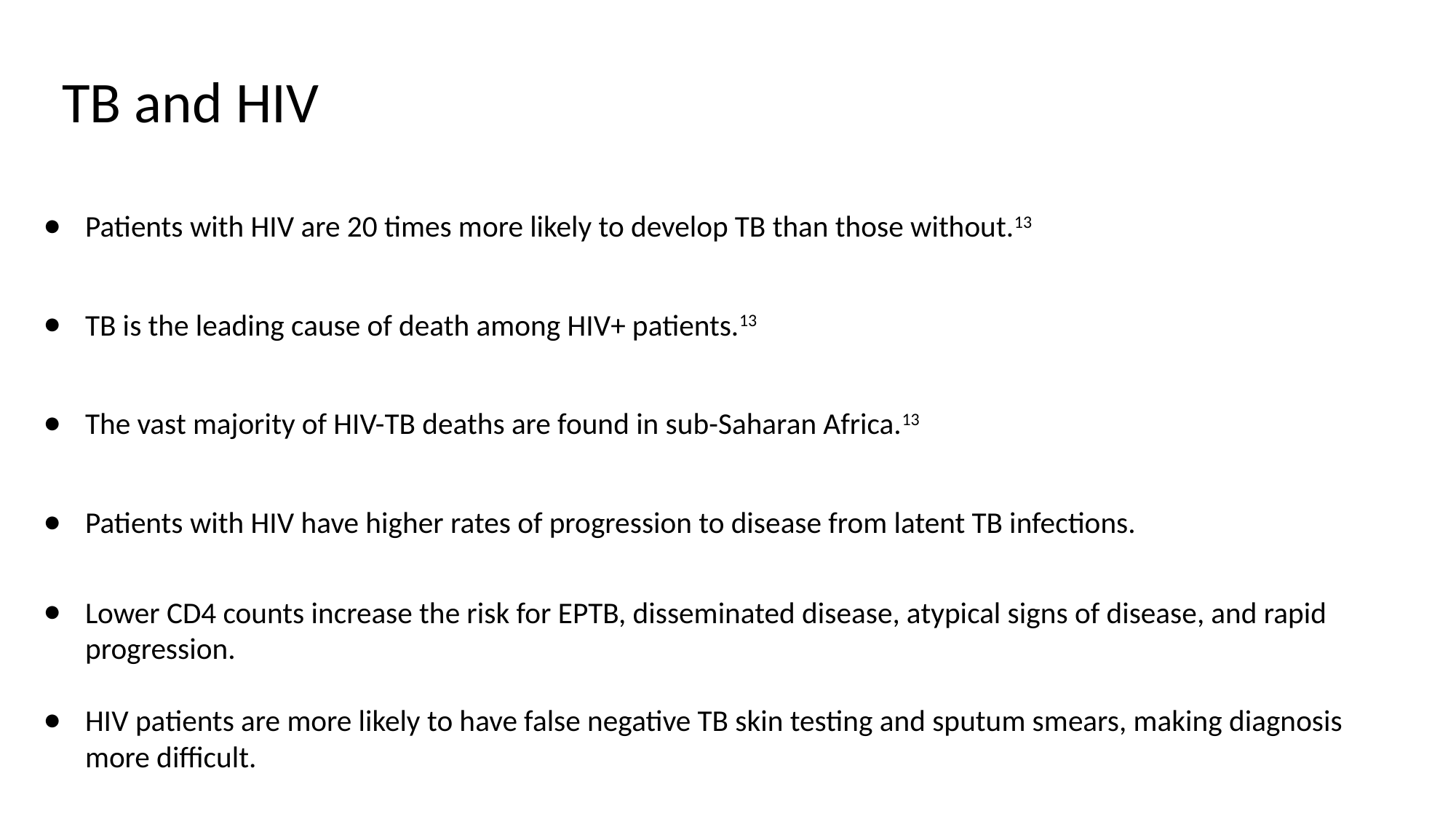

# TB and HIV
Patients with HIV are 20 times more likely to develop TB than those without.13
TB is the leading cause of death among HIV+ patients.13
The vast majority of HIV-TB deaths are found in sub-Saharan Africa.13
Patients with HIV have higher rates of progression to disease from latent TB infections.
Lower CD4 counts increase the risk for EPTB, disseminated disease, atypical signs of disease, and rapid progression.
HIV patients are more likely to have false negative TB skin testing and sputum smears, making diagnosis more difficult.

## Slide 34
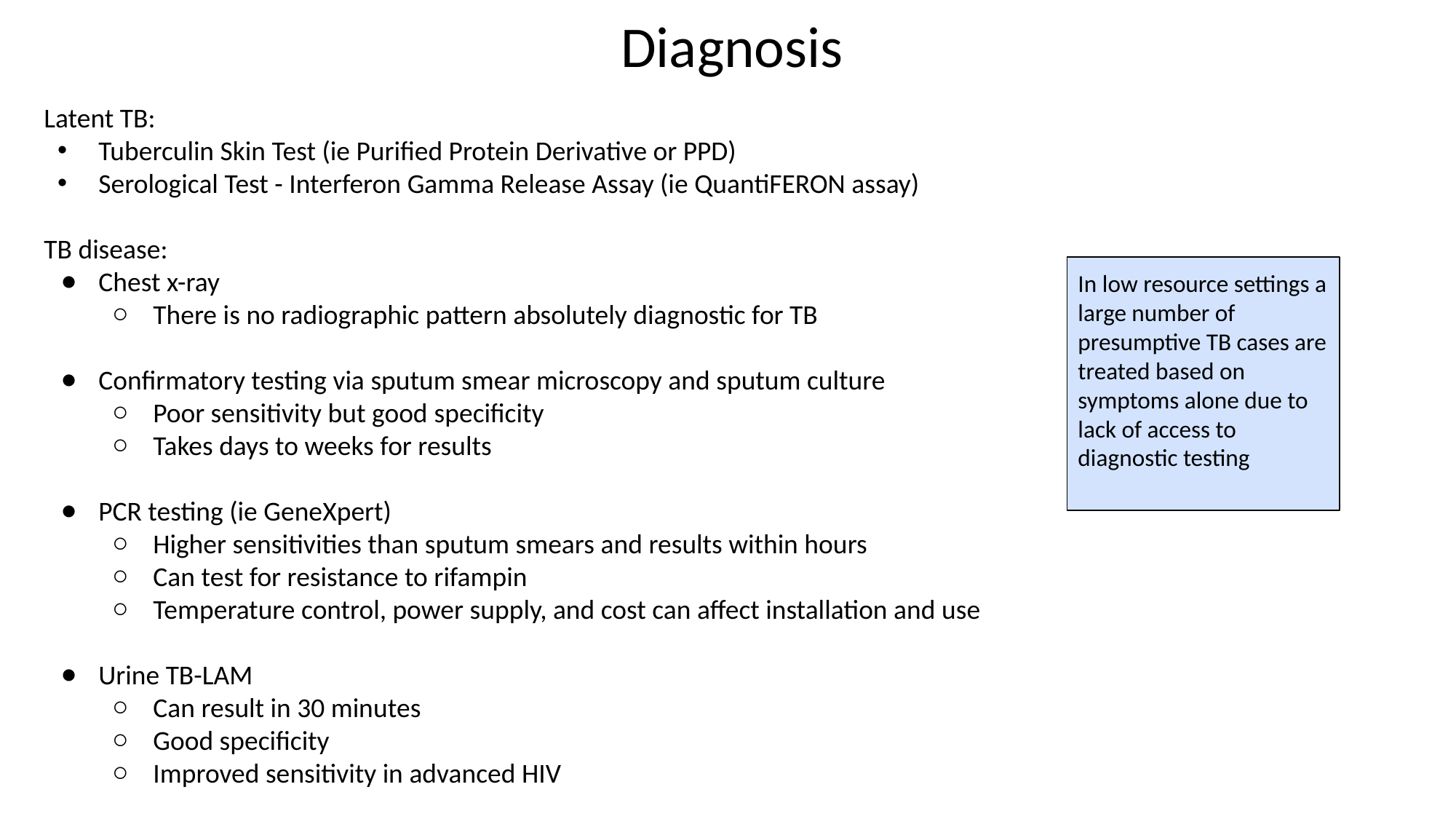

# Diagnosis
Latent TB:
Tuberculin Skin Test (ie Purified Protein Derivative or PPD)
Serological Test - Interferon Gamma Release Assay (ie QuantiFERON assay)
TB disease:
Chest x-ray
There is no radiographic pattern absolutely diagnostic for TB
Confirmatory testing via sputum smear microscopy and sputum culture
Poor sensitivity but good specificity
Takes days to weeks for results
PCR testing (ie GeneXpert)
Higher sensitivities than sputum smears and results within hours
Can test for resistance to rifampin
Temperature control, power supply, and cost can affect installation and use
Urine TB-LAM
Can result in 30 minutes
Good specificity
Improved sensitivity in advanced HIV
In low resource settings a large number of presumptive TB cases are treated based on symptoms alone due to lack of access to diagnostic testing

## Slide 35
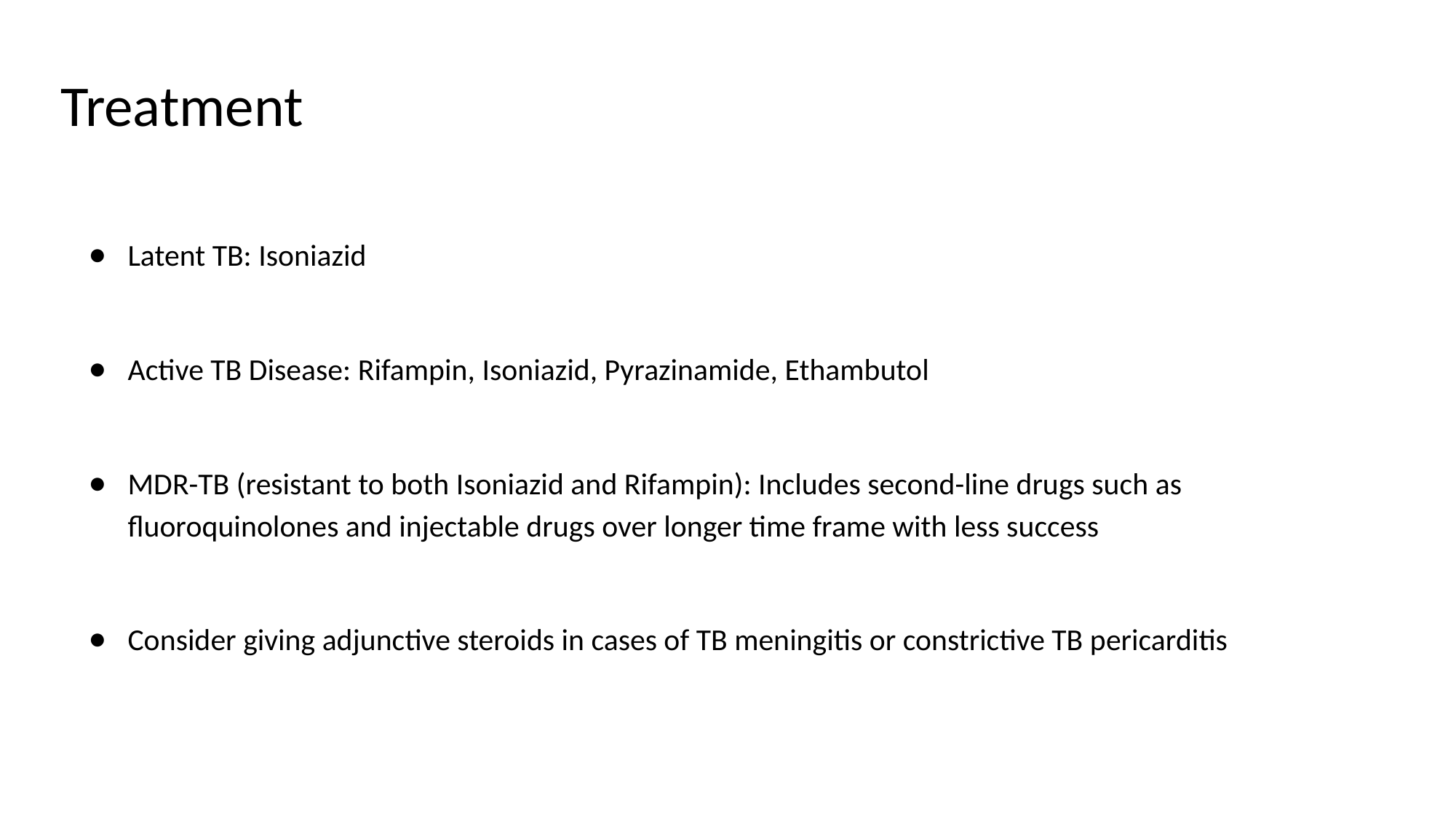

# Treatment
Latent TB: Isoniazid
Active TB Disease: Rifampin, Isoniazid, Pyrazinamide, Ethambutol
MDR-TB (resistant to both Isoniazid and Rifampin): Includes second-line drugs such as fluoroquinolones and injectable drugs over longer time frame with less success
Consider giving adjunctive steroids in cases of TB meningitis or constrictive TB pericarditis

## Slide 36
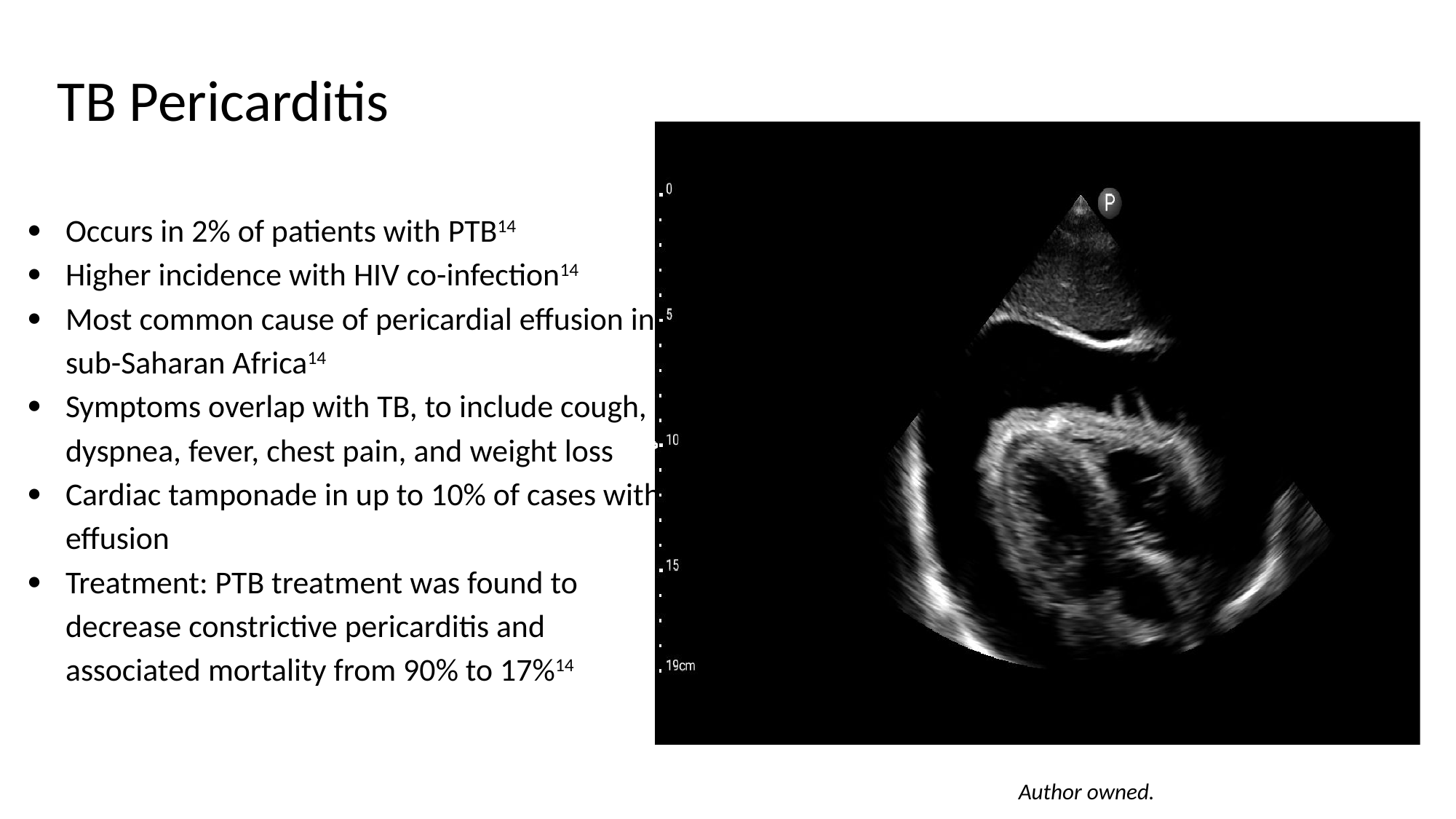

# TB Pericarditis
Occurs in 2% of patients with PTB14
Higher incidence with HIV co-infection14
Most common cause of pericardial effusion in sub-Saharan Africa14
Symptoms overlap with TB, to include cough, dyspnea, fever, chest pain, and weight loss
Cardiac tamponade in up to 10% of cases with effusion
Treatment: PTB treatment was found to decrease constrictive pericarditis and associated mortality from 90% to 17%14
Author owned.

## Slide 37
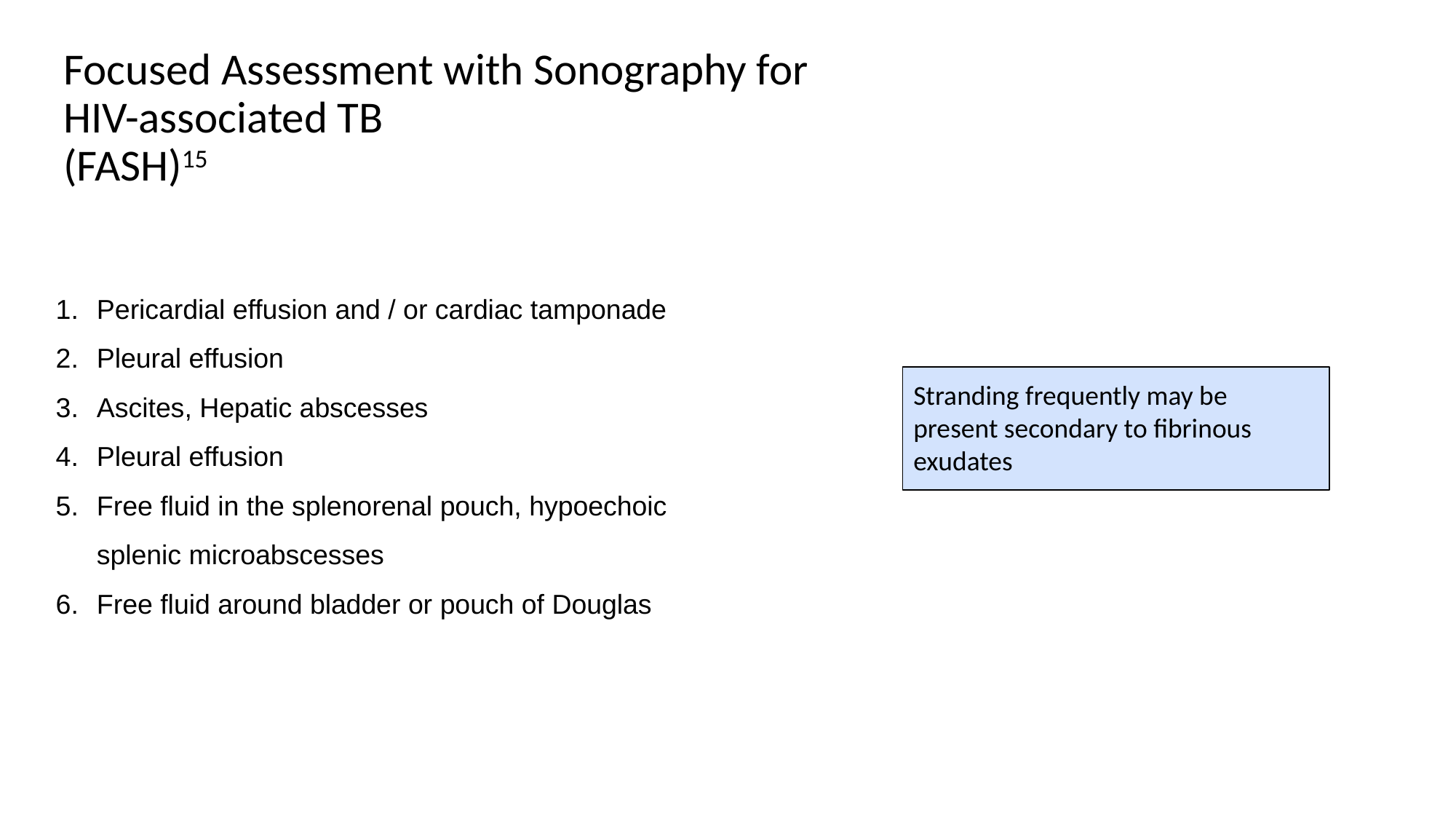

# Focused Assessment with Sonography for HIV-associated TB
(FASH)15
Pericardial effusion and / or cardiac tamponade
Pleural effusion
Ascites, Hepatic abscesses
Pleural effusion
Free fluid in the splenorenal pouch, hypoechoic splenic microabscesses
Free fluid around bladder or pouch of Douglas
Stranding frequently may be present secondary to fibrinous exudates

## Slide 38
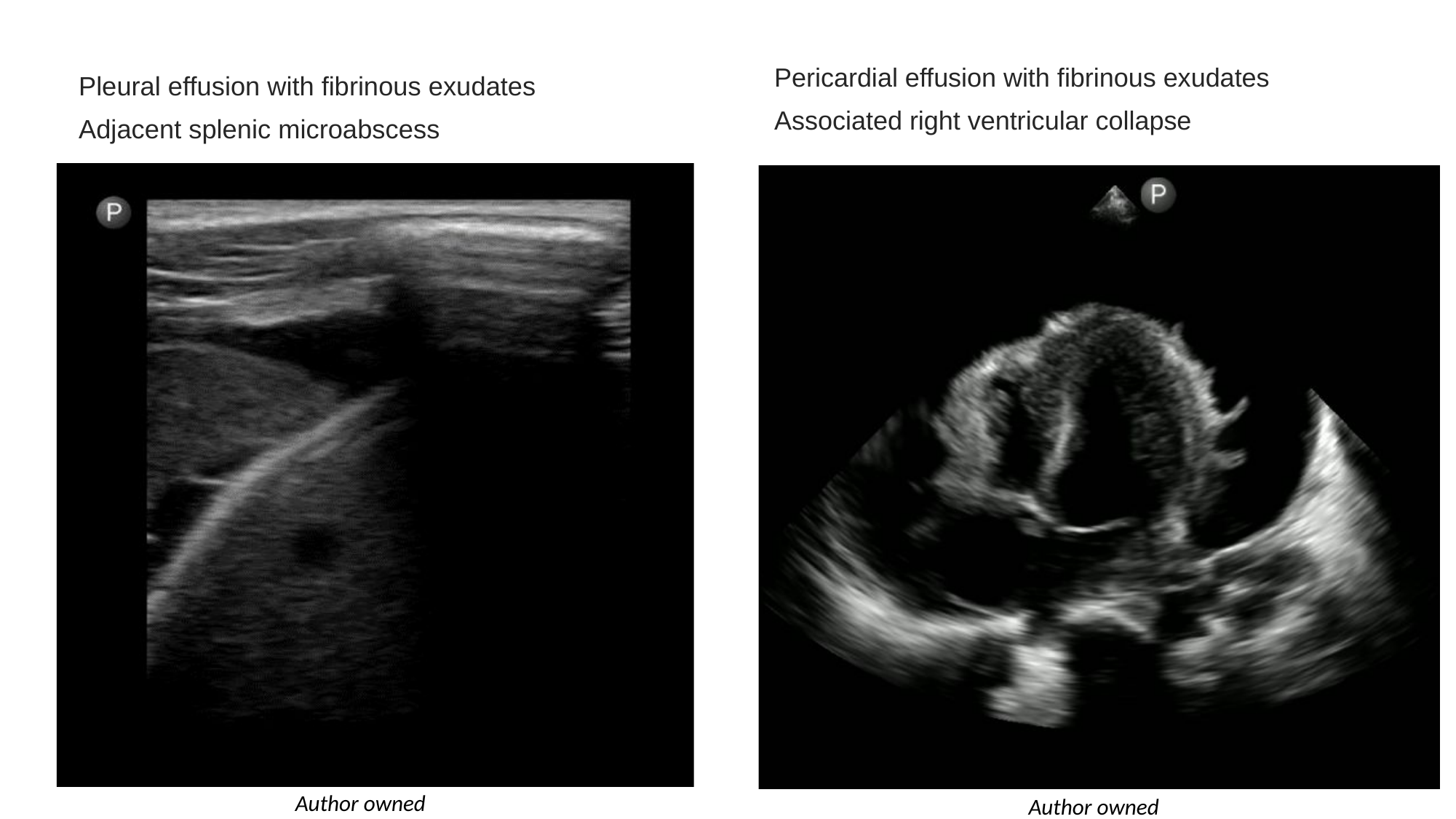

Pericardial effusion with fibrinous exudates
Associated right ventricular collapse
Pleural effusion with fibrinous exudates
Adjacent splenic microabscess
Author owned
Author owned

## Slide 39
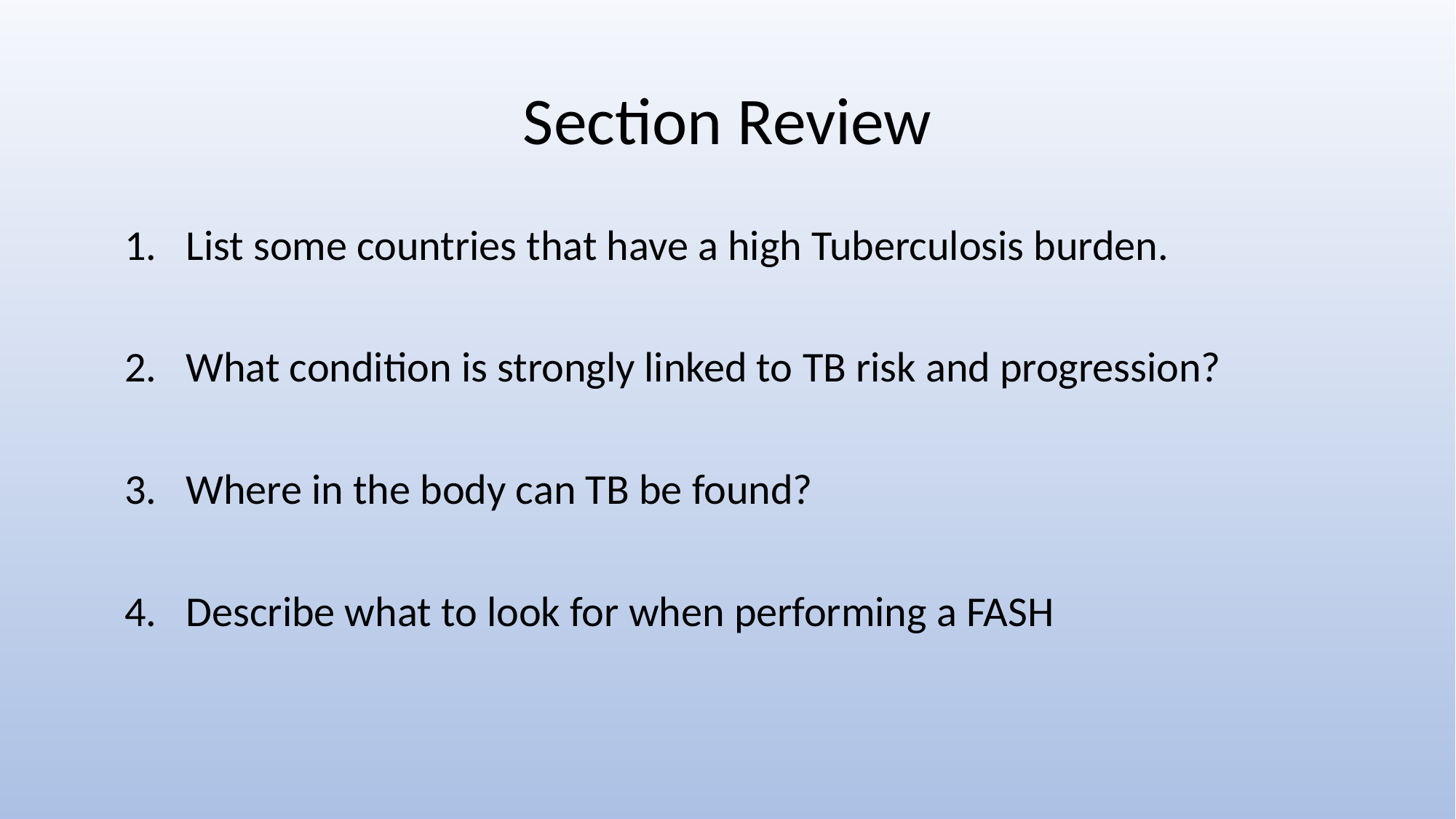

# Section Review
List some countries that have a high Tuberculosis burden.
What condition is strongly linked to TB risk and progression?
Where in the body can TB be found?
Describe what to look for when performing a FASH

## Slide 40
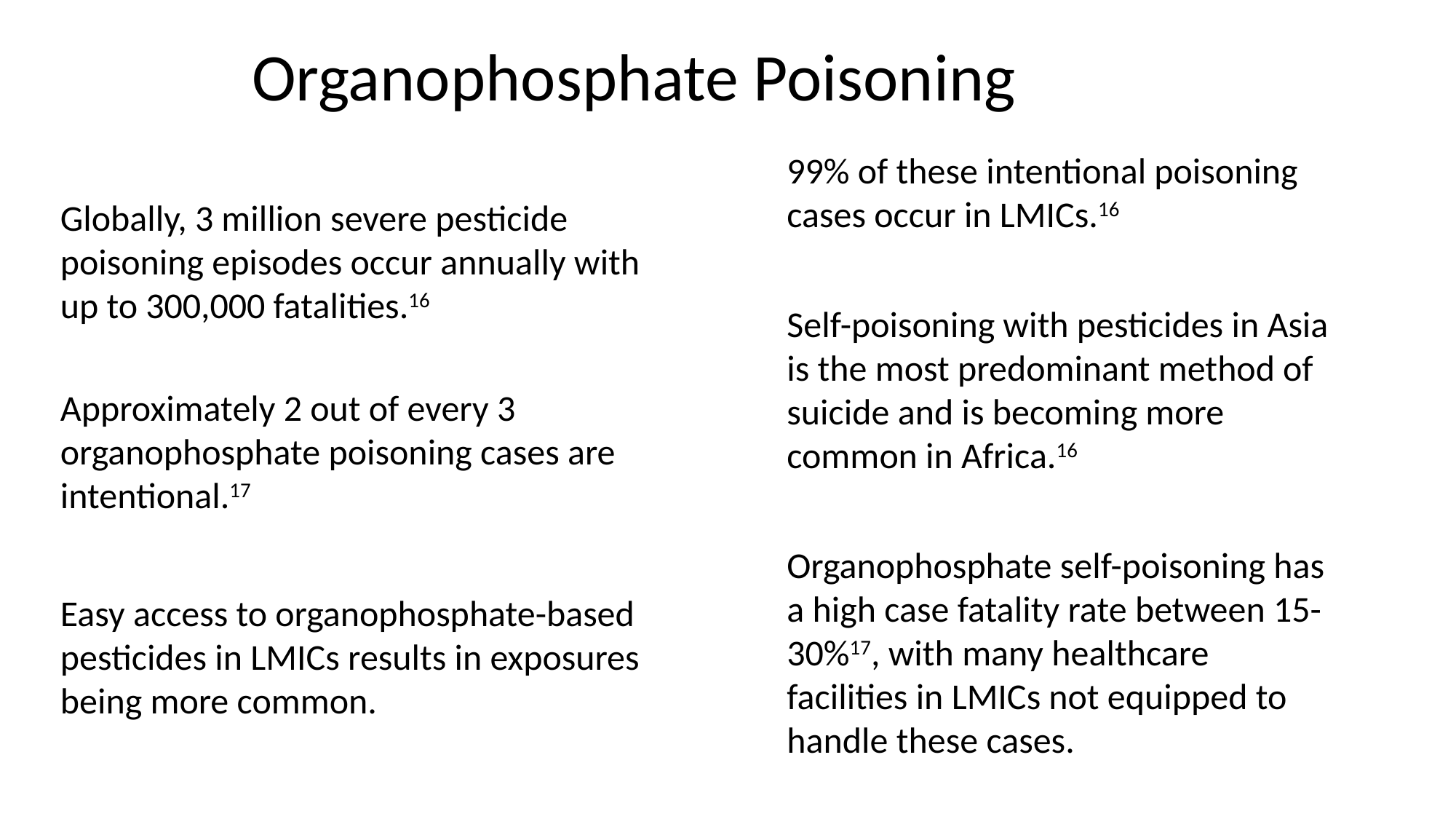

# Organophosphate Poisoning
Globally, 3 million severe pesticide poisoning episodes occur annually with up to 300,000 fatalities.16
Approximately 2 out of every 3 organophosphate poisoning cases are intentional.17
Easy access to organophosphate-based pesticides in LMICs results in exposures being more common.
99% of these intentional poisoning cases occur in LMICs.16
Self-poisoning with pesticides in Asia is the most predominant method of suicide and is becoming more common in Africa.16
Organophosphate self-poisoning has a high case fatality rate between 15-30%17, with many healthcare facilities in LMICs not equipped to handle these cases.

## Slide 41
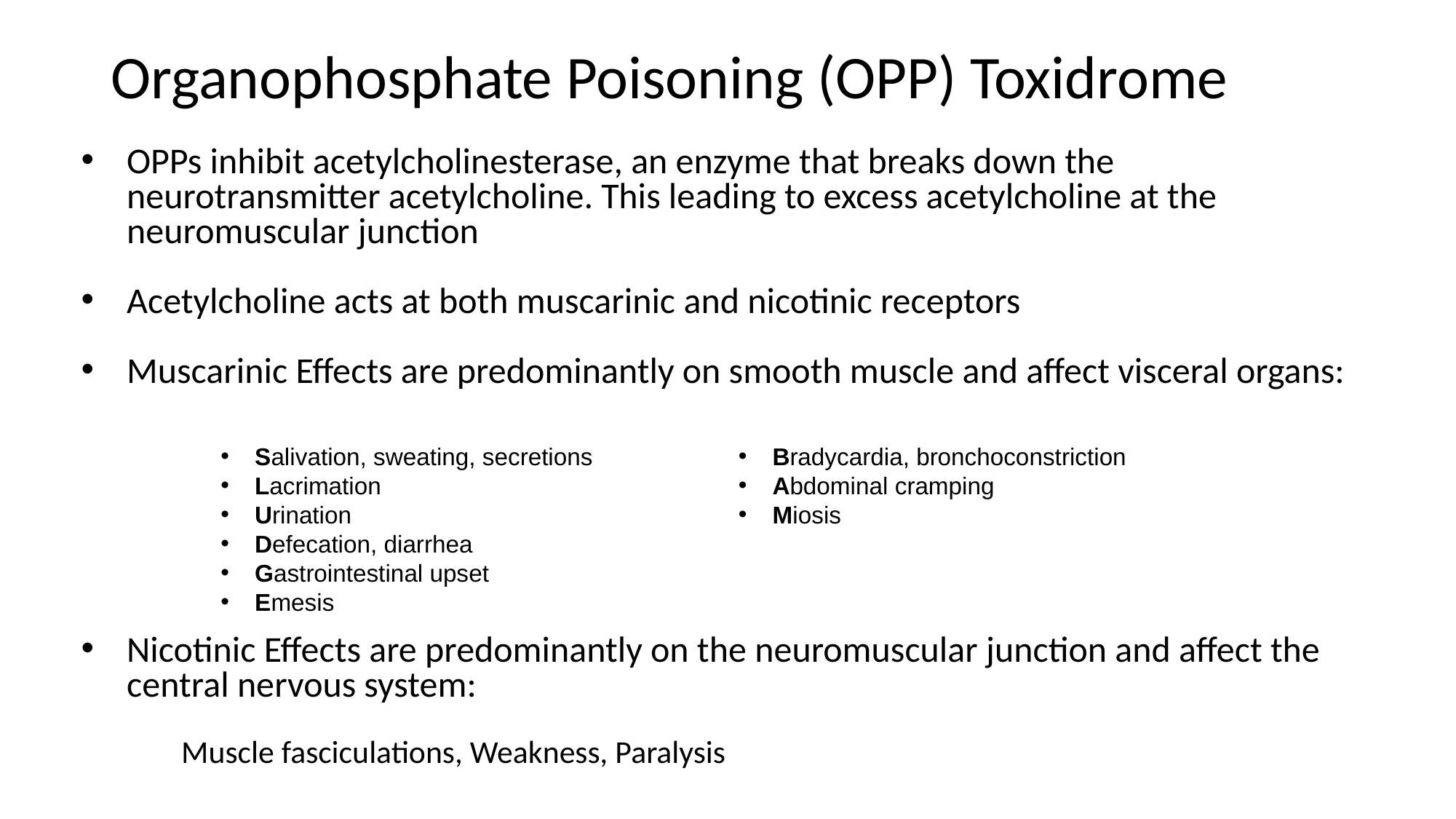

# Organophosphate Poisoning (OPP) Toxidrome
OPPs inhibit acetylcholinesterase, an enzyme that breaks down the neurotransmitter acetylcholine. This leading to excess acetylcholine at the neuromuscular junction
Acetylcholine acts at both muscarinic and nicotinic receptors
Muscarinic Effects are predominantly on smooth muscle and affect visceral organs:
Nicotinic Effects are predominantly on the neuromuscular junction and affect the central nervous system:
Muscle fasciculations, Weakness, Paralysis
Salivation, sweating, secretions
Lacrimation
Urination
Defecation, diarrhea
Gastrointestinal upset
Emesis
Bradycardia, bronchoconstriction
Abdominal cramping
Miosis

## Slide 42
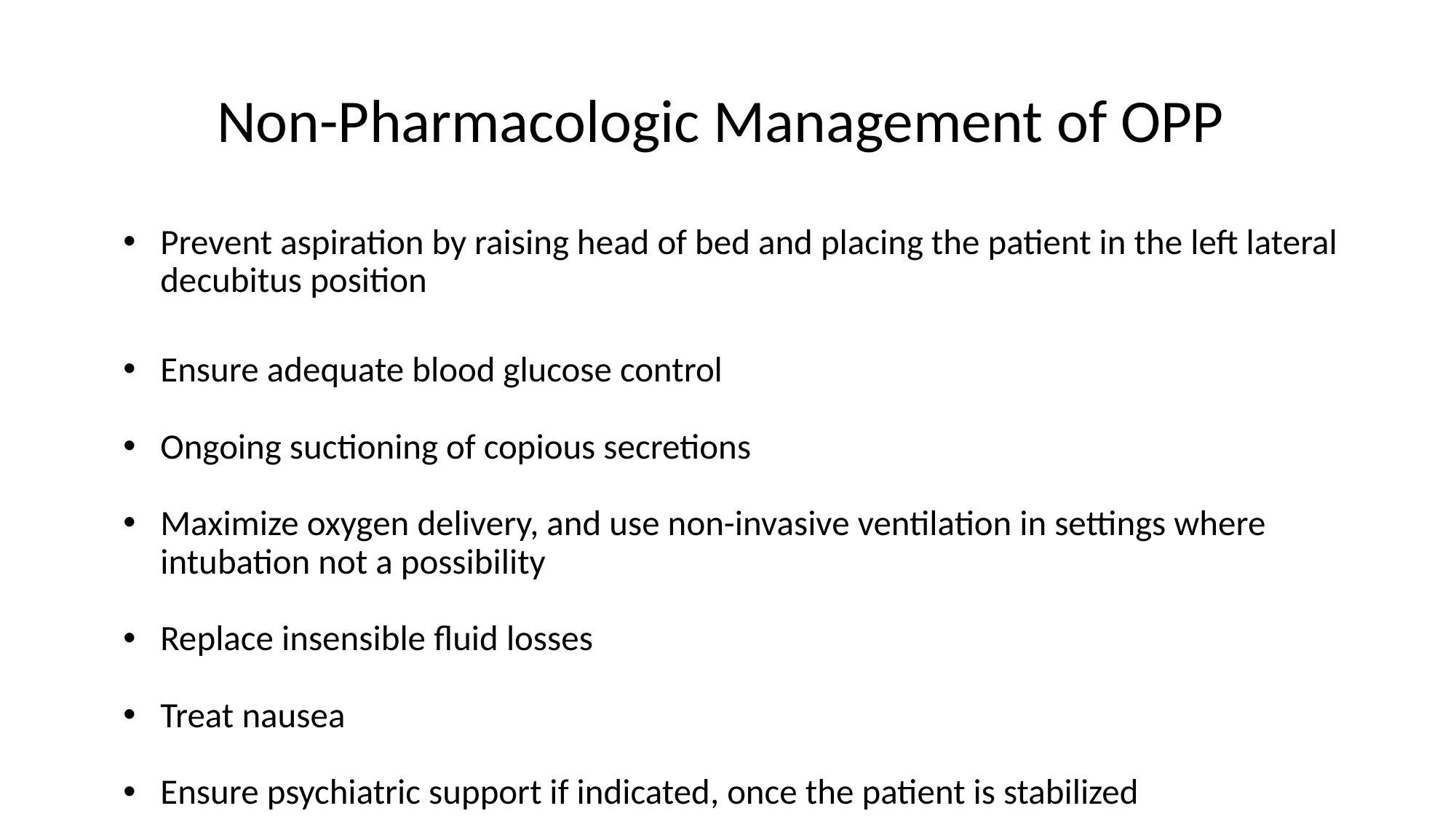

# Non-Pharmacologic Management of OPP
Prevent aspiration by raising head of bed and placing the patient in the left lateral decubitus position
Ensure adequate blood glucose control
Ongoing suctioning of copious secretions
Maximize oxygen delivery, and use non-invasive ventilation in settings where intubation not a possibility
Replace insensible fluid losses
Treat nausea
Ensure psychiatric support if indicated, once the patient is stabilized

## Slide 43
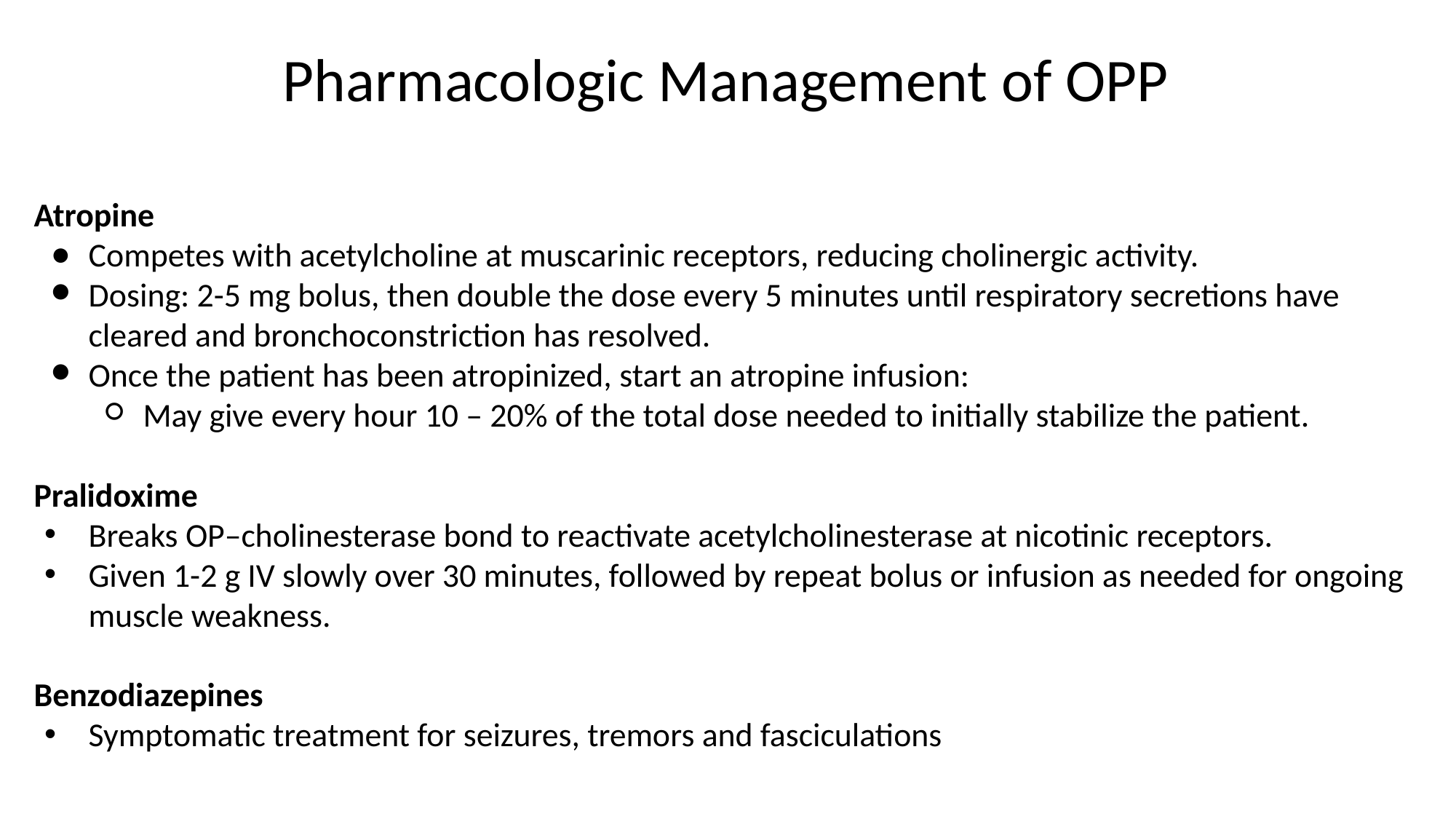

Pharmacologic Management of OPP
Atropine
Competes with acetylcholine at muscarinic receptors, reducing cholinergic activity.
Dosing: 2-5 mg bolus, then double the dose every 5 minutes until respiratory secretions have cleared and bronchoconstriction has resolved.
Once the patient has been atropinized, start an atropine infusion:
May give every hour 10 – 20% of the total dose needed to initially stabilize the patient.
Pralidoxime
Breaks OP–cholinesterase bond to reactivate acetylcholinesterase at nicotinic receptors.
Given 1-2 g IV slowly over 30 minutes, followed by repeat bolus or infusion as needed for ongoing muscle weakness.
Benzodiazepines
Symptomatic treatment for seizures, tremors and fasciculations

## Slide 44
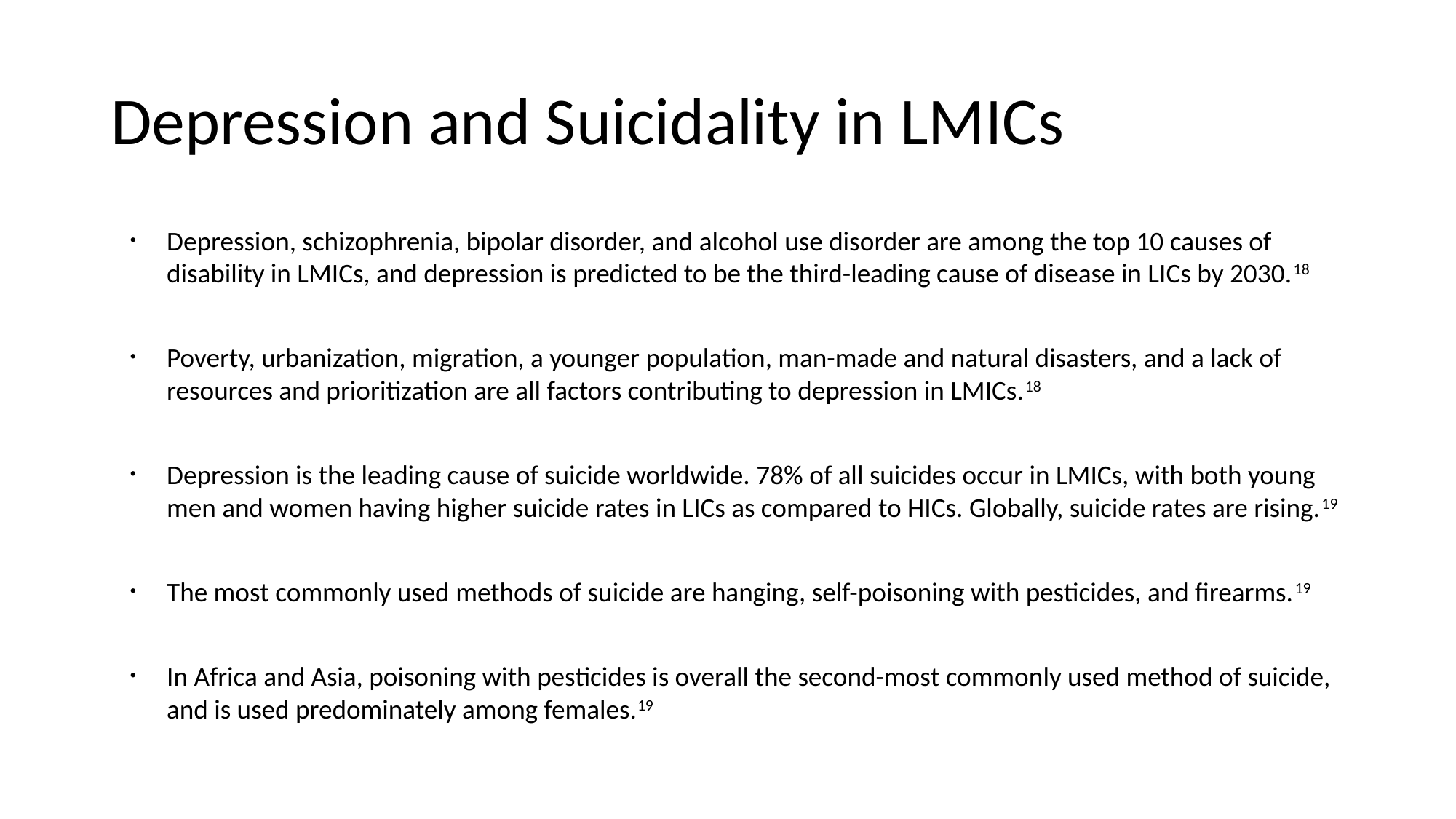

# Depression and Suicidality in LMICs
Depression, schizophrenia, bipolar disorder, and alcohol use disorder are among the top 10 causes of disability in LMICs, and depression is predicted to be the third-leading cause of disease in LICs by 2030.18
Poverty, urbanization, migration, a younger population, man-made and natural disasters, and a lack of resources and prioritization are all factors contributing to depression in LMICs.18
Depression is the leading cause of suicide worldwide. 78% of all suicides occur in LMICs, with both young men and women having higher suicide rates in LICs as compared to HICs. Globally, suicide rates are rising.19
The most commonly used methods of suicide are hanging, self-poisoning with pesticides, and firearms.19
In Africa and Asia, poisoning with pesticides is overall the second-most commonly used method of suicide, and is used predominately among females.19

## Slide 45
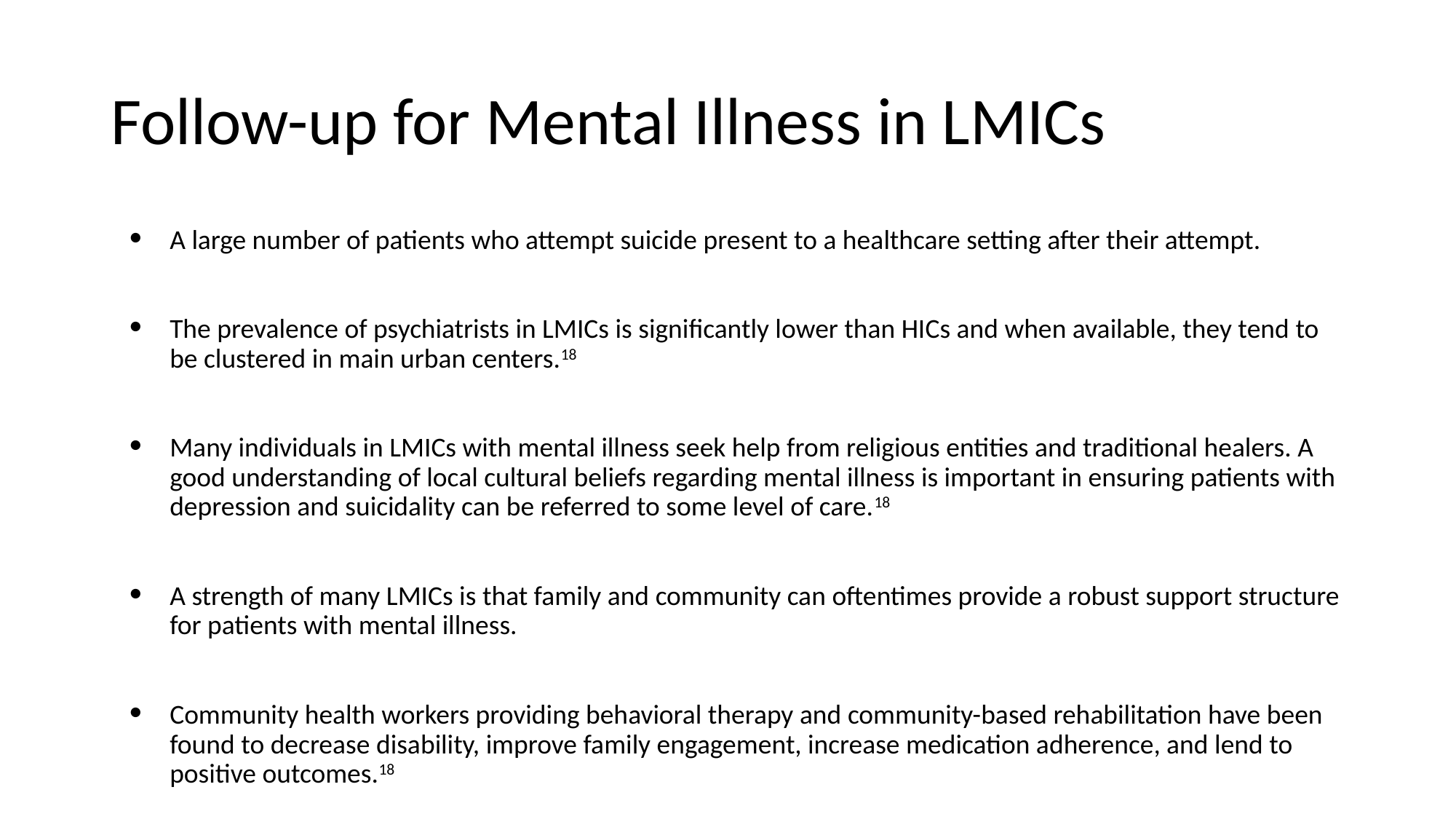

# Follow-up for Mental Illness in LMICs
A large number of patients who attempt suicide present to a healthcare setting after their attempt.
The prevalence of psychiatrists in LMICs is significantly lower than HICs and when available, they tend to be clustered in main urban centers.18
Many individuals in LMICs with mental illness seek help from religious entities and traditional healers. A good understanding of local cultural beliefs regarding mental illness is important in ensuring patients with depression and suicidality can be referred to some level of care.18
A strength of many LMICs is that family and community can oftentimes provide a robust support structure for patients with mental illness.
Community health workers providing behavioral therapy and community-based rehabilitation have been found to decrease disability, improve family engagement, increase medication adherence, and lend to positive outcomes.18

## Slide 46
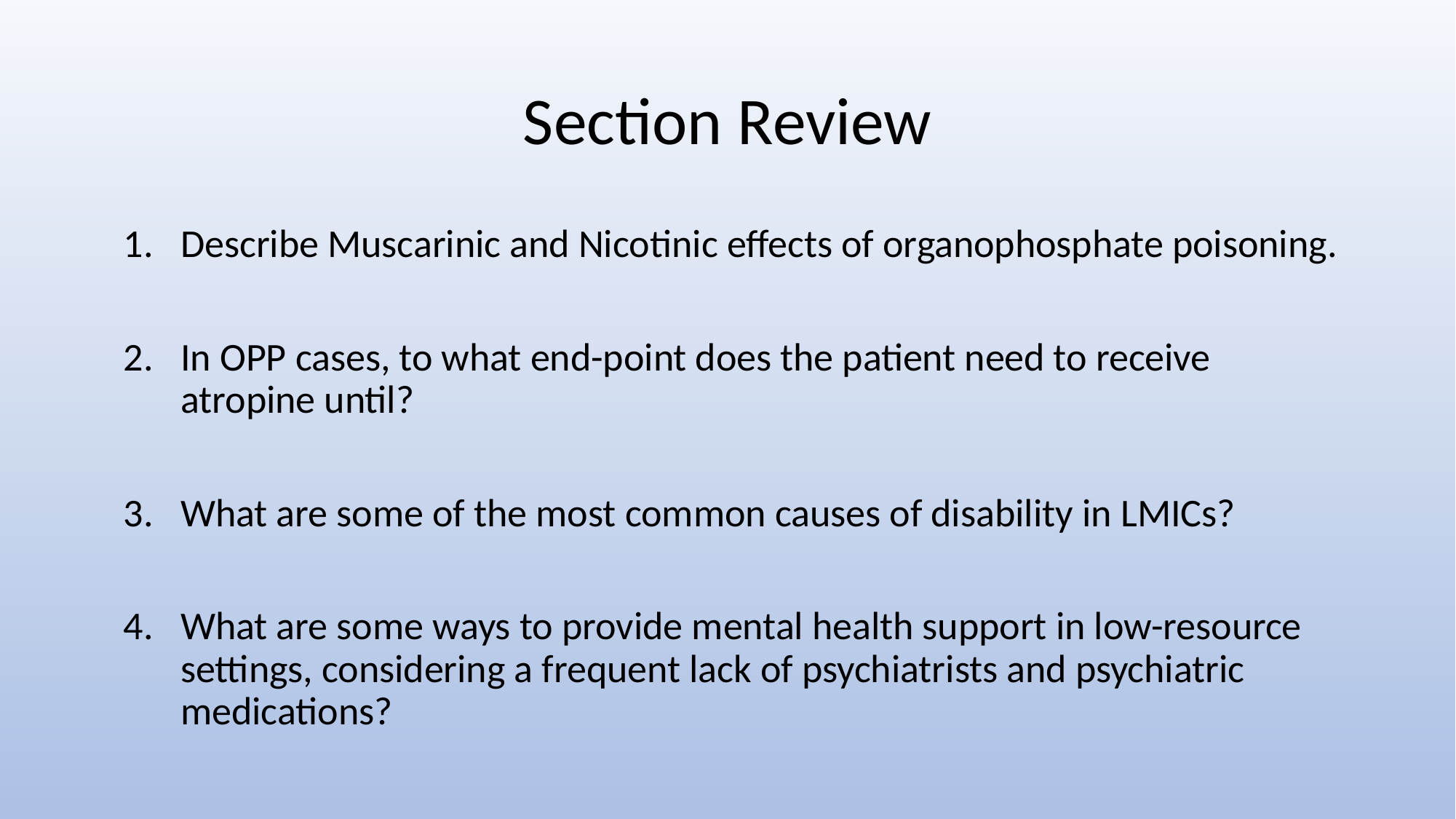

# Section Review
Describe Muscarinic and Nicotinic effects of organophosphate poisoning.
In OPP cases, to what end-point does the patient need to receive atropine until?
What are some of the most common causes of disability in LMICs?
What are some ways to provide mental health support in low-resource settings, considering a frequent lack of psychiatrists and psychiatric medications?

## Slide 47
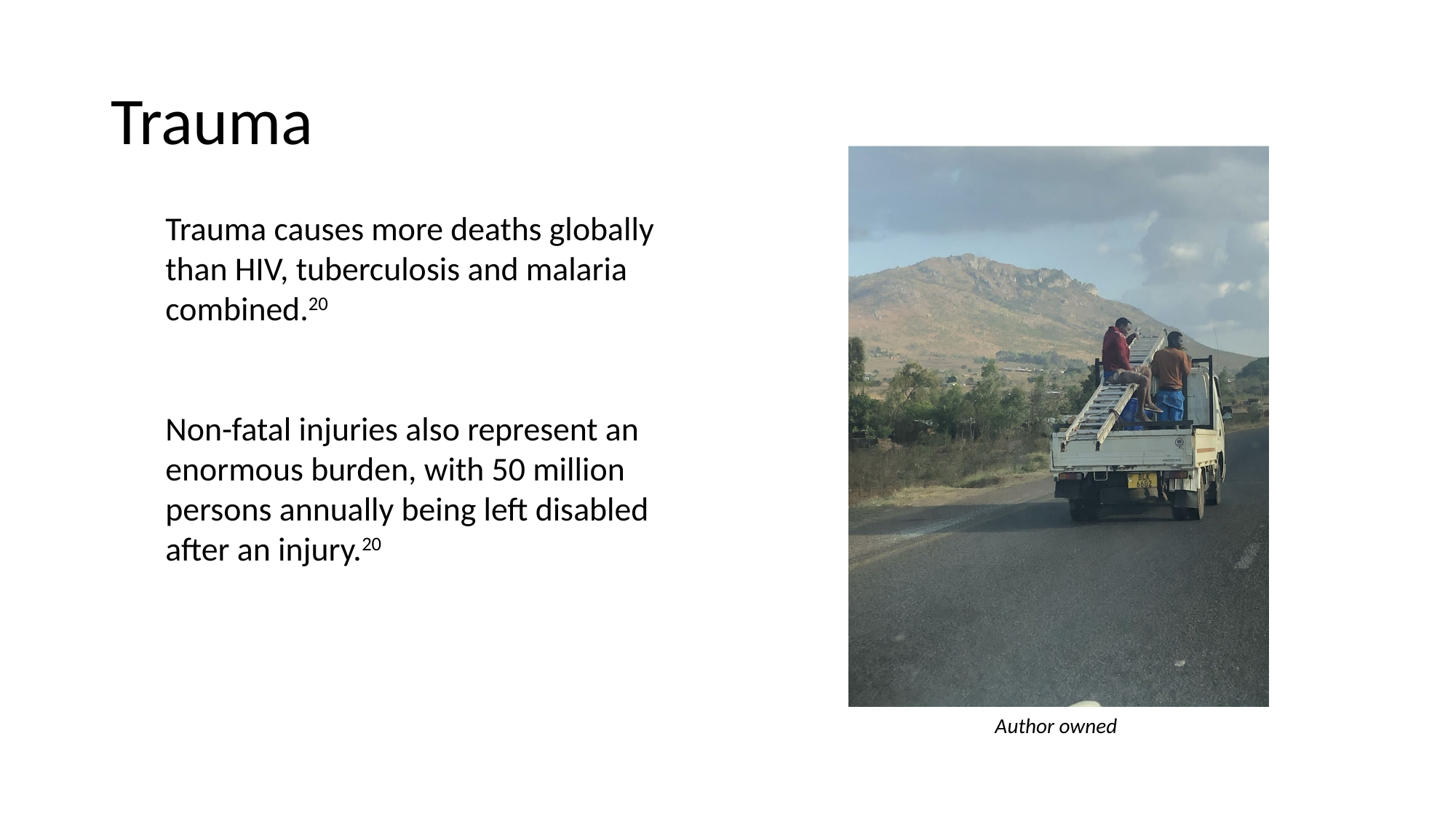

# Trauma
Trauma causes more deaths globally than HIV, tuberculosis and malaria combined.20
Non-fatal injuries also represent an enormous burden, with 50 million persons annually being left disabled after an injury.20
Author owned

## Slide 48
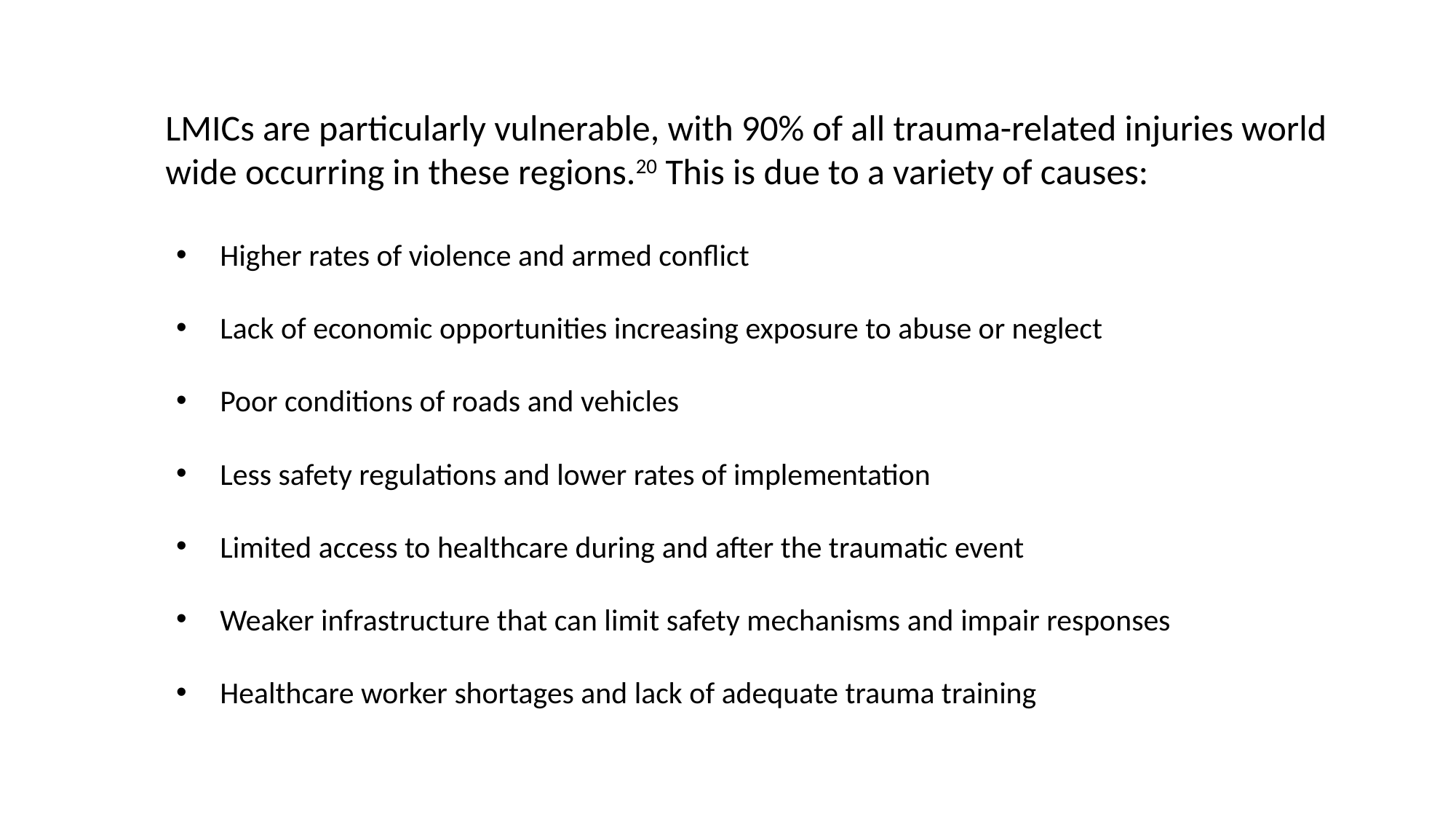

LMICs are particularly vulnerable, with 90% of all trauma-related injuries world wide occurring in these regions.20 This is due to a variety of causes:
Higher rates of violence and armed conflict
Lack of economic opportunities increasing exposure to abuse or neglect
Poor conditions of roads and vehicles
Less safety regulations and lower rates of implementation
Limited access to healthcare during and after the traumatic event
Weaker infrastructure that can limit safety mechanisms and impair responses
Healthcare worker shortages and lack of adequate trauma training

## Slide 49
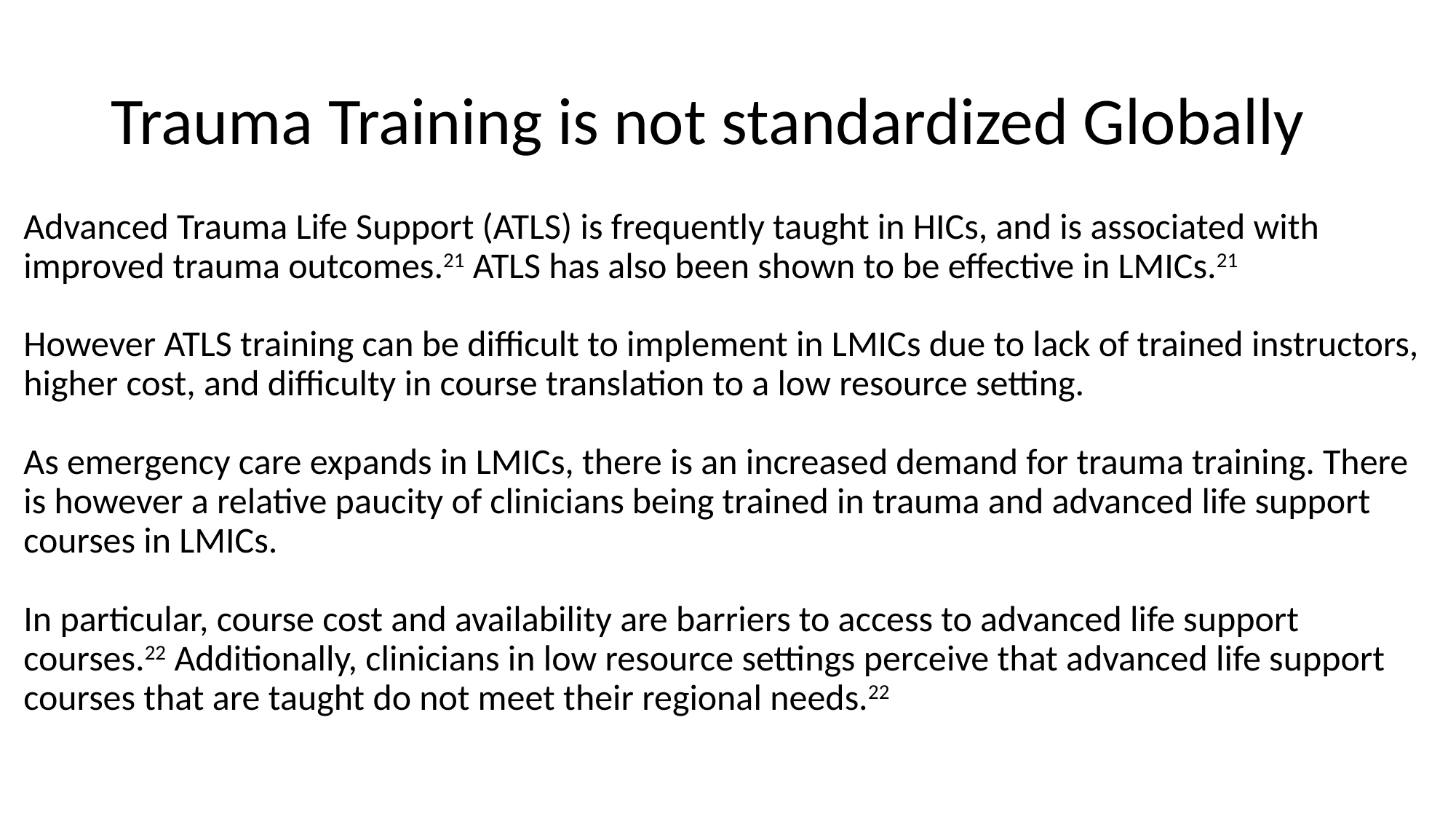

# Trauma Training is not standardized Globally
Advanced Trauma Life Support (ATLS) is frequently taught in HICs, and is associated with improved trauma outcomes.21 ATLS has also been shown to be effective in LMICs.21
However ATLS training can be difficult to implement in LMICs due to lack of trained instructors, higher cost, and difficulty in course translation to a low resource setting.
As emergency care expands in LMICs, there is an increased demand for trauma training. There is however a relative paucity of clinicians being trained in trauma and advanced life support courses in LMICs.
In particular, course cost and availability are barriers to access to advanced life support courses.22 Additionally, clinicians in low resource settings perceive that advanced life support courses that are taught do not meet their regional needs.22

## Slide 50
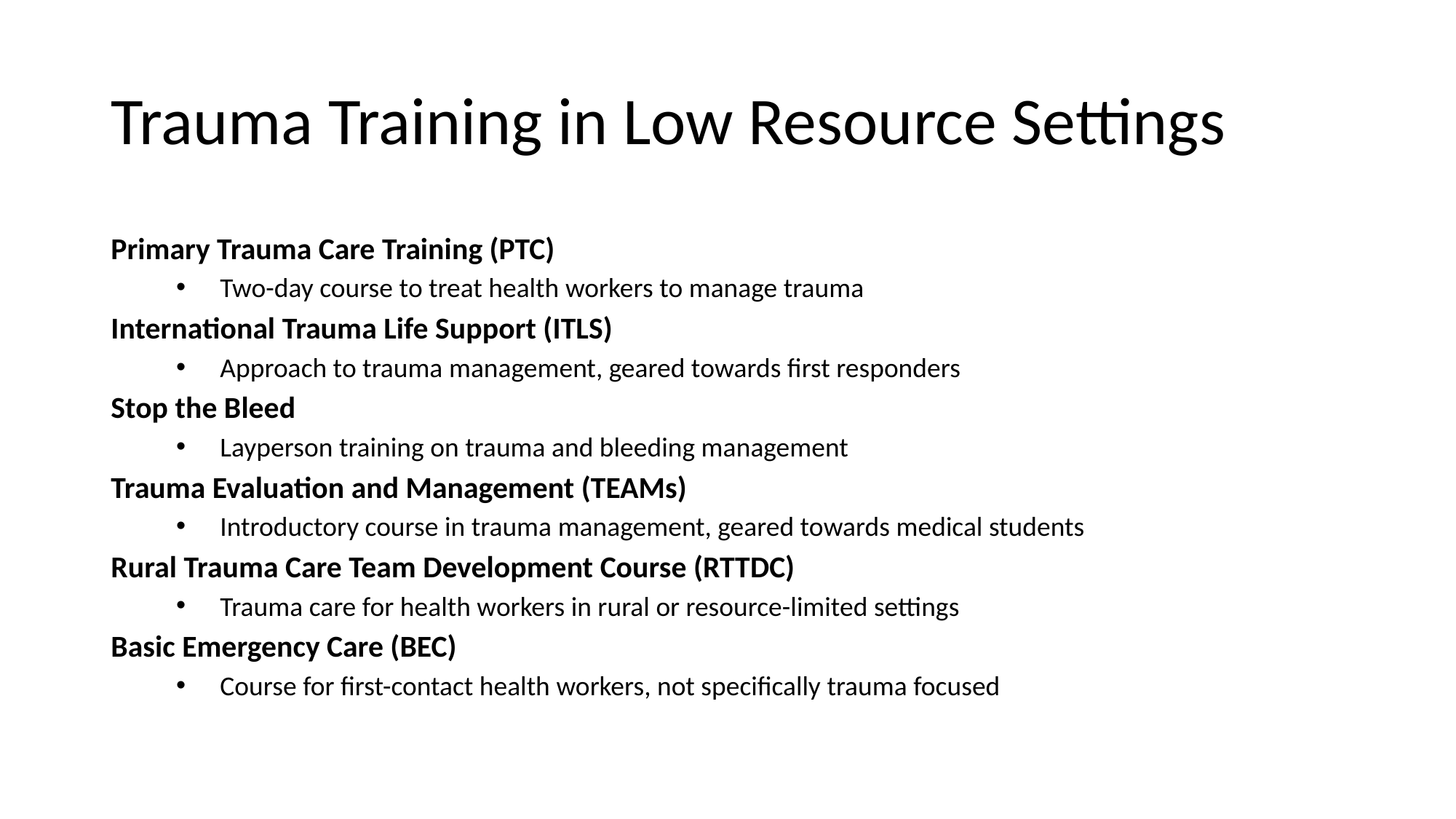

# Trauma Training in Low Resource Settings
Primary Trauma Care Training (PTC)
Two-day course to treat health workers to manage trauma
International Trauma Life Support (ITLS)
Approach to trauma management, geared towards first responders
Stop the Bleed
Layperson training on trauma and bleeding management
Trauma Evaluation and Management (TEAMs)
Introductory course in trauma management, geared towards medical students
Rural Trauma Care Team Development Course (RTTDC)
Trauma care for health workers in rural or resource-limited settings
Basic Emergency Care (BEC)
Course for first-contact health workers, not specifically trauma focused

## Slide 51
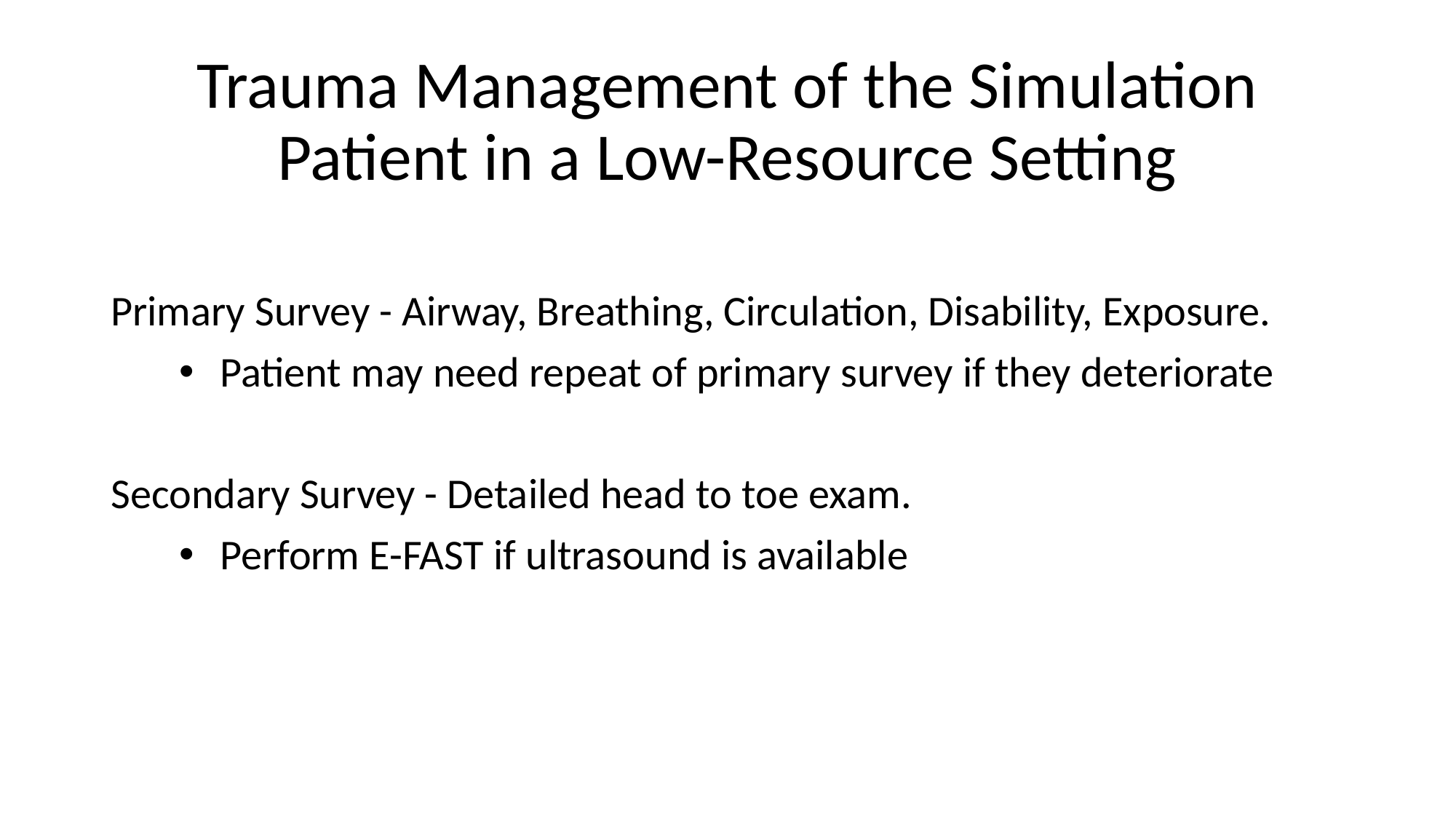

# Trauma Management of the Simulation Patient in a Low-Resource Setting
Primary Survey - Airway, Breathing, Circulation, Disability, Exposure.
Patient may need repeat of primary survey if they deteriorate
Secondary Survey - Detailed head to toe exam.
Perform E-FAST if ultrasound is available

## Slide 52
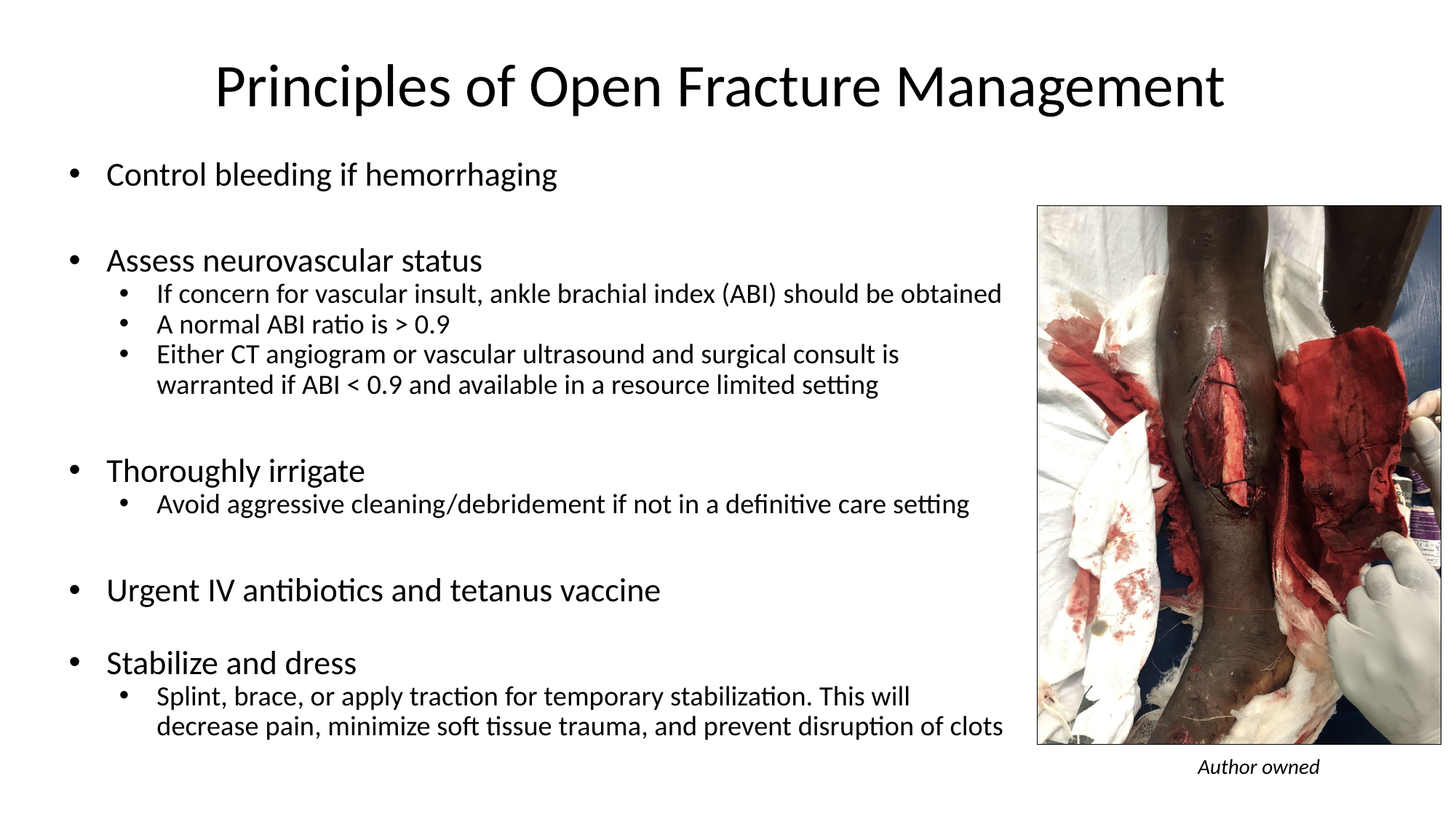

# Principles of Open Fracture Management
Control bleeding if hemorrhaging
Assess neurovascular status
If concern for vascular insult, ankle brachial index (ABI) should be obtained
A normal ABI ratio is > 0.9
Either CT angiogram or vascular ultrasound and surgical consult is warranted if ABI < 0.9 and available in a resource limited setting
Thoroughly irrigate
Avoid aggressive cleaning/debridement if not in a definitive care setting
Urgent IV antibiotics and tetanus vaccine
Stabilize and dress
Splint, brace, or apply traction for temporary stabilization. This will decrease pain, minimize soft tissue trauma, and prevent disruption of clots
Author owned

## Slide 53
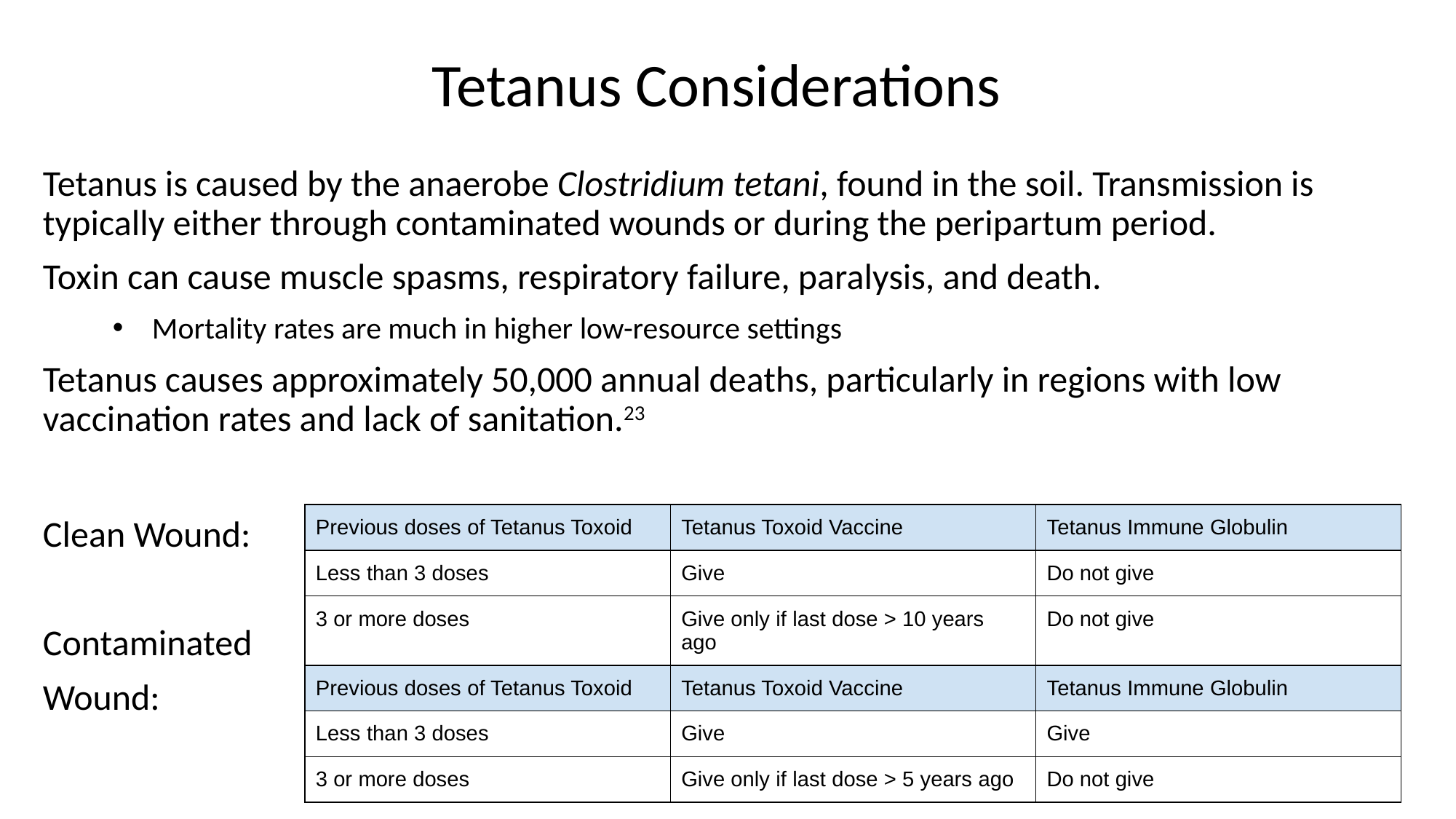

# Tetanus Considerations
Tetanus is caused by the anaerobe Clostridium tetani, found in the soil. Transmission is typically either through contaminated wounds or during the peripartum period.
Toxin can cause muscle spasms, respiratory failure, paralysis, and death.
Mortality rates are much in higher low-resource settings
Tetanus causes approximately 50,000 annual deaths, particularly in regions with low vaccination rates and lack of sanitation.23
Clean Wound:
Contaminated
Wound:
| Previous doses of Tetanus Toxoid | Tetanus Toxoid Vaccine | Tetanus Immune Globulin |
| --- | --- | --- |
| Less than 3 doses | Give | Do not give |
| 3 or more doses | Give only if last dose > 10 years ago | Do not give |
| Previous doses of Tetanus Toxoid | Tetanus Toxoid Vaccine | Tetanus Immune Globulin |
| --- | --- | --- |
| Less than 3 doses | Give | Give |
| 3 or more doses | Give only if last dose > 5 years ago | Do not give |

## Slide 54
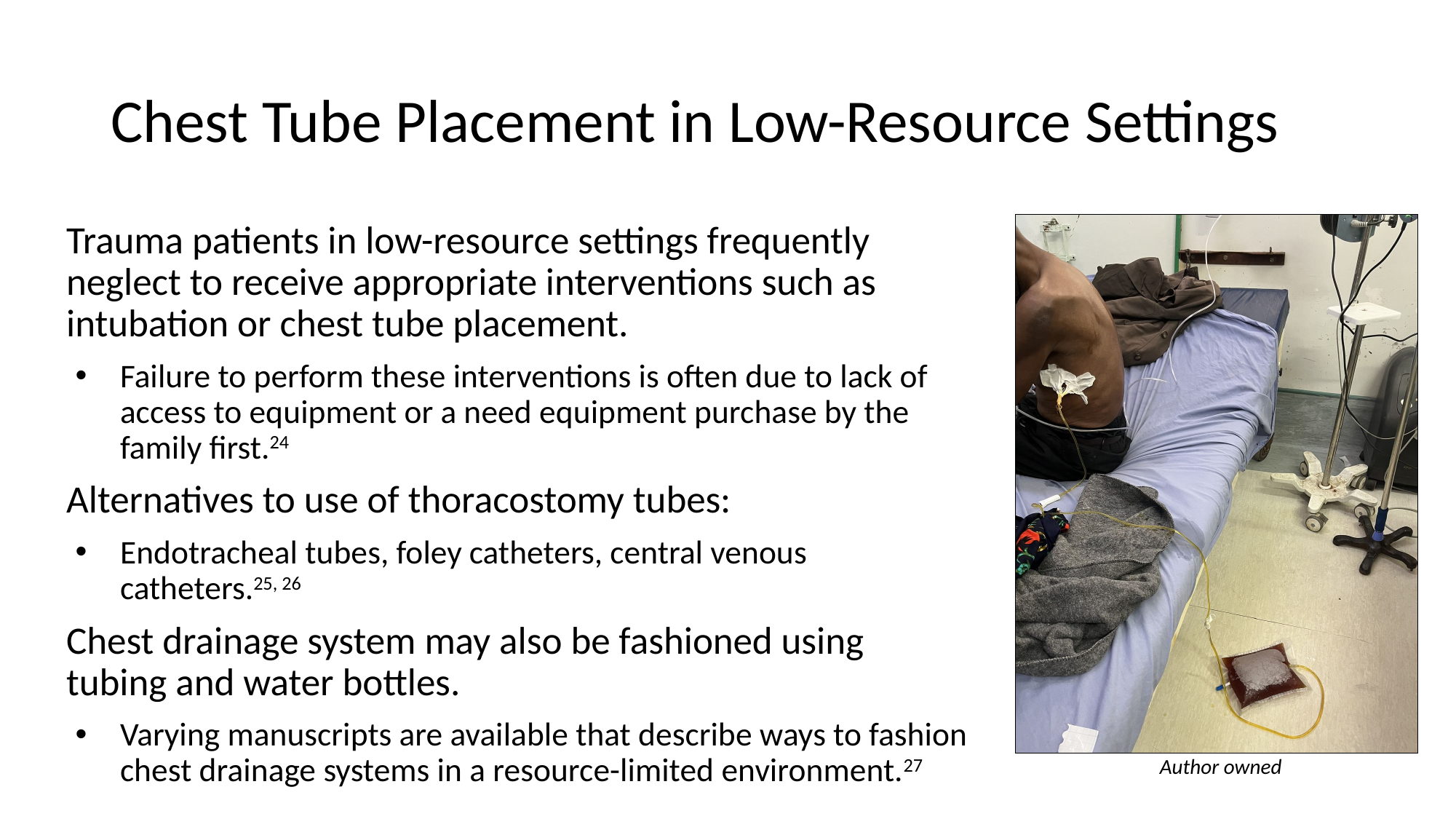

# Chest Tube Placement in Low-Resource Settings
Trauma patients in low-resource settings frequently neglect to receive appropriate interventions such as intubation or chest tube placement.
Failure to perform these interventions is often due to lack of access to equipment or a need equipment purchase by the family first.24
Alternatives to use of thoracostomy tubes:
Endotracheal tubes, foley catheters, central venous catheters.25, 26
Chest drainage system may also be fashioned using tubing and water bottles.
Varying manuscripts are available that describe ways to fashion chest drainage systems in a resource-limited environment.27
Author owned

## Slide 55
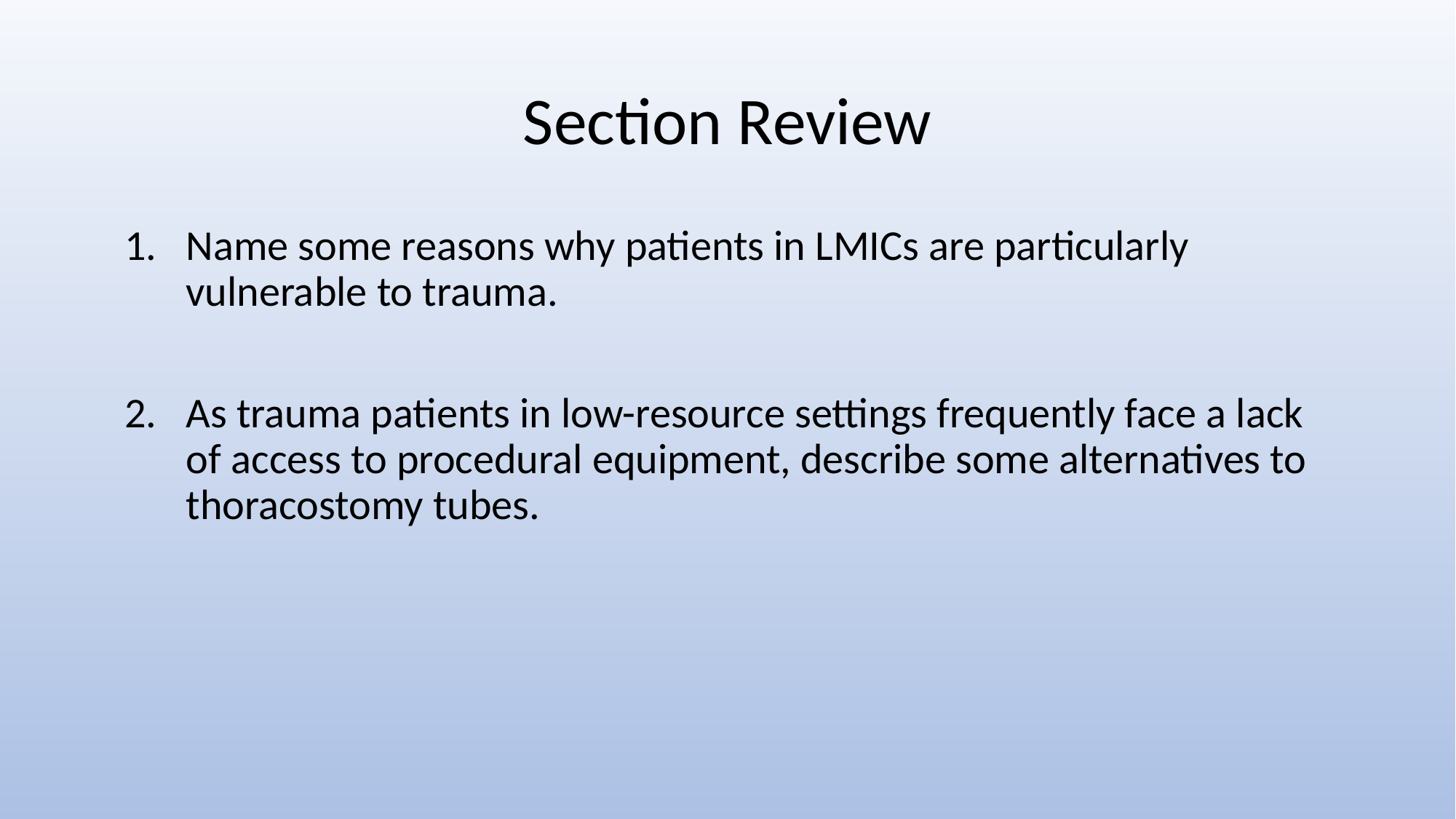

# Section Review
Name some reasons why patients in LMICs are particularly vulnerable to trauma.
As trauma patients in low-resource settings frequently face a lack of access to procedural equipment, describe some alternatives to thoracostomy tubes.

## Slide 56
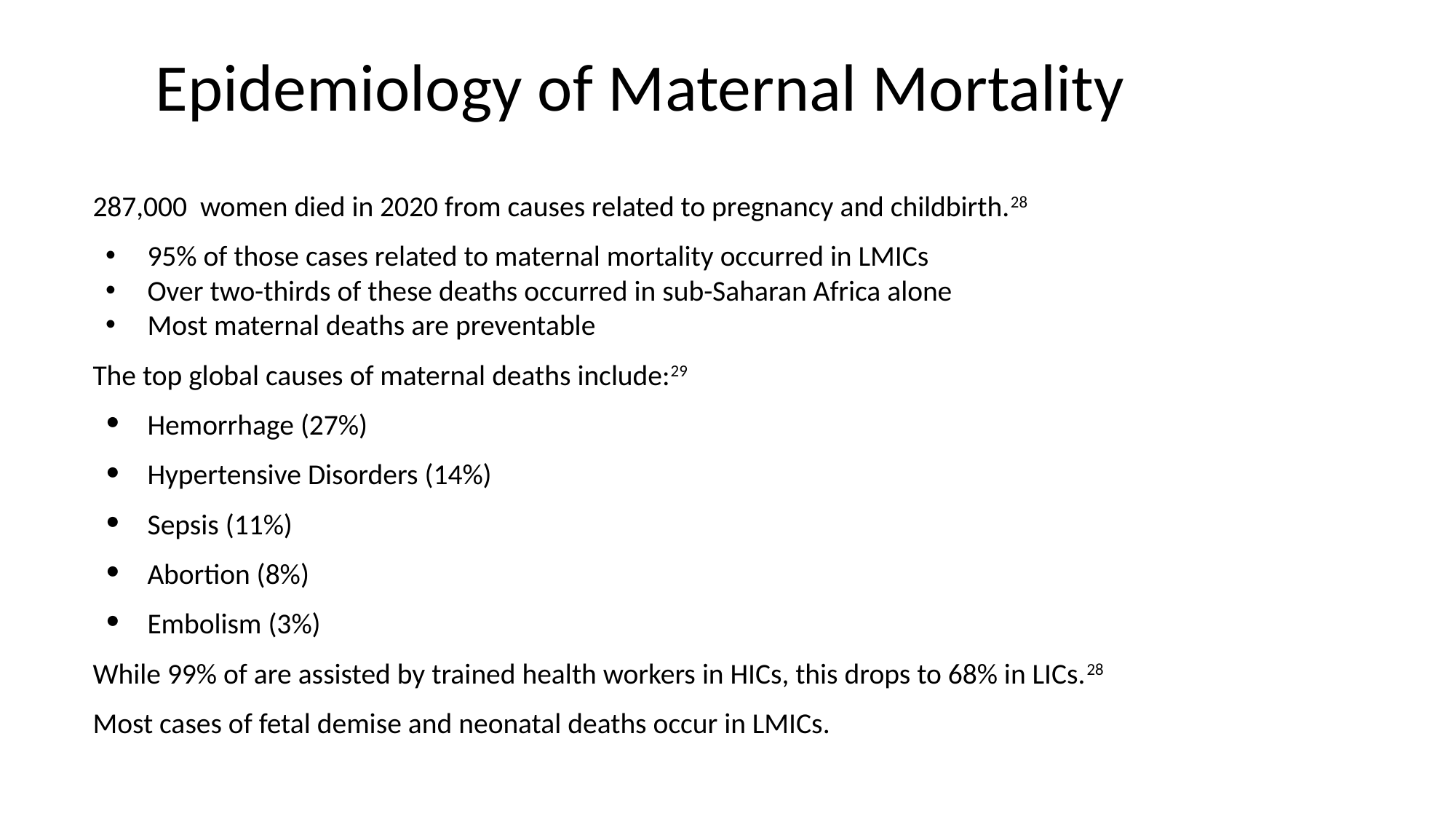

# Epidemiology of Maternal Mortality
287,000 women died in 2020 from causes related to pregnancy and childbirth.28
95% of those cases related to maternal mortality occurred in LMICs
Over two-thirds of these deaths occurred in sub-Saharan Africa alone
Most maternal deaths are preventable
The top global causes of maternal deaths include:29
Hemorrhage (27%)
Hypertensive Disorders (14%)
Sepsis (11%)
Abortion (8%)
Embolism (3%)
While 99% of are assisted by trained health workers in HICs, this drops to 68% in LICs.28
Most cases of fetal demise and neonatal deaths occur in LMICs.

## Slide 57
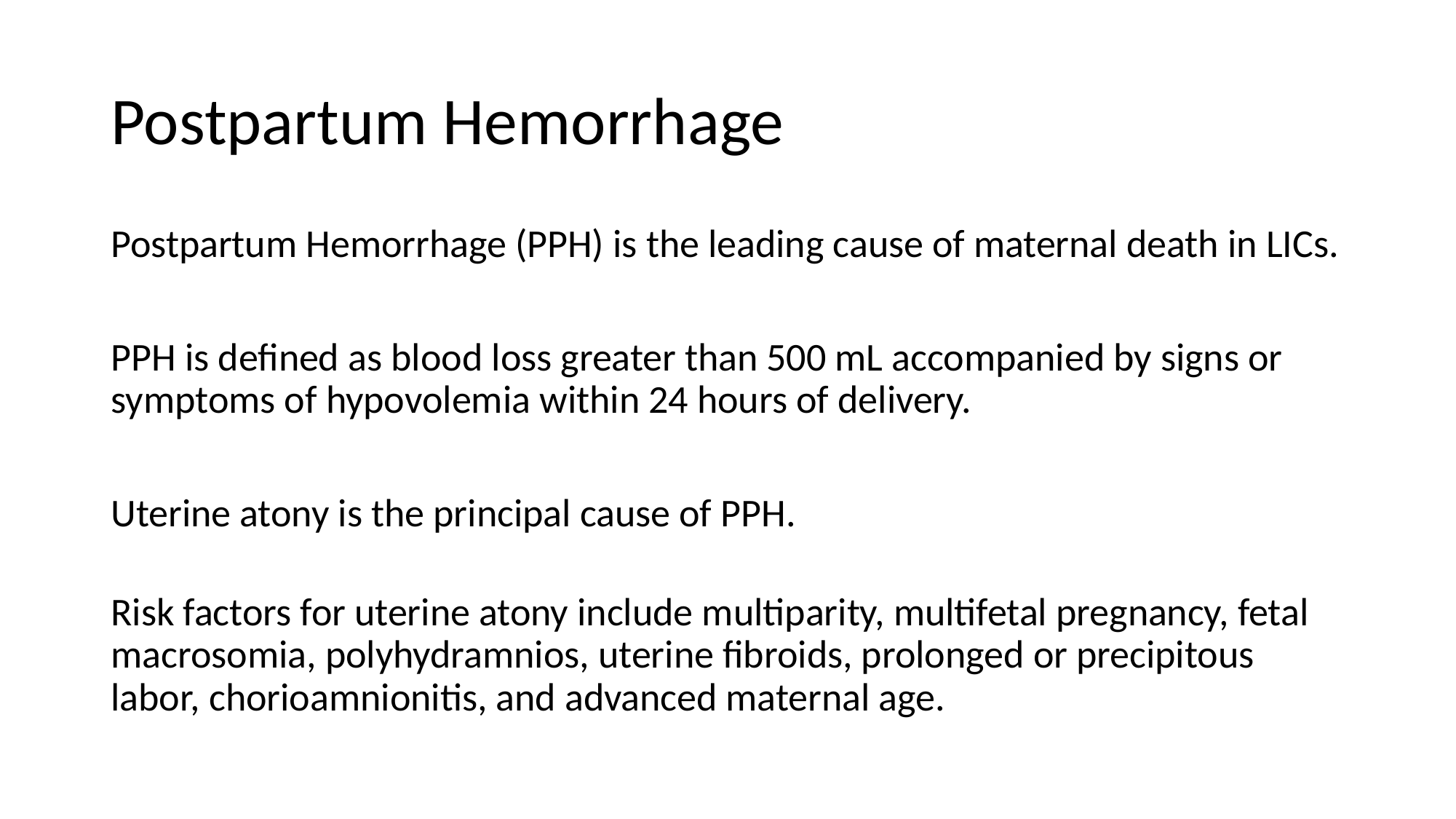

# Postpartum Hemorrhage
Postpartum Hemorrhage (PPH) is the leading cause of maternal death in LICs.
PPH is defined as blood loss greater than 500 mL accompanied by signs or symptoms of hypovolemia within 24 hours of delivery.
Uterine atony is the principal cause of PPH.
Risk factors for uterine atony include multiparity, multifetal pregnancy, fetal macrosomia, polyhydramnios, uterine fibroids, prolonged or precipitous labor, chorioamnionitis, and advanced maternal age.

## Slide 58
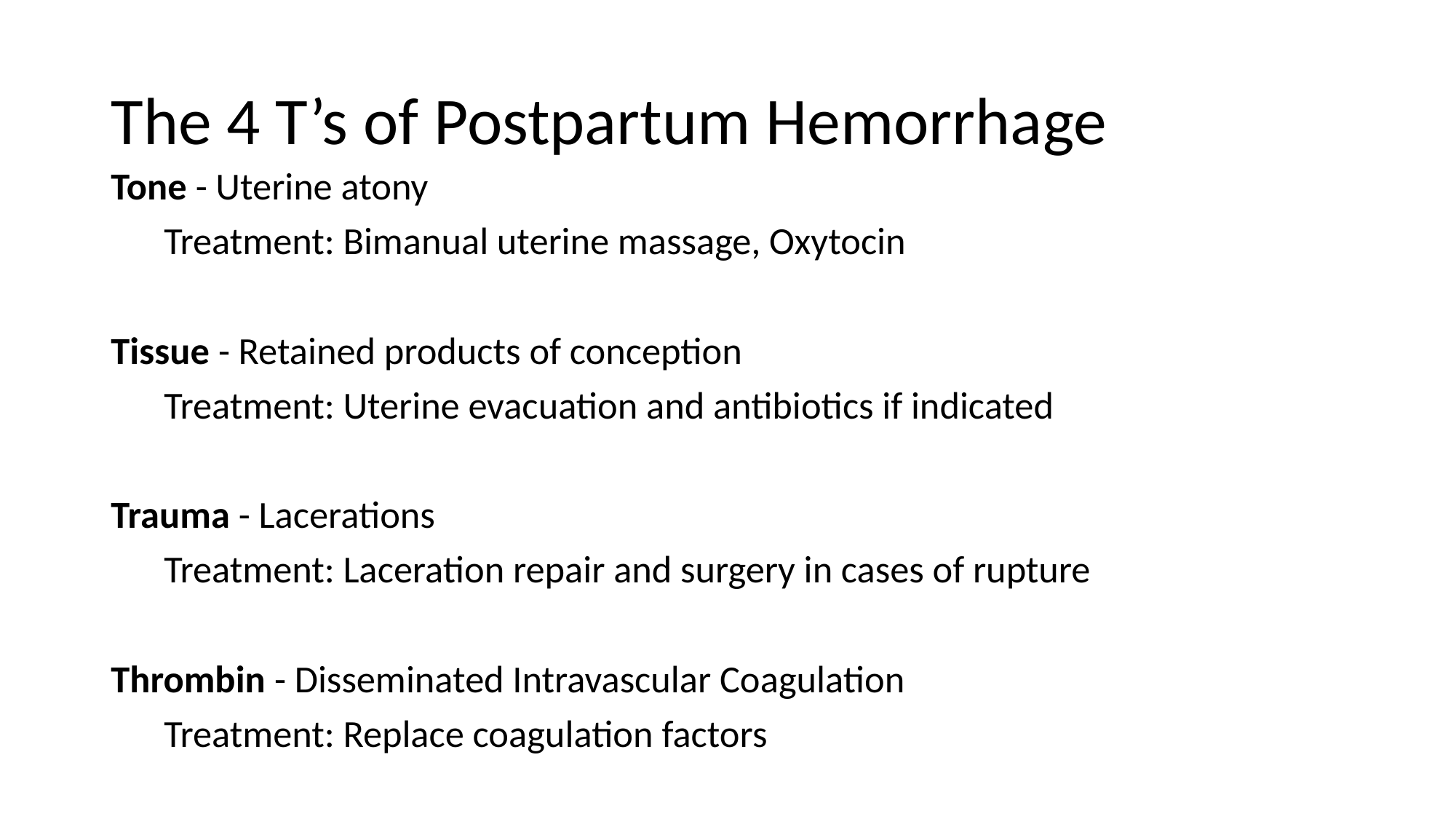

# The 4 T’s of Postpartum Hemorrhage
Tone - Uterine atony
Treatment: Bimanual uterine massage, Oxytocin
Tissue - Retained products of conception
Treatment: Uterine evacuation and antibiotics if indicated
Trauma - Lacerations
Treatment: Laceration repair and surgery in cases of rupture
Thrombin - Disseminated Intravascular Coagulation
Treatment: Replace coagulation factors

## Slide 59
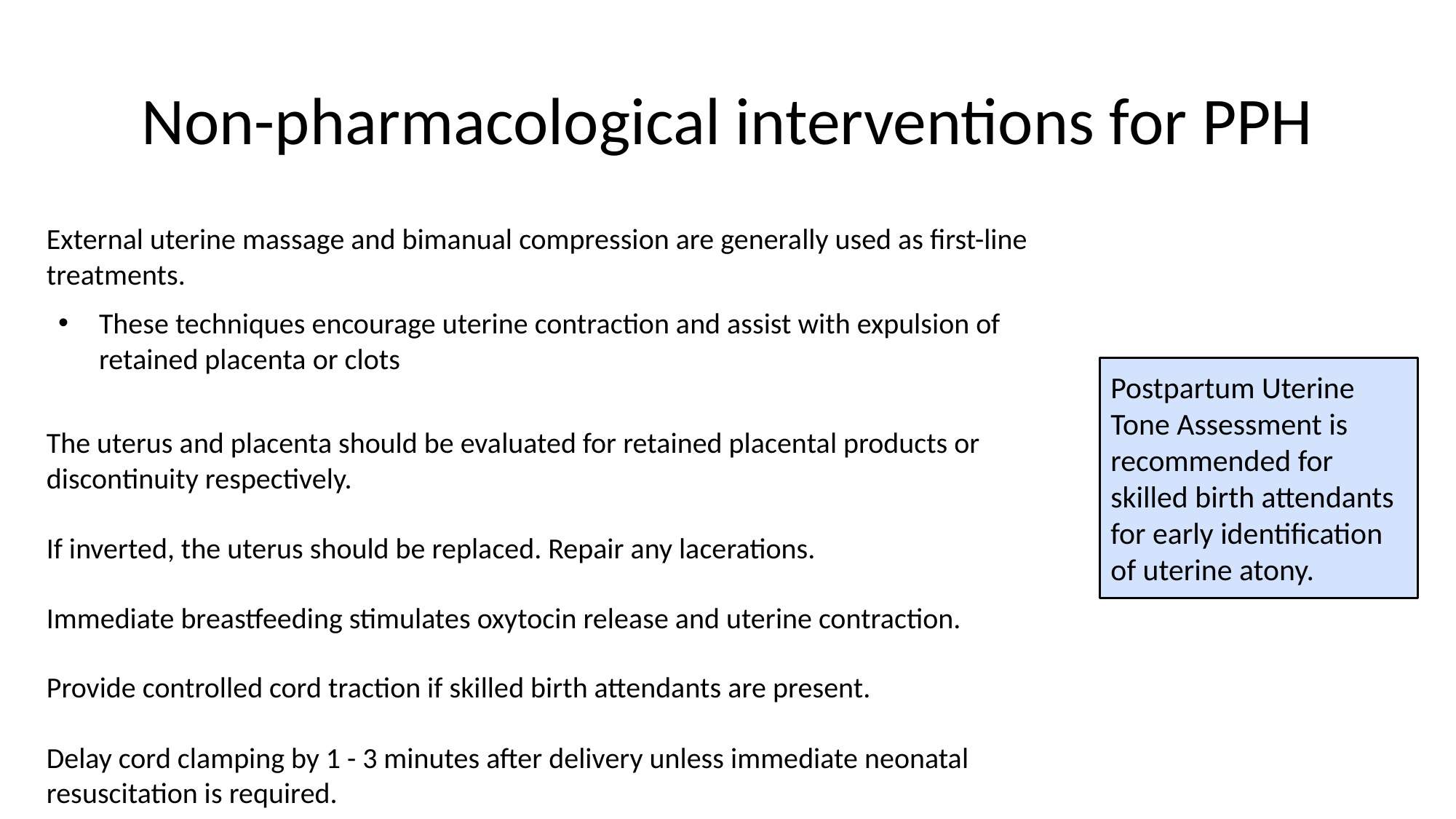

# Non-pharmacological interventions for PPH
External uterine massage and bimanual compression are generally used as first-line treatments.
These techniques encourage uterine contraction and assist with expulsion of retained placenta or clots
The uterus and placenta should be evaluated for retained placental products or discontinuity respectively.
If inverted, the uterus should be replaced. Repair any lacerations.
Immediate breastfeeding stimulates oxytocin release and uterine contraction.
Provide controlled cord traction if skilled birth attendants are present.
Delay cord clamping by 1 - 3 minutes after delivery unless immediate neonatal resuscitation is required.
Postpartum Uterine Tone Assessment is recommended for skilled birth attendants for early identification of uterine atony.

## Slide 60
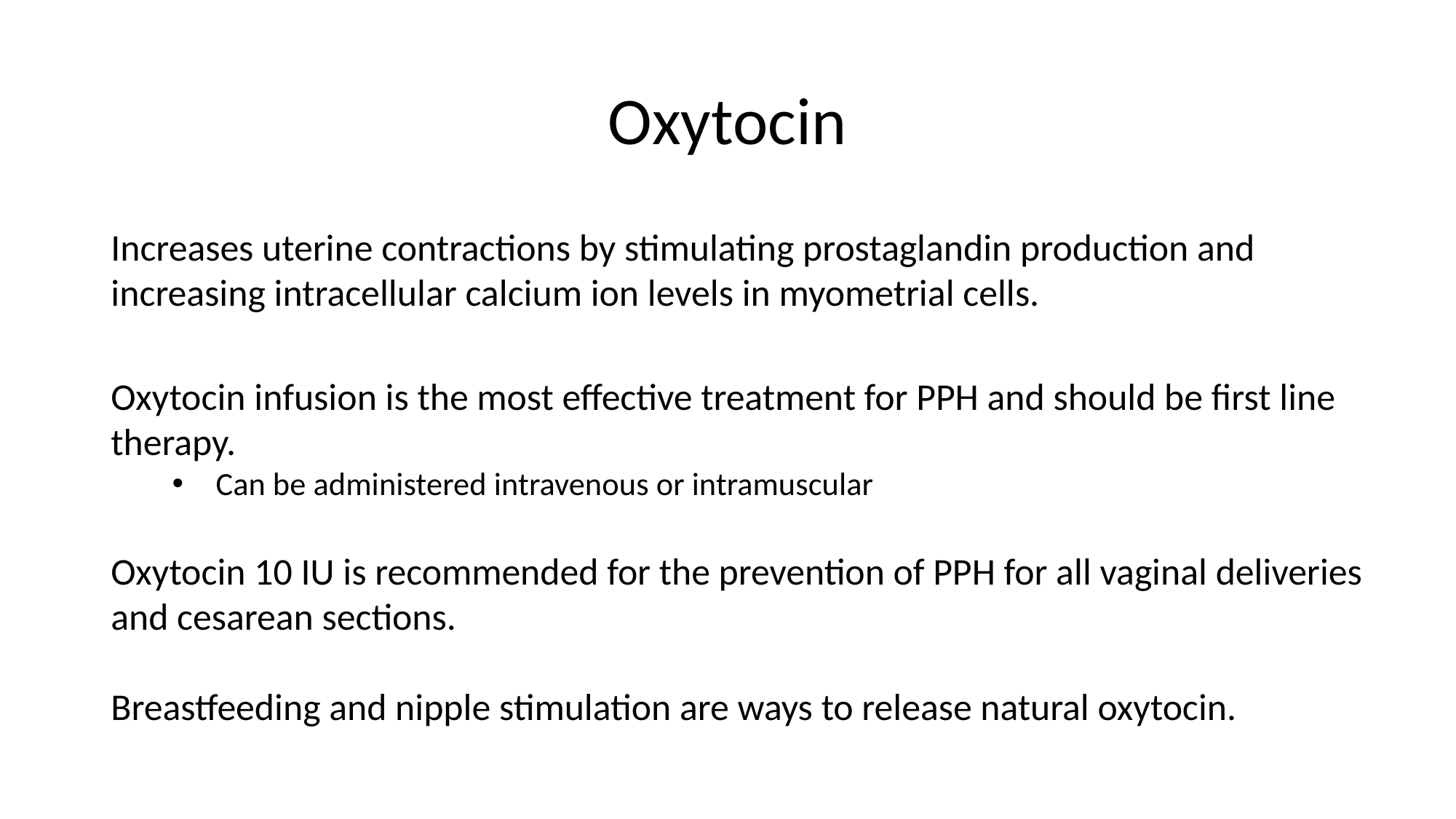

# Oxytocin
Increases uterine contractions by stimulating prostaglandin production and increasing intracellular calcium ion levels in myometrial cells.
Oxytocin infusion is the most effective treatment for PPH and should be first line therapy.
Can be administered intravenous or intramuscular
Oxytocin 10 IU is recommended for the prevention of PPH for all vaginal deliveries and cesarean sections.
Breastfeeding and nipple stimulation are ways to release natural oxytocin.

## Slide 61
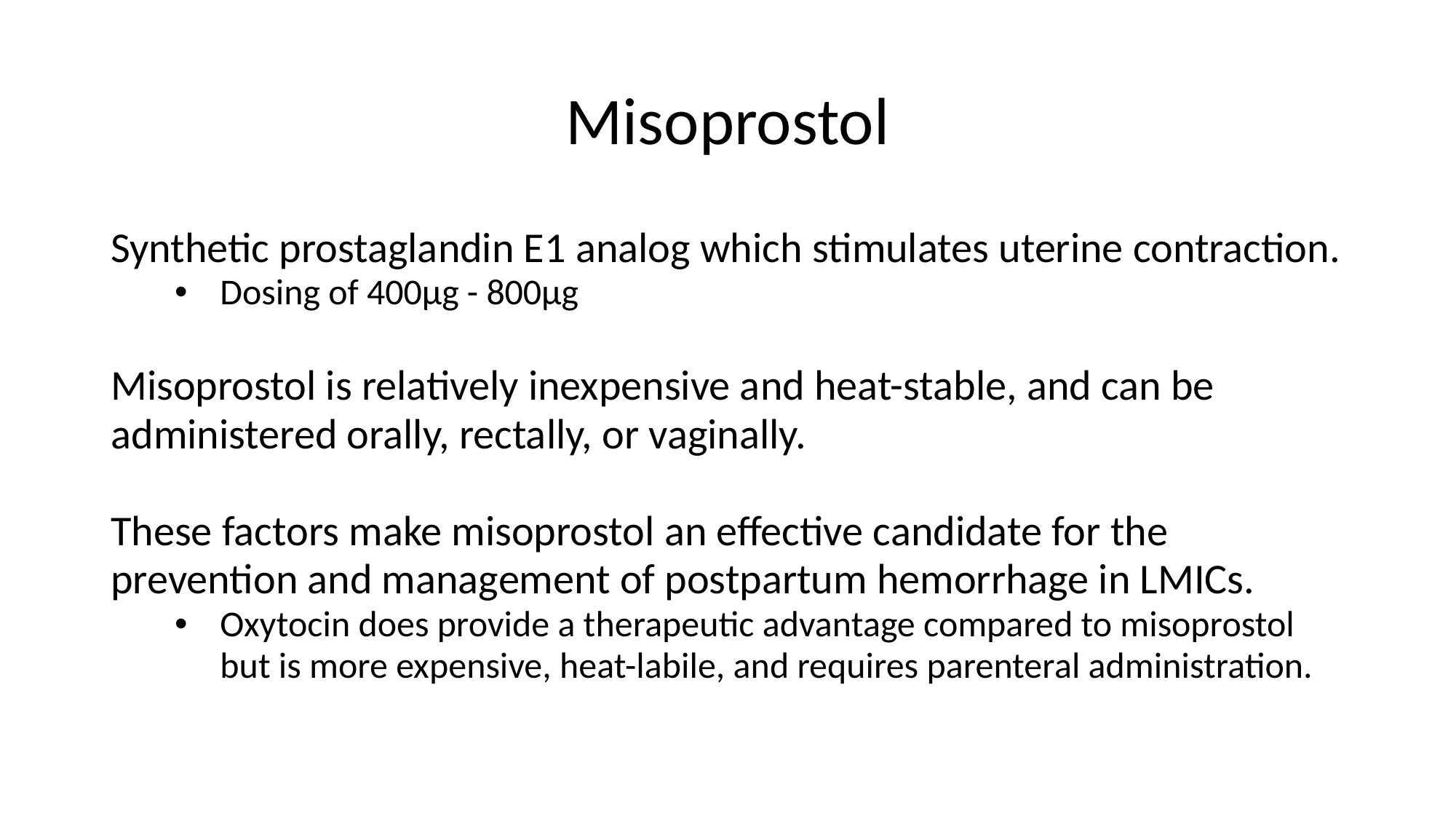

# Misoprostol
Synthetic prostaglandin E1 analog which stimulates uterine contraction.
Dosing of 400μg - 800μg
Misoprostol is relatively inexpensive and heat-stable, and can be administered orally, rectally, or vaginally.
These factors make misoprostol an effective candidate for the prevention and management of postpartum hemorrhage in LMICs.
Oxytocin does provide a therapeutic advantage compared to misoprostol but is more expensive, heat-labile, and requires parenteral administration.

## Slide 62
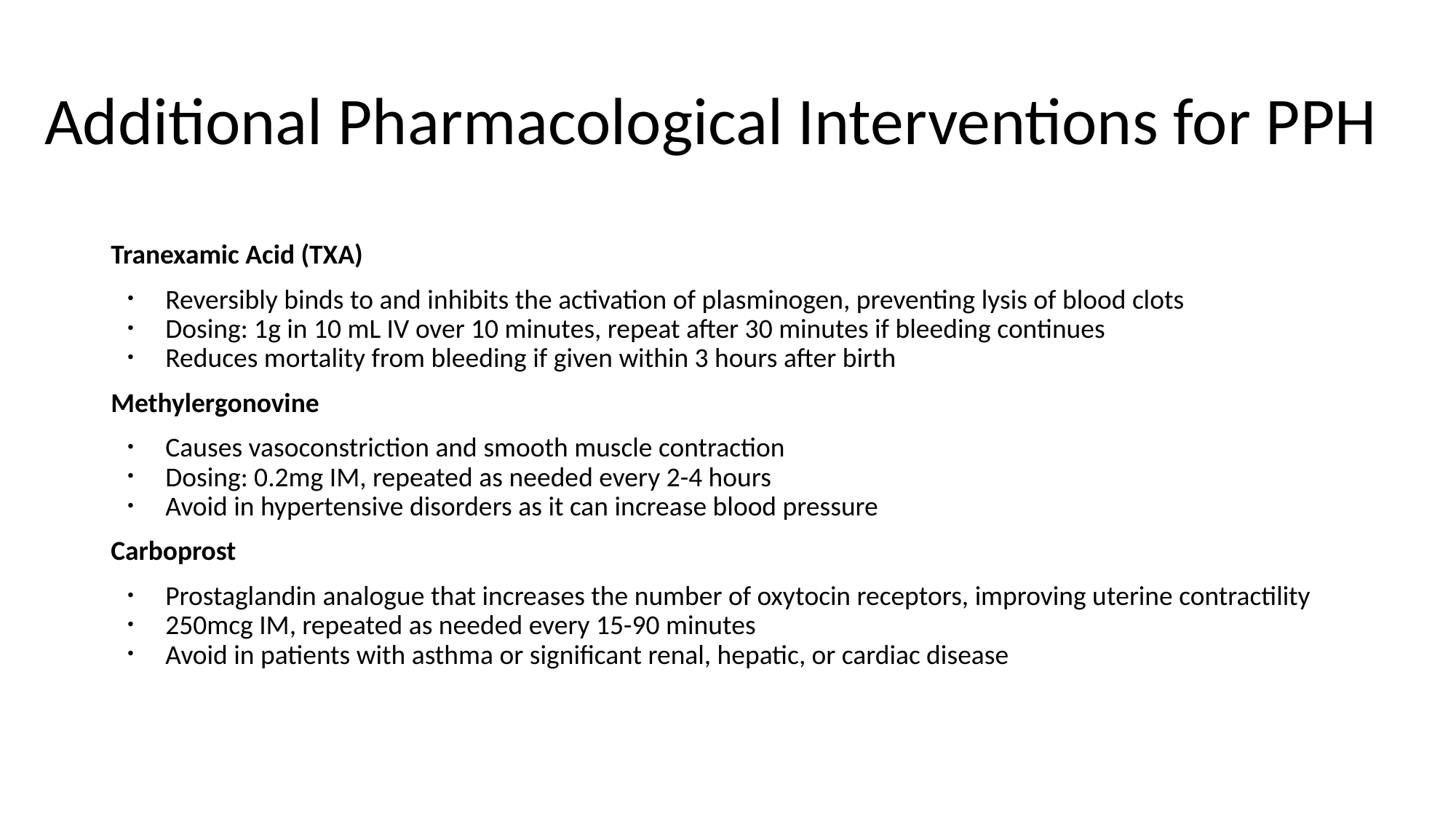

# Additional Pharmacological Interventions for PPH
Tranexamic Acid (TXA)
Reversibly binds to and inhibits the activation of plasminogen, preventing lysis of blood clots
Dosing: 1g in 10 mL IV over 10 minutes, repeat after 30 minutes if bleeding continues
Reduces mortality from bleeding if given within 3 hours after birth
Methylergonovine
Causes vasoconstriction and smooth muscle contraction
Dosing: 0.2mg IM, repeated as needed every 2-4 hours
Avoid in hypertensive disorders as it can increase blood pressure
Carboprost
Prostaglandin analogue that increases the number of oxytocin receptors, improving uterine contractility
250mcg IM, repeated as needed every 15-90 minutes
Avoid in patients with asthma or significant renal, hepatic, or cardiac disease

## Slide 63
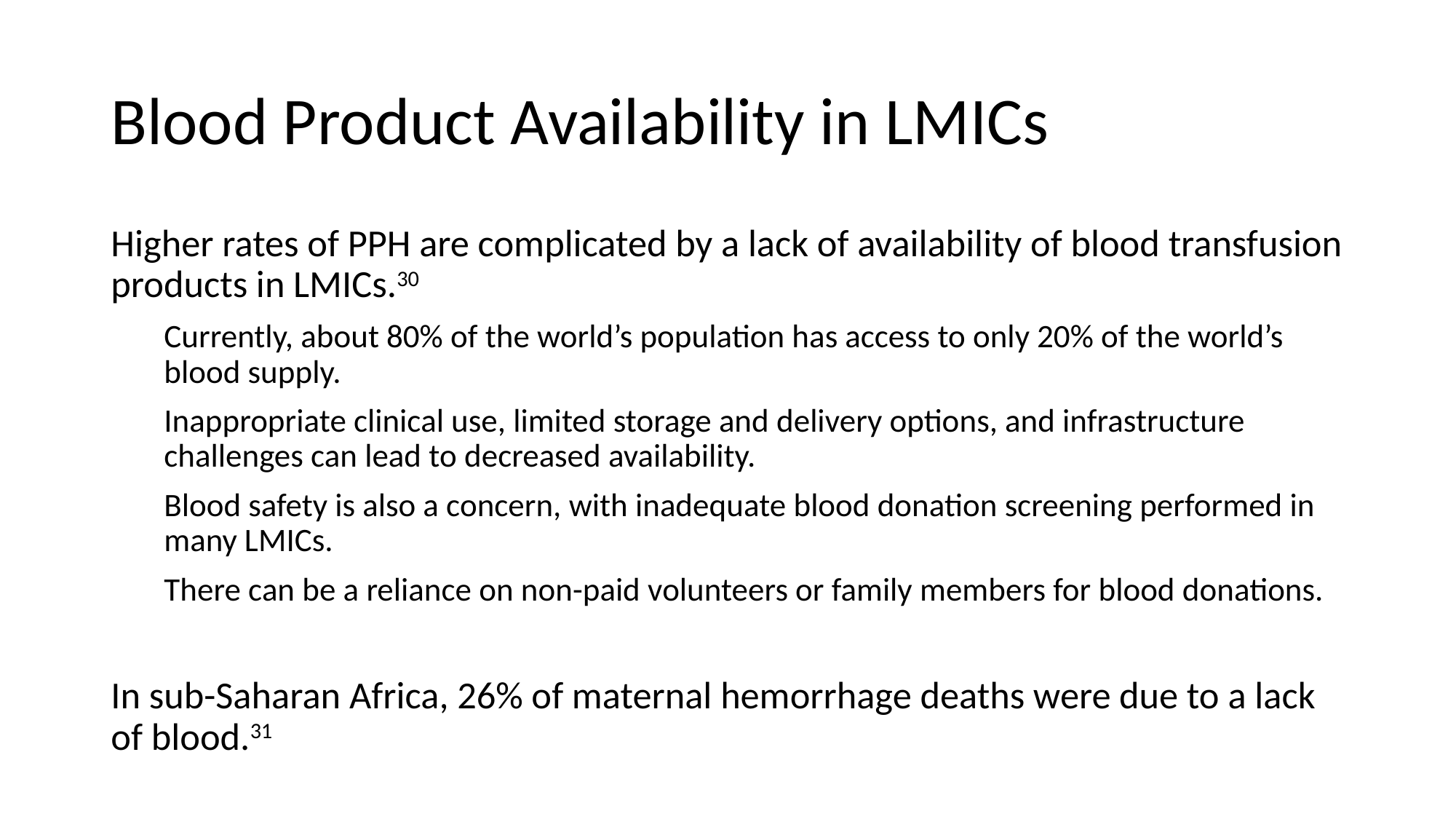

# Blood Product Availability in LMICs
Higher rates of PPH are complicated by a lack of availability of blood transfusion products in LMICs.30
Currently, about 80% of the world’s population has access to only 20% of the world’s blood supply.
Inappropriate clinical use, limited storage and delivery options, and infrastructure challenges can lead to decreased availability.
Blood safety is also a concern, with inadequate blood donation screening performed in many LMICs.
There can be a reliance on non-paid volunteers or family members for blood donations.
In sub-Saharan Africa, 26% of maternal hemorrhage deaths were due to a lack of blood.31

## Slide 64
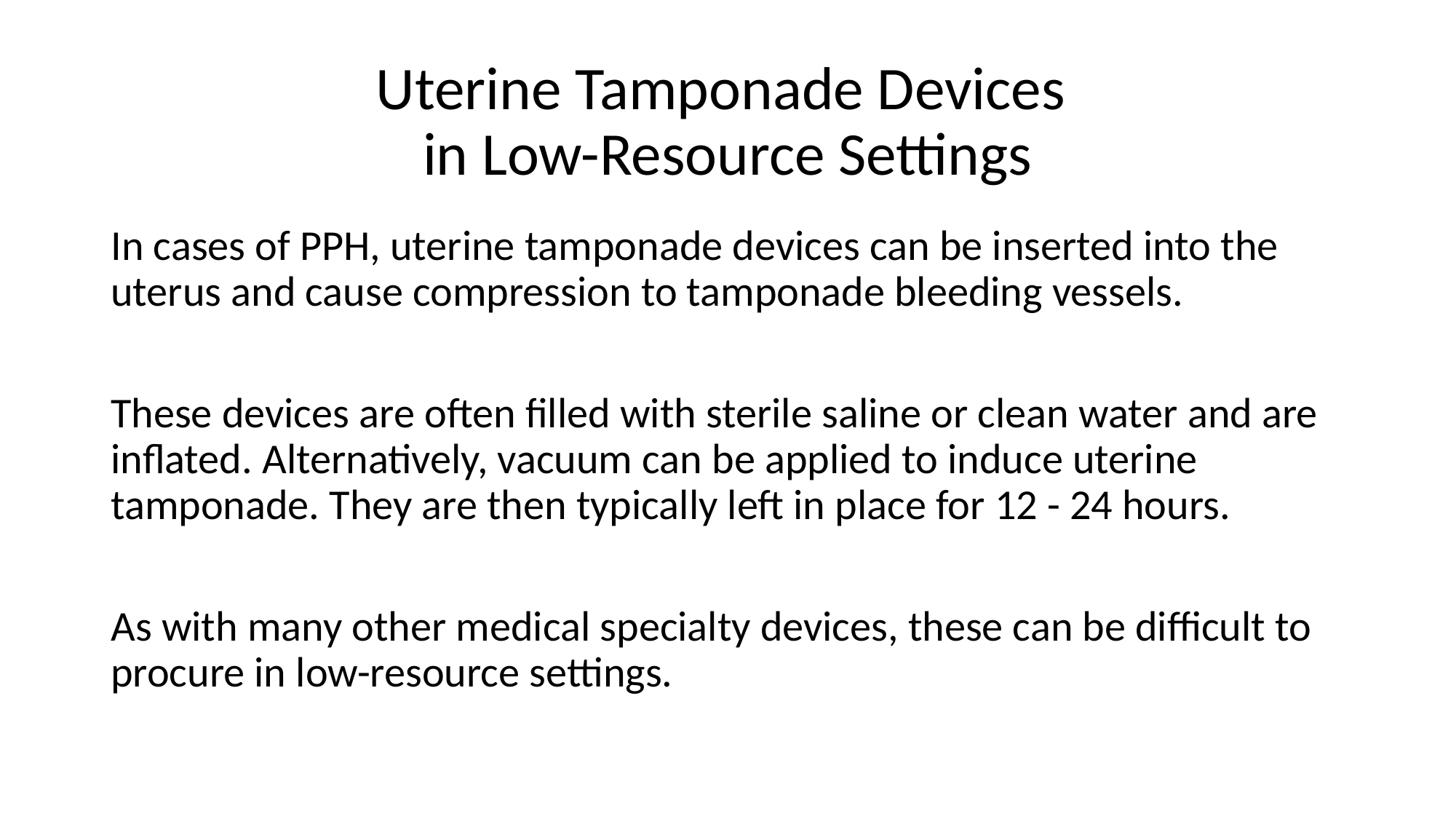

# Uterine Tamponade Devices
in Low-Resource Settings
In cases of PPH, uterine tamponade devices can be inserted into the uterus and cause compression to tamponade bleeding vessels.
These devices are often filled with sterile saline or clean water and are inflated. Alternatively, vacuum can be applied to induce uterine tamponade. They are then typically left in place for 12 - 24 hours.
As with many other medical specialty devices, these can be difficult to procure in low-resource settings.

## Slide 65
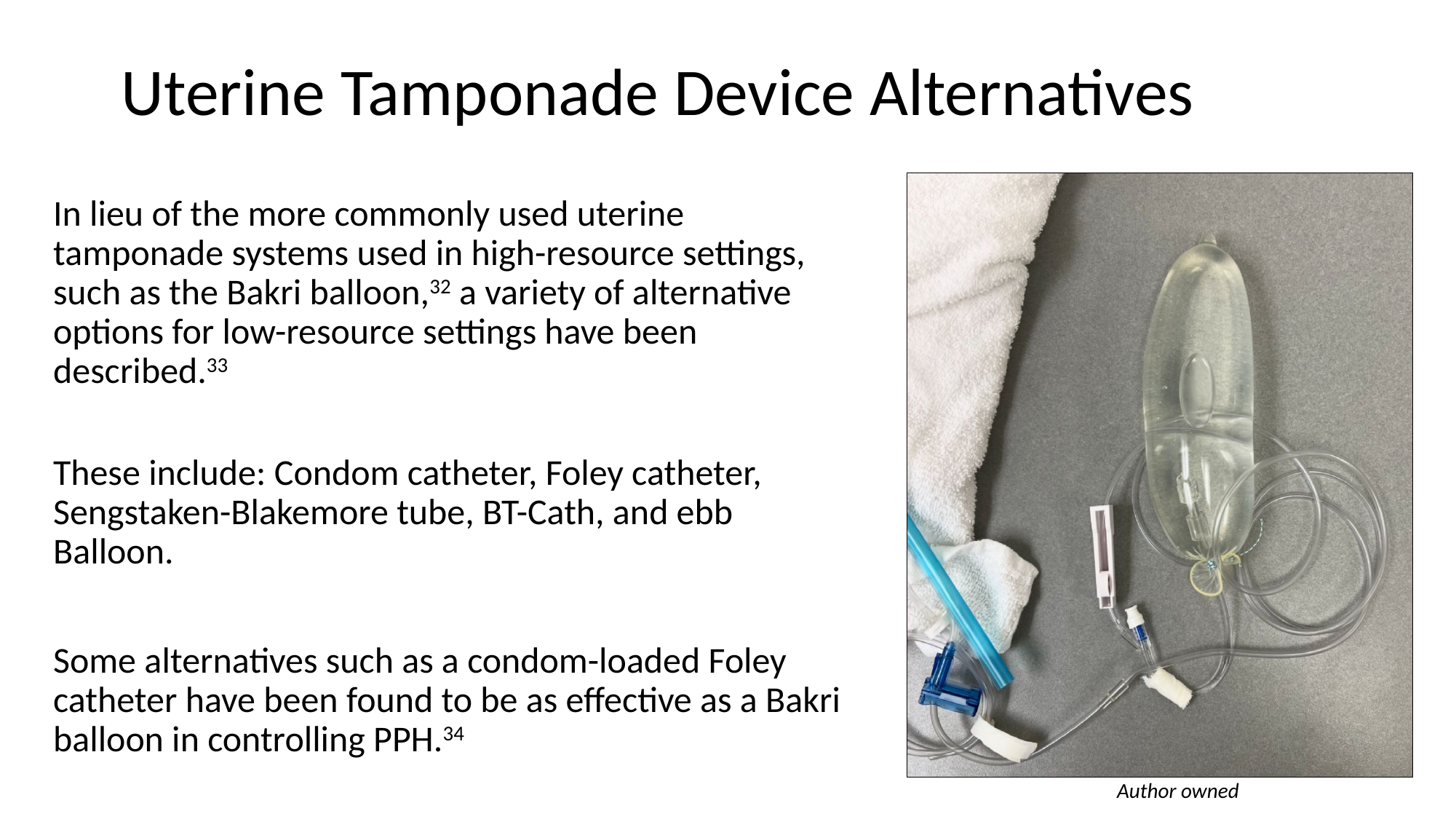

# Uterine Tamponade Device Alternatives
In lieu of the more commonly used uterine tamponade systems used in high-resource settings, such as the Bakri balloon,32 a variety of alternative options for low-resource settings have been described.33
These include: Condom catheter, Foley catheter, Sengstaken-Blakemore tube, BT-Cath, and ebb Balloon.
Some alternatives such as a condom-loaded Foley catheter have been found to be as effective as a Bakri balloon in controlling PPH.34
Author owned

## Slide 66
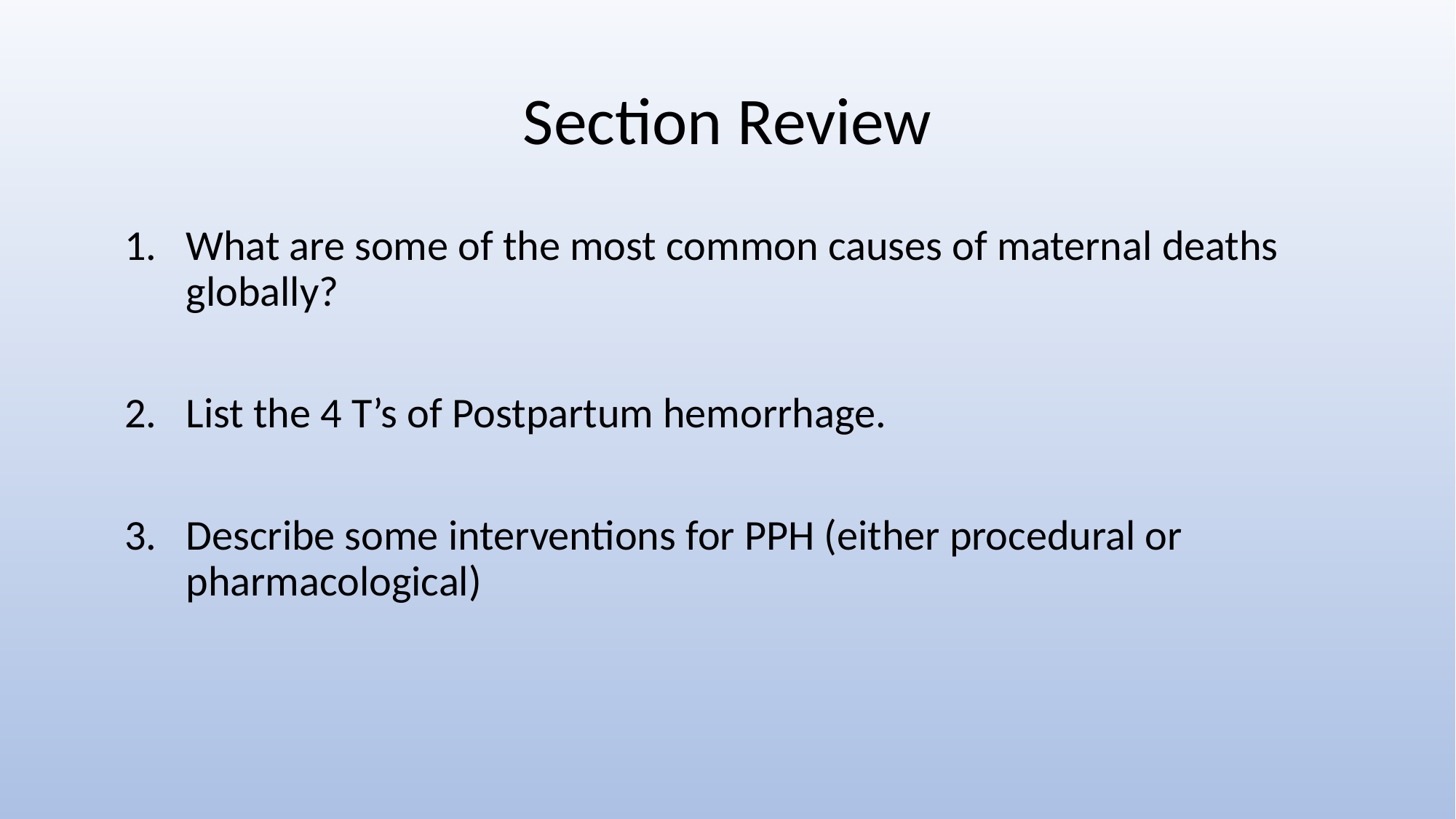

# Section Review
What are some of the most common causes of maternal deaths globally?
List the 4 T’s of Postpartum hemorrhage.
Describe some interventions for PPH (either procedural or pharmacological)

## Slide 67
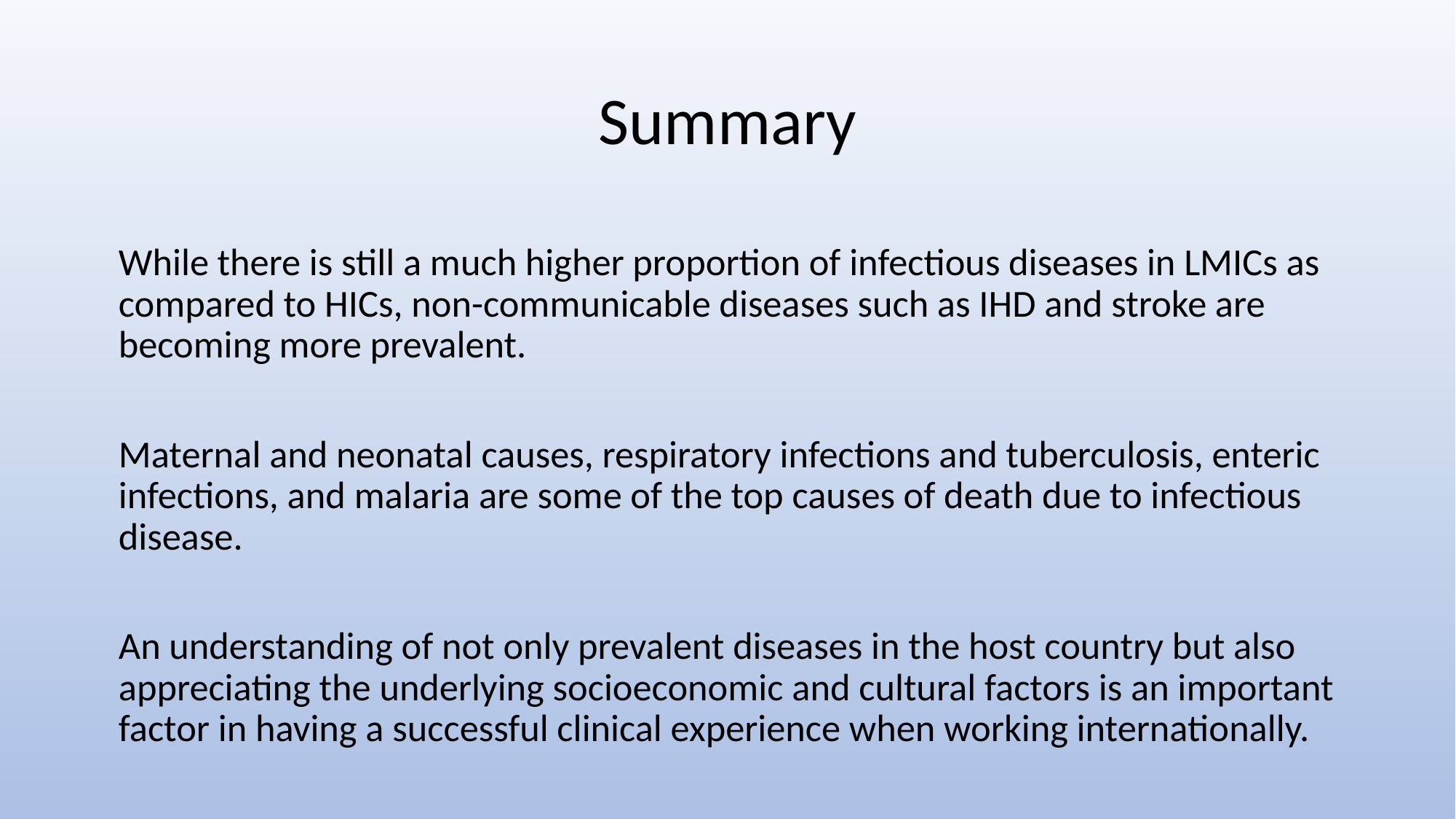

# Summary
While there is still a much higher proportion of infectious diseases in LMICs as compared to HICs, non-communicable diseases such as IHD and stroke are becoming more prevalent.
Maternal and neonatal causes, respiratory infections and tuberculosis, enteric infections, and malaria are some of the top causes of death due to infectious disease.
An understanding of not only prevalent diseases in the host country but also appreciating the underlying socioeconomic and cultural factors is an important factor in having a successful clinical experience when working internationally.

## Slide 68
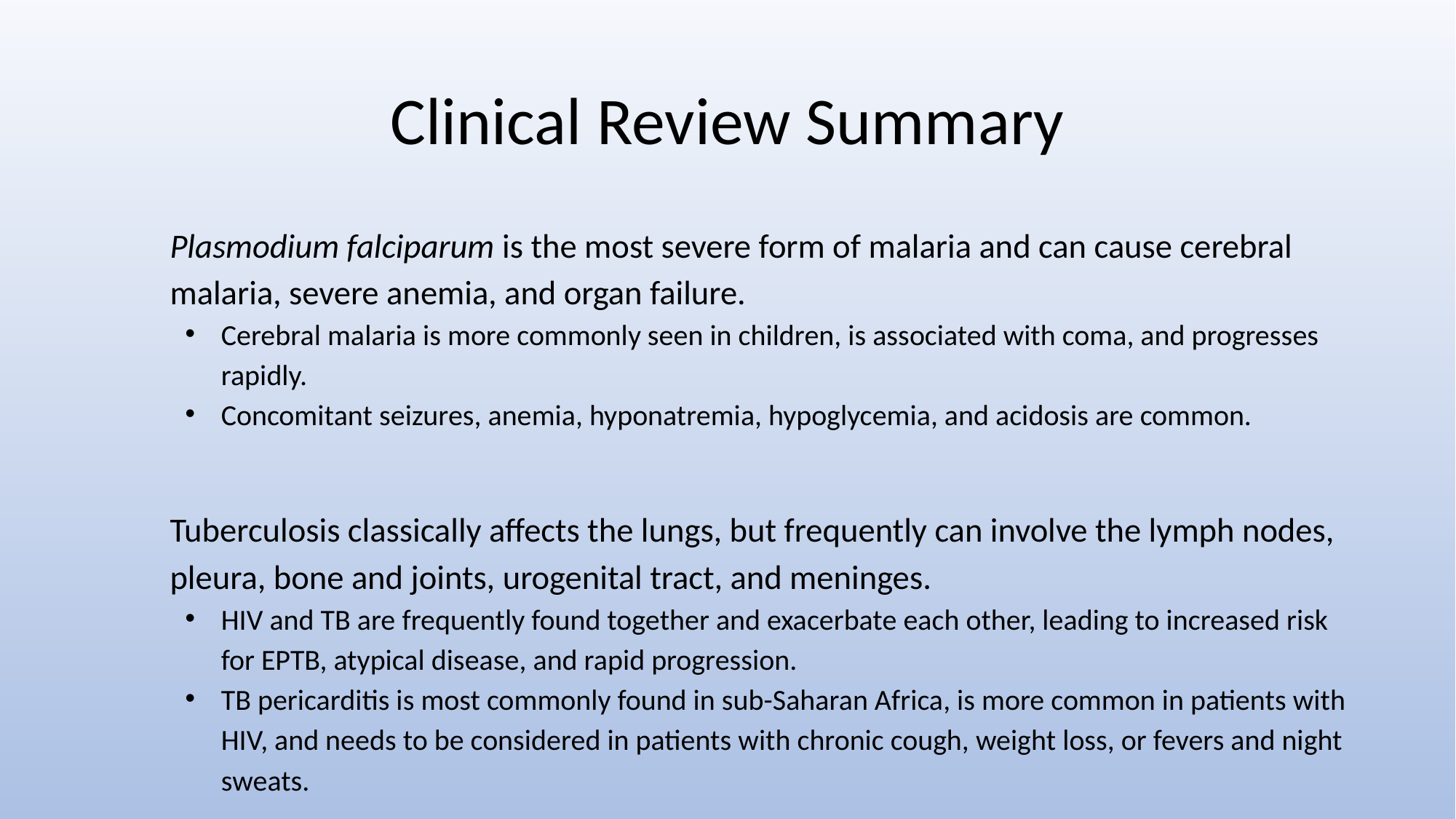

# Clinical Review Summary
Plasmodium falciparum is the most severe form of malaria and can cause cerebral malaria, severe anemia, and organ failure.
Cerebral malaria is more commonly seen in children, is associated with coma, and progresses rapidly.
Concomitant seizures, anemia, hyponatremia, hypoglycemia, and acidosis are common.
Tuberculosis classically affects the lungs, but frequently can involve the lymph nodes, pleura, bone and joints, urogenital tract, and meninges.
HIV and TB are frequently found together and exacerbate each other, leading to increased risk for EPTB, atypical disease, and rapid progression.
TB pericarditis is most commonly found in sub-Saharan Africa, is more common in patients with HIV, and needs to be considered in patients with chronic cough, weight loss, or fevers and night sweats.

## Slide 69
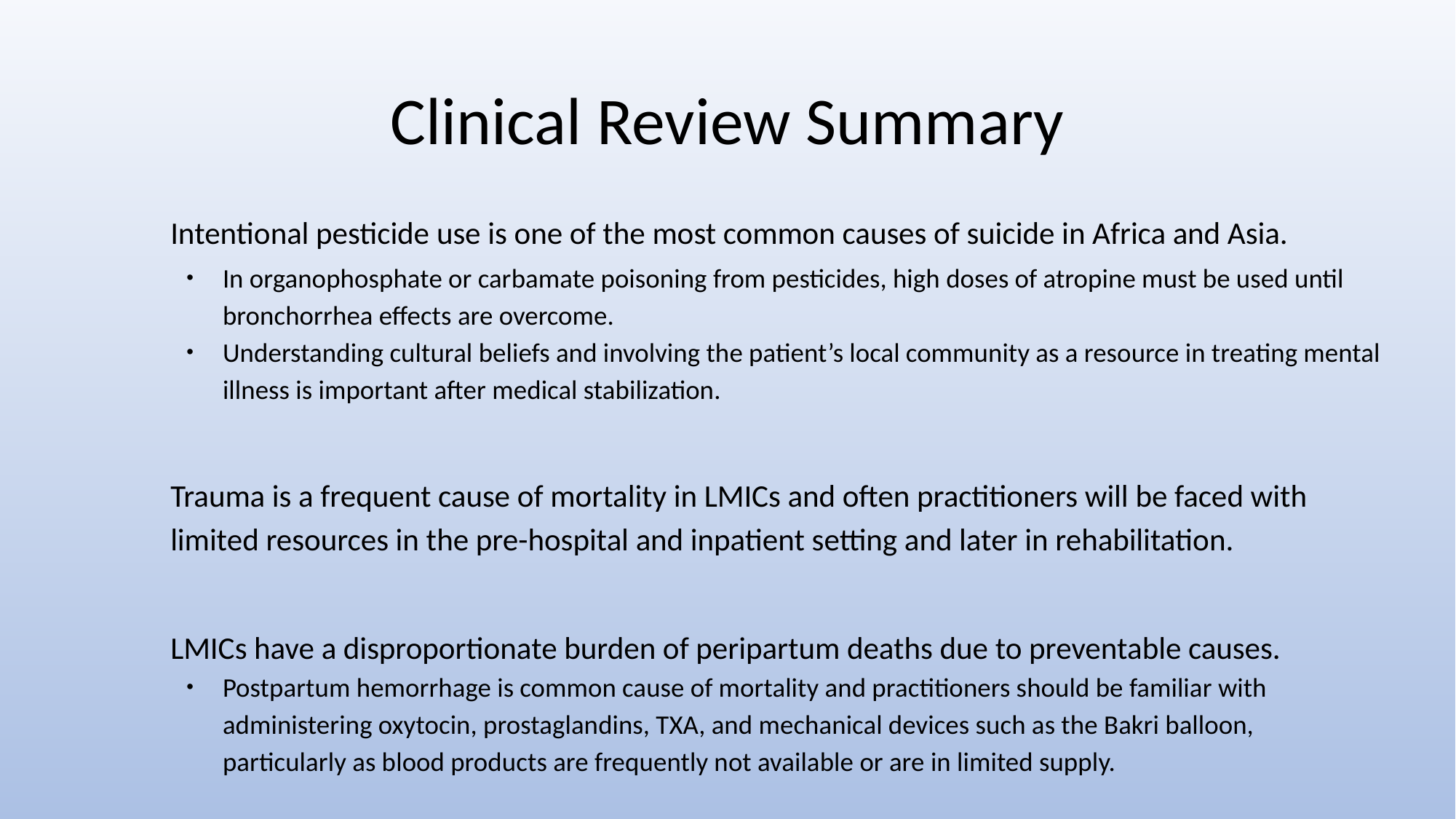

# Clinical Review Summary
Intentional pesticide use is one of the most common causes of suicide in Africa and Asia.
In organophosphate or carbamate poisoning from pesticides, high doses of atropine must be used until bronchorrhea effects are overcome.
Understanding cultural beliefs and involving the patient’s local community as a resource in treating mental illness is important after medical stabilization.
Trauma is a frequent cause of mortality in LMICs and often practitioners will be faced with limited resources in the pre-hospital and inpatient setting and later in rehabilitation.
LMICs have a disproportionate burden of peripartum deaths due to preventable causes.
Postpartum hemorrhage is common cause of mortality and practitioners should be familiar with administering oxytocin, prostaglandins, TXA, and mechanical devices such as the Bakri balloon, particularly as blood products are frequently not available or are in limited supply.

## Slide 70
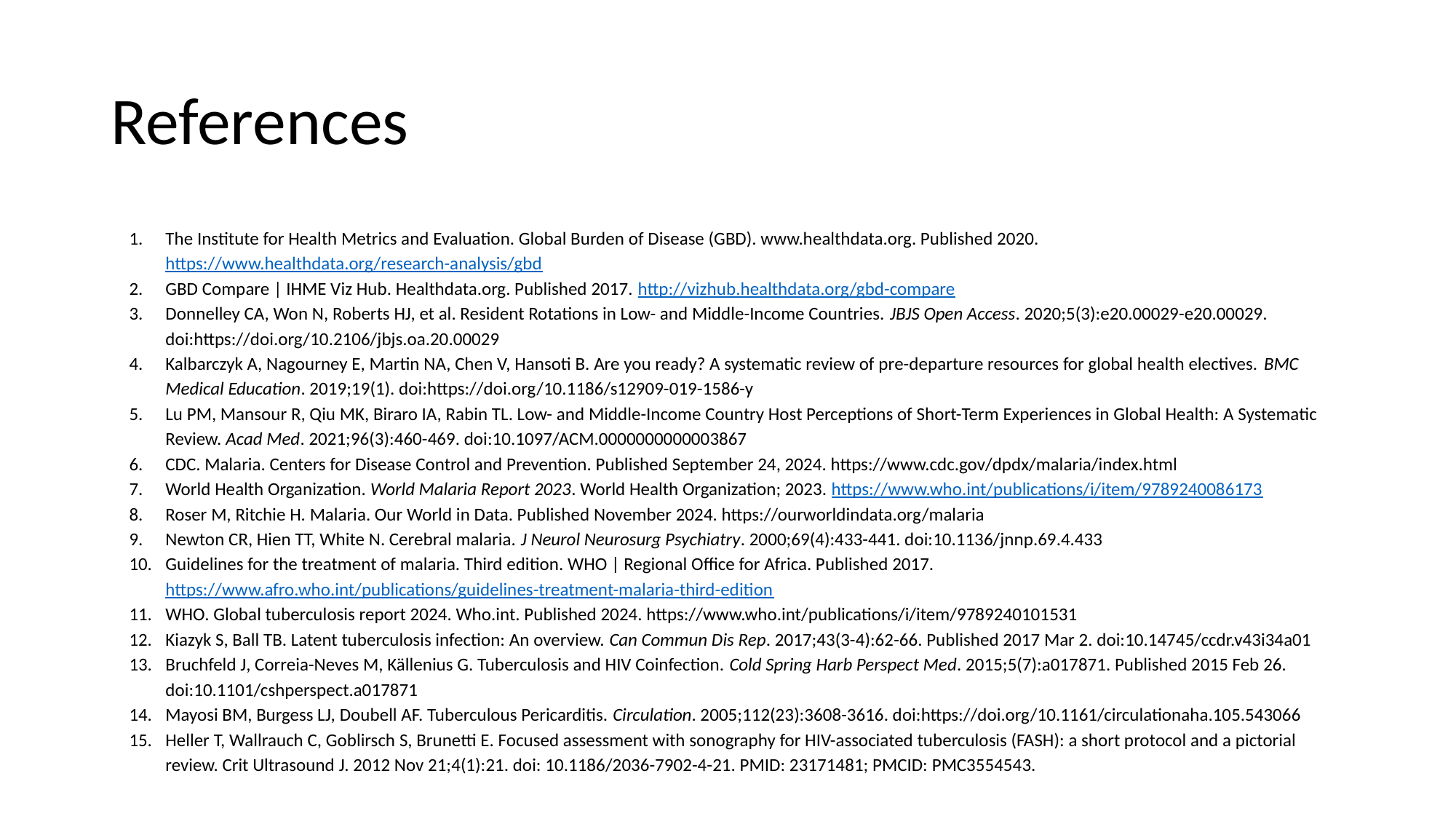

# References
The Institute for Health Metrics and Evaluation. Global Burden of Disease (GBD). www.healthdata.org. Published 2020. https://www.healthdata.org/research-analysis/gbd
GBD Compare | IHME Viz Hub. Healthdata.org. Published 2017. http://vizhub.healthdata.org/gbd-compare
Donnelley CA, Won N, Roberts HJ, et al. Resident Rotations in Low- and Middle-Income Countries. JBJS Open Access. 2020;5(3):e20.00029-e20.00029. doi:https://doi.org/10.2106/jbjs.oa.20.00029
‌Kalbarczyk A, Nagourney E, Martin NA, Chen V, Hansoti B. Are you ready? A systematic review of pre-departure resources for global health electives. BMC Medical Education. 2019;19(1). doi:https://doi.org/10.1186/s12909-019-1586-y
‌Lu PM, Mansour R, Qiu MK, Biraro IA, Rabin TL. Low- and Middle-Income Country Host Perceptions of Short-Term Experiences in Global Health: A Systematic Review. Acad Med. 2021;96(3):460-469. doi:10.1097/ACM.0000000000003867
CDC. Malaria. Centers for Disease Control and Prevention. Published September 24, 2024. https://www.cdc.gov/dpdx/malaria/index.html
‌World Health Organization. World Malaria Report 2023. World Health Organization; 2023. https://www.who.int/publications/i/item/9789240086173
‌Roser M, Ritchie H. Malaria. Our World in Data. Published November 2024. https://ourworldindata.org/malaria
‌Newton CR, Hien TT, White N. Cerebral malaria. J Neurol Neurosurg Psychiatry. 2000;69(4):433-441. doi:10.1136/jnnp.69.4.433
Guidelines for the treatment of malaria. Third edition. WHO | Regional Office for Africa. Published 2017. https://www.afro.who.int/publications/guidelines-treatment-malaria-third-edition
WHO. Global tuberculosis report 2024. Who.int. Published 2024. https://www.who.int/publications/i/item/9789240101531
‌Kiazyk S, Ball TB. Latent tuberculosis infection: An overview. Can Commun Dis Rep. 2017;43(3-4):62-66. Published 2017 Mar 2. doi:10.14745/ccdr.v43i34a01
Bruchfeld J, Correia-Neves M, Källenius G. Tuberculosis and HIV Coinfection. Cold Spring Harb Perspect Med. 2015;5(7):a017871. Published 2015 Feb 26. doi:10.1101/cshperspect.a017871
Mayosi BM, Burgess LJ, Doubell AF. Tuberculous Pericarditis. Circulation. 2005;112(23):3608-3616. doi:https://doi.org/10.1161/circulationaha.105.543066
‌Heller T, Wallrauch C, Goblirsch S, Brunetti E. Focused assessment with sonography for HIV-associated tuberculosis (FASH): a short protocol and a pictorial review. Crit Ultrasound J. 2012 Nov 21;4(1):21. doi: 10.1186/2036-7902-4-21. PMID: 23171481; PMCID: PMC3554543.

## Slide 71
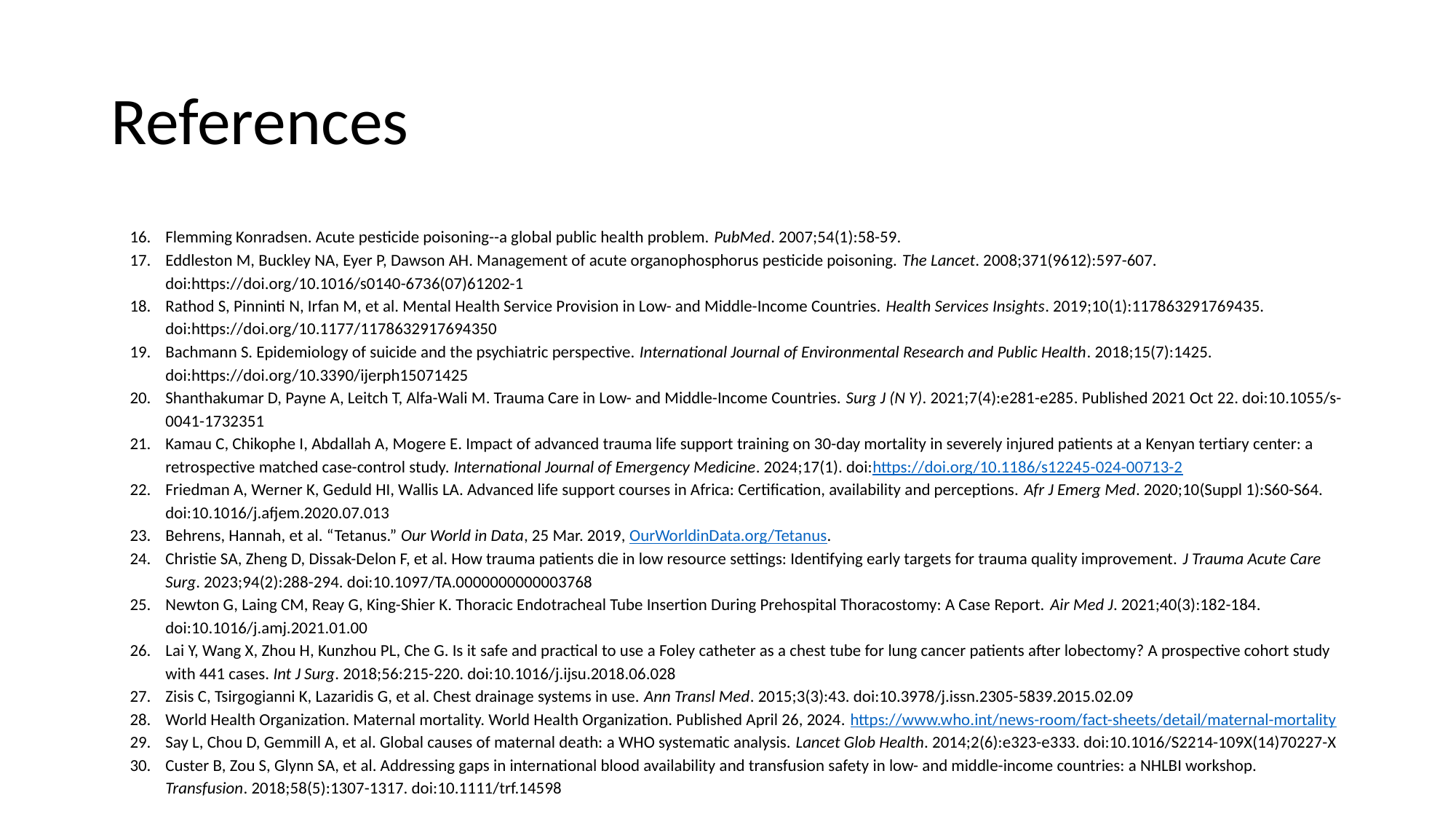

# References
Flemming Konradsen. Acute pesticide poisoning--a global public health problem. PubMed. 2007;54(1):58-59.
Eddleston M, Buckley NA, Eyer P, Dawson AH. Management of acute organophosphorus pesticide poisoning. The Lancet. 2008;371(9612):597-607. doi:https://doi.org/10.1016/s0140-6736(07)61202-1
Rathod S, Pinninti N, Irfan M, et al. Mental Health Service Provision in Low- and Middle-Income Countries. Health Services Insights. 2019;10(1):117863291769435. doi:https://doi.org/10.1177/1178632917694350
Bachmann S. Epidemiology of suicide and the psychiatric perspective. International Journal of Environmental Research and Public Health. 2018;15(7):1425. doi:https://doi.org/10.3390/ijerph15071425
Shanthakumar D, Payne A, Leitch T, Alfa-Wali M. Trauma Care in Low- and Middle-Income Countries. Surg J (N Y). 2021;7(4):e281-e285. Published 2021 Oct 22. doi:10.1055/s-0041-1732351
Kamau C, Chikophe I, Abdallah A, Mogere E. Impact of advanced trauma life support training on 30-day mortality in severely injured patients at a Kenyan tertiary center: a retrospective matched case-control study. International Journal of Emergency Medicine. 2024;17(1). doi:https://doi.org/10.1186/s12245-024-00713-2
Friedman A, Werner K, Geduld HI, Wallis LA. Advanced life support courses in Africa: Certification, availability and perceptions. Afr J Emerg Med. 2020;10(Suppl 1):S60-S64. doi:10.1016/j.afjem.2020.07.013
Behrens, Hannah, et al. “Tetanus.” Our World in Data, 25 Mar. 2019, OurWorldinData.org/Tetanus.
Christie SA, Zheng D, Dissak-Delon F, et al. How trauma patients die in low resource settings: Identifying early targets for trauma quality improvement. J Trauma Acute Care Surg. 2023;94(2):288-294. doi:10.1097/TA.0000000000003768
Newton G, Laing CM, Reay G, King-Shier K. Thoracic Endotracheal Tube Insertion During Prehospital Thoracostomy: A Case Report. Air Med J. 2021;40(3):182-184. doi:10.1016/j.amj.2021.01.00
Lai Y, Wang X, Zhou H, Kunzhou PL, Che G. Is it safe and practical to use a Foley catheter as a chest tube for lung cancer patients after lobectomy? A prospective cohort study with 441 cases. Int J Surg. 2018;56:215-220. doi:10.1016/j.ijsu.2018.06.028
Zisis C, Tsirgogianni K, Lazaridis G, et al. Chest drainage systems in use. Ann Transl Med. 2015;3(3):43. doi:10.3978/j.issn.2305-5839.2015.02.09
World Health Organization. Maternal mortality. World Health Organization. Published April 26, 2024. https://www.who.int/news-room/fact-sheets/detail/maternal-mortality
Say L, Chou D, Gemmill A, et al. Global causes of maternal death: a WHO systematic analysis. Lancet Glob Health. 2014;2(6):e323-e333. doi:10.1016/S2214-109X(14)70227-X
Custer B, Zou S, Glynn SA, et al. Addressing gaps in international blood availability and transfusion safety in low- and middle-income countries: a NHLBI workshop. Transfusion. 2018;58(5):1307-1317. doi:10.1111/trf.14598

## Slide 72
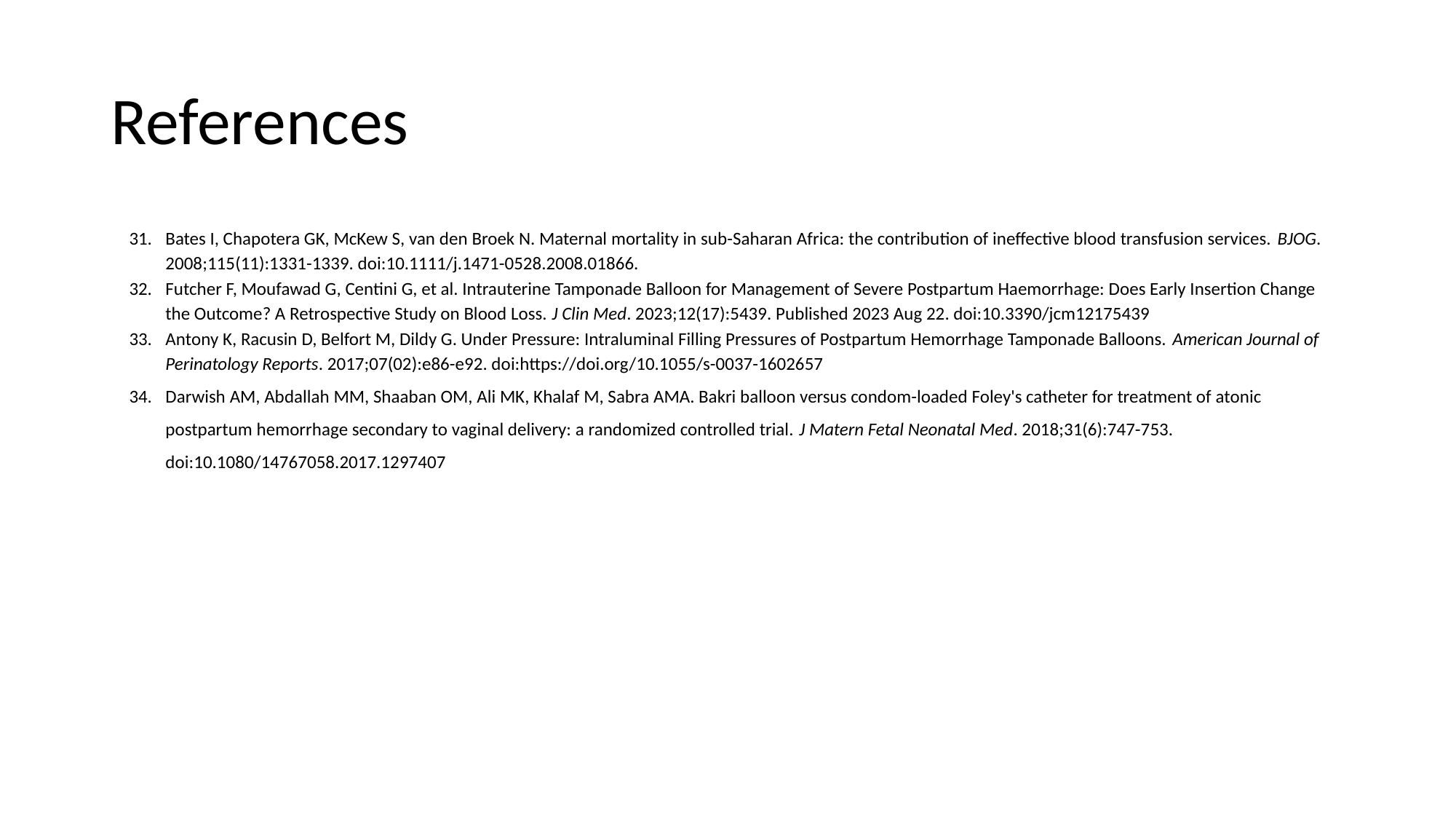

# References
Bates I, Chapotera GK, McKew S, van den Broek N. Maternal mortality in sub-Saharan Africa: the contribution of ineffective blood transfusion services. BJOG. 2008;115(11):1331-1339. doi:10.1111/j.1471-0528.2008.01866.
Futcher F, Moufawad G, Centini G, et al. Intrauterine Tamponade Balloon for Management of Severe Postpartum Haemorrhage: Does Early Insertion Change the Outcome? A Retrospective Study on Blood Loss. J Clin Med. 2023;12(17):5439. Published 2023 Aug 22. doi:10.3390/jcm12175439
Antony K, Racusin D, Belfort M, Dildy G. Under Pressure: Intraluminal Filling Pressures of Postpartum Hemorrhage Tamponade Balloons. American Journal of Perinatology Reports. 2017;07(02):e86-e92. doi:https://doi.org/10.1055/s-0037-1602657
Darwish AM, Abdallah MM, Shaaban OM, Ali MK, Khalaf M, Sabra AMA. Bakri balloon versus condom-loaded Foley's catheter for treatment of atonic postpartum hemorrhage secondary to vaginal delivery: a randomized controlled trial. J Matern Fetal Neonatal Med. 2018;31(6):747-753. doi:10.1080/14767058.2017.1297407
