# Supplementary material for: Advancing Global Health Education: Preparing Emergency Medicine Trainees for Low-Resource Settings Through Simulation-Based Training
Source: MedEdPORTAL. 2026 Mar 10;22:11582. doi: 10.15766/mep_2374-8265.11582 (PMC12972016; doi:10.15766/mep_2374-8265.11582)
Supplement: Supplementary file 1 — Equipment for Implementation.docxTraumatic Hemopneumothorax Case.docxTuberculous Pericarditis Case.docxCerebral Malaria Case.docxOrganophosphate Poisoning Case.docxPostpartum Hemorrhage Case.docxLecture.pptxCourse Evaluation.docx [file mep_2374-8265.11582-s001.zip › H. Course Evaluation.docx]

You are being asked to complete this survey as part of a research study. The purpose of the study is to determine how simulation cases like the ones you participated in today impact your perceptions about working in a resource-limited setting. By completing the survey, you are consenting to have your answers used in a journal publication. This survey is anonymous. You do not have to complete the survey and can stop answering questions at any time. If you choose not to complete this survey there will not be a negative impact on your grade for the rotation or negative impact on your standing in the residency program.

**Post-Participation Survey**

1. What is your level of training?

- PGY-1
- PGY-2
- PGY-3
- MS III
- MS IV

1. Have you participated in a global health elective during residency?

- Yes
- No
- N/A

1. Did you have any international/global health experiences during medical school?

- Yes
- No

1. If you have not yet participated in a global health elective during residency, do you plan to do so?

- Yes
- No

1. **Before** today’s activity, had you ever been involved in the care of a patient with the following conditions:

|  | **YES** | **NO** |
| --- | --- | --- |
| Organophosphate Poisoning |  |  |
| Malaria |  |  |
|  |  |  |
| Pulmonary Tuberculosis |  |  |
| Extrapulmonary Tuberculosis |  |  |
| Postpartum Hemorrhage |  |  |
| Tension Pneumothorax |  |  |

1. This activity increased my confidence in diagnosing and managing patients with **organophosphate poisoning**.
   1. Strongly Disagree
   2. Disagree
   3. Neither agree or disagree
   4. Agree
   5. Strongly Agree
2. This activity increased my confidence in diagnosing and managing patients with **malaria**.
   1. Strongly Disagree
   2. Disagree
   3. Neither agree or disagree
   4. Agree
   5. Strongly Agree
3. This activity increased my confidence in diagnosing and managing patients with **pulmonary tuberculosis**.
   1. Strongly Disagree
   2. Disagree
   3. Neither agree or disagree
   4. Agree
   5. Strongly Agree
4. This activity increased my confidence in diagnosing and managing patients with **extrapulmonary tuberculosis**.
   1. Strongly Disagree
   2. Disagree
   3. Neither agree or disagree
   4. Agree
   5. Strongly Agree
5. This activity increased my confidence in diagnosing and managing patients with **postpartum hemorrhage**.
   1. Strongly Disagree
   2. Disagree
   3. Neither agree or disagree
   4. Agree
   5. Strongly Agree
6. This activity increased my confidence in diagnosing and managing patients with **tension pneumothorax.**
   1. Strongly Disagree
   2. Disagree
   3. Neither agree or disagree
   4. Agree
   5. Strongly Agree
7. This activity increased my confidence in identifying alternative solutions to manage life-threatening emergencies when standard medications/equipment is not available.
   1. Strongly Disagree
   2. Disagree
   3. Neither agree or disagree
   4. Agree
   5. Strongly Agree
8. This activity increased my confidence in my ability to practice emergency medicine in resource-limited settings.
   1. Strongly Disagree
   2. Disagree
   3. Neither agree or disagree
   4. Agree
   5. Strongly Agree
9. Global health simulation cases are important to my education.
   1. Strongly Disagree
   2. Disagree
   3. Neither agree or disagree
   4. Agree
   5. Strongly Agree
10. The residency program should continue to include global health simulation cases in the conference schedule each year.
    1. Strongly Disagree
    2. Disagree
    3. Neither agree or disagree
    4. Agree
    5. Strongly Agree
11. The simulation cases presented today were realistic.
    1. Strongly Disagree
    2. Disagree
    3. Neither agree or disagree
    4. Agree
    5. Strongly Agree
12. The training met the stated objectives.
    1. Strongly Disagree
    2. Disagree
    3. Neither agree or disagree
    4. Agree
    5. Strongly Agree

Comments:
